# Supplementary material for: Pediatric Resident Education in Pulmonary (PREP): A Subspecialty Preparatory Boot Camp Curriculum for Pediatric Residents
Source: MedEdPORTAL. 2021 Jan 7;17:11066. doi: 10.15766/mep_2374-8265.11066 (PMC7809931; doi:10.15766/mep_2374-8265.11066)
Supplement: Supplementary file 1 — Example Agenda.docxOrientation Template.pptxIntroduction to Tracheostomies and Ventilators.pptxCystic Fibrosis JeoPARODY.pptxIntroduction to Airway Clearance and Lung Expansion.pptxInstructor Guide CPT.docxInstructor Guide IS.docxInstructor Guide PEP.docxInstructor Guide PAP.docxInstructor Guide OPEP.docxInstructor Guide Insufflator Exsufflator.docxInstructor Guide HFCWO.docxInstructor Guide IPV.docxPREP Day of Evaluation.docxPREP End of Rotation Evaluation.docxPREP Faculty Feedback Survey.docxPREP Focus Group Guide.docx [file mep_2374-8265.11066-s001.zip › _Educational_Summary_Report_11066.pdf]

MedEdPORTAL® | The AAMC Journal of Teaching and Learning Resources

Original Publication Open Access

Pediatric Resident Education in Pulmonary (PREP): A Subspecialty Preparatory Boot Camp Curriculum for Pediatric Residents

Erin K. Khan, MD*, Deborah R. Liptzin, MD, Joyce Baker, MBA, RRT-NPS, Maxene Meier, MS, Christopher D. Baker, MD, Tai M. Lockspeiser, MD, MHPE

*Corresponding author: ekhan@cmh.edu

Abstract

Introduction: Medical errors can occur any time resident physicians transition between rotations, especially to unfamiliar areas such as subspecialty pediatrics. To combat this, we created and implemented the pediatric resident education in pulmonary (PREP) boot camp using Kern's six-step approach to curriculum development. **Methods:** PREP was a 5-hour session with multiple high-yield components held on the first day of each new rotation, aimed to prepare residents to care for complex pulmonary inpatients, including those with tracheostomy and ventilator dependence, asthma, and cystic fibrosis. The curriculum was evaluated at multiple time points through surveys of residents and faculty and two formal resident focus group sessions. **Results:** PREP was successfully implemented in July 2018 with continued monthly sessions held. Thirty-five residents participated in the first year. Resident perceived preparedness and confidence in taking call duties increased significantly following PREP. All residents rated PREP as *extremely helpful* or *very helpful*, the highest ratings possible. Overall, residents preferred active learning strategies. All qualitative data revealed positive effects of PREP. Clinical faculty in the pulmonology division found PREP similarly helpful and felt that PREP better prepared residents to provide care to pulmonary inpatients than our previous model. **Discussion:** Our monthly preparatory boot camp on the first day of residents' inpatient pulmonary rotation has improved resident experience, preparedness, and ability to care for complex pulmonary patients. The curriculum was adjusted in response to feedback to increase hands-on time and interactive sessions. Protected time for residents and active learning strategies were key to success of PREP.

Keywords
Boot Camp, Tracheostomy, Cystic Fibrosis, Airway Clearance, Quality Improvement/Patient Safety, Pediatric Pulmonology, Case-Based Learning, Clinical Teaching/Bedside Teaching

Educational Objective

By the end of the pediatric resident education in pulmonary boot camp, pediatric residents will be able to advance their knowledge, ability, and comfort level in providing care for the hospitalized pediatric pulmonology patient including those with diagnoses such as cystic fibrosis, tracheostomy and ventilator dependence, and/or inadequate airway clearance.

Citation:
Khan EK, Liptzin DR, Baker J, Meier M, Baker CD, Lockspeiser TM. Pediatric resident education in pulmonary (PREP): a subspecialty preparatory boot camp curriculum for pediatric residents. *MedEdPORTAL*. 2021;17:11066.
https://doi.org/10.15766/mep_2374-8265.11066

Introduction

Medical errors can occur any time resident physicians transition between rotations, especially to unfamiliar hospital units, a phenomenon coined by Sobolewski et al as "the April Effect."[1] This contrasts with the so-called July Effect in which there are reports of increased medical errors during the first month of the academic calendar, when recent medical school graduates become practicing resident physicians.[2] Orientation to residency has been shown to improve confidence, reduce anxiety, and increase resident contentment.[3-6] There are limited data published regarding the transitions of residents to new and unfamiliar rotations within their residency training.

Many medical schools, residencies, and professional organizations have adopted boot camp models to aid in postgraduate or postresidency transitions.[7-10] Many of these boot camp models are structured as large learning experiences

Copyright © 2021 Khan et al. This is an open-access publication distributed under the terms of the Creative Commons Attribution license.

at a single point in time, such as during the transition into residency. Although helpful in the initial transition, these boot camp experiences lack the benefit of just-in-time (JIT) learning delivered immediately before caring for patients in subspecialized areas of practice. JIT learning is an educational framework which emphasizes providing focused and tailored teaching of topics at the point in time when application of that knowledge is most needed.[11] Studies using JIT training have resulted in positive knowledge, skills, and confidence gains amongst medical professionals.[11-12] Though JIT training appears to be an ideal model for instruction during resident transitions, only one publication highlighted timely training for resident transition to a pediatric subspecialty rotation. A high-yield educational boot camp enhanced readiness and confidence in pediatric residents transitioning to the neonatal intensive care unit at the Children's Hospital of New Orleans.[13] A review of published educational materials in PubMed and *MedEdPORTAL* yielded no citations related to improving resident knowledge in the care of the pediatric pulmonary patients or preparing residents to transition to the pediatric pulmonary unit.

Pediatric pulmonology is a special area of subspecialty pediatrics, with new patient populations that residents may not encounter prior to participating in a pulmonology experience. In our center, the Children's Hospital Colorado (CHCO), patients with cystic fibrosis (CF), noninvasive positive pressure ventilation (NIPPV) requirements, and tracheostomy and ventilator (TV) dependence are admitted almost exclusively to our pediatric pulmonology unit. At CHCO, all pediatric residents are required to complete an inpatient pediatric pulmonary rotation during their second year of training. During this rotation they care for patients with asthma, CF, NIPPV requirements, and TV dependence. This is often the first experience our residents have in caring for patients with CF or TV dependence, prompting the need for enhanced education for residents prior to taking over patient care. Additionally, significantly more acute patient events occur on our pulmonary unit, and over 60% of code blue or rapid response events require transfer to the pediatric intensive care unit, compared to only 37% hospital-wide at CHCO (data not publicly available, taken from secure institutional source). High patient acuity and an unfamiliar patient care environment raises the potential for an increase in medical errors in this vulnerable patient population, prompting the need for formal education so residents can provide safe and quality care to pulmonary inpatients.

We therefore aimed to provide PGY 2 residents rotating through inpatient pulmonary at CHCO with the fundamental knowledge, skills, and attitudes required to provide quality care to a core set of patients on the inpatient pulmonary team through participation in the pediatric resident education in pulmonary (PREP) boot camp. With the goal of improving resident experience and preparedness in caring for complex pulmonary inpatients while utilizing the JIT training framework, we created a monthly preparatory boot camp on the first day of the pediatric residents' inpatient pulmonary rotation.

Methods

Using the six-step approach to curriculum development as described by Kern and Thomas,[14] we created PREP. Details of these six steps, including problem identification, needs assessment, goals and learning objectives, educational strategies, implementation, and evaluation and feedback, are detailed below.

Problem Identification/Needs Assessment

As a problem identification and needs assessment for this curriculum, we extensively reviewed the literature, as well as internal evaluations of the inpatient pediatric pulmonary rotation completed by the residents in the 5 previous years. Residents expressed a desire for timely, structured education in unfamiliar patient populations such as those with CF or TV dependence. Education was previously provided in an on-the-fly manner, without a structured curriculum in place. A high census often translated to missed educational opportunities. During informal focus groups, residents voiced concerns of service obligations getting in the way of formal education on the first day of a rotation, when they are trying to learn about new patients and take on a new provider role. Residents expressed a desire to have time dedicated to didactic sessions prior to rotating into unfamiliar areas of patient care. Unfortunately, the nature of resident schedules and hospital staffing had prevented this from occurring.

Purpose, Goals, and Learning Objectives

The primary goal of PREP was to equip residents with fundamental knowledge and skills to provide quality care to a core set of pediatric pulmonology inpatients. Specific learning objectives for each of the sessions were creating by reviewing the American Board of Pediatrics specifications unique to pediatric pulmonology,[15] reviewing feedback from residents, and discussing with attending physicians from the pulmonary team.

Educational Strategies

Given that the emphasis was on development and enhancement of residents' actual skills and application of knowledge gained during the sessions, the session was designed to be hands-on

Copyright © 2021 Khan et al. This is an open-access publication distributed under the terms of the Creative Commons Attribution license.

and emphasized active learning. No prerequisite knowledge was required of the learners.

Implementation

A schedule for PREP sessions was created prior to the beginning of the 2018-2019 academic year. PREP was held monthly on the first day of the residents' inpatient pediatric pulmonary rotation. We worked with the pediatric chief residents to ensure no resident starting pulmonary would be postcall and therefore unable to attend. Prior to taking over patient care, residents participated in PREP to learn to care for the complex pulmonary patients. Meanwhile, the inpatient service was staffed by pulmonary advanced practice providers, a fellow, and attending(s). This allowed the resident physicians to focus on learning without patient care obligations getting in the way.

PREP was a 5-hour session with multiple high-yield components of interactive lectures and hands-on sessions. Instructors for each session should be knowledgeable in the content being taught and may include pediatric pulmonology attending or fellow physicians, nurses and/or respiratory therapists skilled in the care of TV-dependent patients and/or familiar with various airway clearance and lung expansion devices and their use.

Appendix A contains an example agenda detailing the schedule of events. The individual components, specific learning objectives, and supplies needed for each session are described below.

Orientation to inpatient pulmonology (Appendix B): This 30-minute session included details unique and specific to the CHCO pulmonary unit, including important staff members, rounding and presentation guidelines, and electronic medical record pointers. By the end of this session, learners will be able to: (1) Recognize integral members of the inpatient pulmonology team; and (2) Understand the unique electronic medical record complexities of inpatient pulmonology. Appendix B is a template PowerPoint presentation, which can be adapted to any other hospital system. Implementation required audiovisual equipment (computer, connections, projector).

Introduction to TV lecture (Appendix C): This 45-minute session provided a general overview of TV in the form of an interactive PowerPoint lecture. By the end of this session, learners will be able to: (1) Identify indications for placing a tracheostomy tube; (2) Differentiate various types of tracheostomy tubes, including cuffed and uncuffed; (3) Differentiate various ventilator modes; and (4) Understand common tracheostomy tube complications and interventions. Appendix C is a copy of the presentation used in our session. We recommend adapting the presentation to include specific equipment used in the hospital system providing the education. Implementation required audiovisual equipment (computer, connections, projector) and sample tracheostomy tubes, including cuffed and uncuffed examples.

TV simulation session: A 60-minute hands-on low-fidelity simulation activity by Khan et al[16] was downloaded from *MedEdPORTAL* for implementation. The simulation included three scenarios of acute inpatient events in the TV-dependent patient. Each learner should have the opportunity to participate in the simulation, gaining hands-on experience. Specific learning objectives, personnel and equipment required, and a standardized debriefing template following each scenario was included in the respective instructor guides.

CF JeoPARODY interactive lecture (Appendix D): This 45-minute session was an interactive lecture in the form of a game show activity providing an overview of CF care and acute management of the hospitalized CF patient. By the end of this session, learners will be able to: (1) Recognize the pathogens commonly associated with the pulmonary complications of CF; (2) Recognize the pulmonary and extrapulmonary complications of CF in children of various ages; and (3) Develop a differential of hemoptysis in a patient with CF. Appendix D contained the presentation with various categories to guide discussion. CF JeoPARODY was presented with permission of the template creators of Youth Downloads.[17] Implementation required audiovisual equipment (computer, connections, projector), whiteboards with dry-erase markers (alternative: paper with writing utensils) for score keeping and final round answer documentation, buzzers, and candy/prizes (optional).

Introduction to airway clearance and lung expansion devices interactive lecture (Appendix E): This 30-minute session was an interactive PowerPoint lecture providing an overview of airway clearance and lung expansion devices. By the end of this session, learners will be able to: (1) Differentiate airway clearance devices from lung expansion techniques; and (2) Identify patient populations that would benefit from various airway clearance and/or lung expansion devices. Appendix E contained lecture slides to guide the presentation. Implementation required audiovisual equipment (computer, connections, projector).

Hands-on airway clearance and lung expansion devices session (Appendices F-M): This 75-minute session was a hands-on interactive session allowing learners to try various airway clearance and lung expansion devices. By the end of this session, learners will be able to: (1) Describe each device and how it

Copyright © 2021 Khan et al. This is an open-access publication distributed under the terms of the Creative Commons Attribution license.

works; (2) Identify which patient population best supports the need for each device; (3) Discuss when to transition patient from one airway clearance therapy to another; (4) Discuss how to evaluate the effectiveness of various therapies; and (5) Discuss and implement appropriate treatment settings and modifications in various devices to support adequate airway clearance.

Appendices F-M included facilitator guides for common devices and modalities including manual chest physiotherapy, incentive spirometry, positive expiratory pressure device (PEP), intermittent positive airway pressure (PAP) therapy, oscillatory positive expiratory pressure devices (OPEP), insufflator/exsufflator device, high frequency chest wall oscillation device (HFCWO), and intrapulmonary percussive ventilation device (IPV). Implementation required the following equipment:

- Bacteria filter for each learner.
- Mouthpiece for each learner (optional).
- Percussive cup.
- Incentive spirometer.
- PEP therapy device (TheraPEP).
- Intermittent PAP therapy device (EzPAP).
- OPEP therapy devices (Acapella and Aerobika).
- Insufflator-exsufflator device (Cough Assist) with machine tubing.
- HFCWO device (such as Vest) with machine tubing and vest or wrap.
- IPV device with tubing.
- Oxygen delivery with tubing.

Evaluation and Feedback

Residents completed a paper survey just prior to PREP and immediately after PREP that used several 5-point Likert scales to assess their perceived confidence and preparedness in starting the rotation and taking overnight call (Appendix N). Open-ended questions provided qualitative evaluation of the curriculum. Debriefing sessions were held with residents throughout their month-long rotation to assess utility of the PREP curriculum. At the end of their rotation, residents completed an online survey evaluating the overall effectiveness of PREP (Appendix O). Formal feedback was sought from pulmonary faculty via online survey (Appendix P). Quantitative analysis of survey responses included calculations of frequency. The preparedness ratings before and after PREP boot camp were compared using Student's *t* test.

Two formal resident focus groups were held during the first year of PREP to provide a basis for qualitative evaluation of patient-centered outcomes and results (Appendix Q). Each focus group was audio recorded and transcribed with resident identifiers removed. Two researchers independently reviewed each transcript and coded the data to identify common themes. The researchers then developed a coding schema based on discussion until consensus was achieved. The focus group transcripts and all narrative survey responses were then coded with the final coding schema. Discussion amongst the researchers led to the final emergent themes. This is an optional evaluation method and would optimally be held by an individual with experience in qualitative data gathering and focus group facilitation.

Results

PREP was successfully implemented starting in July 2018. Sessions were held monthly on the first day of each resident rotation with two to three residents participating in each session. Thirty-five residents participated during the first year. All residents (35 of 35) completed paper surveys before and after PREP. Resident perceived preparedness in taking overnight call increased significantly following PREP, with a mean preparedness score on a 5-point Likert scale (1 = *not at all prepared*, 5 = *extremely prepared*) of 2.7 on prePREP evaluations increasing to 3.8 on postPREP evaluations ($p \leq .0001$). On prePREP evaluations, a majority rated themselves as *somewhat prepared* (79%) or *not so prepared* (34%) with only one individual (3%) claiming to be *very prepared*. On postPREP evaluations, there was a positive shift in preparedness ratings, with a majority now rating as *very prepared* (79%) or *somewhat prepared* (34%), 7% *extremely prepared*, and none *not so prepared* or *not at all prepared* (Figure). All residents (35 of 35) rated PREP as *extremely helpful* (32 of 35, 91%) or *very helpful* (3 of 35, 9%), the highest possible ratings.

Average ratings of the importance of each component of PREP for overall learning demonstrated the utility of individual curriculum components (Table 1). Residents most enjoyed the TV simulation ($M = 4.9$) and the hands-on airway clearance and lung expansion devices session ($M = 4.8$). Initially, residents reported the worst areas of PREP to be the asthma lecture ($M = 3.7$) resulting in curriculum revisions to reduce lecture time and increase hands-on time, after which a majority of residents reported nothing to be the worst part of PREP on postPREP evaluations.

Of residents, 83% (29 of 35) responded to the end-of-rotation online survey. On this survey, residents continued to report that PREP was helpful in enhancing their education during the rotation, with 100% still reporting PREP as *extremely helpful* (25 of 29, 86%) or *very helpful* (4 of 29, 14%). All qualitative

Copyright © 2021 Khan et al. This is an open-access publication distributed under the terms of the Creative Commons Attribution license.

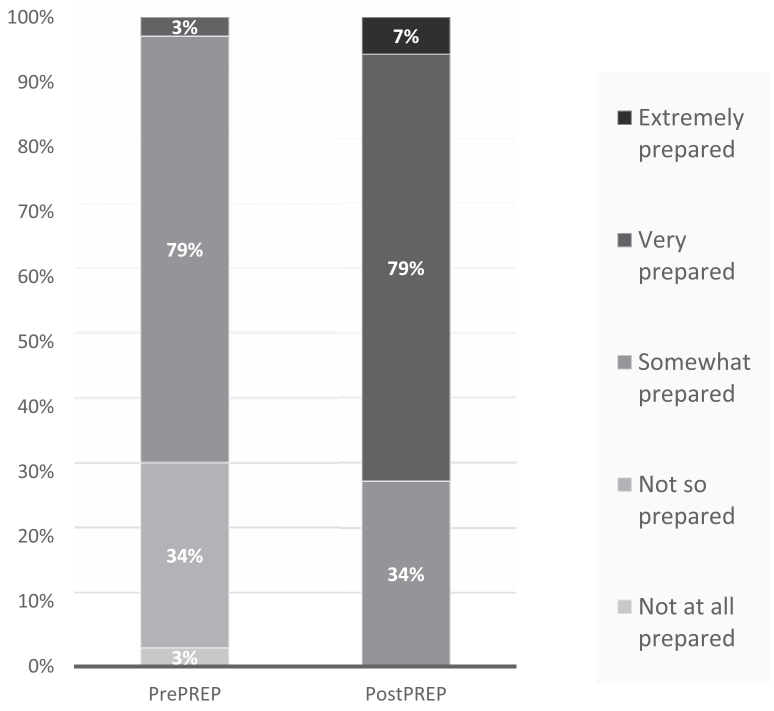

Figure. Preparedness ratings of learners at pre- and postPREP boot camp on a 5-point Likert scale (1 = *not at all prepared*, 5 = *extremely prepared*). The 1.1-point increase in average score from pre- to postPREP boot camp was significant ($p \leq .0001$). Abbreviation: PREP, pediatric resident education in pulmonary.

data from open-ended survey questions revealed the positive effects of PREP. In addition, eight residents participated in two voluntary focus groups to evaluate the program and the focus group data was added to the qualitative data from the surveys. Thematic saturation was achieved after the second focus group, therefore no additional focus groups were completed. In addition to numerous general positive comments about PREP noted in both the qualitative comments from the end of rotation surveys and the focus groups, six main themes emerged: (1) PREP decreased resident anxiety; (2) PREP helped residents learn about contingency planning for emergencies; (3) certain aspects of the content were deemed most helpful; (4) residents valued the opportunity to learn and practice key skills; (5) residents applied what they learned on their pulmonary rotation and beyond; (6) residents appreciated dedicated time for teaching. Table 2 summarized these themes with a detailed definition of each theme along with example quotes from the focus group sessions and/or end-of-rotation surveys.

Table 1. Average Resident Ratings of the Importance of Each PREP Boot Camp Session

PREP Session	M^a
Orientation to inpatient pulmonology (n = 35).	4.1
Introduction to TV lecture (n = 35).	4.7
TV simulation session (n = 35).	4.9
CF JeoPARODY interactive lecture (n = 35).	4.5
Introduction to airway clearance and lung expansion devices interactive lecture (n = 35).	4.7
Hands-on airway clearance and lung expansion devices session (n = 35).	4.8
Introduction to asthma lecture (n = 15).[b]	3.7
Pulmonary function testing hands-on session (n = 15).[b]	4.1
Introduction to bronchoscopies hands-on session (n = 15).[b]	3.8

Abbreviation: CF, cystic fibrosis; PREP, pediatric resident education in pulmonary; TV, tracheostomy and ventilator.
[a]Rated on a 5-point Likert scale (1 = *not at all important*, 5 = *extremely important*).
[b]These sessions were excluded from later PREP boot camp iterations based on initial resident feedback.

Of clinical faculty, 86% (18 of 21) responded to the online evaluation of PREP. Most (15 of 18, 83%) faculty members spent over 2 weeks on clinical service working with residents during the first year of PREP with 7 out of 18 (39%) having spent more than 4 weeks on clinical service. Three respondents had not participated in clinical service since initiation of PREP and were excluded from analysis. All (15 of 15) found PREP to be *extremely valuable* or *very valuable* to the residents' educational experience. Qualitative analysis of faculty responses was uniformly positive. When asked what skills the residents have gained as a result of PREP, faculty feedback included similar themes to the residents' report:

- Overall patient care: All faculty who provided a narrative response reported residents increased ability to care for

Copyright © 2021 Khan et al. This is an open-access publication distributed under the terms of the Creative Commons Attribution license.

Table 2. Summary of Resident Responses to PostPREP Surveys and Focus Group Sessions

Theme	Code	Code Definition	Example Quote
PREP decreased resident anxiety.	Anxiety	Residents have anxiety taking call in an unfamiliar setting, such as the pulmonary unit, and PREP helped to decrease this anxiety and/or improve confidence.	"It was really nice to have the boot camp ahead of that first call night because, while it was still a very nerve-wracking experience, I felt like I had some tools in my pocket for some common like high acuity scenarios."
	Alone	Unique to the pulmonary inpatient rotation, residents feel alone, without in-house back-up while on call.	"Because it's also like, you know, our first time truly by ourselves without another provider on our service or a fellow in-house. So it's scary."
PREP helped residents learn about contingency planning for emergencies.	Contingency planning	Residents found PREP helpful in developing contingency plans, especially for overnight emergencies.	"I was on call the first day and within my first hour I was called to evaluate a patient who was apneic, bradycardic, desatting to 50%, and mottled. I called a code, switched out his trach, which was plugged, and bagged him up, which was the exact practice example we went over in boot camp. I felt confident with the initial management; whereas I would have been very uncomfortable without having the boot camp ...I felt prepared to provide the appropriate response."
Certain aspects of the content were deemed most helpful.	CF	Residents reported increased knowledge of treatment and emergencies in the CF population.	"We discussed how to manage hemoptysis; I had a patient with CF who developed mild to moderate hemoptysis overnight and I knew what to do."
	New and acute	Residents desired a focus on new, acute, and emergent scenarios rather than familiar or known topics in PREP.	"Because the thing you want out of boot camp is, 'How am I going to survive my first call night?"'
Residents valued the opportunity to learn and practice key skills.	Tracheostomy care and troubleshooting	Residents reported increased knowledge of tracheostomy tubes and/or the ability to troubleshoot tracheostomy tube complications.	"I learned how to change a trach, how to troubleshoot when a child [with tracheostomy and ventilator dependence] is having difficulty. High pressure alarms? Try suctioning. Low pressure alarms? Make sure everything is connected. Tachypnea/hypoxemia/struggling [to breathe]? Change the trach."
	Airway clearance use	Residents valued learning how to use certain airway clearance therapies.	"Learning about the various forms of airway clearance was helpful in understanding how it would help our patients."
Residents applied what they learned on their pulmonary rotation and beyond.	Applying skills	Residents took what they learned in PREP and used it while caring for patients during their pulmonary rotation, most often being examples from overnight call.	"My first thing I recall is running over there and knowing who the patient was and thinking I had a sense of what might be going on. Like, we are going to do a trach change. If we haven't already, like as soon as I get in the room. So, you know, if it had been my first call night and I hadn't had the pulmonary boot camp, I don't know what my first thought would have been running into the room, so I am appreciative of that. And, you know, we did a trach change and he was stable."
	Beyond pulmonary	Residents used skills and knowledge gained during PREP in other areas and rotations of residency.	"I had a couple of patients in the ICU who are chronic trach patients, adults with cystic fibrosis and I really felt that what I learned from my pulmonary rotation really translated over, made me more confident taking care of those patients. Like, 'Oh yeah, the airway clearance! Here are all the options!' So that made me think like that carried over and benefitted me. And that was like 3 or 4 months out in my pulmonary rotations. So yeah, some of it stuck."
Residents appreciated dedicated time for teaching.	Time	A key component of the success of PREP was the protected time allowed for learning before taking on patient care responsibilities.	"I definitely think compared to the other rotations, it was for sure the smoothest transition day. It helps to have that learning, not to have to round that first morning. You have that afternoon to sort of meet your patients, talk to the families, prepare your thoughts for the night or the next day."
	Regularly scheduled teaching	Though PREP provided great foundational knowledge, residents desired regularly scheduled teaching throughout their pulmonary rotation.	"I wondered too, if sometimes it feels like the boot camp becomes a license to not do teaching throughout the month."

Abbreviations: CF, cystic fibrosis, PREP, pediatric resident education in pulmonary.

pulmonary patients. One stated that PREP "helps to better prepare them for starting their pulmonary month. I think this directly translates to improved patient safety and patient care."

- Tracheostomy care and troubleshooting: Faculty also noted residents increased ability to care for the tracheostomy-dependent patient, often commenting on an increased foundation of knowledge surrounding tracheostomy tubes and complications.
- CF care: Many commented on the residents' increased knowledge of CF patient care, with one stating, "Their knowledge of CF care and airway clearance seems improved compared to prior residents."

Copyright © 2021 Khan et al. This is an open-access publication distributed under the terms of the Creative Commons Attribution license.

- Emergency planning: Faculty responded that residents were more prepared to deal with emergent scenarios. A faculty answered, “Practical skills around trach emergencies have prepared residents to take the lead in emergent scenarios.”
- Increased confidence: Many respondents noticed increased resident confidence and comfort when starting their pulmonary rotation.

Discussion

Our monthly PREP boot camp on the first day of residents' inpatient pulmonary rotation has improved resident experience, preparedness, and ability to care for complex pulmonary patients. Our curriculum was the first of its kind, addressing the gap in training education for residents transitioning to pediatric pulmonology. Protected time for residents, stakeholder buy-in, and active learning strategies were key to success of PREP.

PREP was successful due to many factors which emerged as common themes in our data analysis, one being the unique nature and characteristics of our inpatient pediatric pulmonary experience. This was the first rotation where our residents were on call in the hospital by themselves without backup such as a senior resident or fellow in-house. It was an anxiety-provoking rotation due to the acuity of patients on the pediatric pulmonary floor and the lack of exposure to such patient populations prior to this experience. The content of PREP was highly tailored to their specific experience, providing them with prime patient-specific examples that later emerged during their inpatient experience, with contingency plans for when they were on call alone, and with specific skills that were used over and over again, allowing them to retain such skills for later use and promote increased confidence with actual patient care experiences. The timing of PREP allowed for JIT learning prior to beginning the rotation, as well as protected time to focus solely on learning prior to beginning patient care experience.

Our center successfully implemented this blocked boot camp model, which allowed residents to fully participate in the half-day educational session without patient care obligations interrupting learning opportunities on the first day of the new rotation. We chose to provide the blocked 5-hour protected session based on initial feedback during the problem identification portion of our curriculum development. Another rotation at our center employed an adapted orientation model with daily sessions spaced out over the first week of the rotation. Resident feedback of this model was highly negative, reporting many issues of interfering obligations with rounding and taking on patient care, along with concerns for missing key sessions due to postcall absences. Though less ideal, other centers could thoughtfully consider a more spaced out approach to PREP.

The curriculum was adjusted in response to feedback in the first 6 months. Residents appreciated active learning strategies, therefore we aimed to increase hands-on time and interactive sessions during PREP and reduced lecture time. Residents also desired more emphasis on overnight emergencies during PREP given the pressing concern of their first call night, with long-term pulmonary educational topics covered later. We created a longitudinal learning calendar for the rest of the residents' inpatient month based on this feedback moving topics such as outpatient asthma management to later in their pulmonary rotation.

Our evaluation methods provided evidence of the impact of the curriculum. Reactions from residents and faculty were uniformly positive. Residents liked PREP and found it useful. PREP improved resident perceived preparedness in taking call and caring for pulmonary inpatients. Self-reflection following their pulmonary rotation noted that PREP was highly useful in their educational experience. Residents were able to express how PREP enhanced their ability to provide care to the complex pulmonary inpatients on postrotation evaluations 4-8 weeks following participation in PREP. Specific patient care examples included items such as intervening on tracheostomy plugging or evaluating hemoptysis in a patient with CF, scenarios that were specifically discussed during PREP. Formal resident focus group sessions held 1-10 months following resident completion of inpatient pulmonary provided additional evidence that residents were able to take knowledge and skills learned specifically during PREP and apply the new knowledge and skills to multiple real-life patient care experiences, both during inpatient pulmonary and to other patient care experiences such as the emergency department, ICU, and outpatient care. Faculty members who regularly staffed the pulmonary unit and directly observed the residents' during actual patient care experiences similarly reported gains in resident confidence and ability to care for pulmonary-specific patients.

In addition to the formal evaluation measures, we received a significant amount of unsolicited positive feedback from team members outside of the pulmonary division. Due to the overwhelmingly positive response from learners and faculty, other clinical subspecialty pediatrics rotations in our center, including the rotation that had previously used daily sessions spread over the first week, have started similar blocked boot camp-style curricula to enhance learner education and

Copyright © 2021 Khan et al. This is an open-access publication distributed under the terms of the Creative Commons Attribution license.

preparation when transitioning to their rotations. Future directions will include the development of a framework which others may use to develop similar boot camp curricula for various subspecialty areas.

Adequate equipment, including access to audiovisual equipment, various airway clearance devices, and simulation equipment may present a barrier to successful implementation of similar programs. We recommend checking all audiovisual equipment prior to holding the first educational session, since functioning equipment is crucial to success. In times of high patient demand, we occasionally lacked availability of specific airway clearance or lung expansion devices. Residents who were not able to physically try an insufflator/exsufflator device, but still learned of the concept behind appropriate use, did not report decreased importance scores on postsession evaluations. Simulation equipment is expensive and can be difficult to obtain. We combatted this by using low-fidelity simulation equipment, which could easily be implemented in any center.

Other limitations and challenges to sustainability included continued support for inpatient care as well as protected time for PREP instructors. We found this to be a barrier in the early stages of PREP initiation. However, once the utility of PREP proved to be significant in enhancing resident education and ability to care for patients, members of the pulmonary care team were more willing to donate time to support the sustainability of PREP. Having multiple members able to teach various components of PREP relieved some burden, rather than requiring a specific individual to run the entire 5-hour session each month. An additional limitation of this work was that the evaluation approach did not directly measure behavioral change or knowledge gains. However, the comments from both residents and faculty describing specific patient-centered instances of applied learning gained from PREP indirectly demonstrated positive knowledge and behavioral gains. We intentionally did not assess knowledge gains with a multiple-choice test, as our primary outcome of interest of PREP was the impact on direct patient care experiences.

In conclusion, our PREP boot camp curriculum improved learner knowledge, skills, and attitudes in perceived ability to care for complex pulmonology inpatients. We plan to evaluate patient outcome data as our hypothesis remains that patient care has improved following this increased education of our resident providers. We hope that anyone in pediatric pulmonology as well as other areas of subspecialty practice may be able to adopt a similar model to enhance learner education and patient care experience.

Appendices

A. Example Agenda.docx
B. Orientation Template.pptx
C. Introduction to Tracheostomies and Ventilators.pptx
D. Cystic Fibrosis JeoPARODY.pptx
E. Introduction to Airway Clearance and Lung Expansion.pptx
F. Instructor Guide CPT.docx
G. Instructor Guide IS.docx
H. Instructor Guide PEP.docx
I. Instructor Guide PAP.docx
J. Instructor Guide OPEP.docx
K. Instructor Guide Insufflator Exsufflator.docx
L. Instructor Guide HFCWO.docx
M. Instructor Guide IPV.docx
N. PREP Day of Evaluation.docx
O. PREP End of Rotation Evaluation.docx
P. PREP Faculty Feedback Survey.docx
Q. PREP Focus Group Guide.docx

All appendices are peer reviewed as integral parts of the Original Publication.

Erin K. Khan, MD: Fellow, Department of Pediatrics, Section of Pulmonology and Sleep Medicine, University of Colorado School of Medicine; ORCID: https://orcid.org/0000-0001-6629-6164

Deborah R. Liptzin, MD: Assistant Professor, Department of Pediatrics, Section of Pulmonology and Sleep Medicine, University of Colorado School of Medicine

Joyce Baker, MBA, RRT-NPS: Asthma Clinical Program Coordinator, Department of Pediatrics, Section of Pulmonology and Sleep Medicine, University of Colorado School of Medicine

Maxene Meier, MS: Research Instructor, Department of Pediatrics, University of Colorado School of Medicine

Christopher D. Baker, MD: Associate Professor, Department of Pediatrics, Section of Pulmonology and Sleep Medicine, University of Colorado School of Medicine; Director of Ventilator Care Program, University of Colorado School of Medicine

Tai M. Lockspeiser, MD, MHPE: Associate Professor, Department of Pediatrics, University of Colorado School of Medicine; Assistant Dean of Medical Education, University of Colorado School of Medicine;

Disclosures

None to report.

Copyright © 2021 Khan et al. This is an open-access publication distributed under the terms of the Creative Commons Attribution license.

Funding/Support
None to report.

Prior Presentations
Khan EK, Meier M, Baker CB, Liptzin DR, Lockspeiser TM. Pulmonary resident boot camp: a novel subspecialty preparatory curriculum for pediatric residents. Poster presented at: American Thoracic Society International Conference; May 2019; Dallas, TX.

Ethical Approval
The Colorado Multiple Institutional Review Board approved this study.

References

1. Sobolewski B, Kerrey BT, Geis GL, Bria CL, Mittiga MR, Gonzalez del Rey JA. The April effect: a multimedia orientation approach to improve rotation transitions during pediatric residency. *Acad Pediatr*. 2016;16(3):220-223. https://doi.org/10.1016/j.acap.2016.02.005
2. Shah AY, Abreo A, Akar-Ghibril N, Cady RF, Shah RK. Is the "July effect" real? Pediatric trainee reported medical errors and adverse events. *Pediatr Qual Saf*. 2017;2(2):e018. https://doi.org/10.1097/pq9.0000000000000018
3. Antonoff MB, Swanson JA, Acton RD, et al. Improving surgery intern confidence through the implementation of expanded orientation sessions. *Surgery*. 2010;148(2):181-186. https://doi.org/10.1016/j.surg.2010.04.006
4. Hiraoka M, Kamikawa G, McCartin R, Kaneshiro B. A pilot structured resident orientation curriculum improves the confidence of incoming first-year obstetrics and gynecology residents. *Hawaii J Med Public Health*. 2013;72(11):387-390.
5. Andolsek KM, Murphy G, Pinheiro S, Petrusa E, Tuck T, Weinerth J. Efficacy and efficiency of webcast orientations versus live resident orientations: results of a 2-year survey. *J Grad Med Educ*. 2010;2(1):136-140. https://doi.org/10.4300/JGME-D-09-00105.1
6. Klingenberg C, Skadberg B. Orientation programme for paediatric residents. *Tidsskr Nor Laegeforen*. 2007;127(16):2105-2108.
7. Blackmore C, Austin J, Lopushinsky SR, Donnon T. Effects of postgraduate medical education "boot camps" on clinical skills, knowledge, and confidence: a meta-analysis. *J Grad Med Educ*. 2014;6(4):643-652. https://doi.org/10.4300/JGME-D-13-00373.1
8. Malekzadah S, Malloy KM, Chu EE, Tompkins J, Battista A, Deutsch ES. ORL emergencies boot camp: using simulation to onboard residents. *Laryngocope*. 2011;121(10):2114-2121. https://doi.org/10.1002/lary.22146
9. Ataya R, Dasgupta R, Blanda R, Moftakhar Y, Hughes PG, Ahmed R. Emergency medicine residency boot camp curriculum: a pilot study. *West J Emerg Med*. 2015;16(2):356-361. https://doi.org/10.5811/westjem.2015.1.23931
10. Kelly M, Posa M. Transition to pediatric practice: a residency elective experience to prepare senior pediatric residents for general pediatric primary care. *MedEdPORTAL*. 2016;12:10506. https://doi.org/10.15766/mep_2374-8265.10506
11. Cheng YT, Liu DR, Wang VJ. Teaching splinting techniques using a just-in-time training instructional video. *Pediatr Emerg Care*. 2017;33(3):166-170. https://doi.org/10.1097/PEC.0000000000000390
12. De Gagne JC, Park HK, Hall K, Woodward A, Yamane S, Kim SS. Microlearning in health professions education: scoping review. *JMIR Med Educ*. 2019;5(2):e13997. https://doi.org/10.2196/13997
13. Surcouf JW, Mumphrey CG, Barkemeyer BM, et al. Neonatal intensive care unit boot camp: a preparatory curriculum for pediatric residents. *MedEdPORTAL* 2018;14:10720. https://doi.org/10.15766/mep_2374-8265.10720
14. Kern DE, Thomas PA, Hughes MT, eds. *Curriculum Development for Medical Education: A Six-Step Approach*. Johns Hopkins University Press; 1998.
15. General Pediatrics Content Outline: In-Training, Certification, and Maintenance of Certification Exams. The American Board of Pediatrics. Accessed May 2018. https://www.abp.org/sites/abp/files/gp_contentoutline_2017.pdf
16. Khan EK, Lockspeiser TM, et al. "When in doubt, change it out": A case-based simulation for pediatric residents caring for hospitalized tracheostomy dependent children. *MedEdPORTAL*. 2020;16:10994. https://doi.org/10.15766/mep_2374-8265.10994
17. Youth Downloads. Accessed November 10, 2020. https://www.youthdownloads.com

Received: January 21, 2020
Accepted: September 20, 2020
Published: January 7, 2021

Copyright © 2021 Khan et al. This is an open-access publication distributed under the terms of the Creative Commons Attribution license.
