# Supplementary material for: Pediatric Resident Education in Pulmonary (PREP): A Subspecialty Preparatory Boot Camp Curriculum for Pediatric Residents
Source: MedEdPORTAL. 2021 Jan 7;17:11066. doi: 10.15766/mep_2374-8265.11066 (PMC7809931; doi:10.15766/mep_2374-8265.11066)
Supplement: Supplementary file 1 — Example Agenda.docxOrientation Template.pptxIntroduction to Tracheostomies and Ventilators.pptxCystic Fibrosis JeoPARODY.pptxIntroduction to Airway Clearance and Lung Expansion.pptxInstructor Guide CPT.docxInstructor Guide IS.docxInstructor Guide PEP.docxInstructor Guide PAP.docxInstructor Guide OPEP.docxInstructor Guide Insufflator Exsufflator.docxInstructor Guide HFCWO.docxInstructor Guide IPV.docxPREP Day of Evaluation.docxPREP End of Rotation Evaluation.docxPREP Faculty Feedback Survey.docxPREP Focus Group Guide.docx [file mep_2374-8265.11066-s001.zip › A. Example Agenda.docx]

**Welcome to Pediatric Resident Education in Pulmonary (PREP) Boot Camp!**

**Agenda**

| Time | Event | Location |
| --- | --- | --- |
| 15 minutes | Welcome and Introduction to PREP Boot Camp | Classroom |
| 20-30 minutes | Inpatient Pulmonary Orientation | Classroom |
| 45 minutes | Introduction to Tracheostomies and Ventilator Modes - Lecture | Classroom |
| 60-75 minutes | T/V Simulation Session | Classroom vs  Patient Room vs Simulation Center |
| 45-60 minutes | CF Jeopardy | Classroom |
| 30 minutes | Airway Clearance Lecture | Classroom |
| 60-75 minutes | Hands On Session: Airway Clearance | Classroom vs  Patient Room |
