# Supplementary material for: Pediatric Resident Education in Pulmonary (PREP): A Subspecialty Preparatory Boot Camp Curriculum for Pediatric Residents
Source: MedEdPORTAL. 2021 Jan 7;17:11066. doi: 10.15766/mep_2374-8265.11066 (PMC7809931; doi:10.15766/mep_2374-8265.11066)
Supplement: Supplementary file 1 — Example Agenda.docxOrientation Template.pptxIntroduction to Tracheostomies and Ventilators.pptxCystic Fibrosis JeoPARODY.pptxIntroduction to Airway Clearance and Lung Expansion.pptxInstructor Guide CPT.docxInstructor Guide IS.docxInstructor Guide PEP.docxInstructor Guide PAP.docxInstructor Guide OPEP.docxInstructor Guide Insufflator Exsufflator.docxInstructor Guide HFCWO.docxInstructor Guide IPV.docxPREP Day of Evaluation.docxPREP End of Rotation Evaluation.docxPREP Faculty Feedback Survey.docxPREP Focus Group Guide.docx [file mep_2374-8265.11066-s001.zip › B. Orientation Template.pptx]

## Slide 1
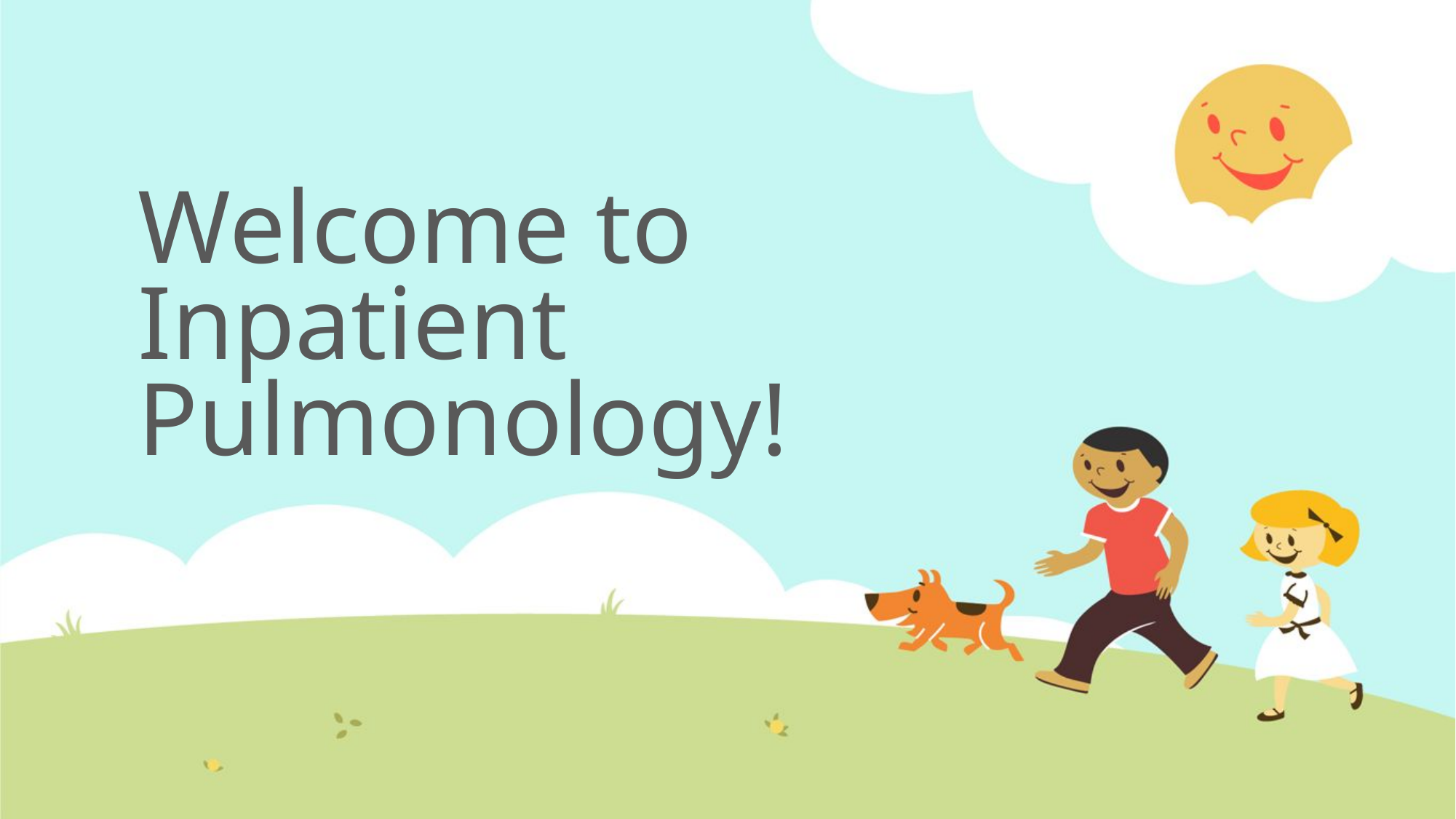

# Welcome to Inpatient Pulmonology!

## Slide 2
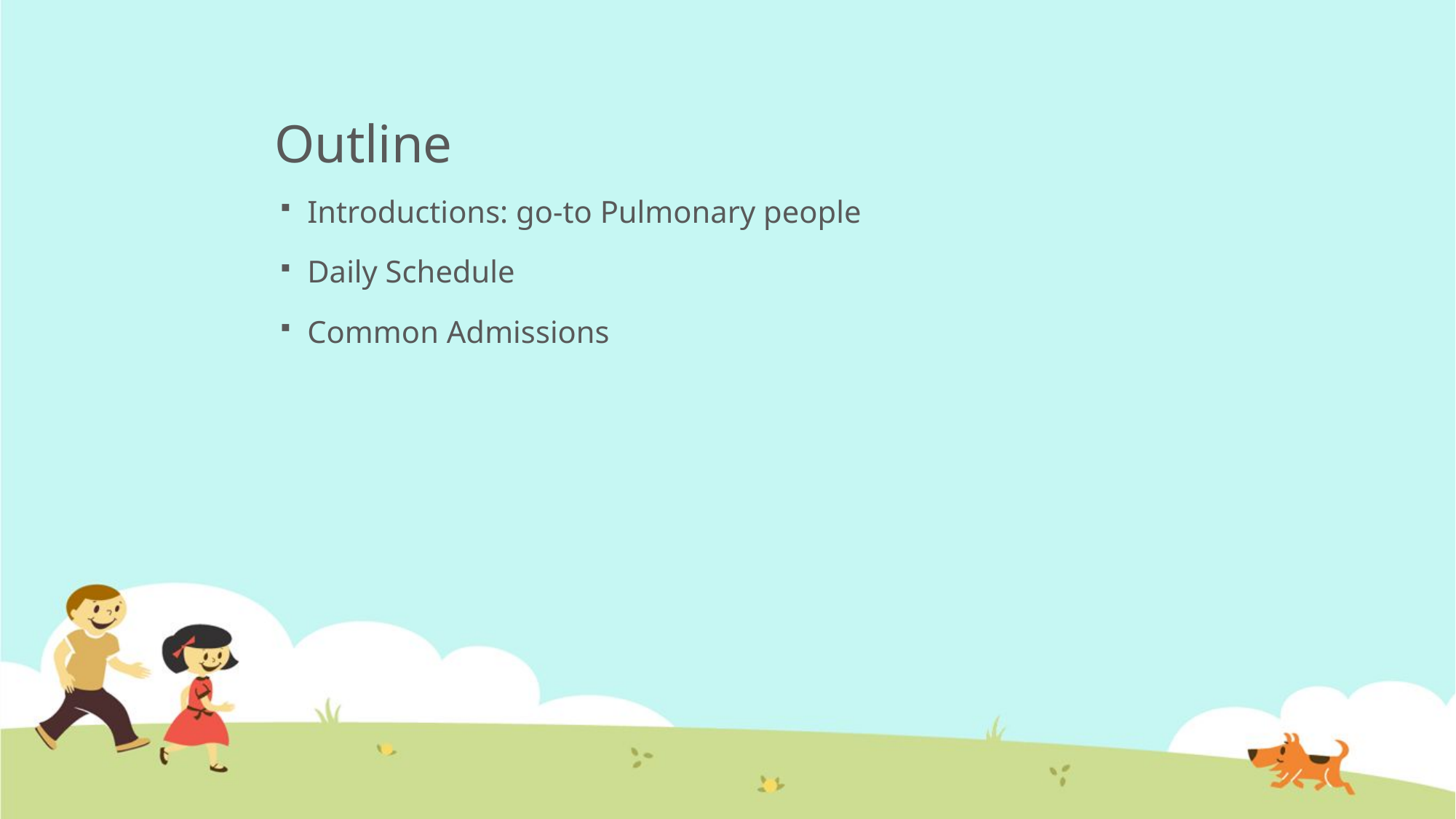

# Outline
Introductions: go-to Pulmonary people
Daily Schedule
Common Admissions

## Slide 3
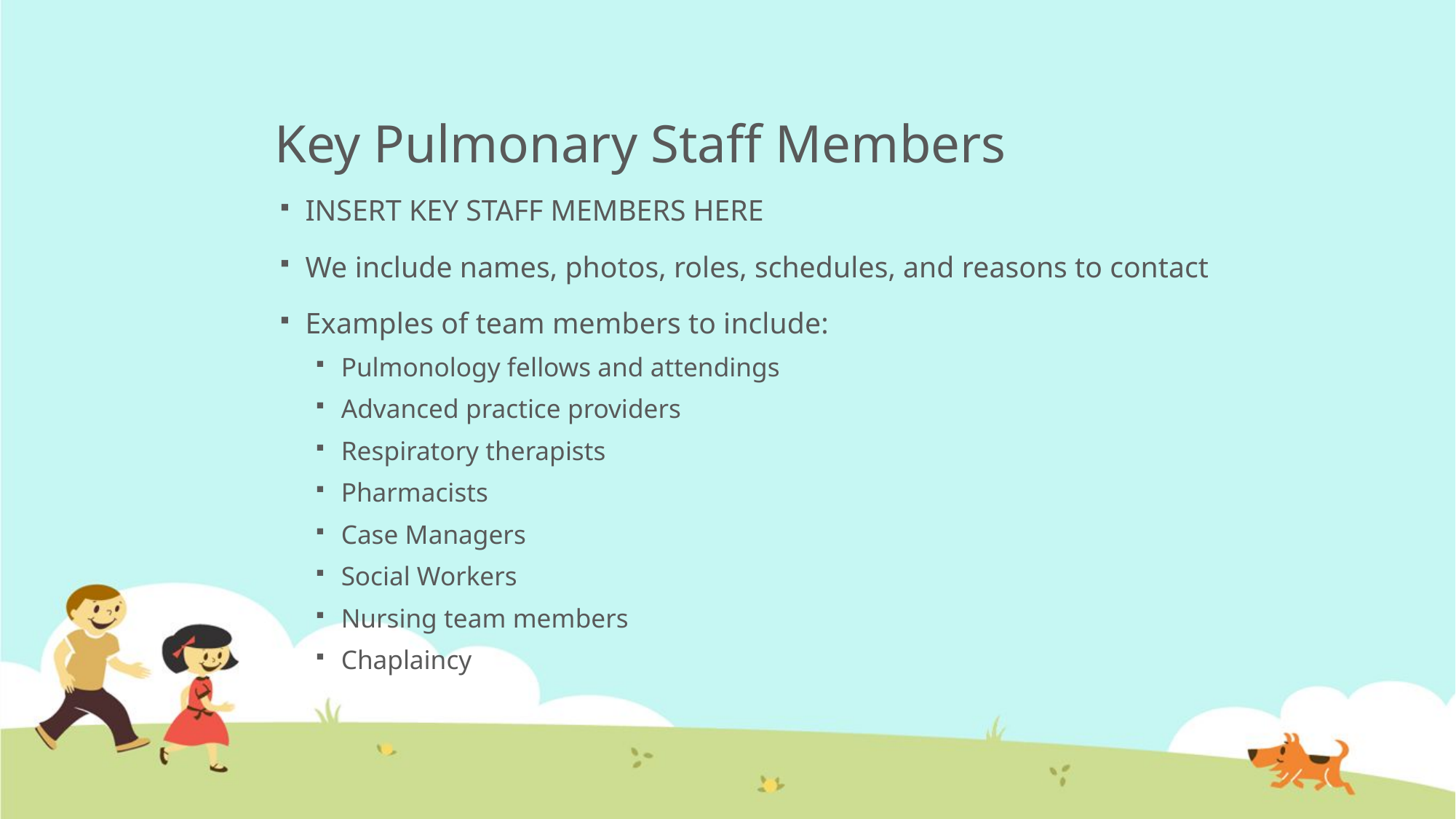

# Key Pulmonary Staff Members
INSERT KEY STAFF MEMBERS HERE
We include names, photos, roles, schedules, and reasons to contact
Examples of team members to include:
Pulmonology fellows and attendings
Advanced practice providers
Respiratory therapists
Pharmacists
Case Managers
Social Workers
Nursing team members
Chaplaincy

## Slide 4
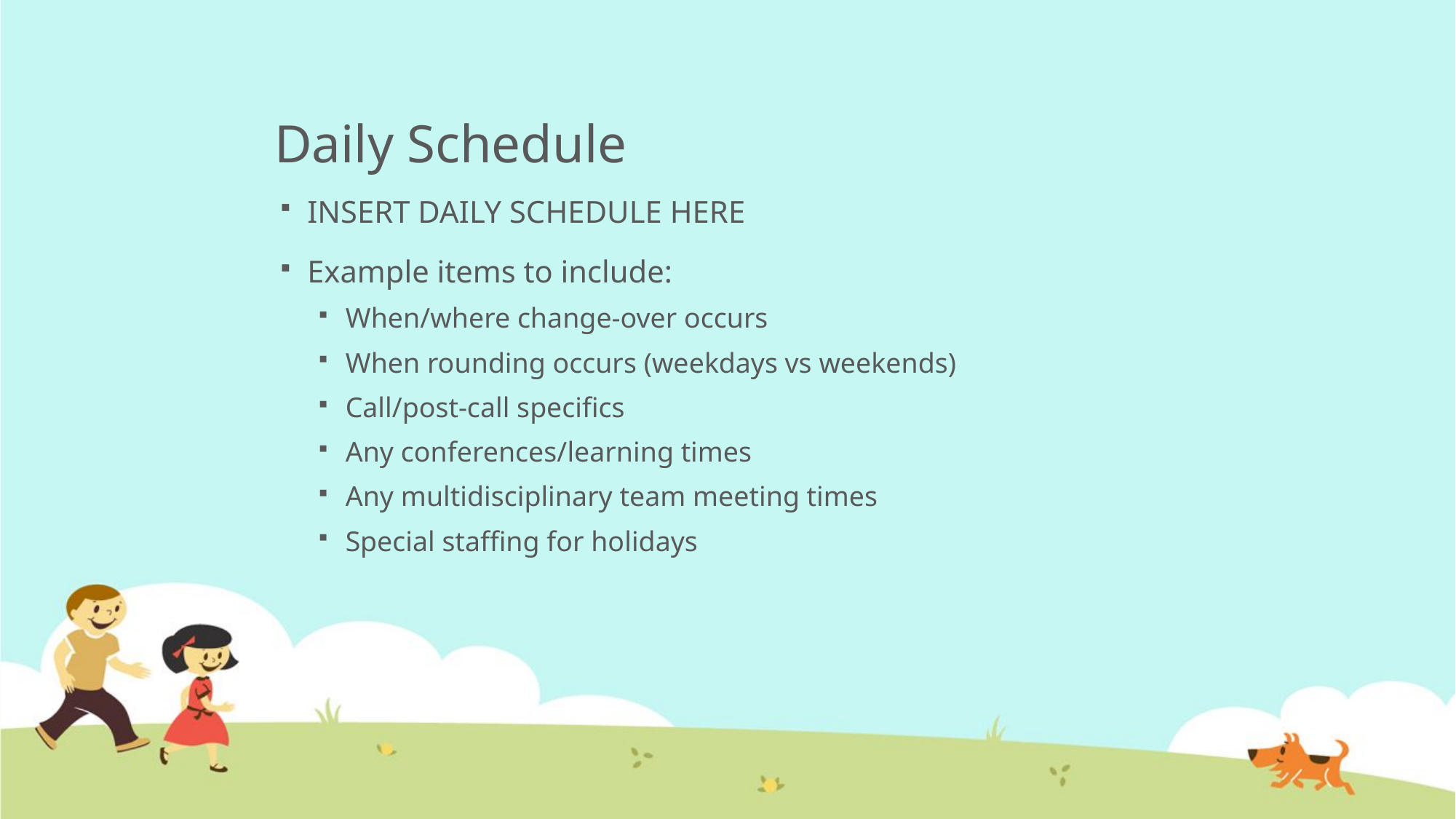

# Daily Schedule
INSERT DAILY SCHEDULE HERE
Example items to include:
When/where change-over occurs
When rounding occurs (weekdays vs weekends)
Call/post-call specifics
Any conferences/learning times
Any multidisciplinary team meeting times
Special staffing for holidays

## Slide 5
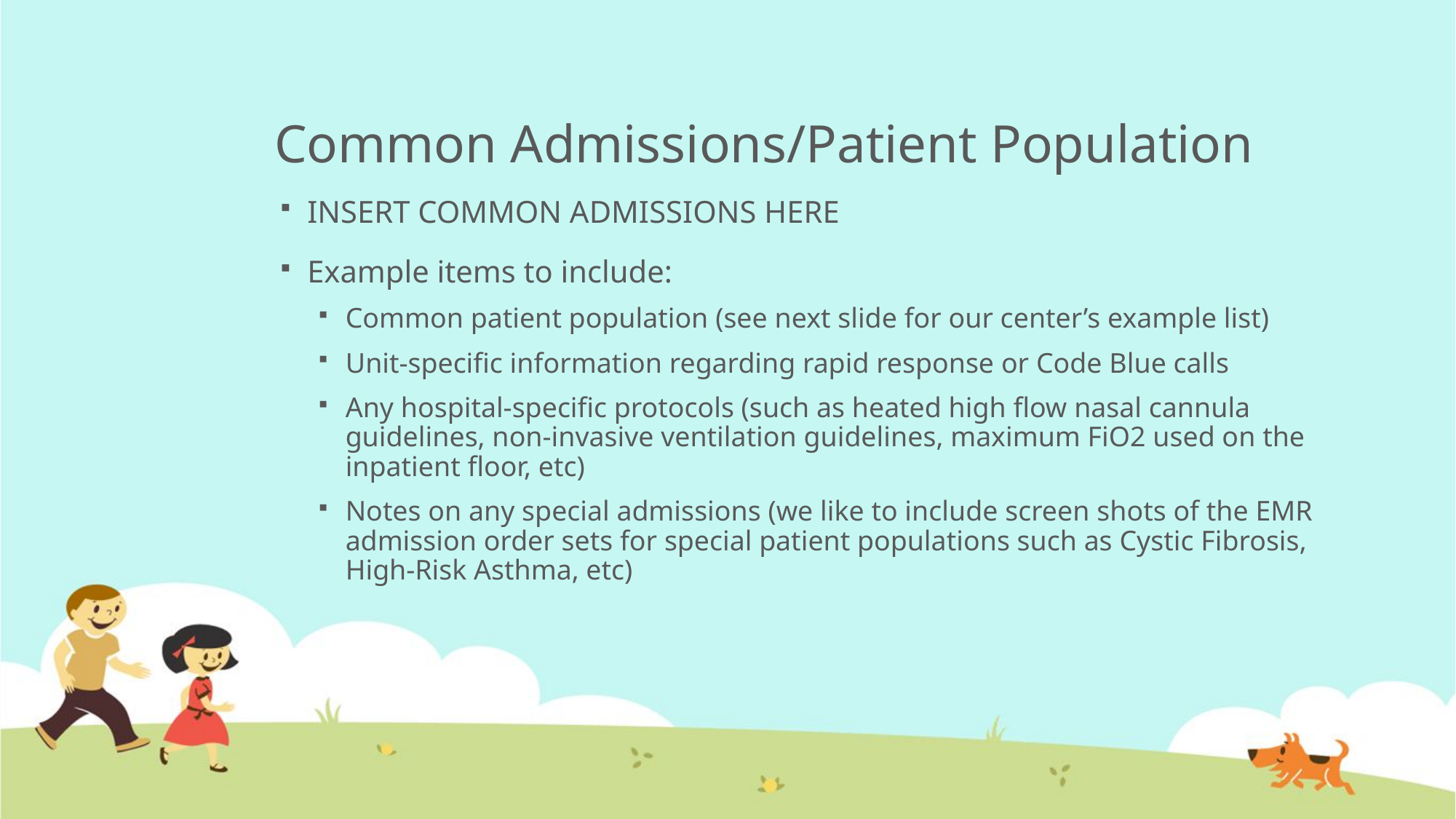

# Common Admissions/Patient Population
INSERT COMMON ADMISSIONS HERE
Example items to include:
Common patient population (see next slide for our center’s example list)
Unit-specific information regarding rapid response or Code Blue calls
Any hospital-specific protocols (such as heated high flow nasal cannula guidelines, non-invasive ventilation guidelines, maximum FiO2 used on the inpatient floor, etc)
Notes on any special admissions (we like to include screen shots of the EMR admission order sets for special patient populations such as Cystic Fibrosis, High-Risk Asthma, etc)

## Slide 6
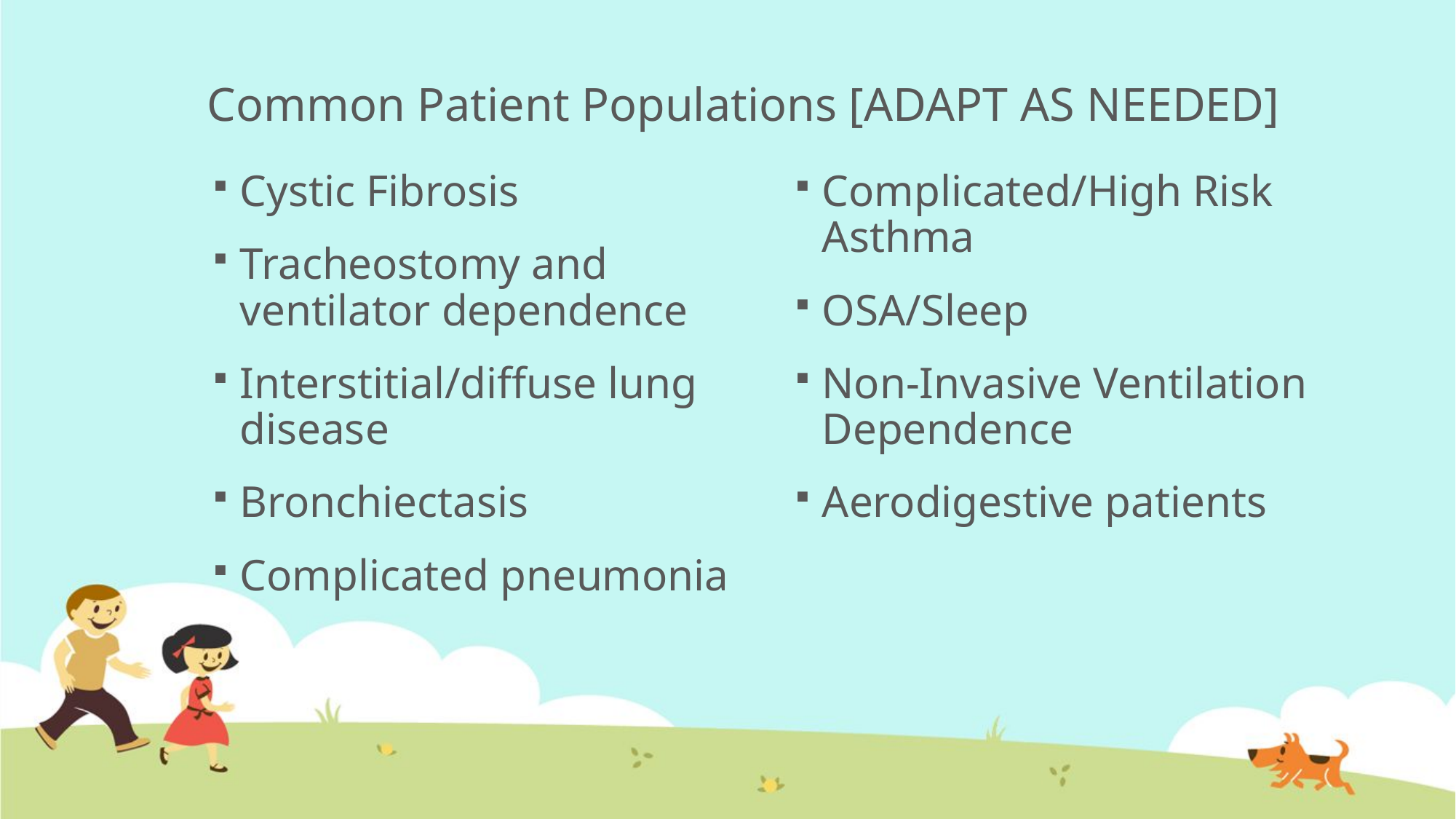

# Common Patient Populations [ADAPT AS NEEDED]
Cystic Fibrosis
Tracheostomy and ventilator dependence
Interstitial/diffuse lung disease
Bronchiectasis
Complicated pneumonia
Complicated/High Risk Asthma
OSA/Sleep
Non-Invasive Ventilation Dependence
Aerodigestive patients

## Slide 7
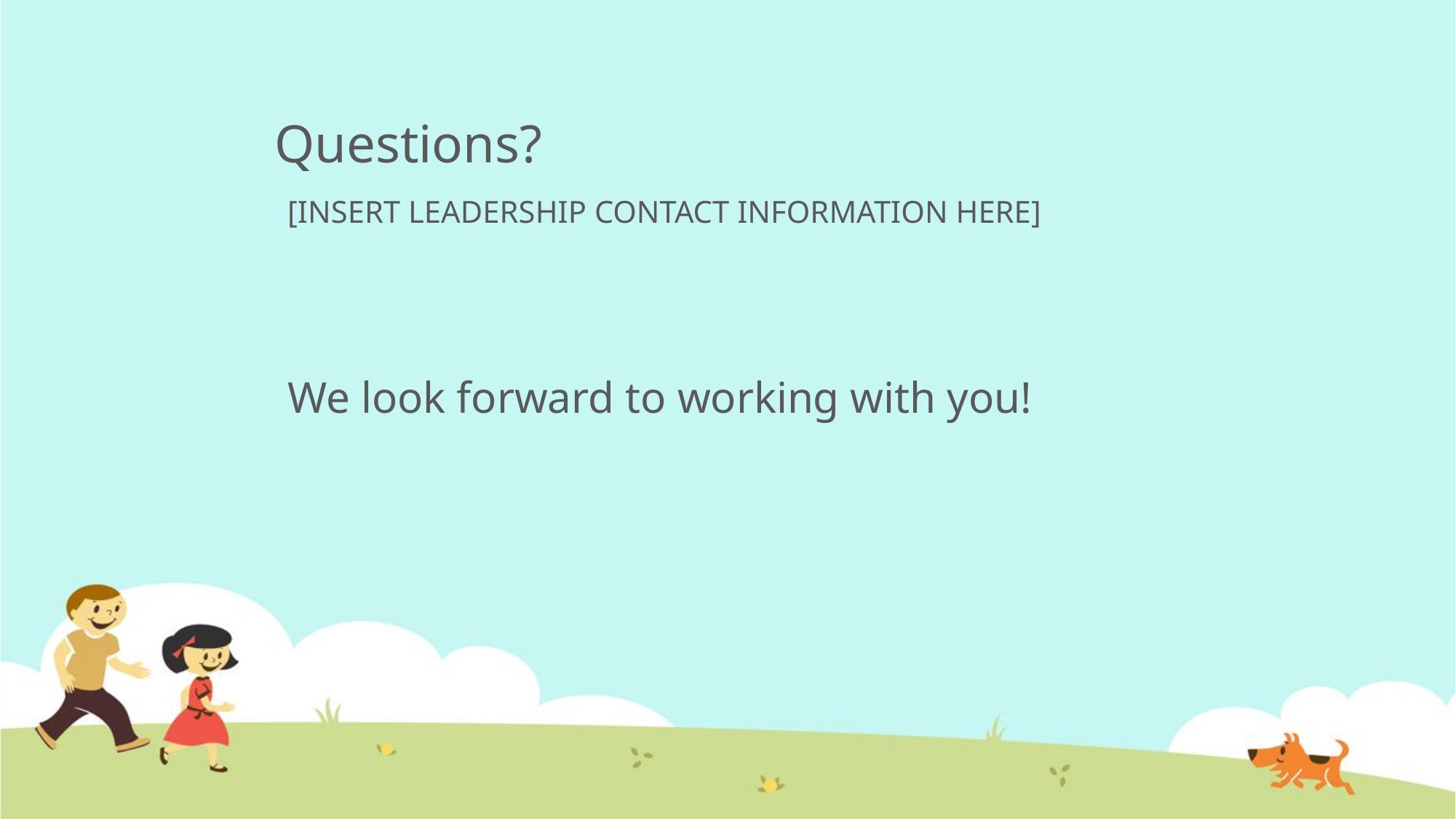

# Questions?
[INSERT LEADERSHIP CONTACT INFORMATION HERE]
We look forward to working with you!
