# Supplementary material for: Pediatric Resident Education in Pulmonary (PREP): A Subspecialty Preparatory Boot Camp Curriculum for Pediatric Residents
Source: MedEdPORTAL. 2021 Jan 7;17:11066. doi: 10.15766/mep_2374-8265.11066 (PMC7809931; doi:10.15766/mep_2374-8265.11066)
Supplement: Supplementary file 1 — Example Agenda.docxOrientation Template.pptxIntroduction to Tracheostomies and Ventilators.pptxCystic Fibrosis JeoPARODY.pptxIntroduction to Airway Clearance and Lung Expansion.pptxInstructor Guide CPT.docxInstructor Guide IS.docxInstructor Guide PEP.docxInstructor Guide PAP.docxInstructor Guide OPEP.docxInstructor Guide Insufflator Exsufflator.docxInstructor Guide HFCWO.docxInstructor Guide IPV.docxPREP Day of Evaluation.docxPREP End of Rotation Evaluation.docxPREP Faculty Feedback Survey.docxPREP Focus Group Guide.docx [file mep_2374-8265.11066-s001.zip › C. Intro to Tracheostomies and Ventilators.pptx]

## Slide 1
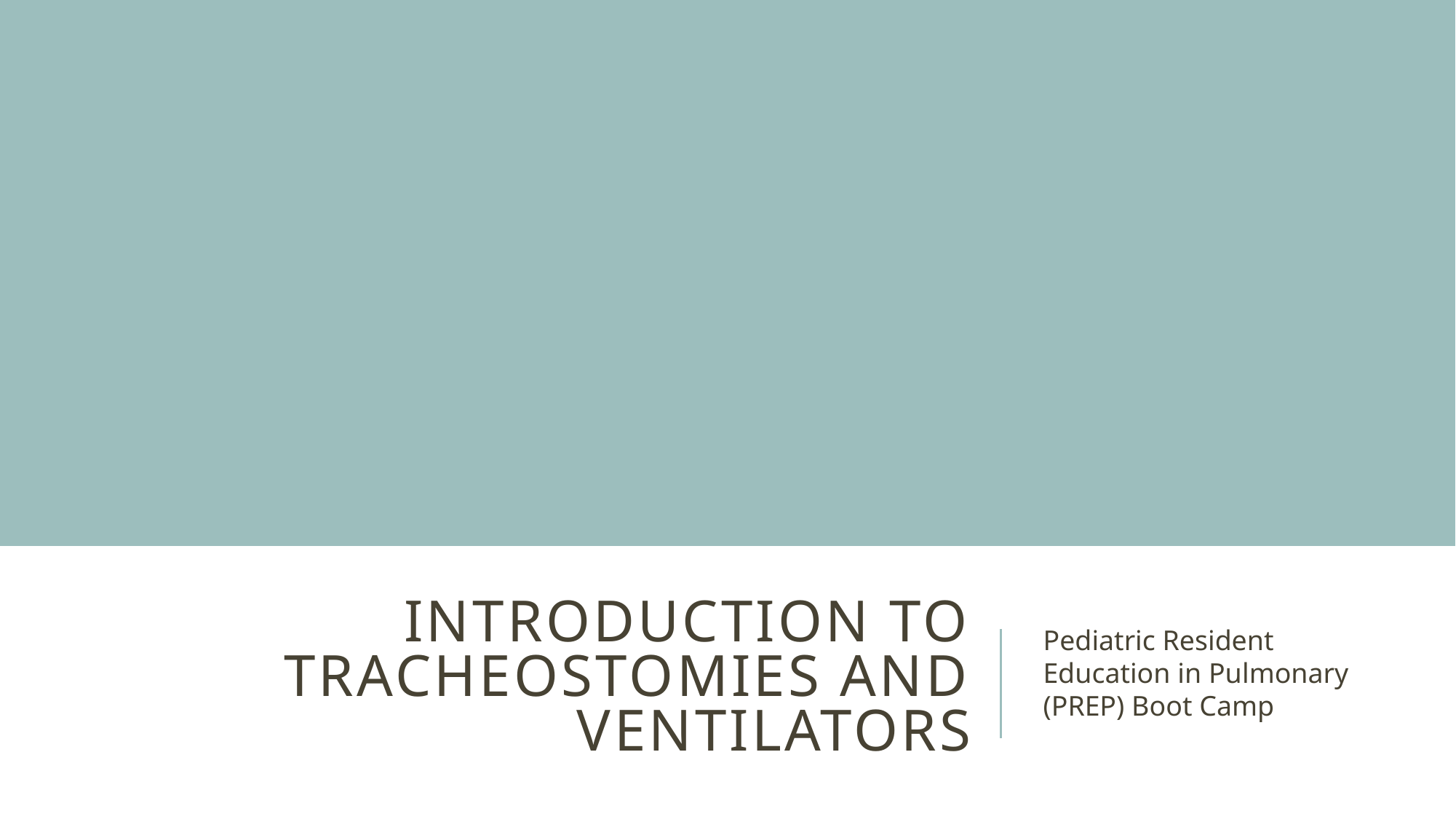

Pediatric Resident Education in Pulmonary (PREP) Boot Camp
# Introduction to Tracheostomies and Ventilators

## Slide 2
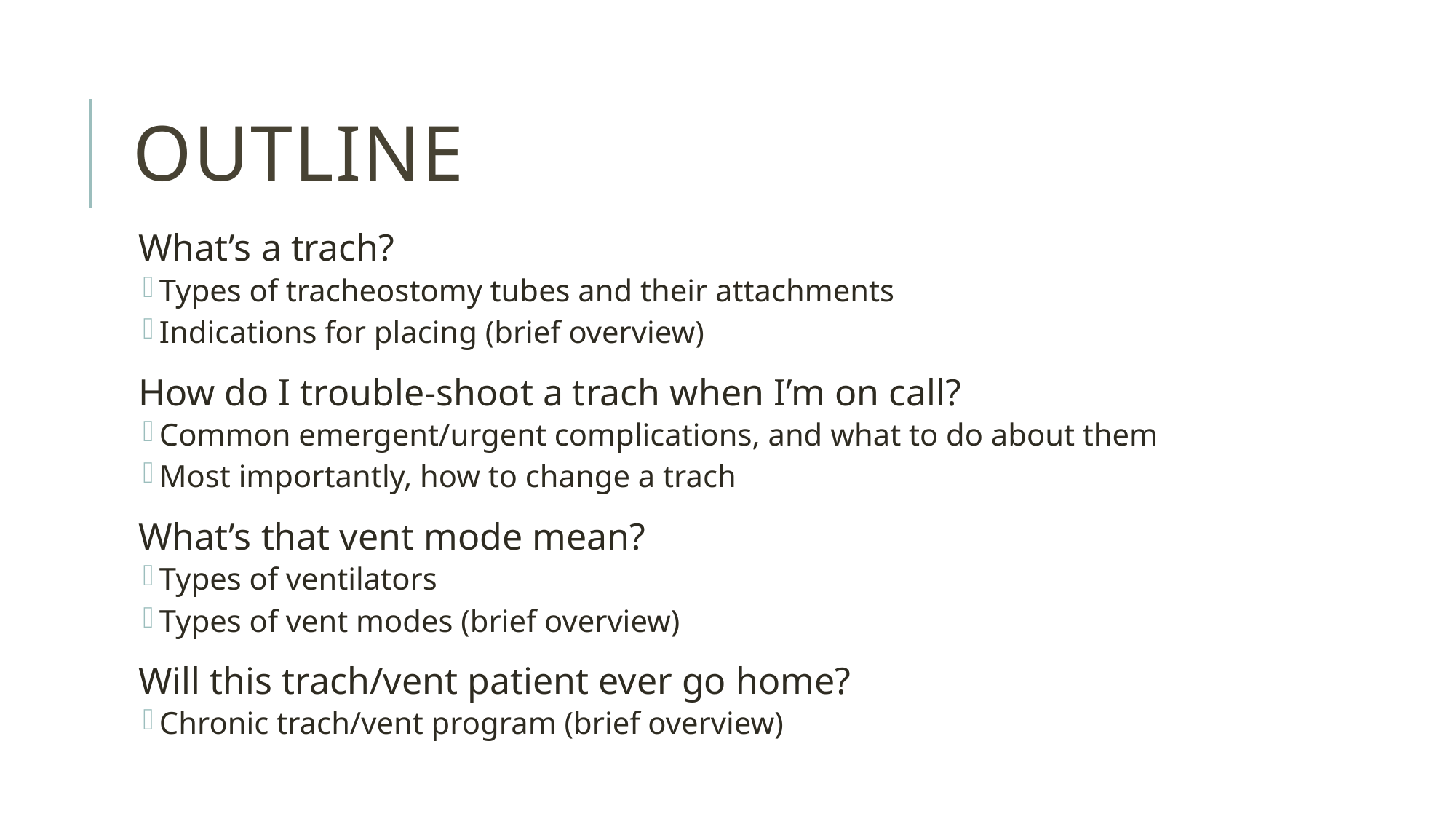

# Outline
What’s a trach?
Types of tracheostomy tubes and their attachments
Indications for placing (brief overview)
How do I trouble-shoot a trach when I’m on call?
Common emergent/urgent complications, and what to do about them
Most importantly, how to change a trach
What’s that vent mode mean?
Types of ventilators
Types of vent modes (brief overview)
Will this trach/vent patient ever go home?
Chronic trach/vent program (brief overview)

## Slide 3
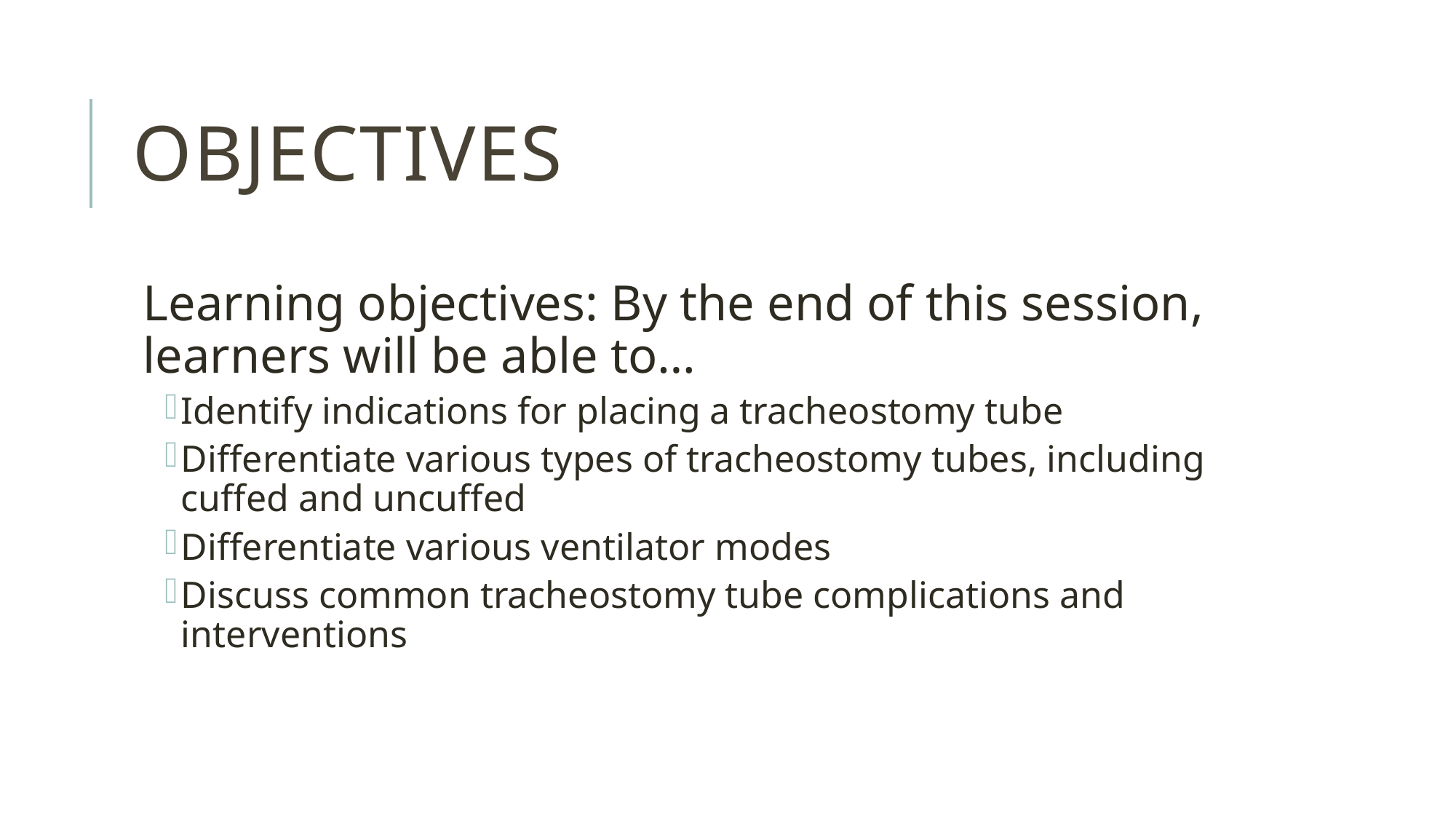

# Objectives
Learning objectives: By the end of this session, learners will be able to…
Identify indications for placing a tracheostomy tube
Differentiate various types of tracheostomy tubes, including cuffed and uncuffed
Differentiate various ventilator modes
Discuss common tracheostomy tube complications and interventions

## Slide 4
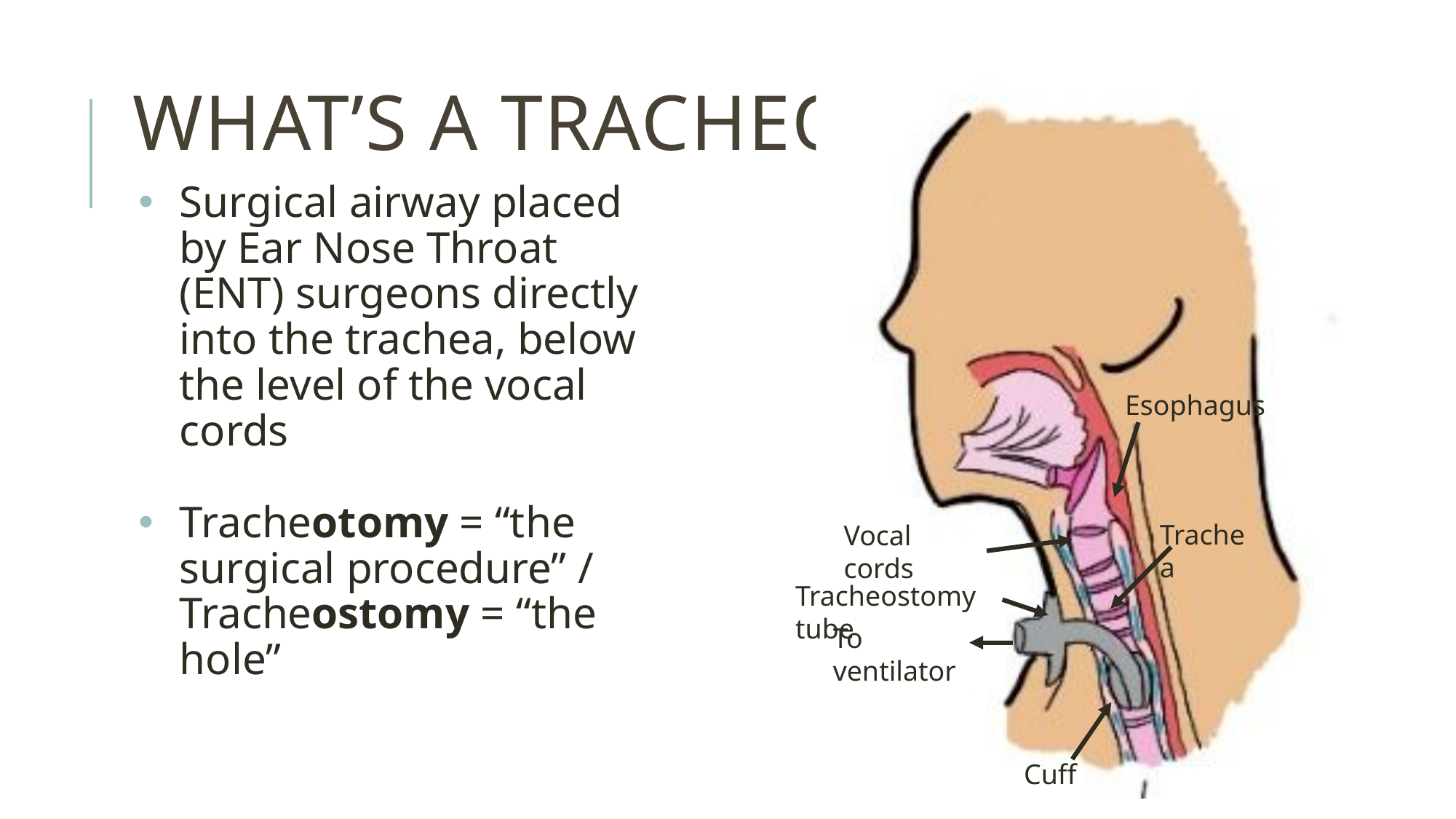

Esophagus
Trachea
Vocal cords
Tracheostomy tube
To ventilator
Cuff
# What’s a tracheostomy?
Surgical airway placed by Ear Nose Throat (ENT) surgeons directly into the trachea, below the level of the vocal cords
Tracheotomy = “the surgical procedure” / Tracheostomy = “the hole”

## Slide 5
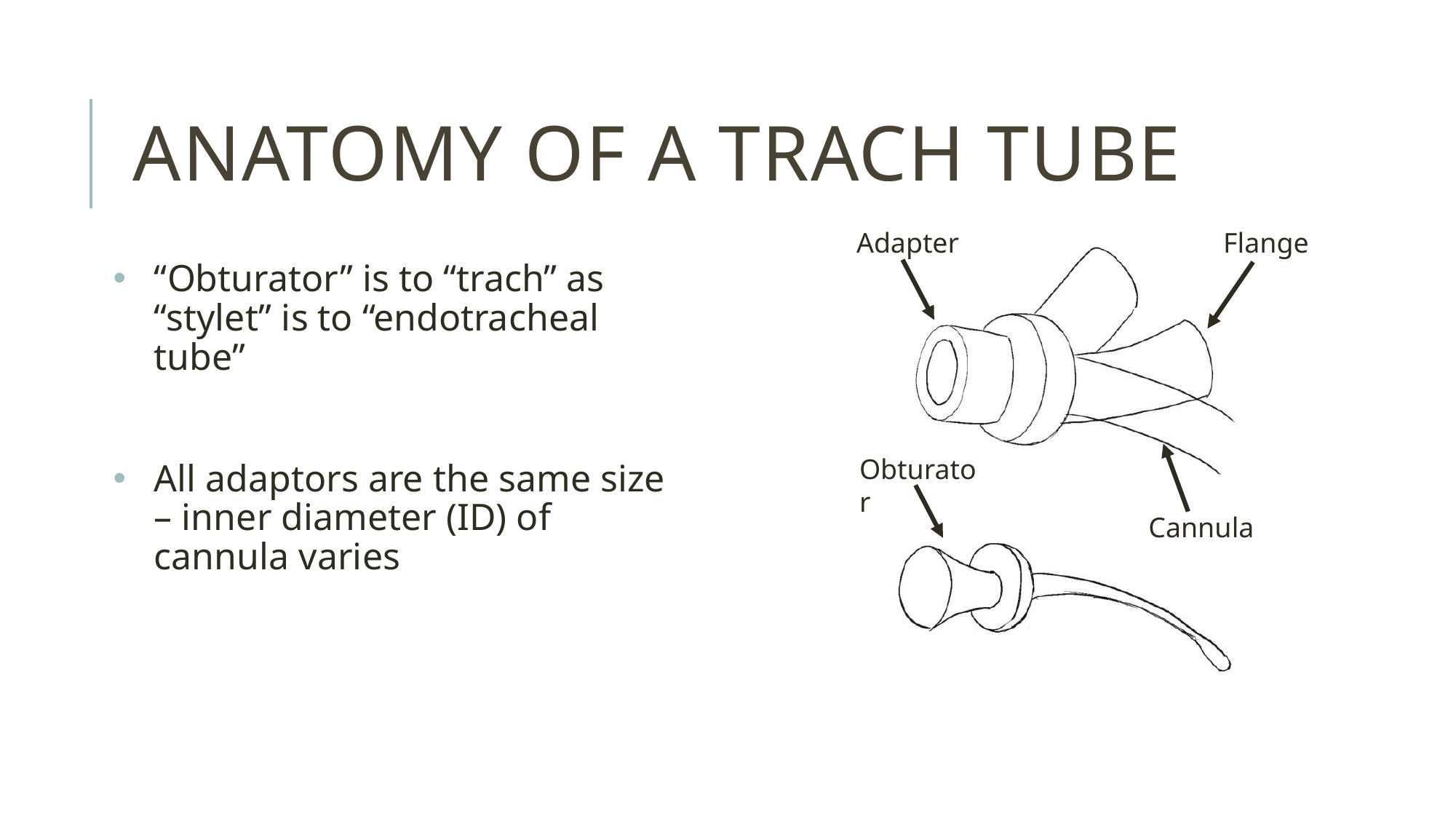

# Anatomy of a trach tube
Adapter
Flange
Obturator
Cannula
“Obturator” is to “trach” as “stylet” is to “endotracheal tube”
All adaptors are the same size – inner diameter (ID) of cannula varies

## Slide 6
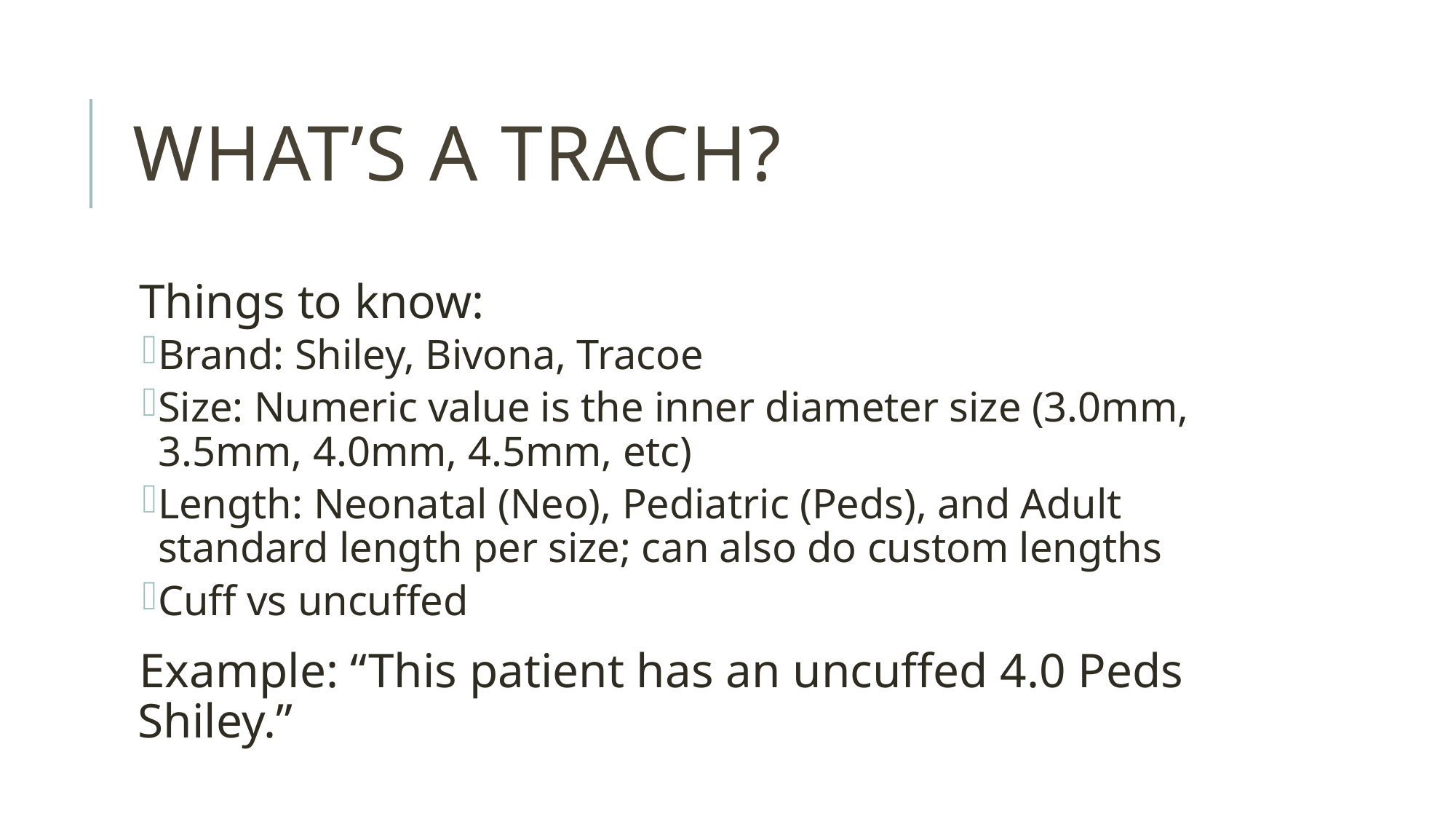

# What’s a trach?
Things to know:
Brand: Shiley, Bivona, Tracoe
Size: Numeric value is the inner diameter size (3.0mm, 3.5mm, 4.0mm, 4.5mm, etc)
Length: Neonatal (Neo), Pediatric (Peds), and Adult standard length per size; can also do custom lengths
Cuff vs uncuffed
Example: “This patient has an uncuffed 4.0 Peds Shiley.”

## Slide 7
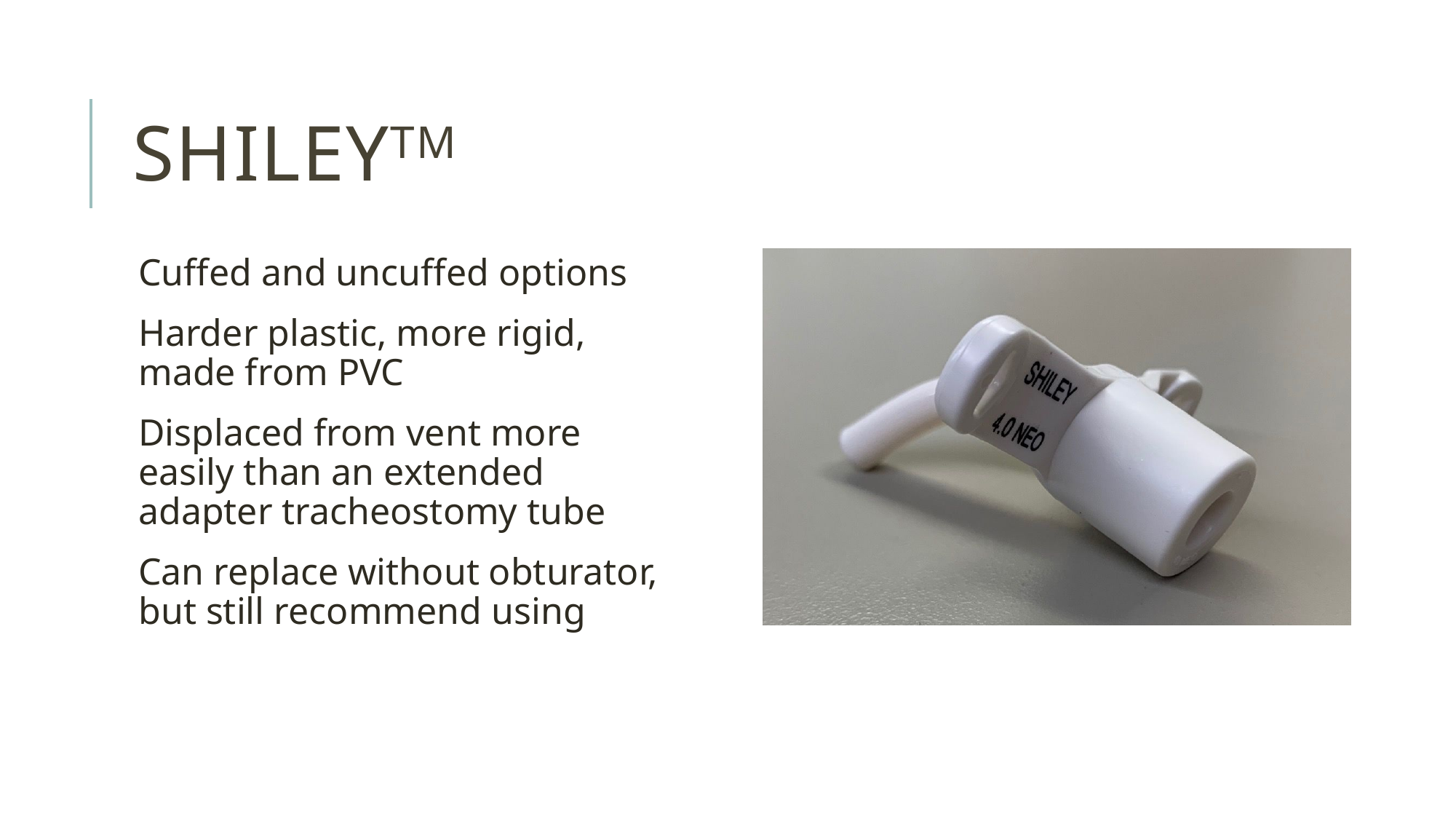

# ShileYTM
Cuffed and uncuffed options
Harder plastic, more rigid, made from PVC
Displaced from vent more easily than an extended adapter tracheostomy tube
Can replace without obturator, but still recommend using

## Slide 8
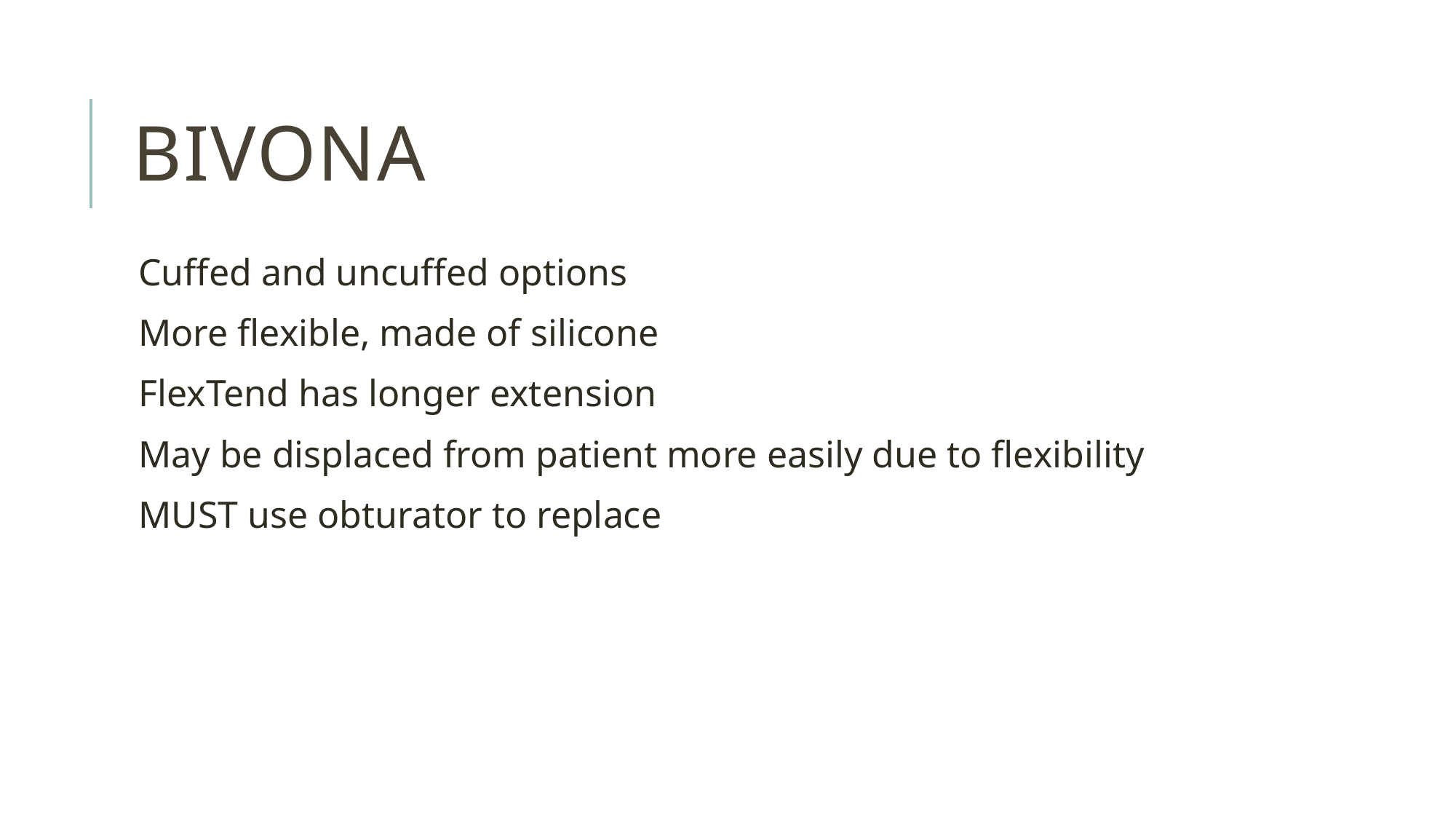

# Bivona
Cuffed and uncuffed options
More flexible, made of silicone
FlexTend has longer extension
May be displaced from patient more easily due to flexibility
MUST use obturator to replace

## Slide 9
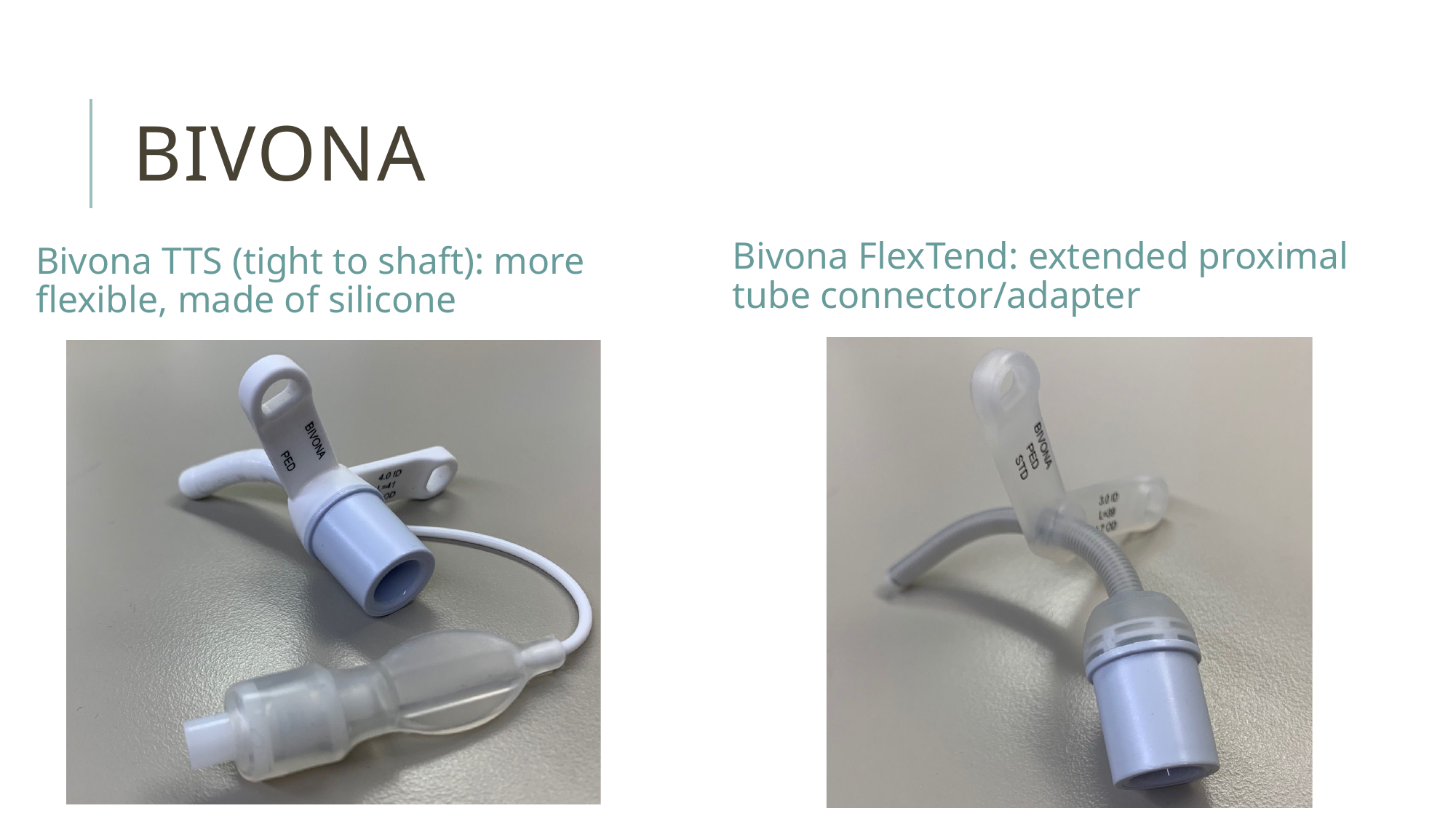

# Bivona
Bivona TTS (tight to shaft): more flexible, made of silicone
Bivona FlexTend: extended proximal tube connector/adapter

## Slide 10
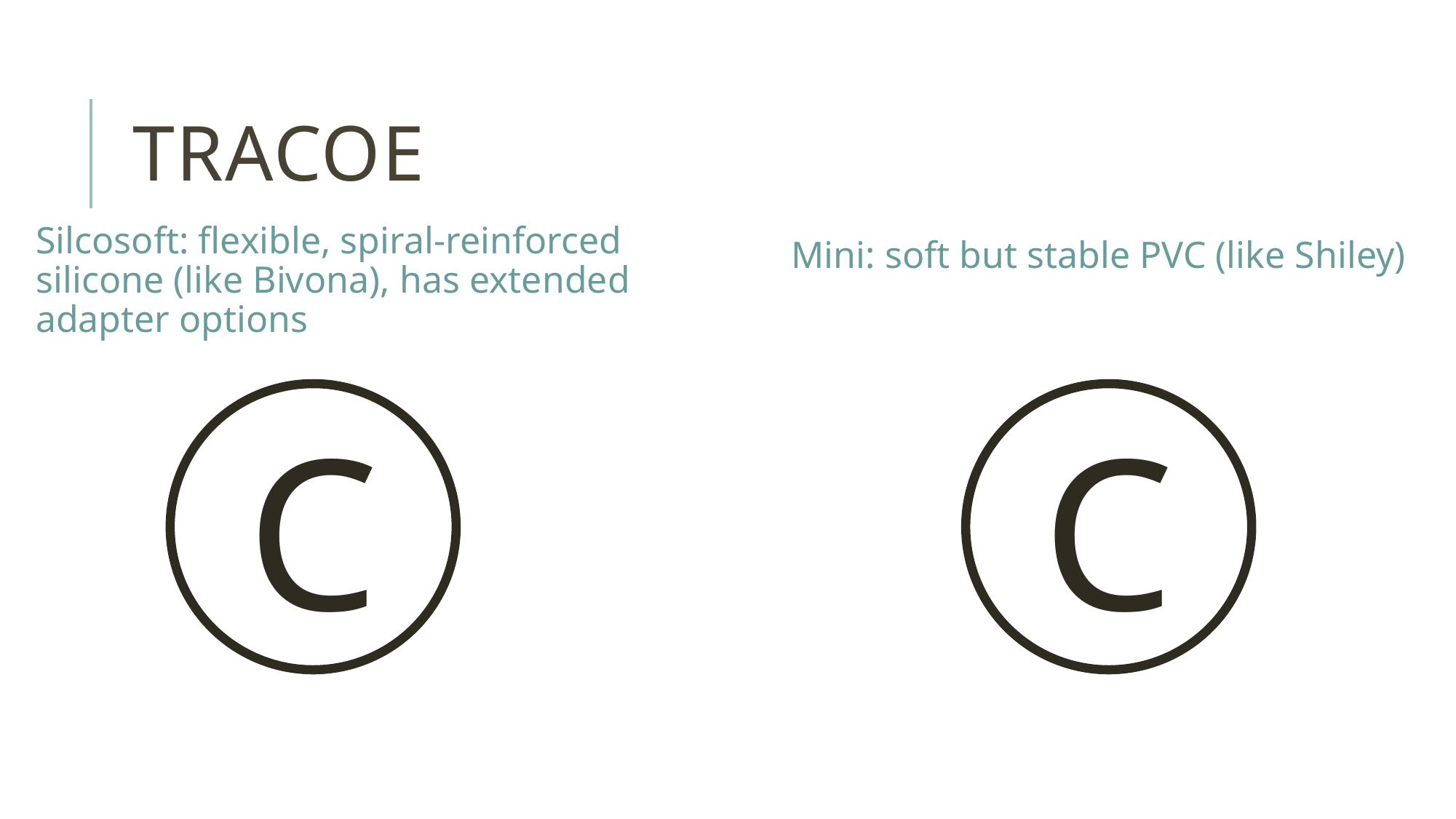

# Tracoe
Silcosoft: flexible, spiral-reinforced silicone (like Bivona), has extended adapter options
Mini: soft but stable PVC (like Shiley)
C
C

## Slide 11
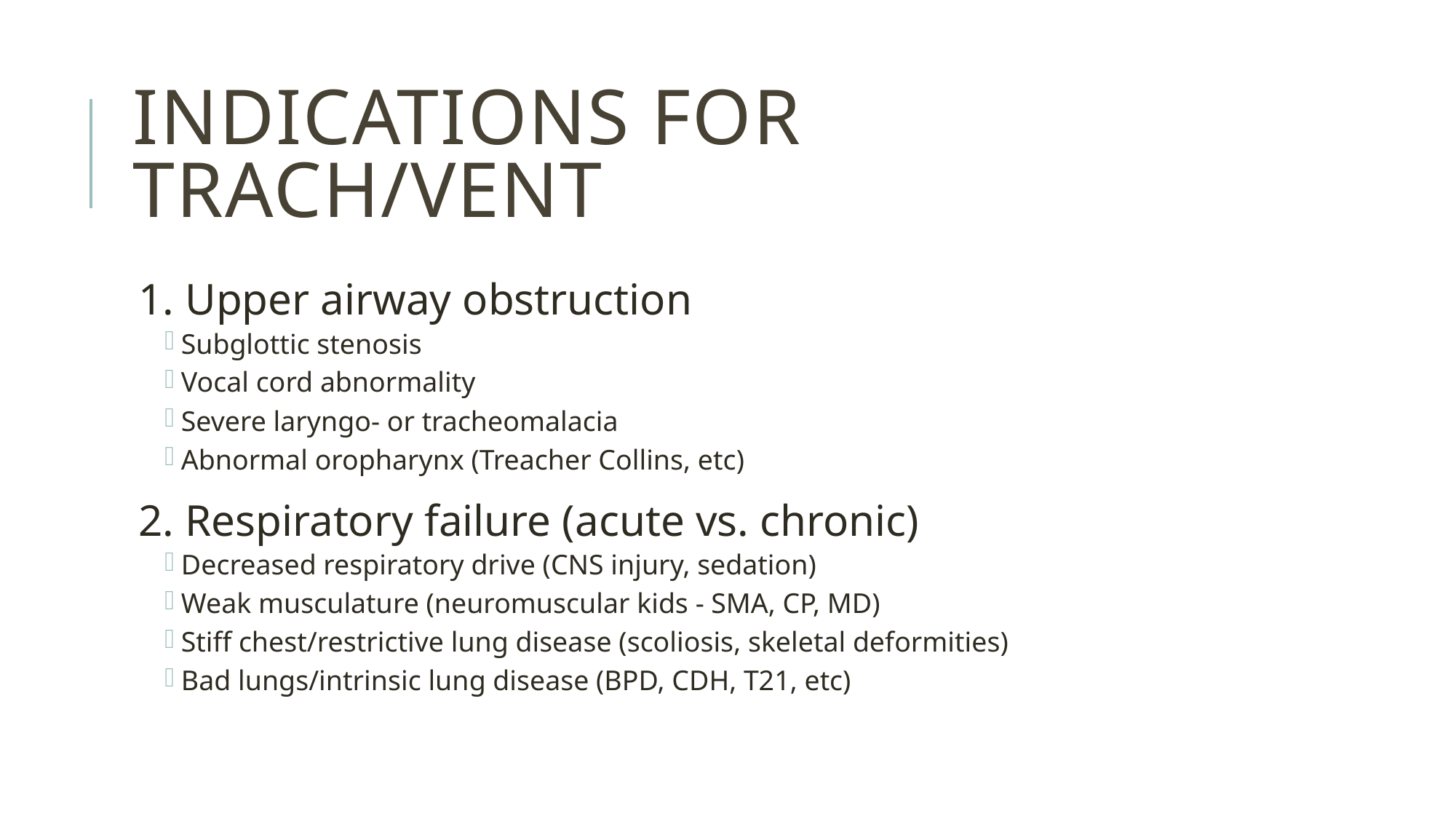

# Indications for trach/VENt
1. Upper airway obstruction
Subglottic stenosis
Vocal cord abnormality
Severe laryngo- or tracheomalacia
Abnormal oropharynx (Treacher Collins, etc)
2. Respiratory failure (acute vs. chronic)
Decreased respiratory drive (CNS injury, sedation)
Weak musculature (neuromuscular kids - SMA, CP, MD)
Stiff chest/restrictive lung disease (scoliosis, skeletal deformities)
Bad lungs/intrinsic lung disease (BPD, CDH, T21, etc)

## Slide 12
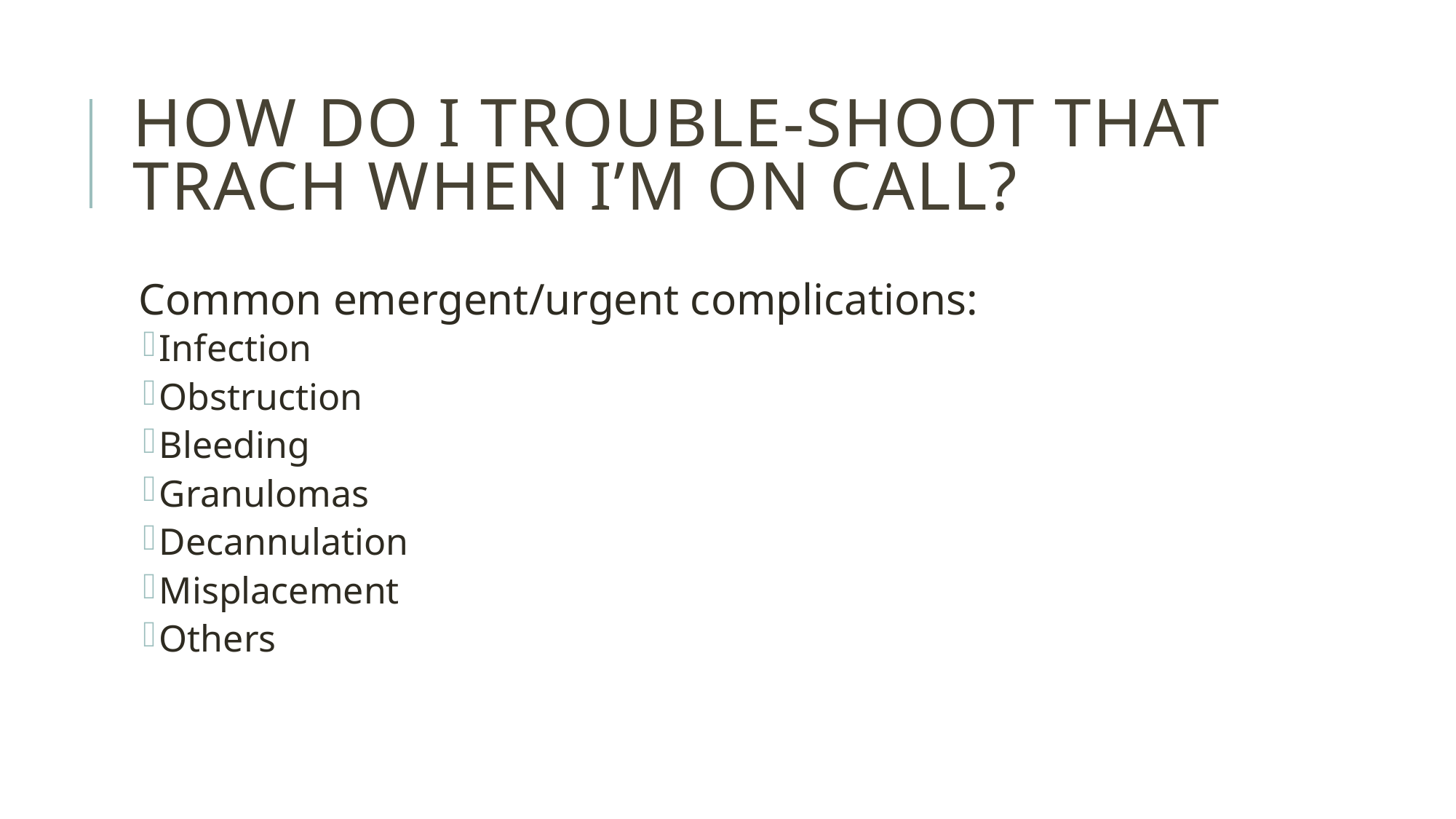

# How do I trouble-shoot that trach when I’m on call?
Common emergent/urgent complications:
Infection
Obstruction
Bleeding
Granulomas
Decannulation
Misplacement
Others

## Slide 13
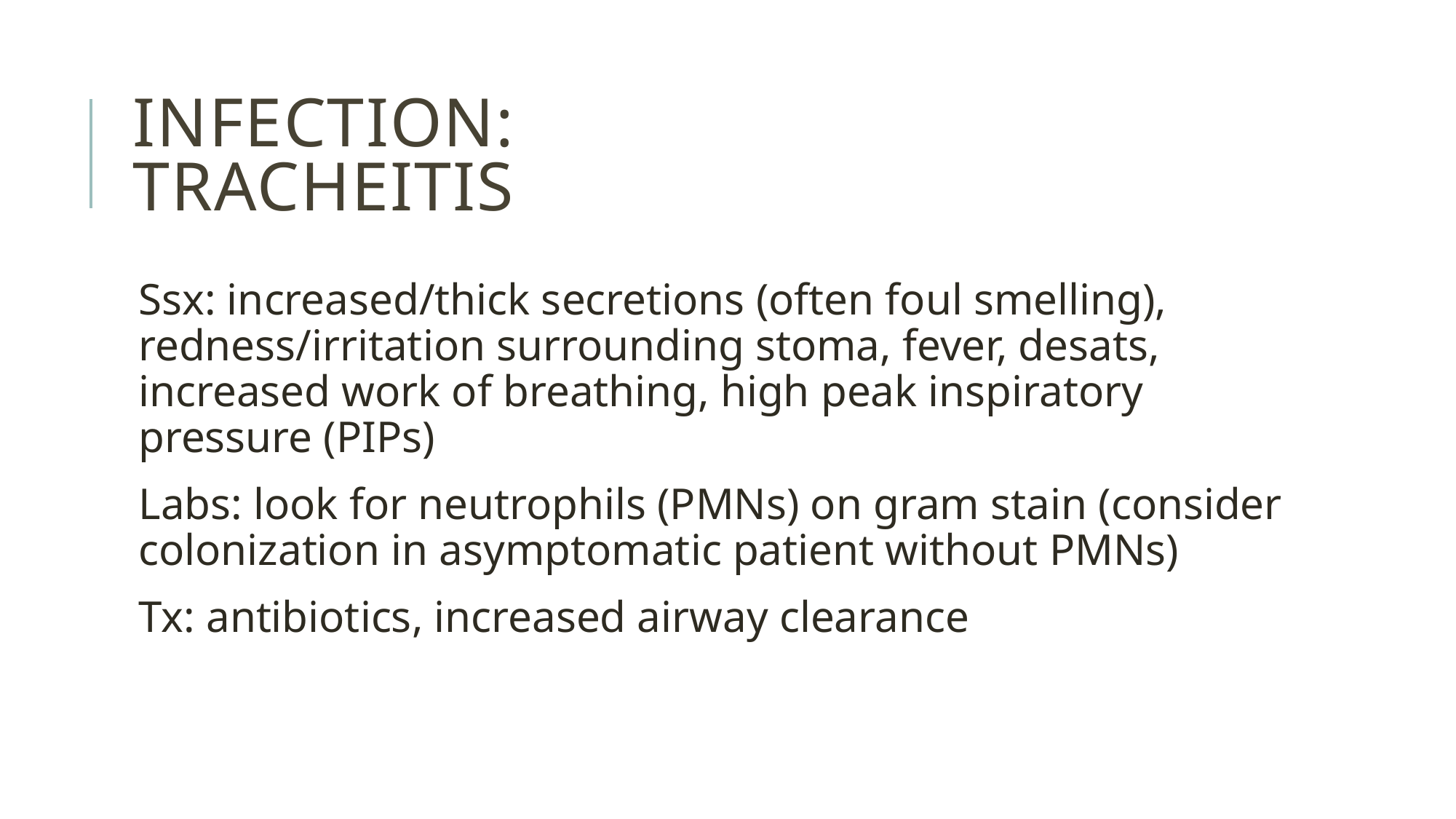

# Infection: tracheitis
Ssx: increased/thick secretions (often foul smelling), redness/irritation surrounding stoma, fever, desats, increased work of breathing, high peak inspiratory pressure (PIPs)
Labs: look for neutrophils (PMNs) on gram stain (consider colonization in asymptomatic patient without PMNs)
Tx: antibiotics, increased airway clearance

## Slide 14
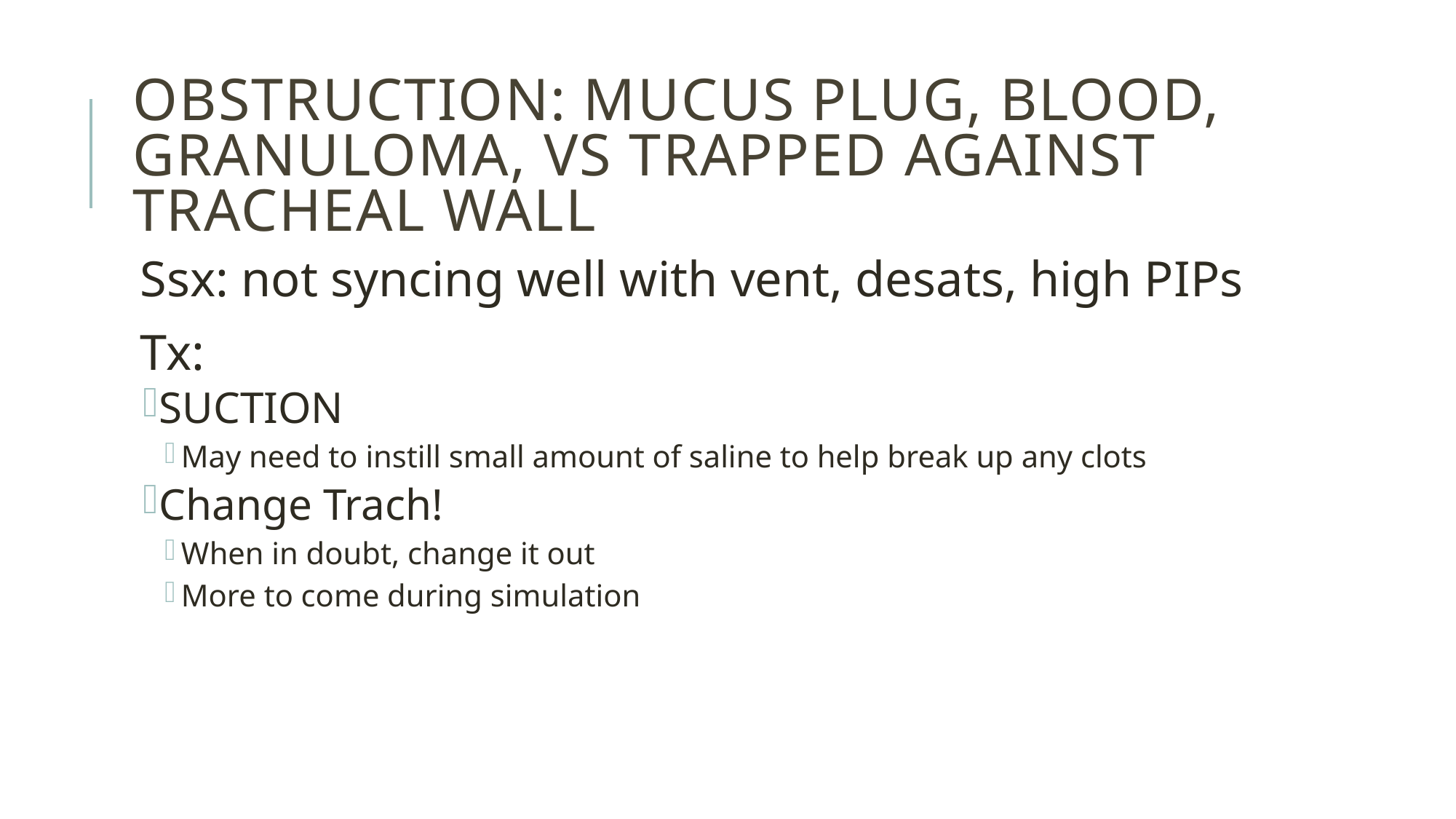

# Obstruction: mucus plug, blood, Granuloma, vs trapped against tracheal wall
Ssx: not syncing well with vent, desats, high PIPs
Tx:
SUCTION
May need to instill small amount of saline to help break up any clots
Change Trach!
When in doubt, change it out
More to come during simulation

## Slide 15
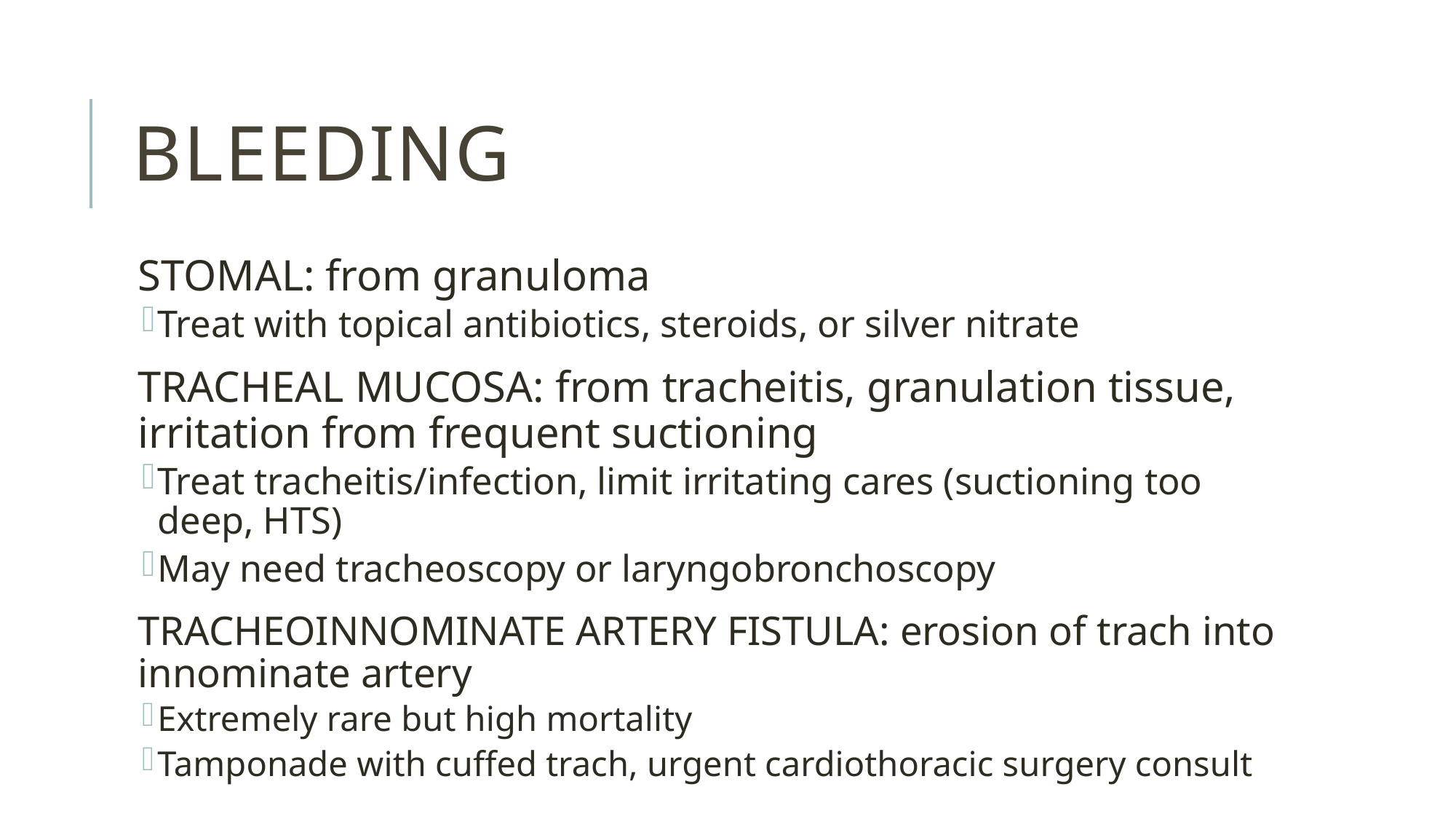

# BLEEDING
STOMAL: from granuloma
Treat with topical antibiotics, steroids, or silver nitrate
TRACHEAL MUCOSA: from tracheitis, granulation tissue, irritation from frequent suctioning
Treat tracheitis/infection, limit irritating cares (suctioning too deep, HTS)
May need tracheoscopy or laryngobronchoscopy
TRACHEOINNOMINATE ARTERY FISTULA: erosion of trach into innominate artery
Extremely rare but high mortality
Tamponade with cuffed trach, urgent cardiothoracic surgery consult

## Slide 16
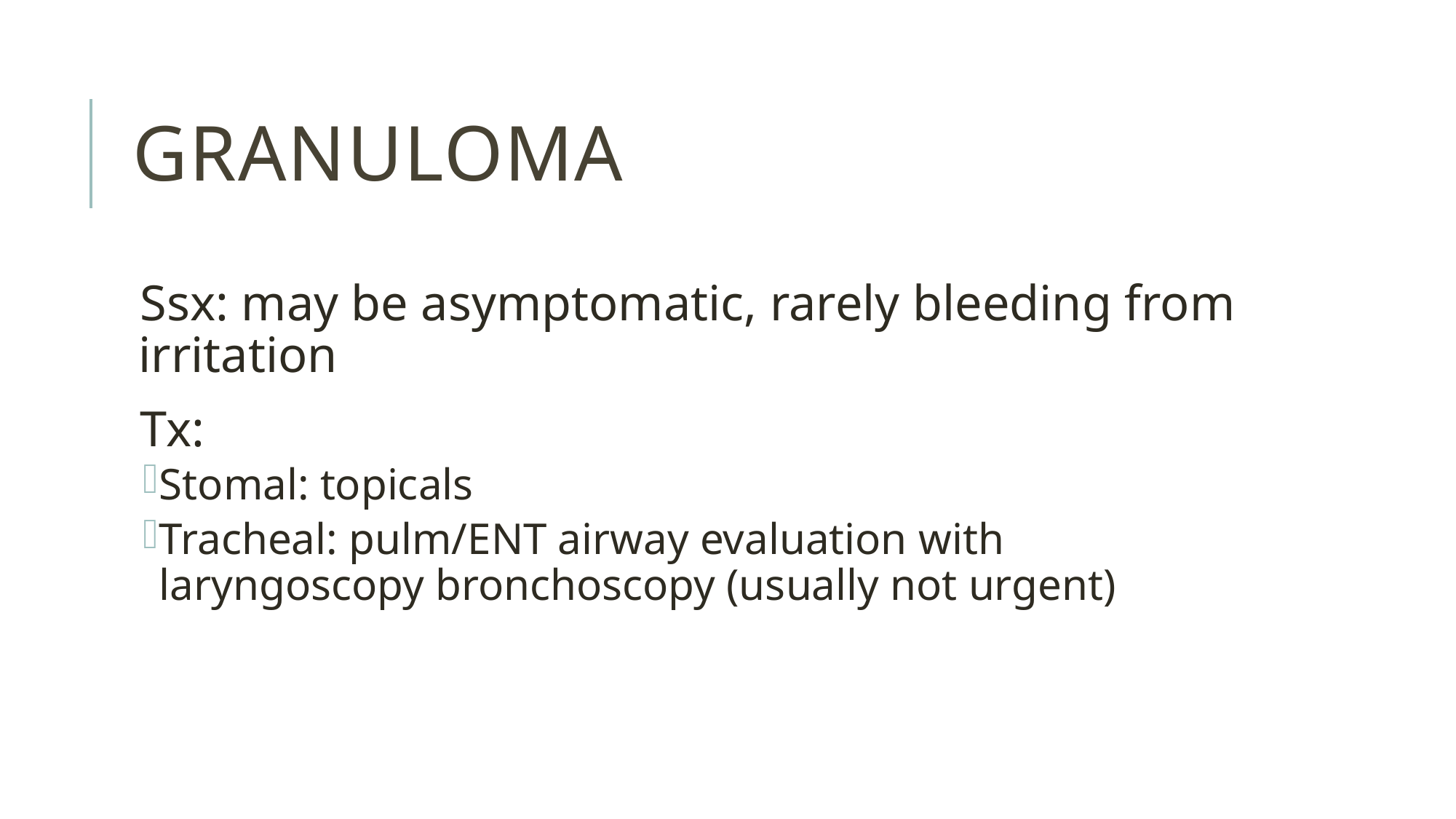

# Granuloma
Ssx: may be asymptomatic, rarely bleeding from irritation
Tx:
Stomal: topicals
Tracheal: pulm/ENT airway evaluation with laryngoscopy bronchoscopy (usually not urgent)

## Slide 17
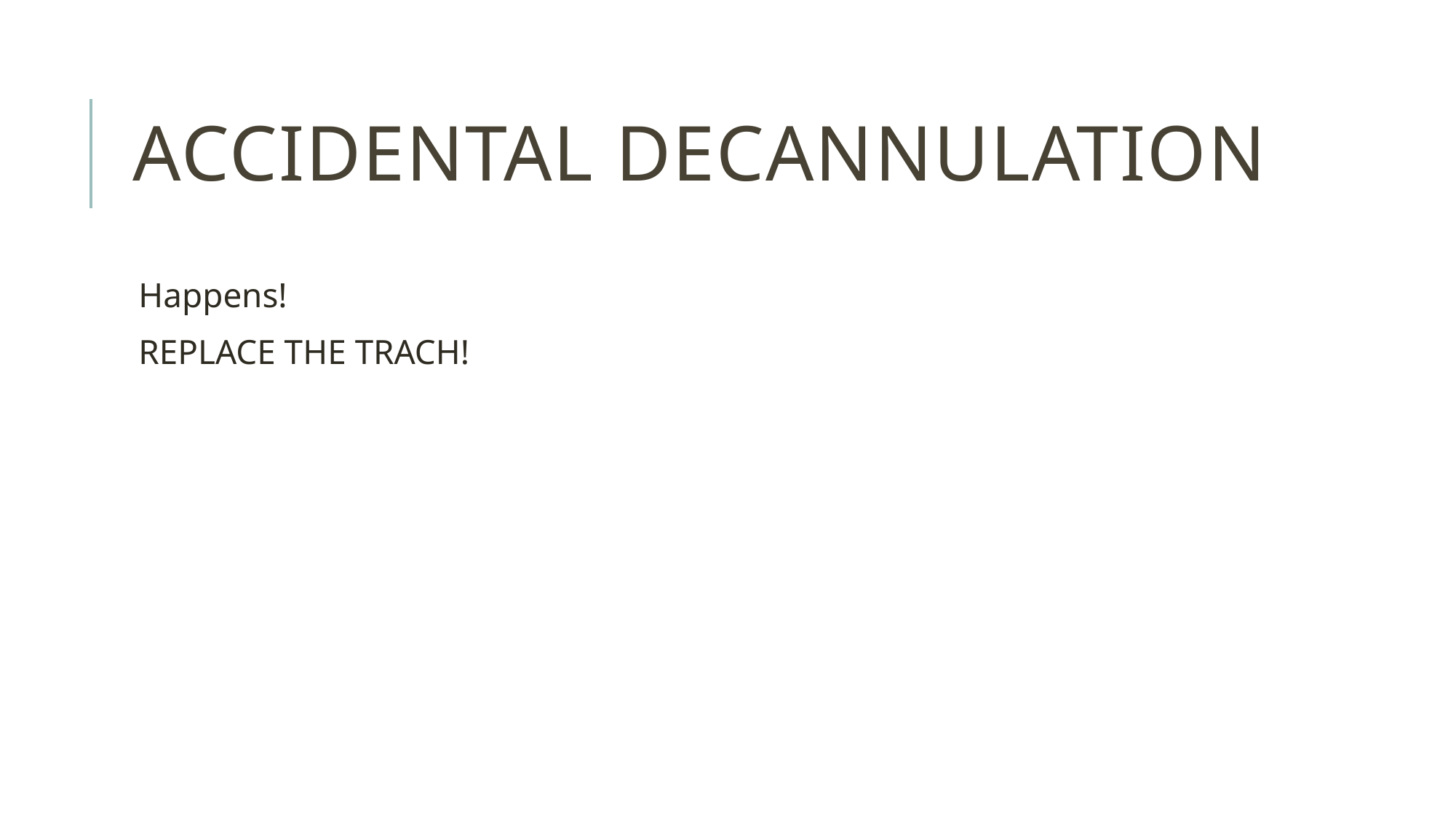

# Accidental Decannulation
Happens!
REPLACE THE TRACH!

## Slide 18
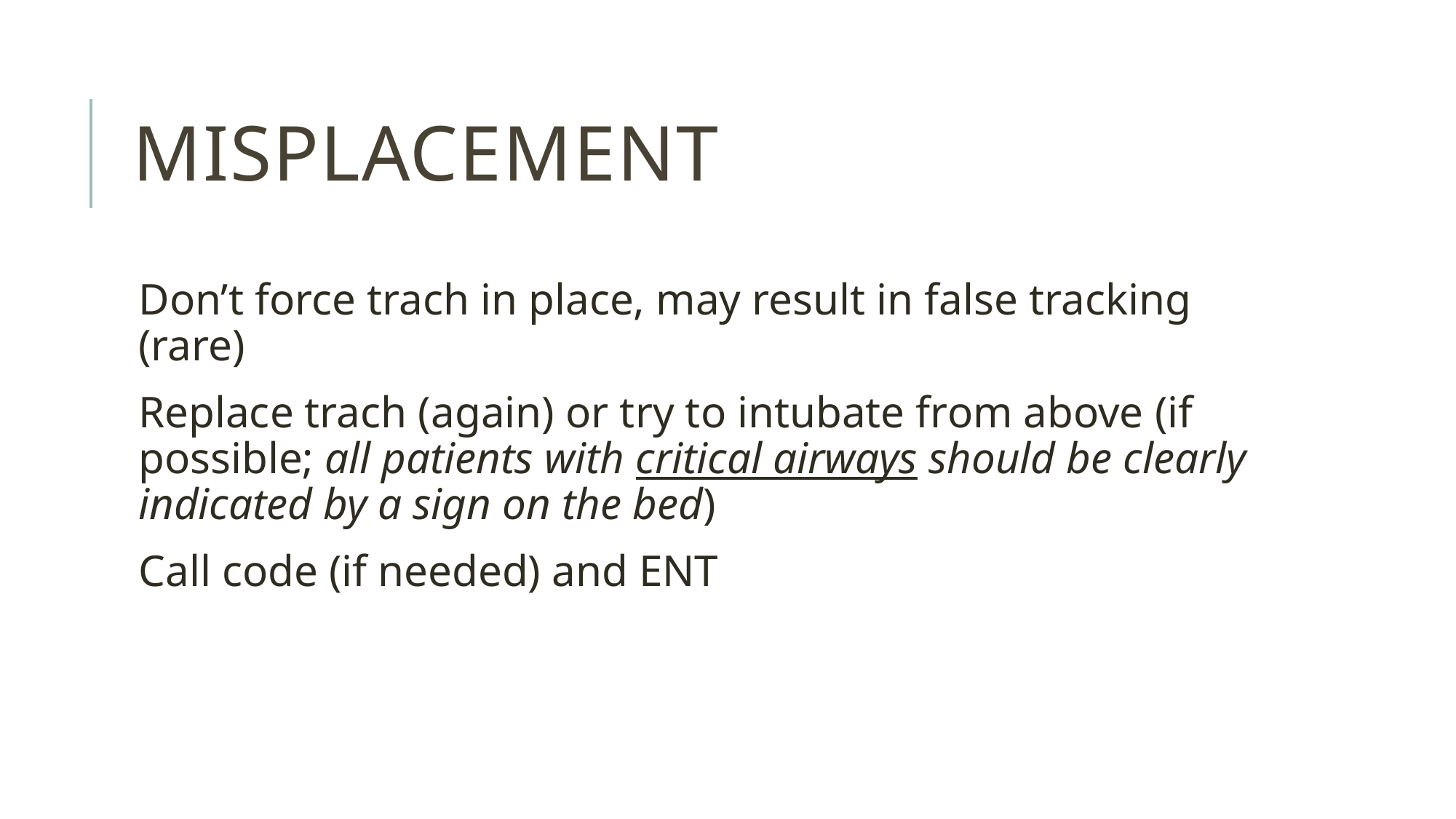

# Misplacement
Don’t force trach in place, may result in false tracking (rare)
Replace trach (again) or try to intubate from above (if possible; all patients with critical airways should be clearly indicated by a sign on the bed)
Call code (if needed) and ENT

## Slide 19
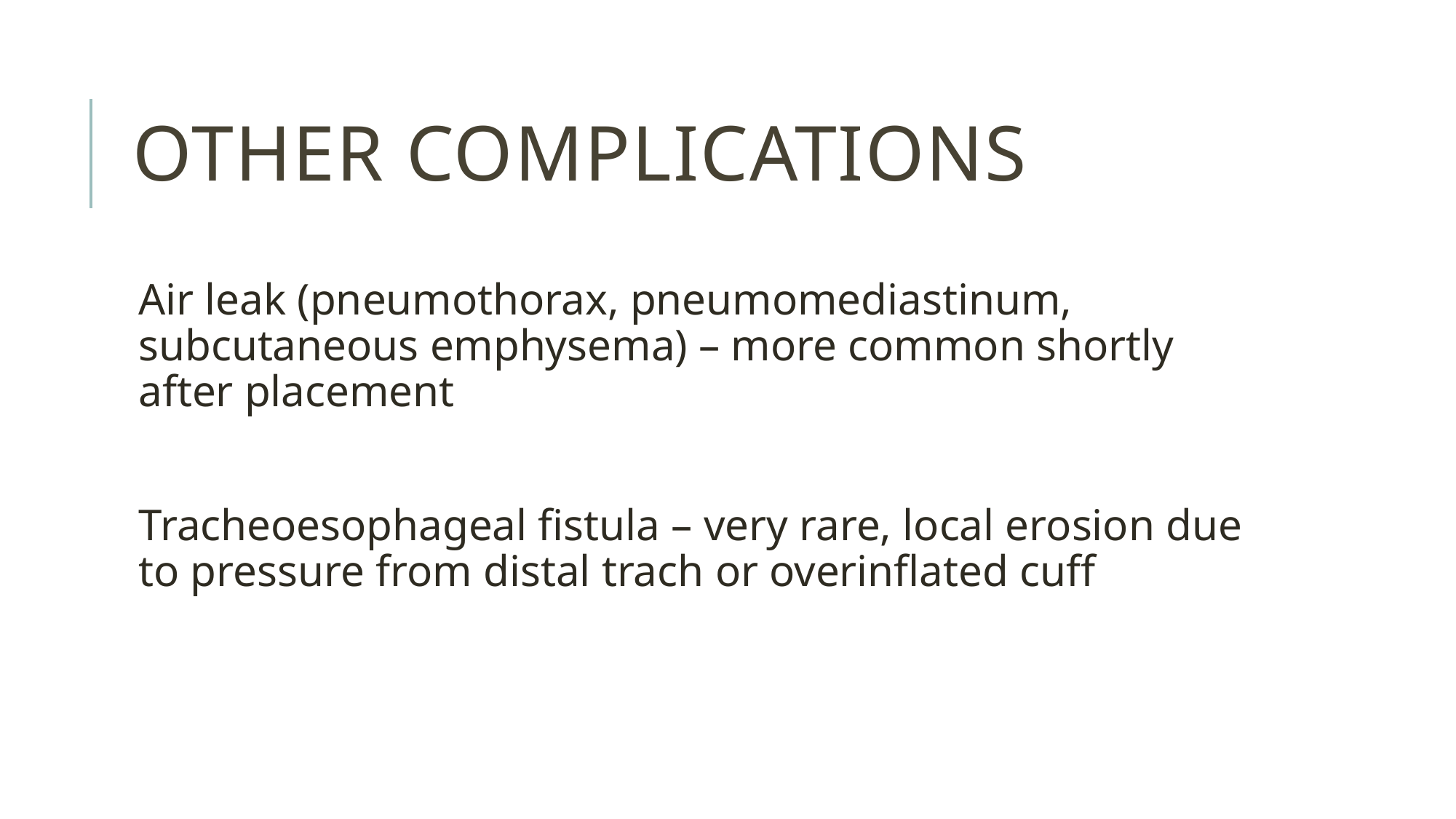

# Other Complications
Air leak (pneumothorax, pneumomediastinum, subcutaneous emphysema) – more common shortly after placement
Tracheoesophageal fistula – very rare, local erosion due to pressure from distal trach or overinflated cuff

## Slide 20
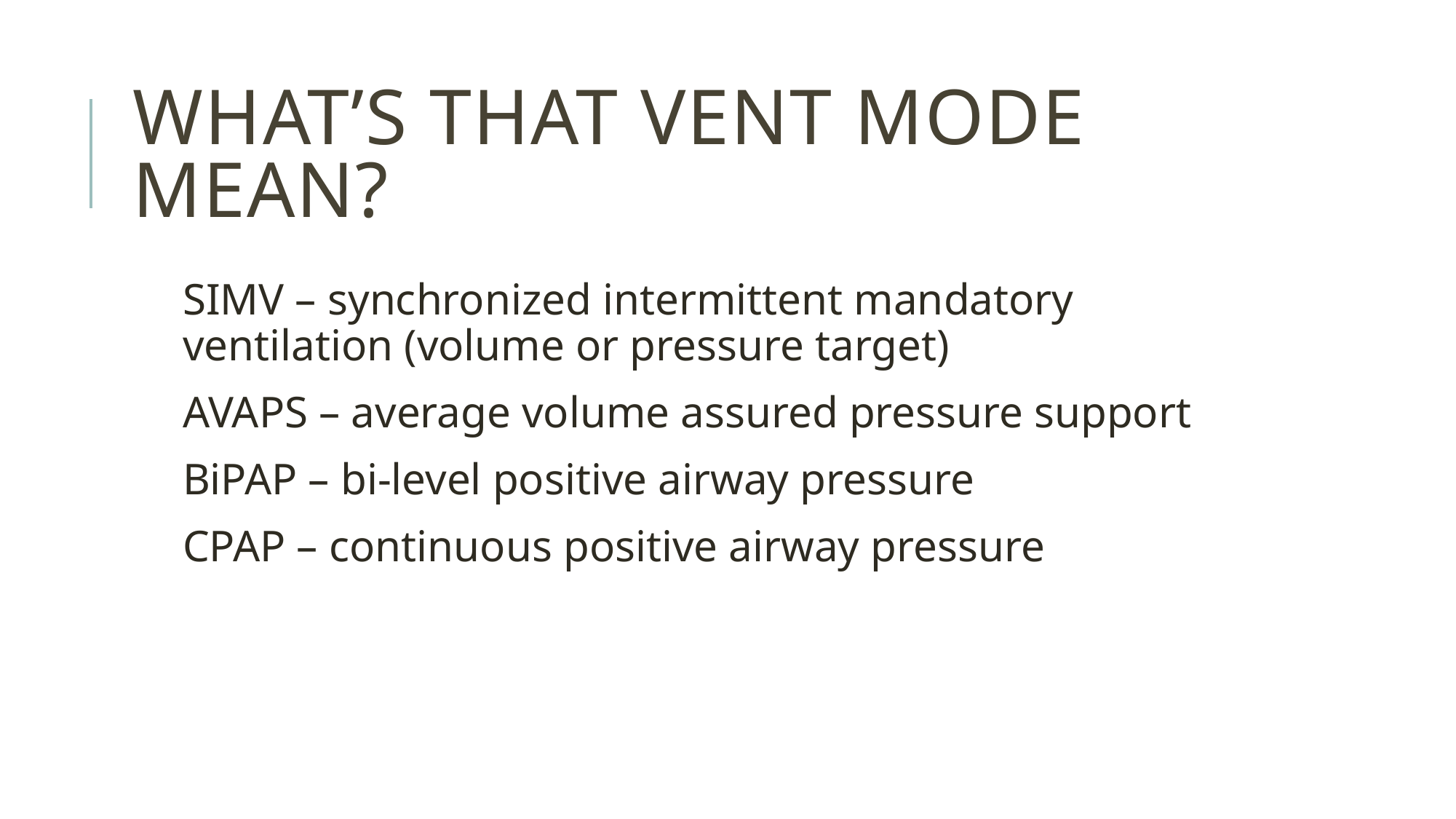

# What’s that vent mode mean?
SIMV – synchronized intermittent mandatory ventilation (volume or pressure target)
AVAPS – average volume assured pressure support
BiPAP – bi-level positive airway pressure
CPAP – continuous positive airway pressure

## Slide 21
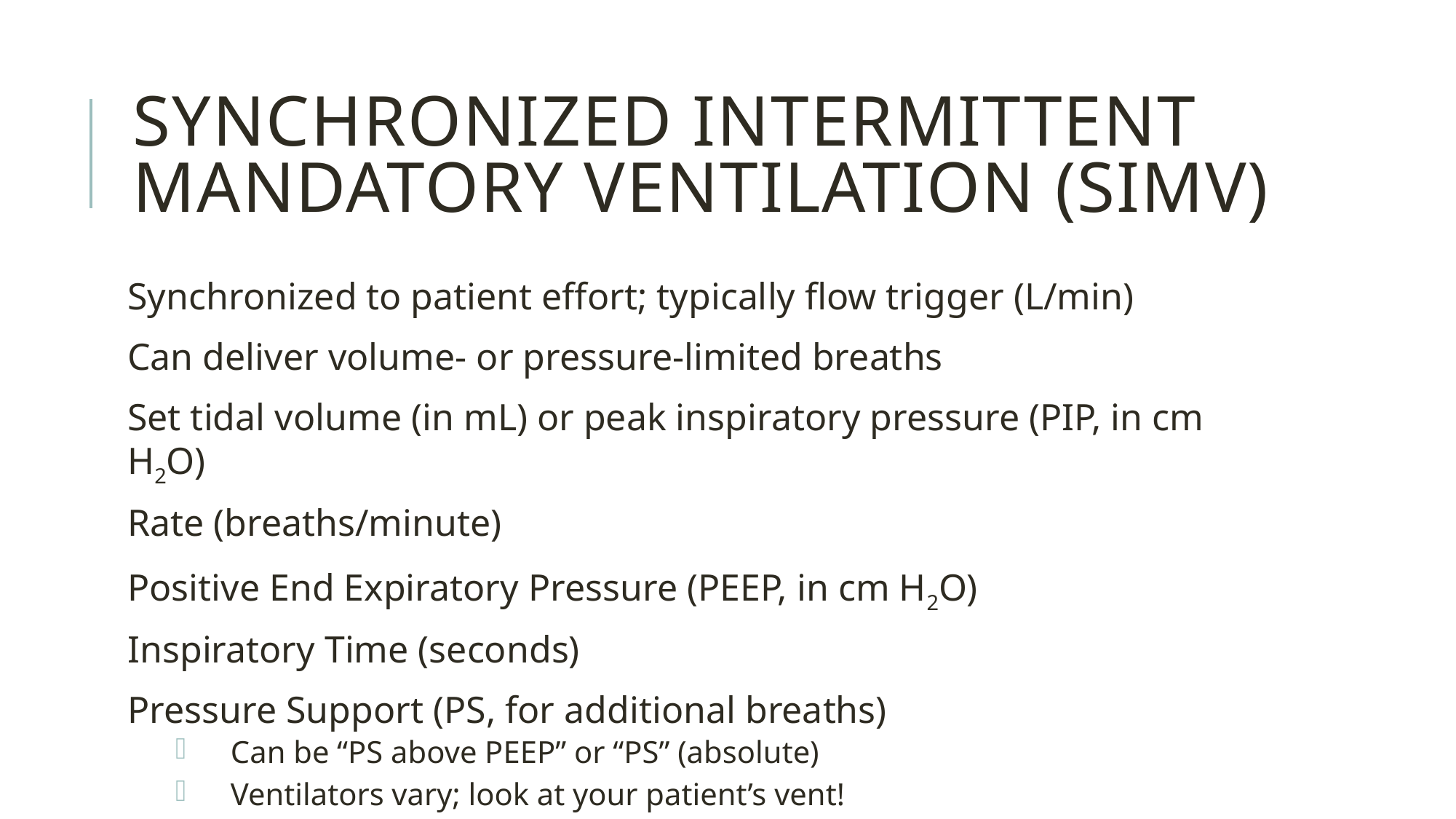

# Synchronized Intermittent Mandatory Ventilation (SIMV)
Synchronized to patient effort; typically flow trigger (L/min)
Can deliver volume- or pressure-limited breaths
Set tidal volume (in mL) or peak inspiratory pressure (PIP, in cm H2O)
Rate (breaths/minute)
Positive End Expiratory Pressure (PEEP, in cm H2O)
Inspiratory Time (seconds)
Pressure Support (PS, for additional breaths)
Can be “PS above PEEP” or “PS” (absolute)
Ventilators vary; look at your patient’s vent!

## Slide 22
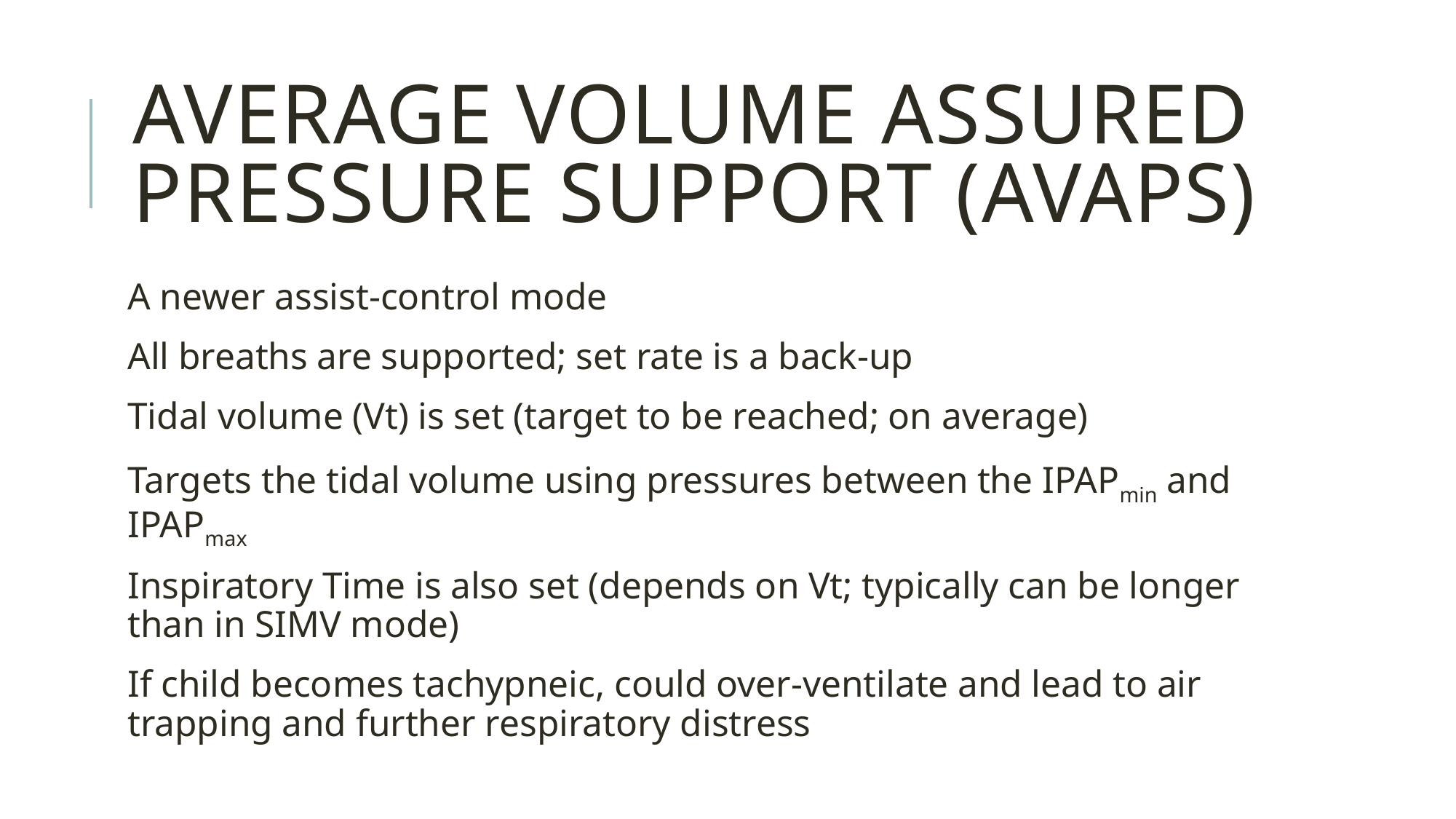

# Average Volume Assured Pressure Support (AVAPS)
A newer assist-control mode
All breaths are supported; set rate is a back-up
Tidal volume (Vt) is set (target to be reached; on average)
Targets the tidal volume using pressures between the IPAPmin and IPAPmax
Inspiratory Time is also set (depends on Vt; typically can be longer than in SIMV mode)
If child becomes tachypneic, could over-ventilate and lead to air trapping and further respiratory distress

## Slide 23
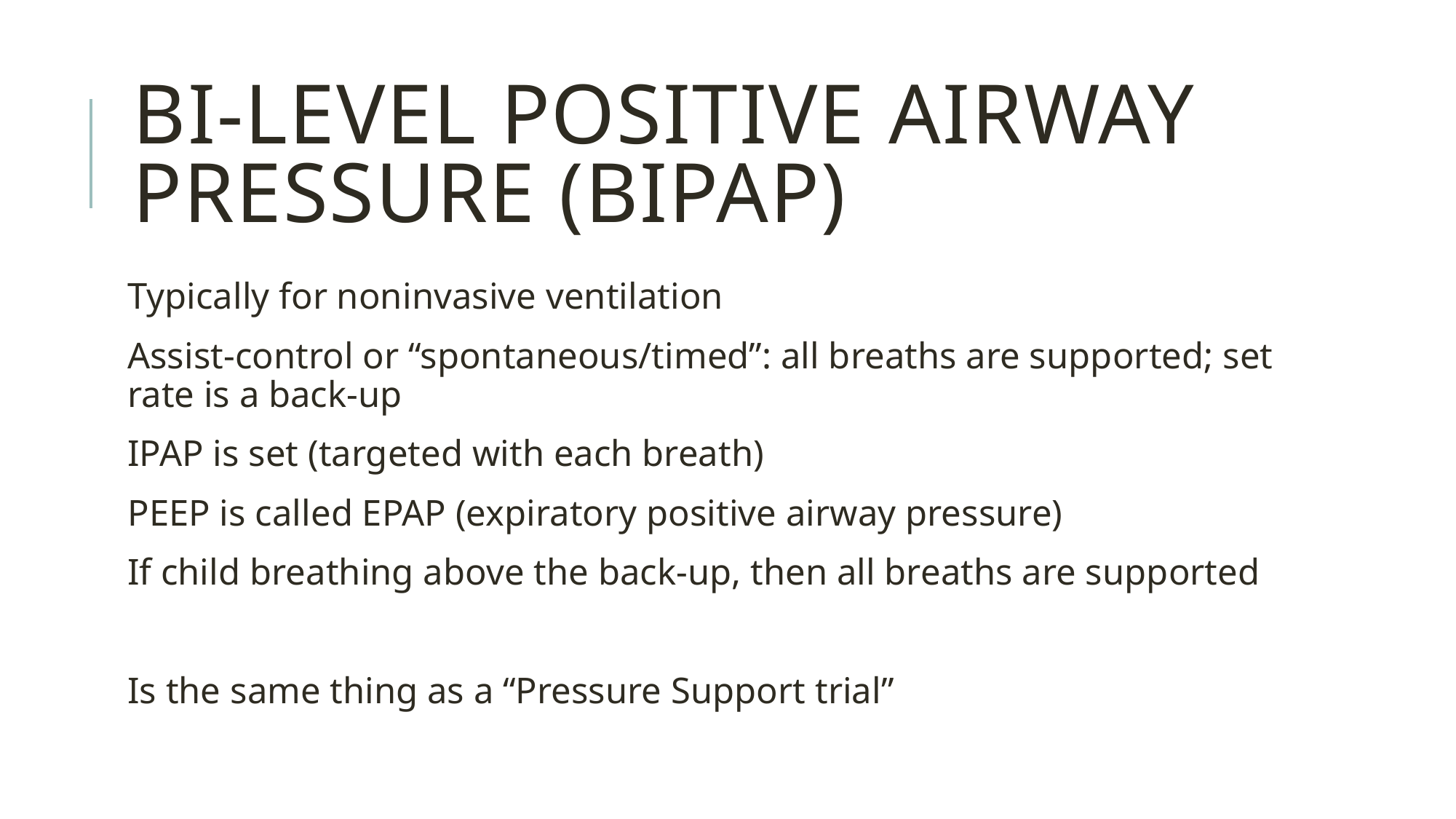

# Bi-Level Positive Airway Pressure (BiPAP)
Typically for noninvasive ventilation
Assist-control or “spontaneous/timed”: all breaths are supported; set rate is a back-up
IPAP is set (targeted with each breath)
PEEP is called EPAP (expiratory positive airway pressure)
If child breathing above the back-up, then all breaths are supported
Is the same thing as a “Pressure Support trial”

## Slide 24
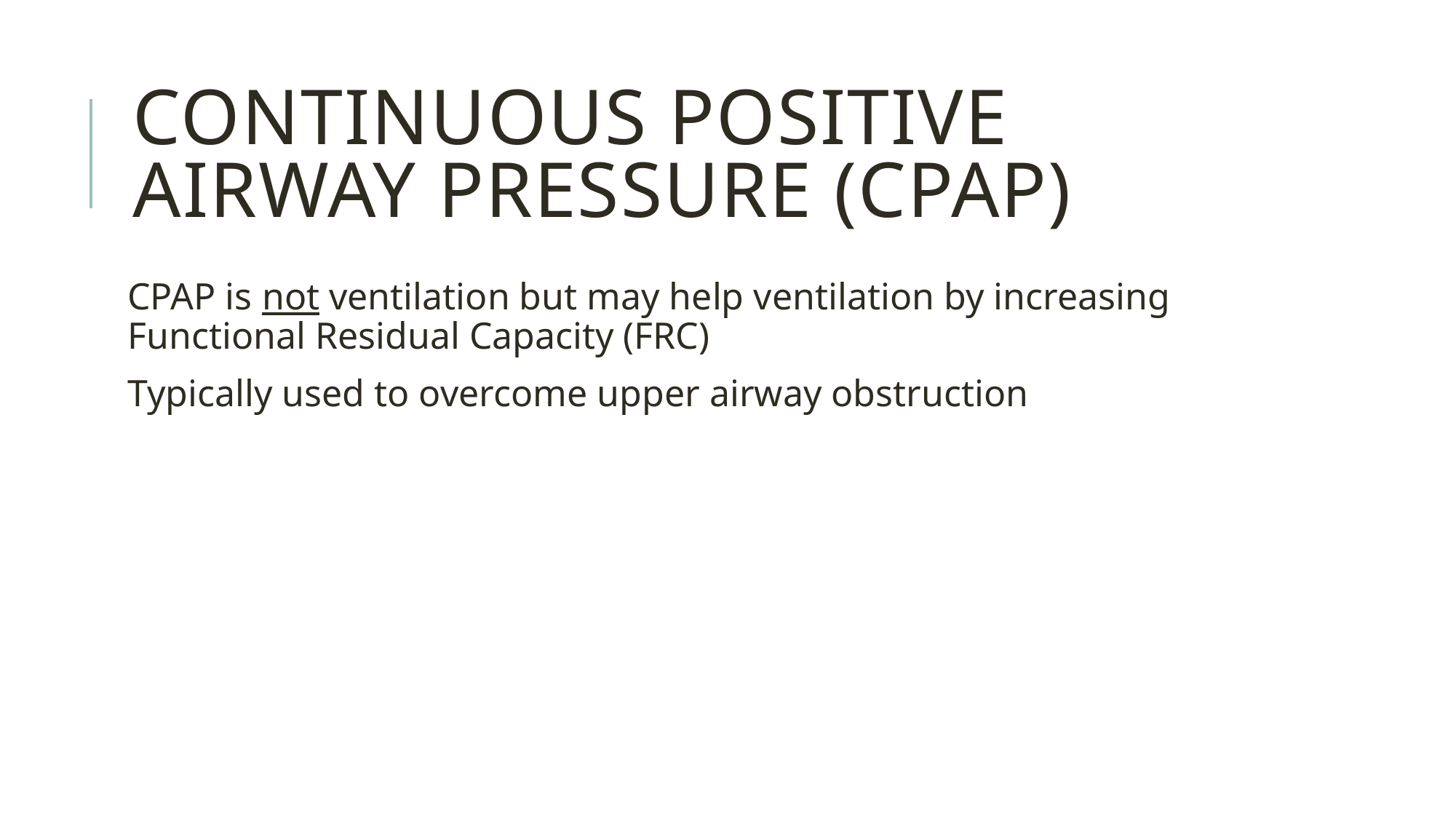

# Continuous Positive Airway Pressure (CPAP)
CPAP is not ventilation but may help ventilation by increasing Functional Residual Capacity (FRC)
Typically used to overcome upper airway obstruction

## Slide 25
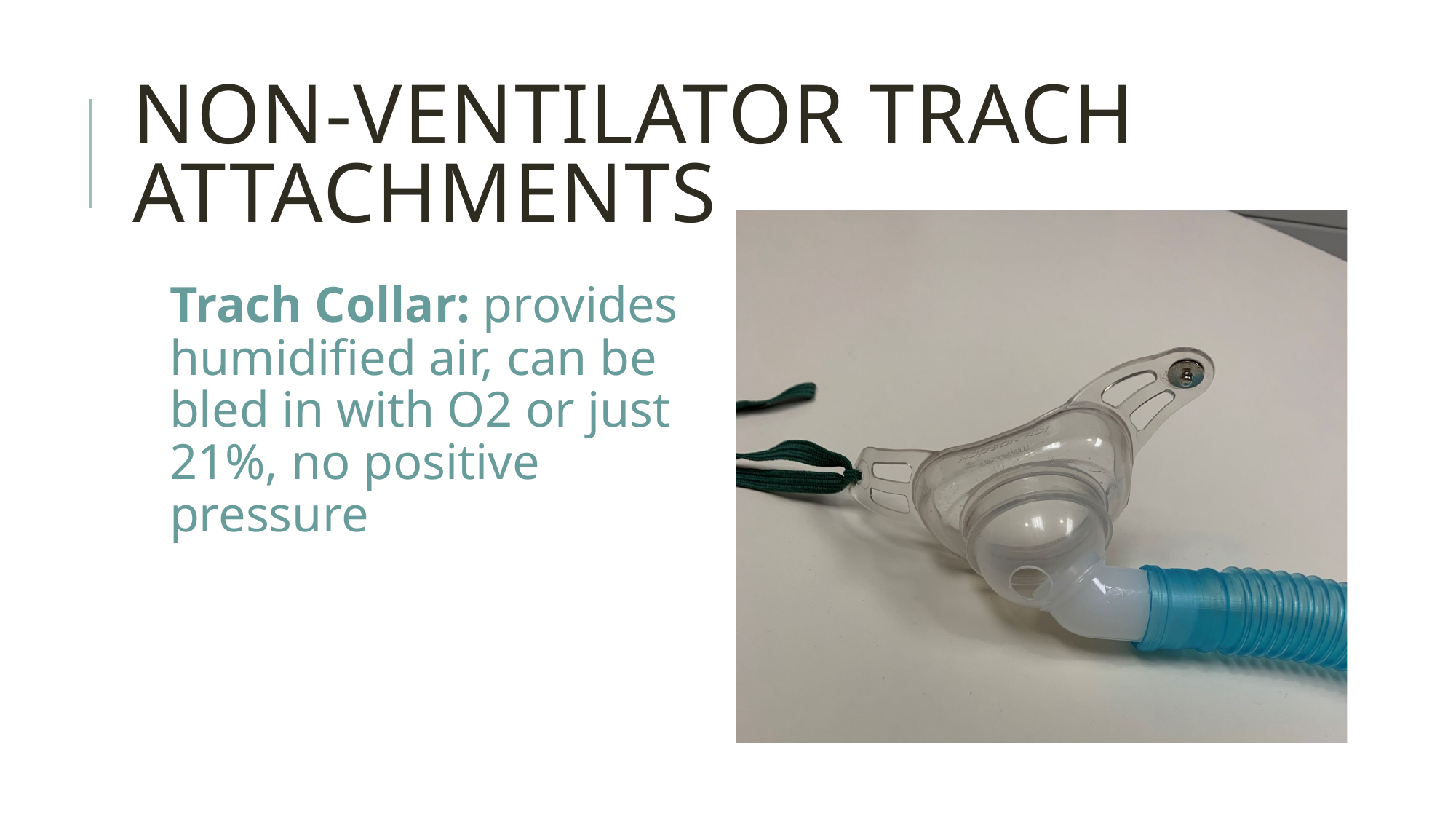

# Non-ventilator trach attachments
Trach Collar: provides humidified air, can be bled in with O2 or just 21%, no positive pressure

## Slide 26
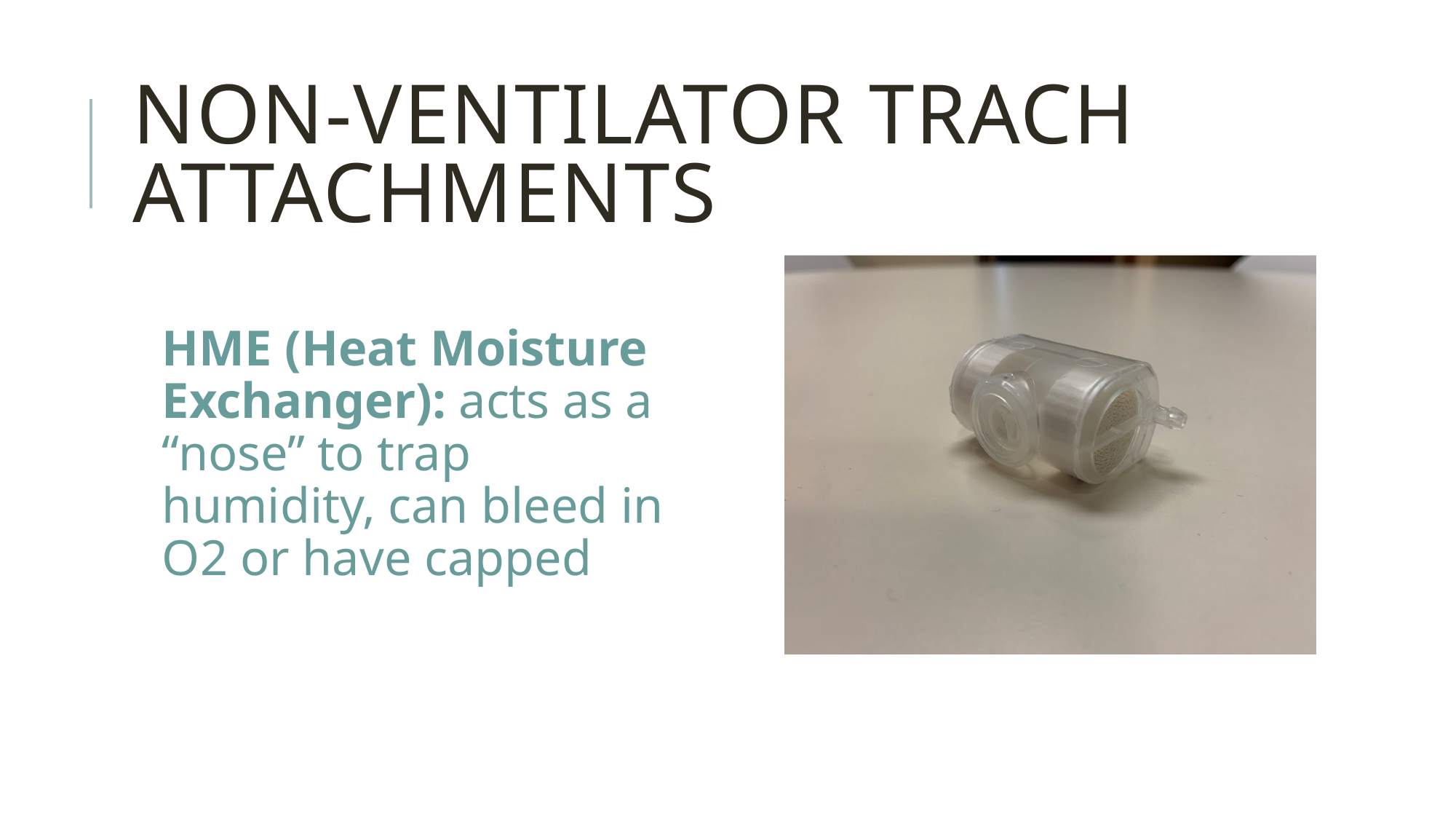

# Non-ventilator trach attachments
HME (Heat Moisture Exchanger): acts as a “nose” to trap humidity, can bleed in O2 or have capped

## Slide 27
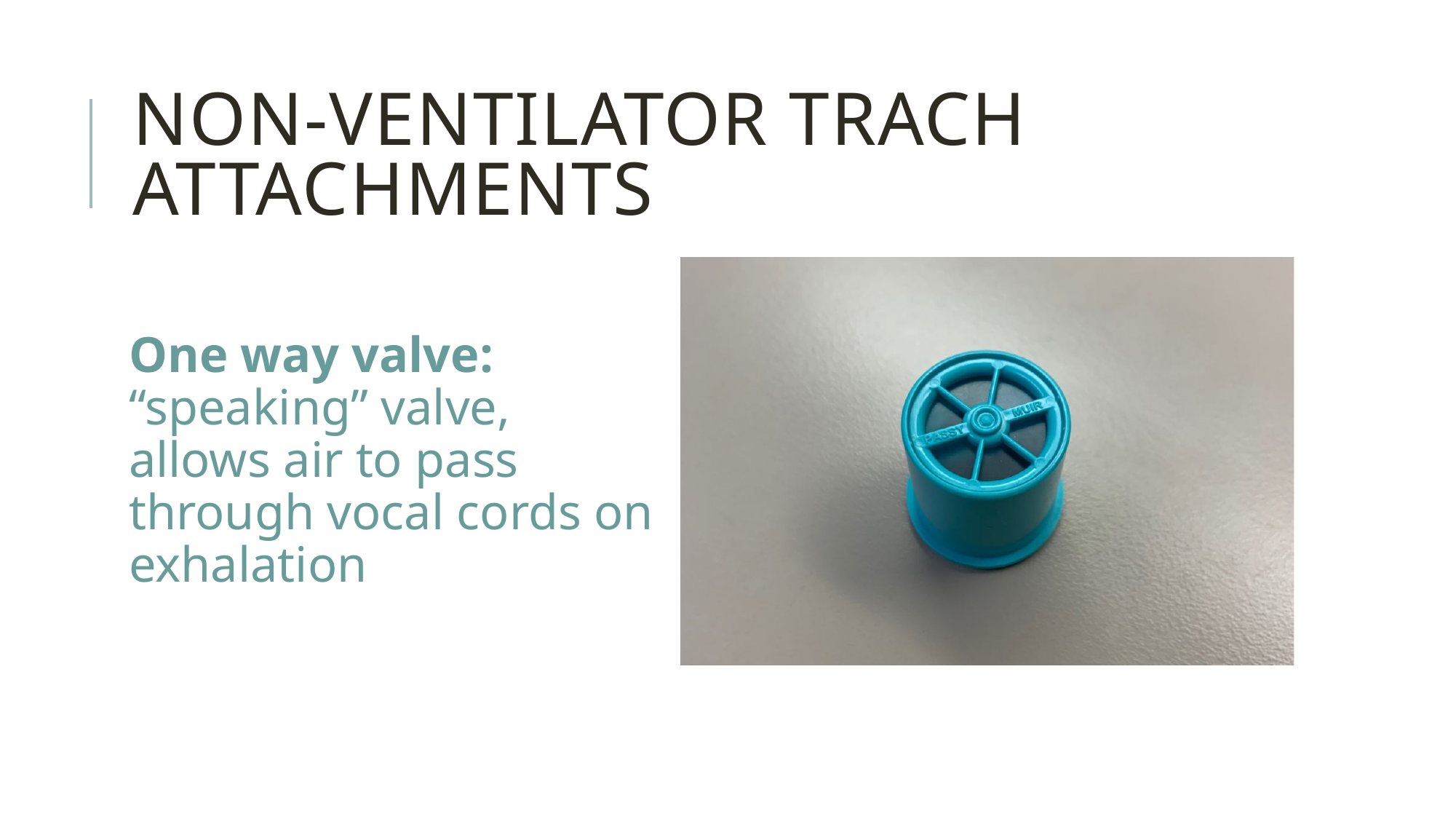

# Non-ventilator trach attachments
One way valve: “speaking” valve, allows air to pass through vocal cords on exhalation

## Slide 28
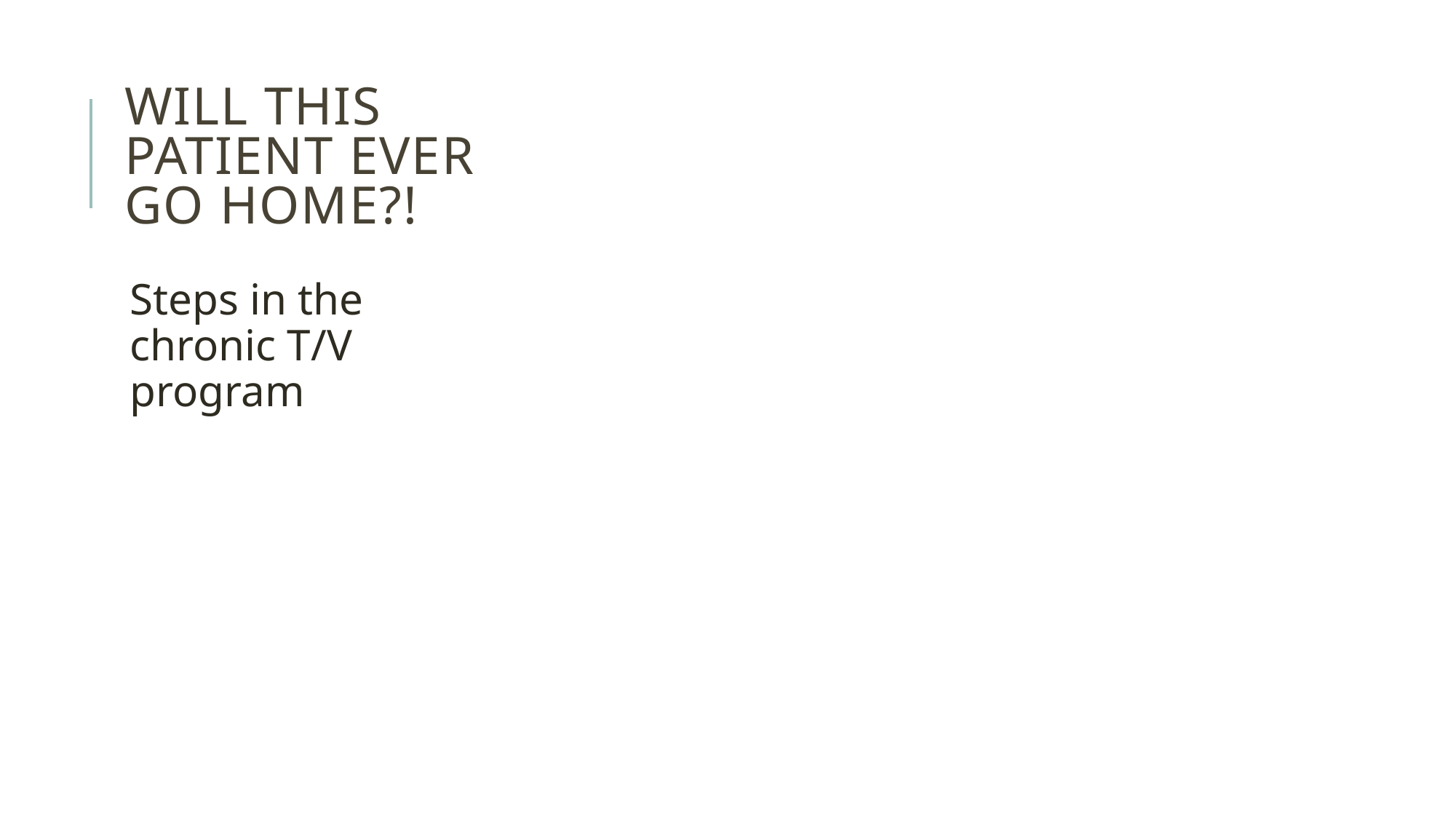

# Will this patient ever go home?!
Steps in the chronic T/V program

## Slide 29
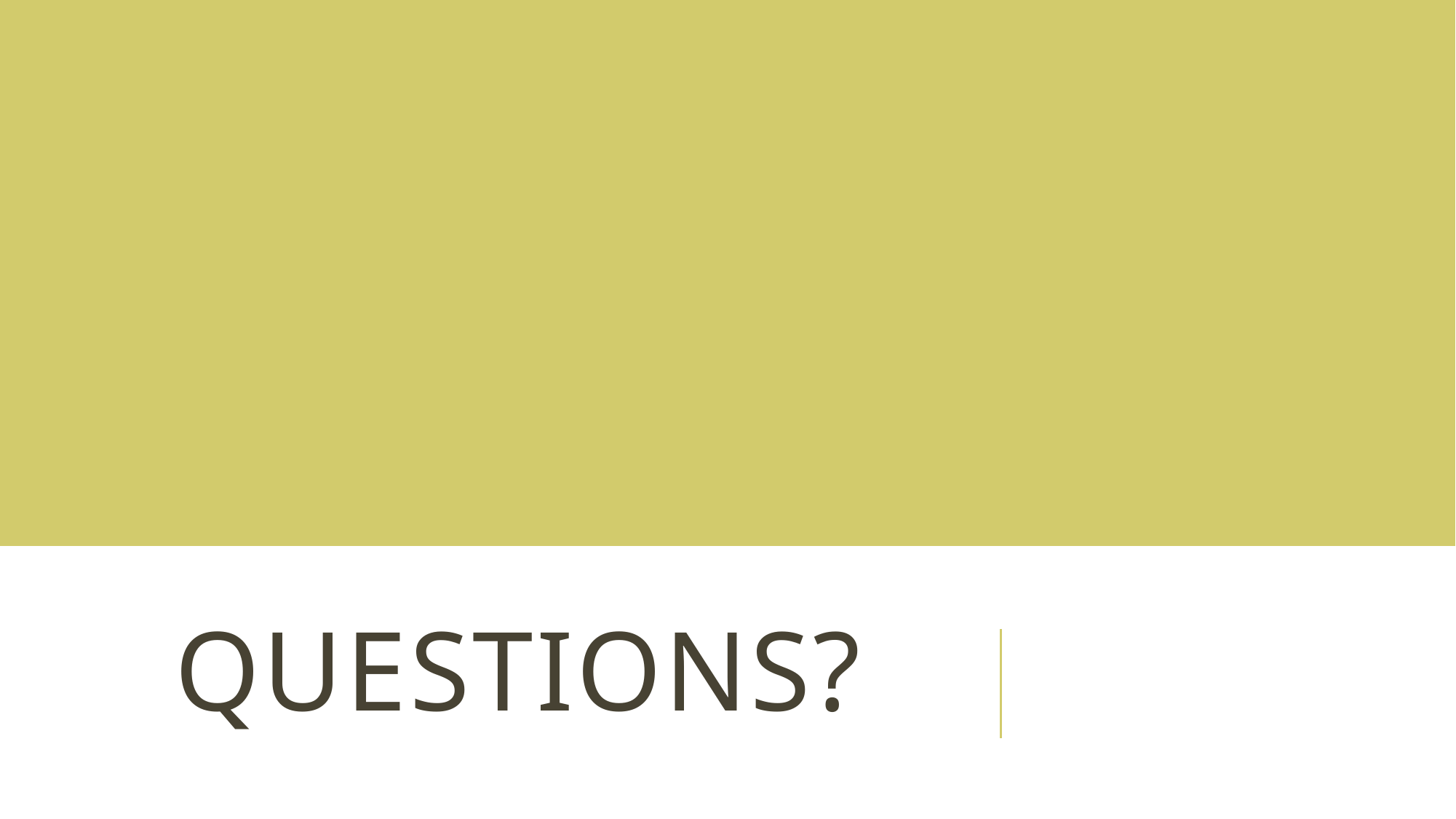

# Questions?
