# Supplementary material for: Pediatric Resident Education in Pulmonary (PREP): A Subspecialty Preparatory Boot Camp Curriculum for Pediatric Residents
Source: MedEdPORTAL. 2021 Jan 7;17:11066. doi: 10.15766/mep_2374-8265.11066 (PMC7809931; doi:10.15766/mep_2374-8265.11066)
Supplement: Supplementary file 1 — Example Agenda.docxOrientation Template.pptxIntroduction to Tracheostomies and Ventilators.pptxCystic Fibrosis JeoPARODY.pptxIntroduction to Airway Clearance and Lung Expansion.pptxInstructor Guide CPT.docxInstructor Guide IS.docxInstructor Guide PEP.docxInstructor Guide PAP.docxInstructor Guide OPEP.docxInstructor Guide Insufflator Exsufflator.docxInstructor Guide HFCWO.docxInstructor Guide IPV.docxPREP Day of Evaluation.docxPREP End of Rotation Evaluation.docxPREP Faculty Feedback Survey.docxPREP Focus Group Guide.docx [file mep_2374-8265.11066-s001.zip › D. Cystic Fibrosis JeoPARODY.pptx]

## Slide 1
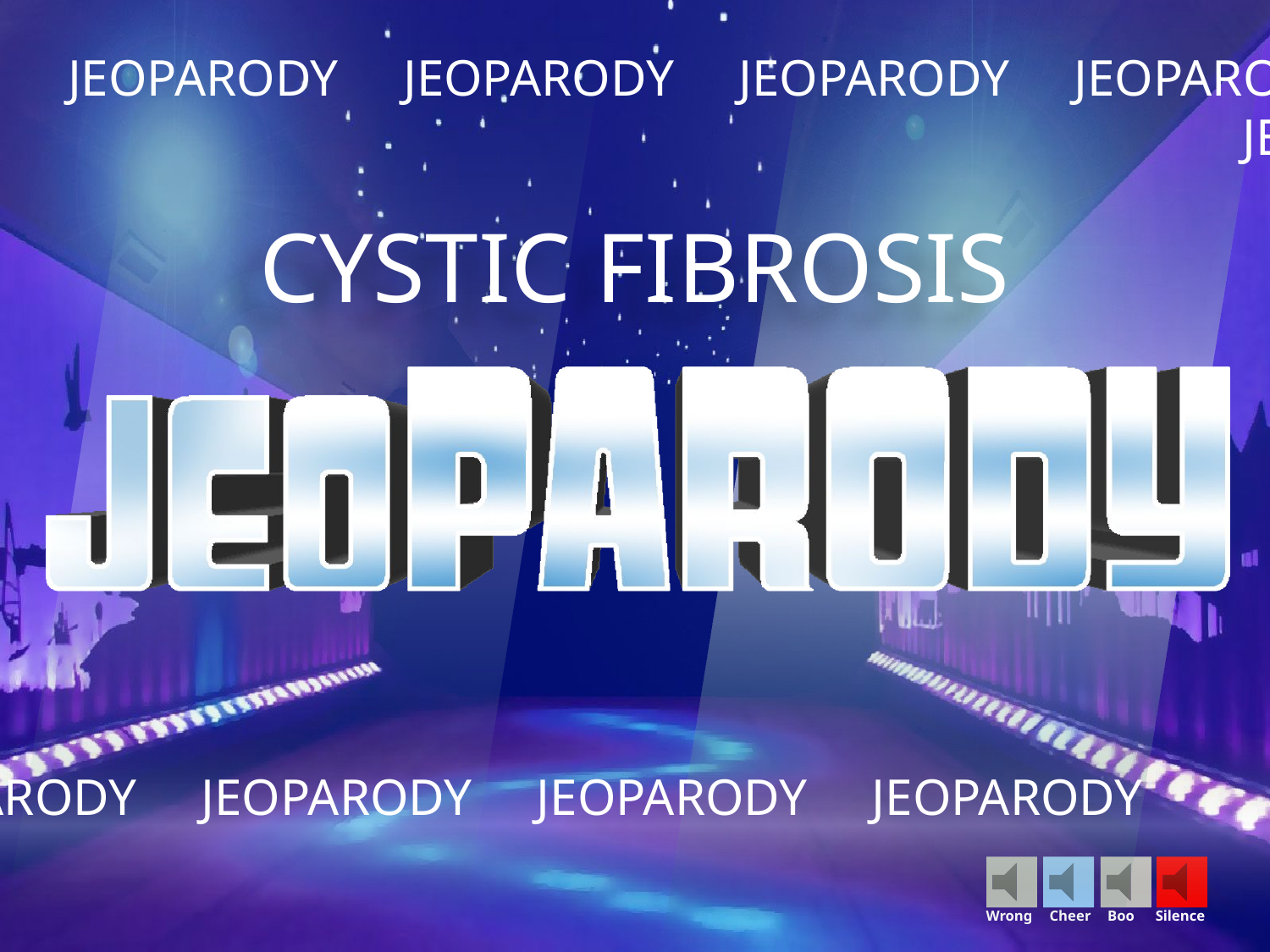

JEOPARODY JEOPARODY JEOPARODY JEOPARODY JEOPARODY JEOPARODY JEOPARODY JEOPARODY JEOPARODY JEOPARODY JEOPARODY JEOPARODY JEOPARODY JEOPARODY JEOPARODY JEOPARODY JEOPARODY JEOPARODY JEOPARODY
CYSTIC FIBROSIS
JEOPARODY JEOPARODY JEOPARODY JEOPARODY JEOPARODY JEOPARODY JEOPARODY JEOPARODY JEOPARODY JEOPARODY JEOPARODY JEOPARODY JEOPARODY JEOPARODY JEOPARODY JEOPARODY JEOPARODY JEOPARODY JEOPARODY

## Slide 2
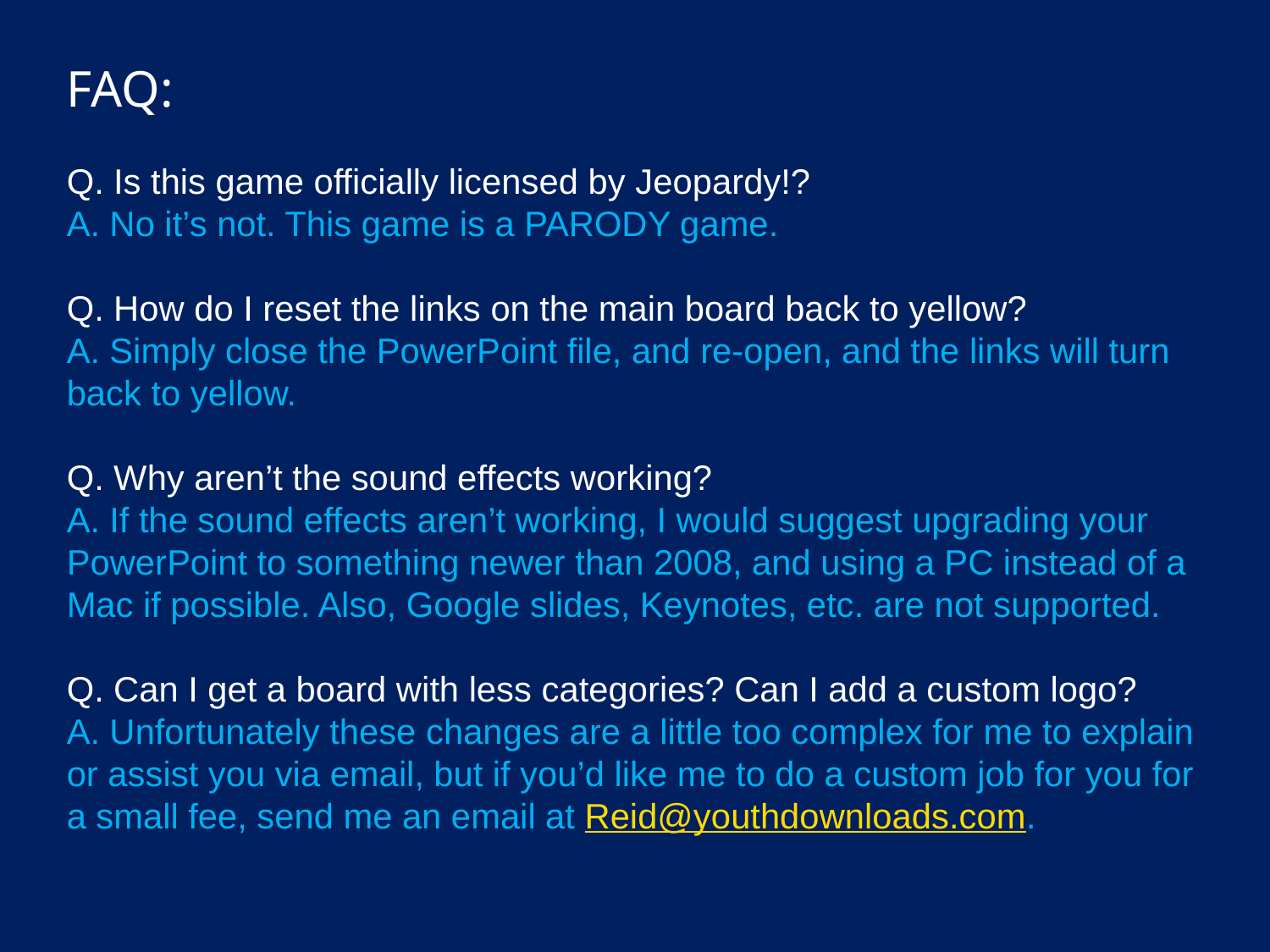

FAQ:
Q. Is this game officially licensed by Jeopardy!?
A. No it’s not. This game is a PARODY game.
Q. How do I reset the links on the main board back to yellow?
A. Simply close the PowerPoint file, and re-open, and the links will turn back to yellow.
Q. Why aren’t the sound effects working?
A. If the sound effects aren’t working, I would suggest upgrading your PowerPoint to something newer than 2008, and using a PC instead of a Mac if possible. Also, Google slides, Keynotes, etc. are not supported.
Q. Can I get a board with less categories? Can I add a custom logo?
A. Unfortunately these changes are a little too complex for me to explain or assist you via email, but if you’d like me to do a custom job for you for a small fee, send me an email at Reid@youthdownloads.com.

## Slide 3
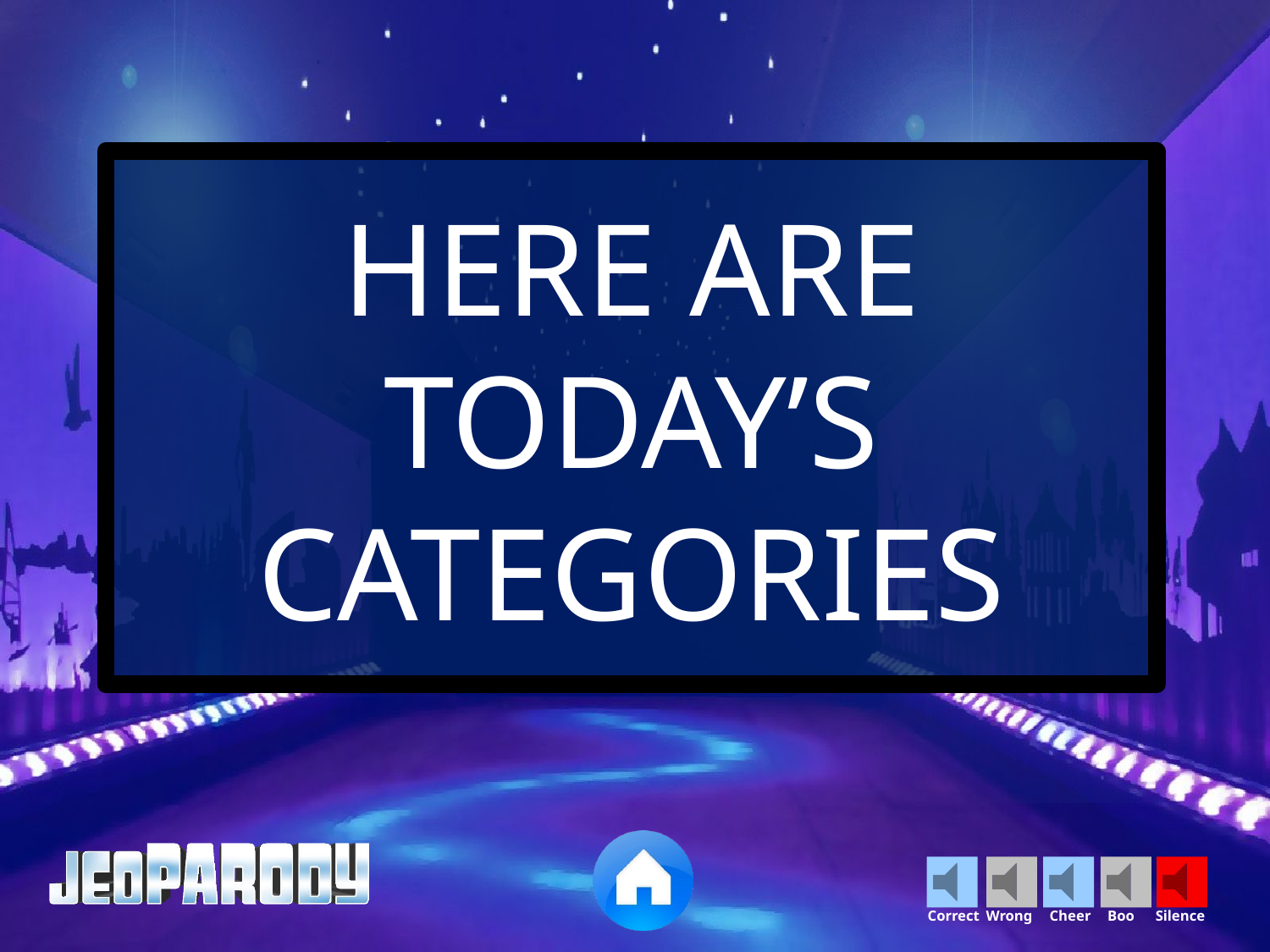

HERE ARE TODAY’S CATEGORIES

## Slide 4
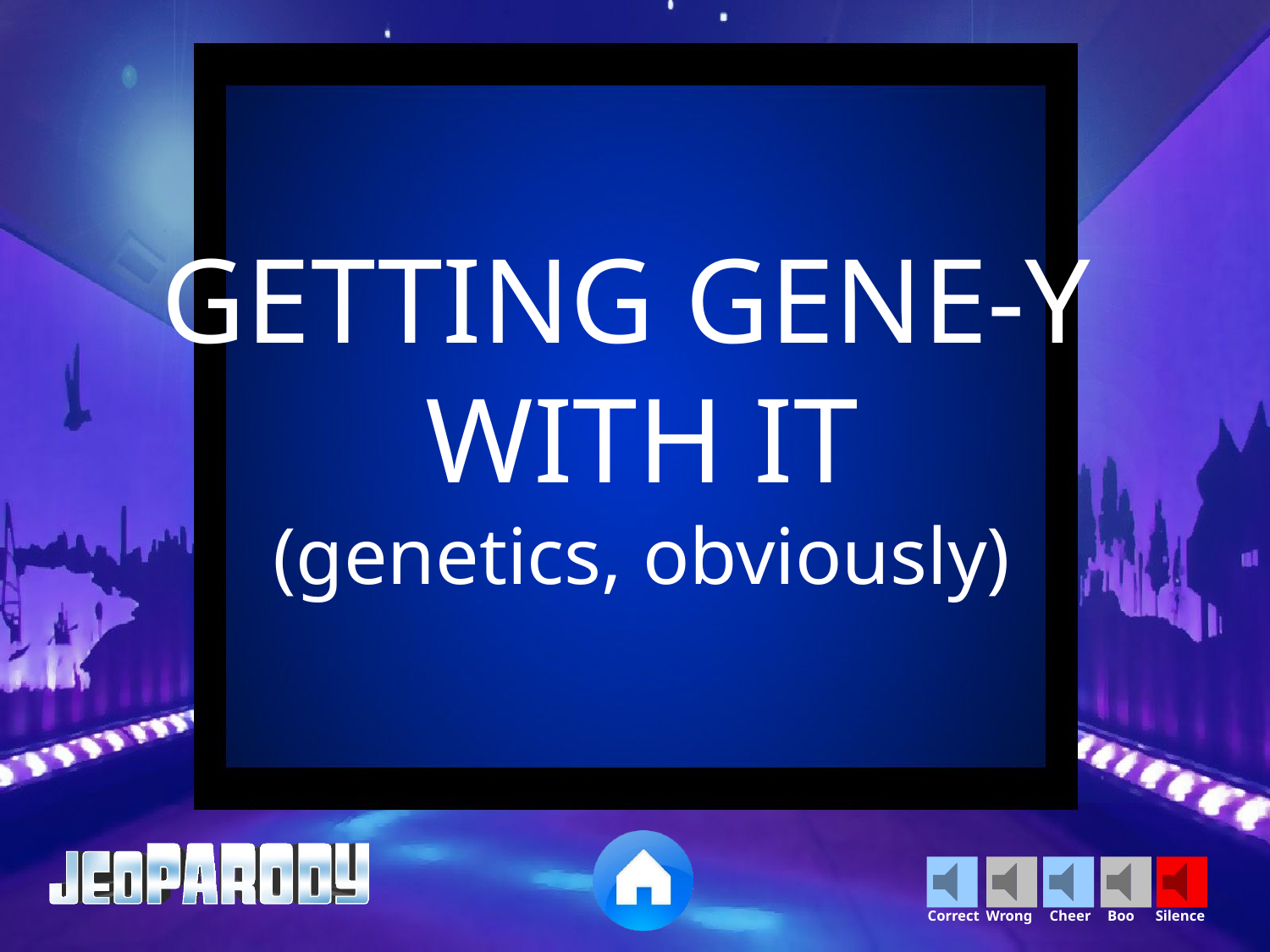

GETTING GENE-Y
WITH IT
(genetics, obviously)

## Slide 5
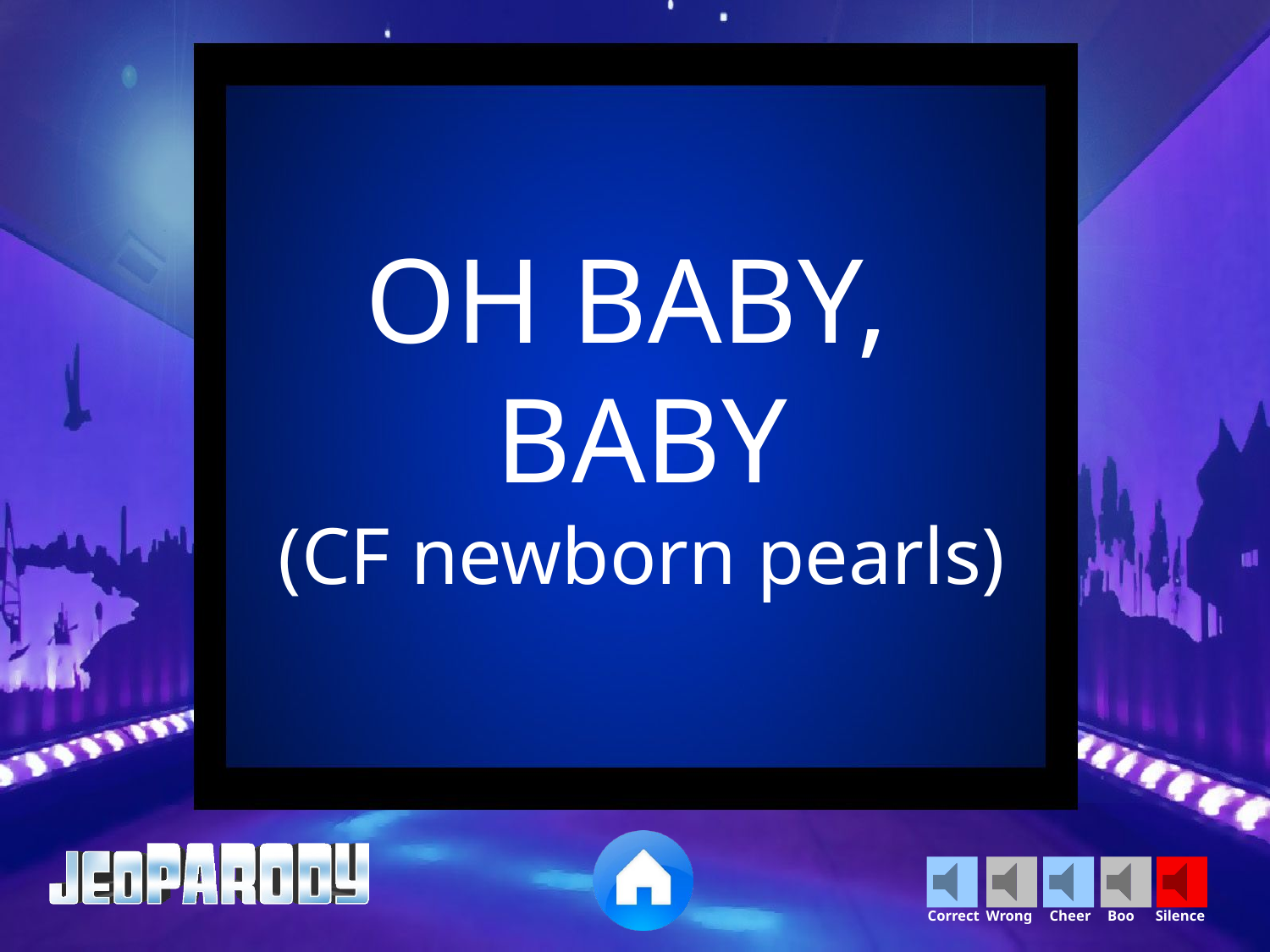

OH BABY,
BABY
(CF newborn pearls)

## Slide 6
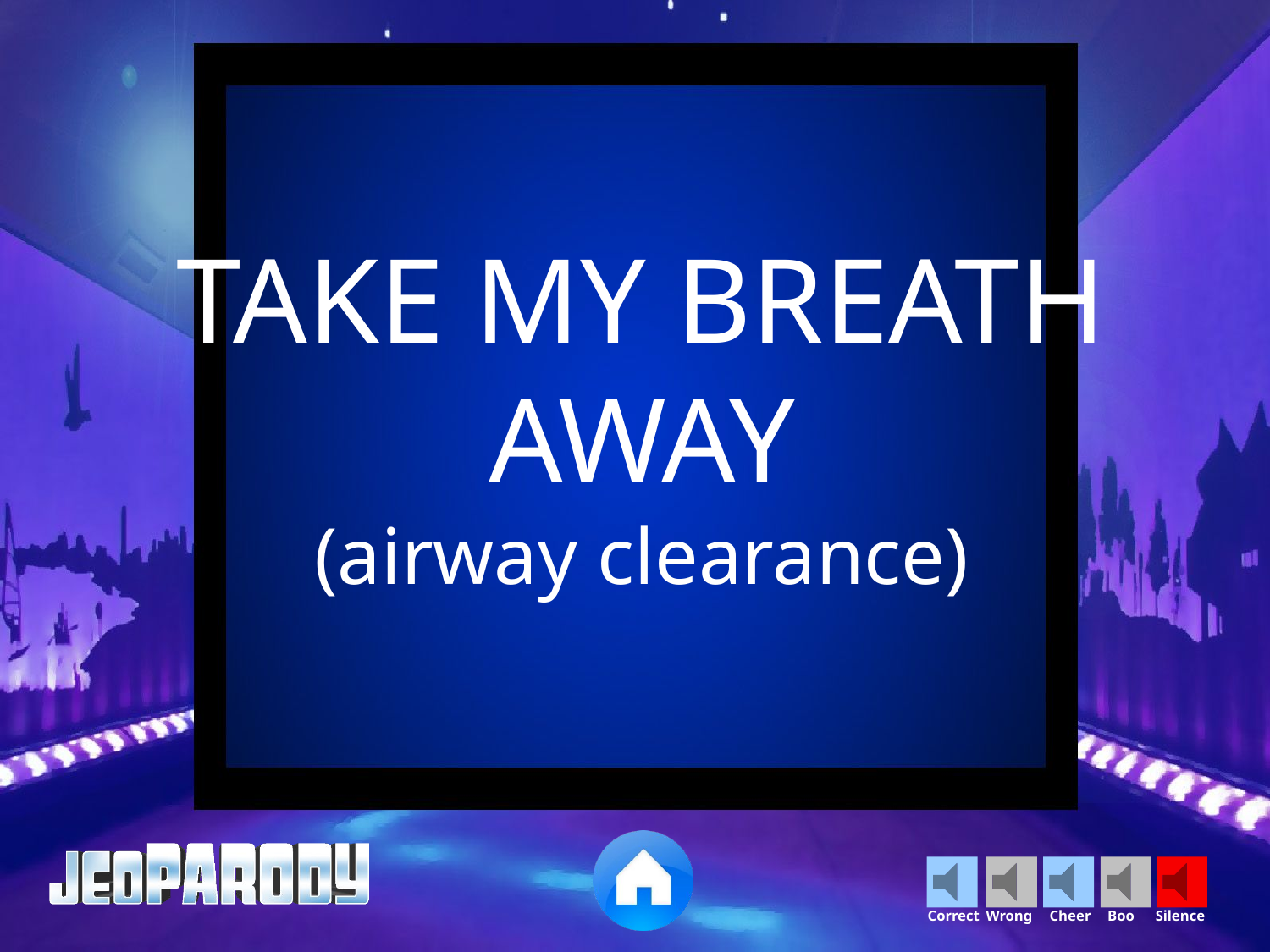

TAKE MY BREATH AWAY
(airway clearance)

## Slide 7
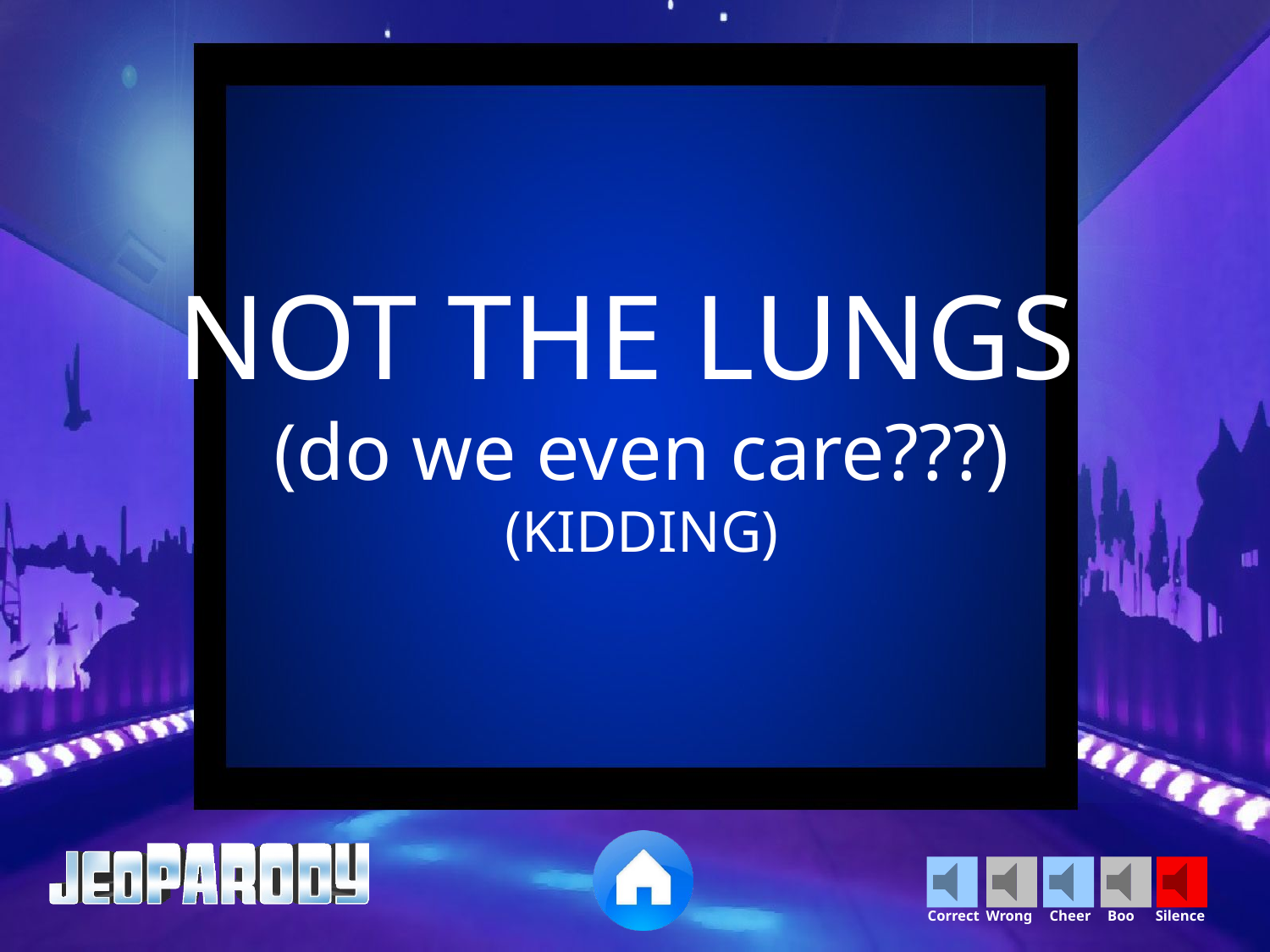

NOT THE LUNGS
(do we even care???)
(KIDDING)

## Slide 8
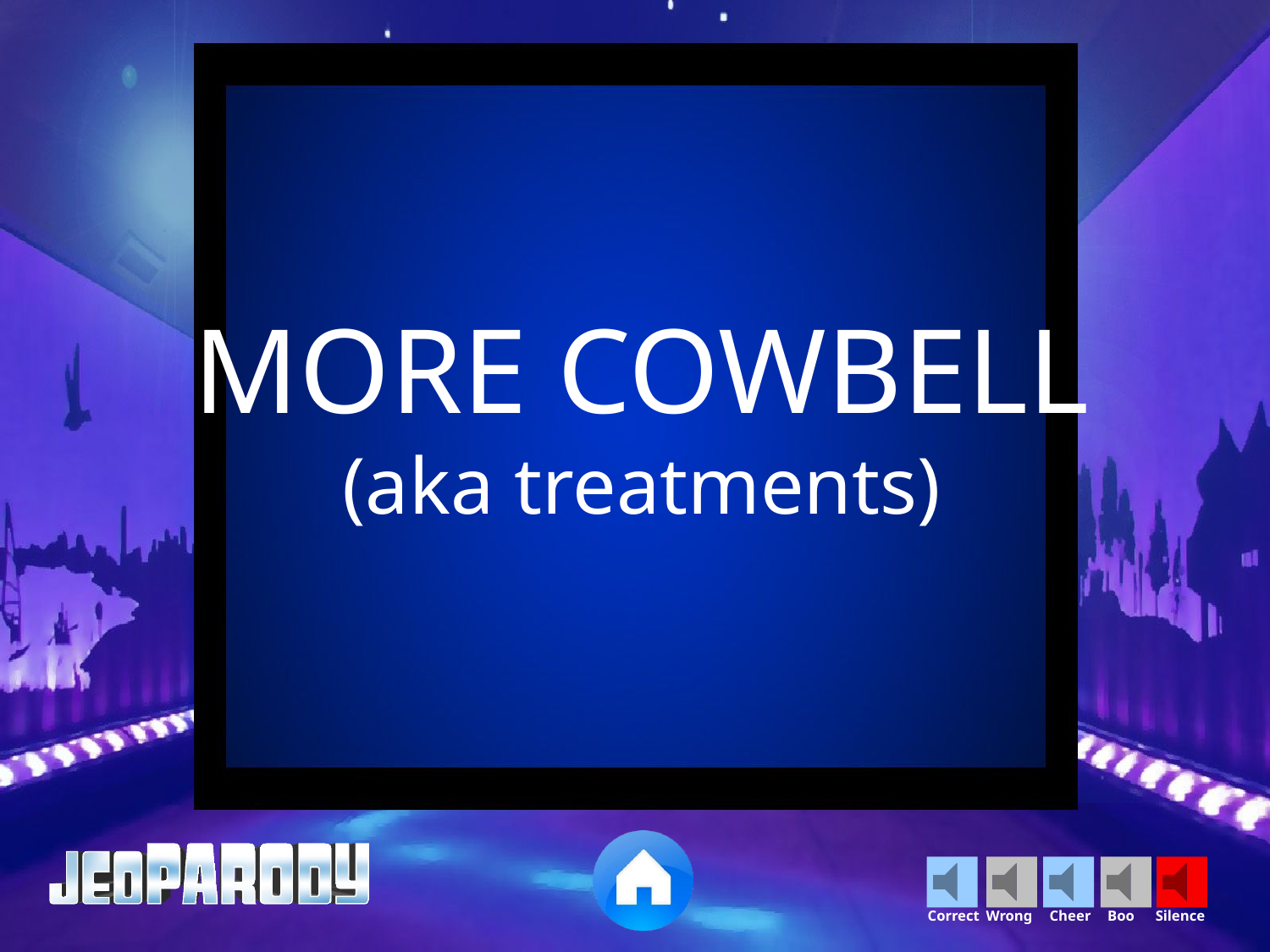

MORE COWBELL
(aka treatments)

## Slide 9
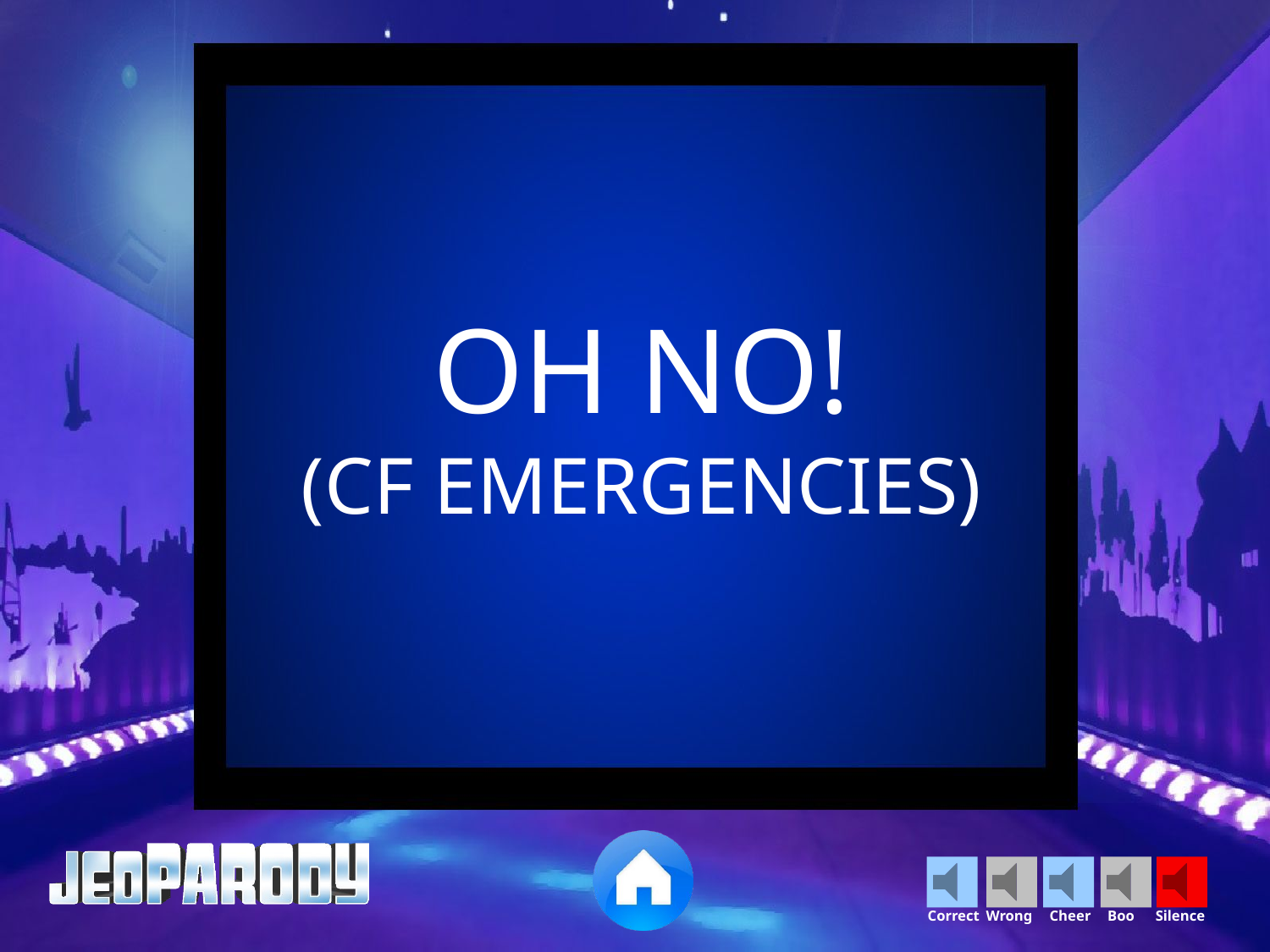

OH NO!
(CF EMERGENCIES)

## Slide 10
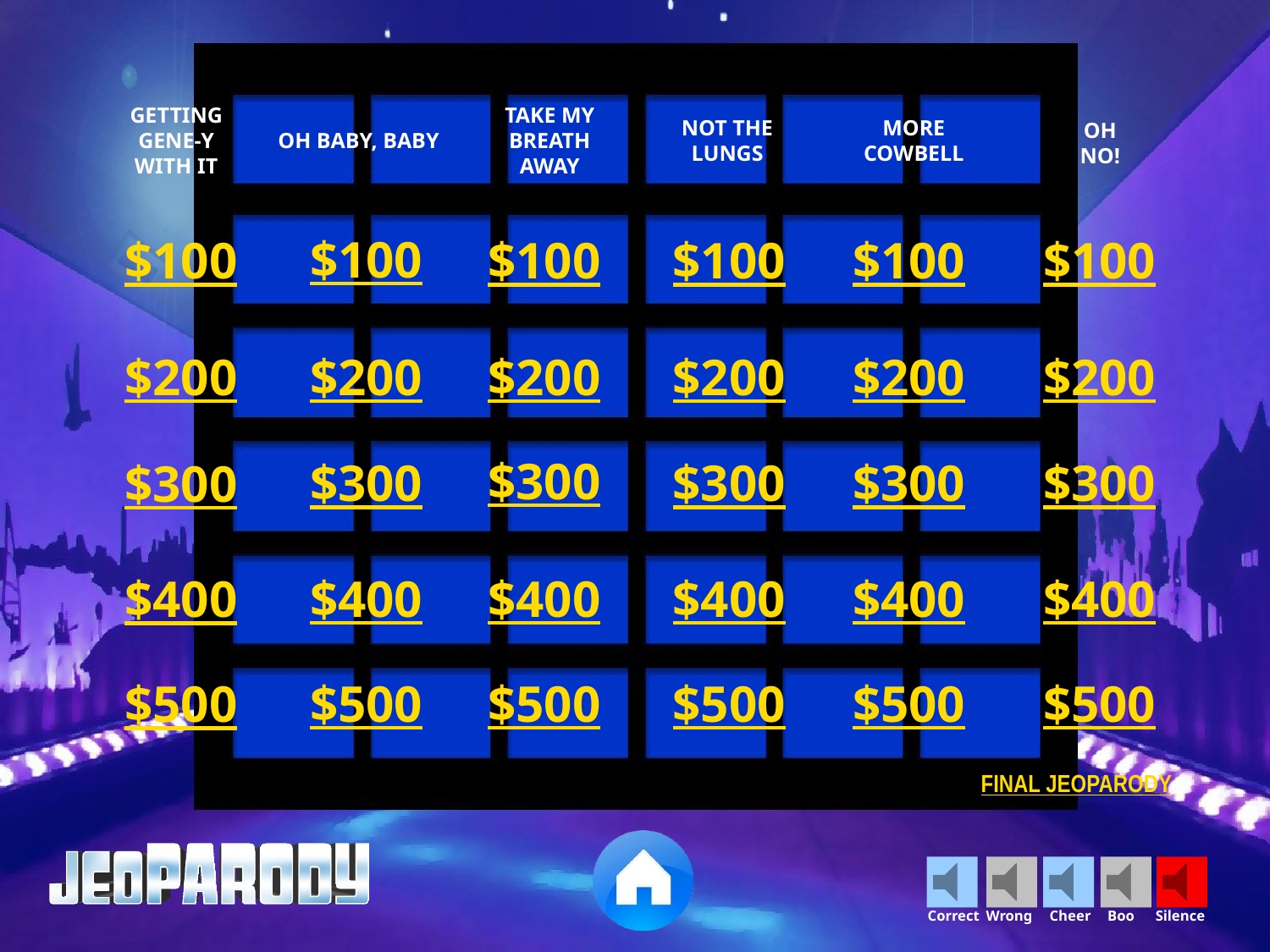

TAKE MY BREATH AWAY
GETTING GENE-Y WITH IT
OH BABY, BABY
NOT THE LUNGS
MORE COWBELL
OH
NO!
$100
$100
$100
$100
$100
$100
$200
$200
$200
$200
$200
$200
$300
$300
$300
$300
$300
$300
$400
$400
$400
$400
$400
$400
$500
$500
$500
$500
$500
$500
FINAL JEOPARODY

## Slide 11
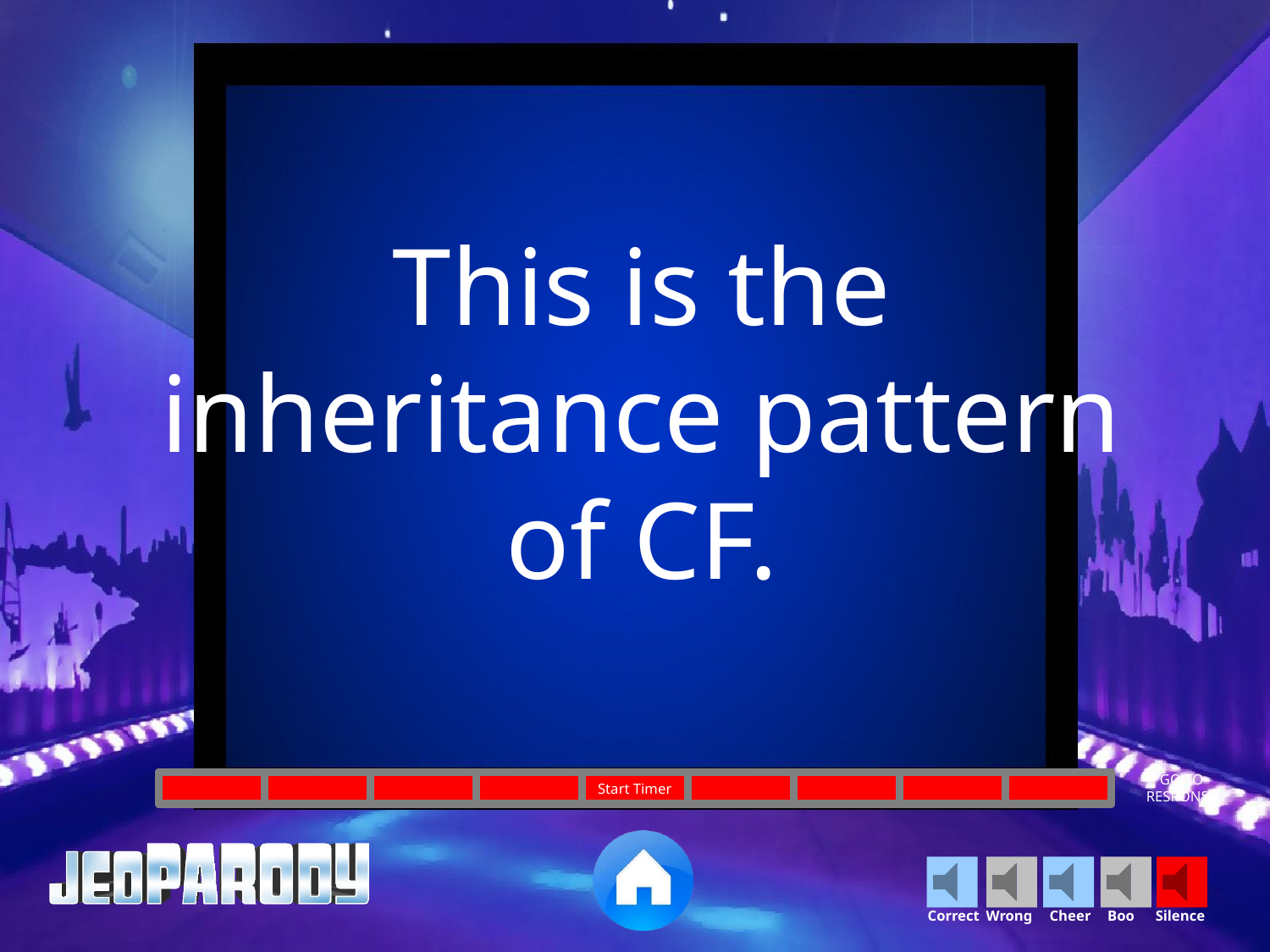

This is the inheritance pattern of CF.

## Slide 12
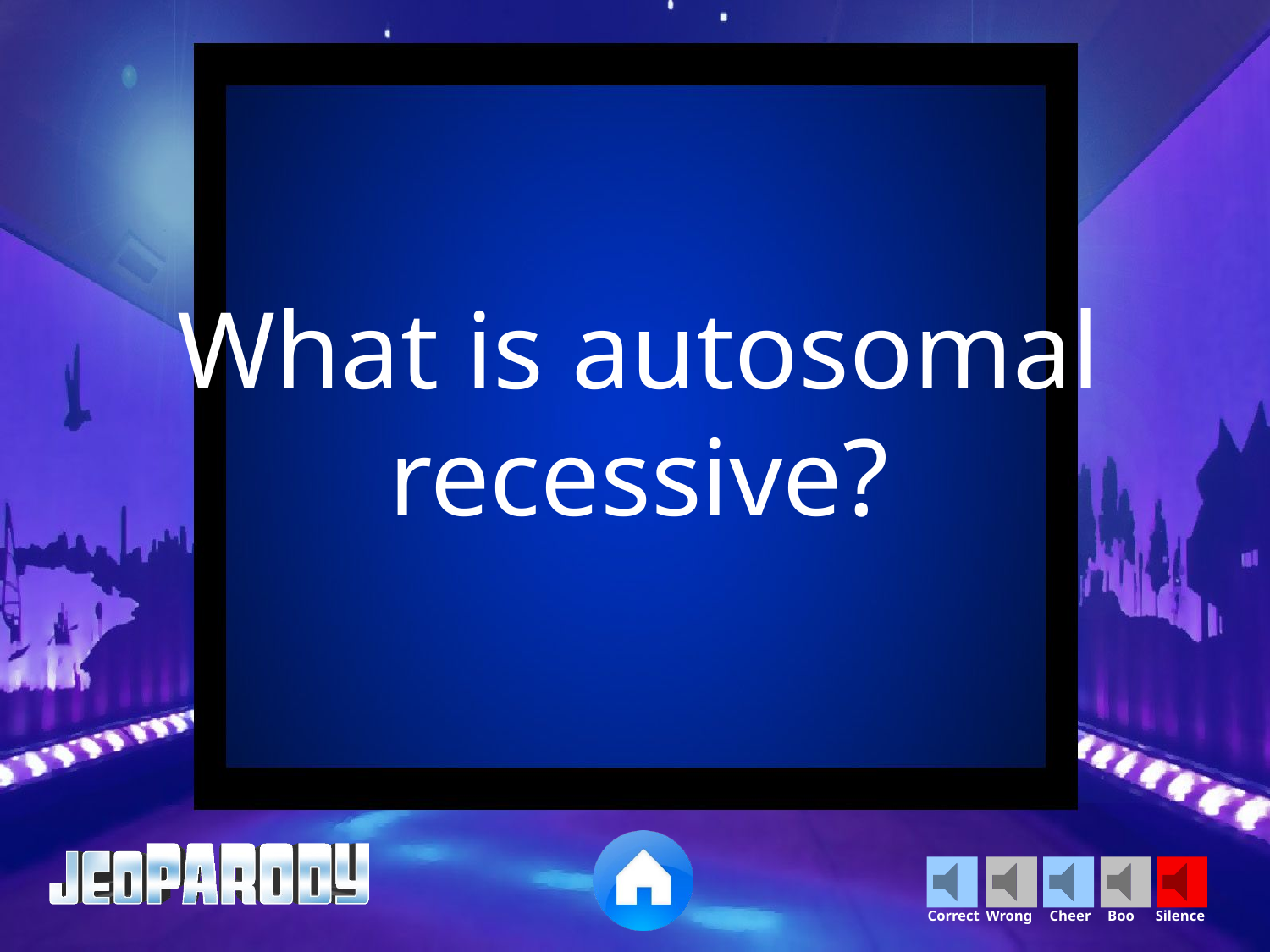

What is autosomal recessive?

## Slide 13
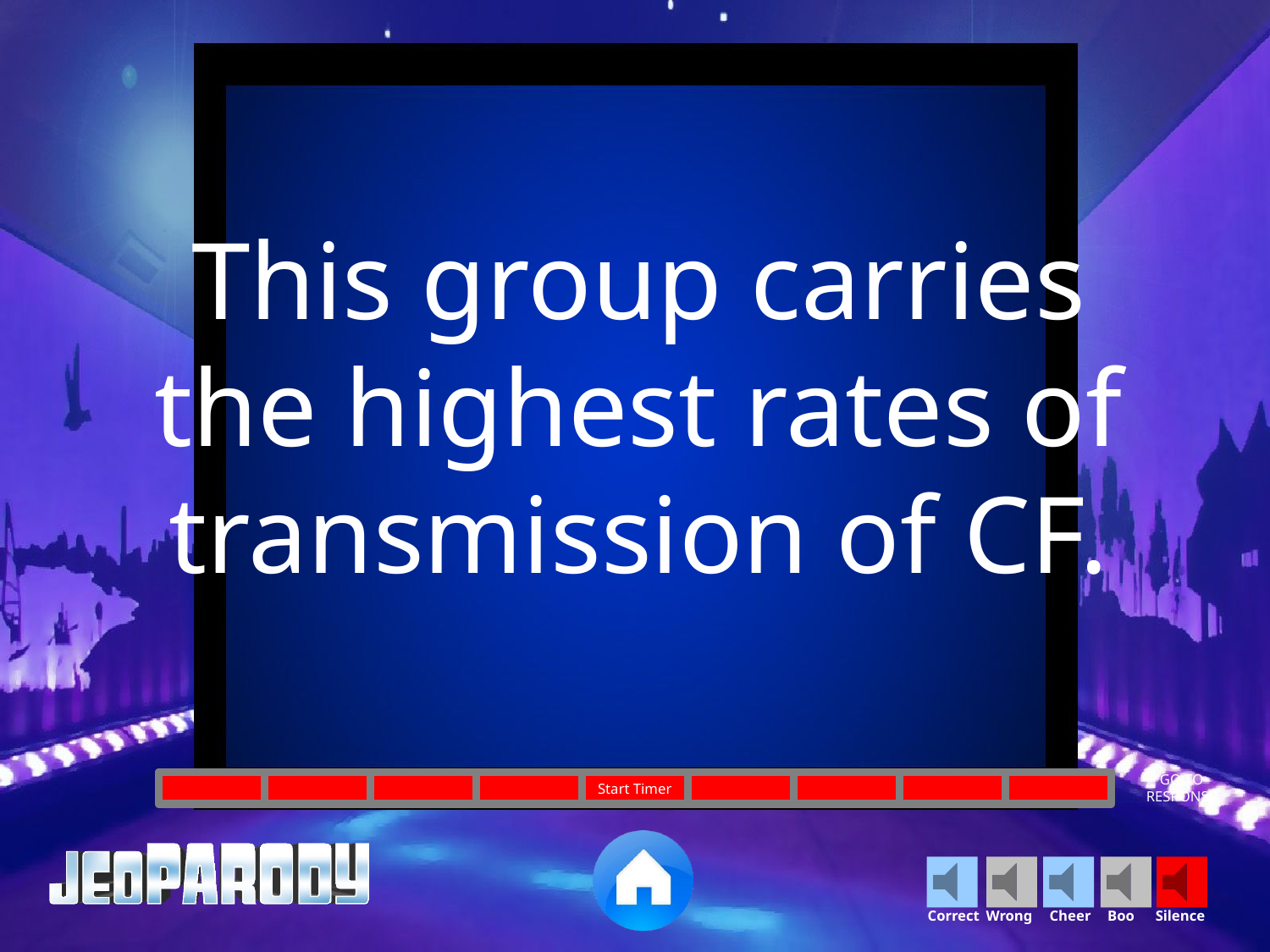

This group carries the highest rates of transmission of CF.

## Slide 14
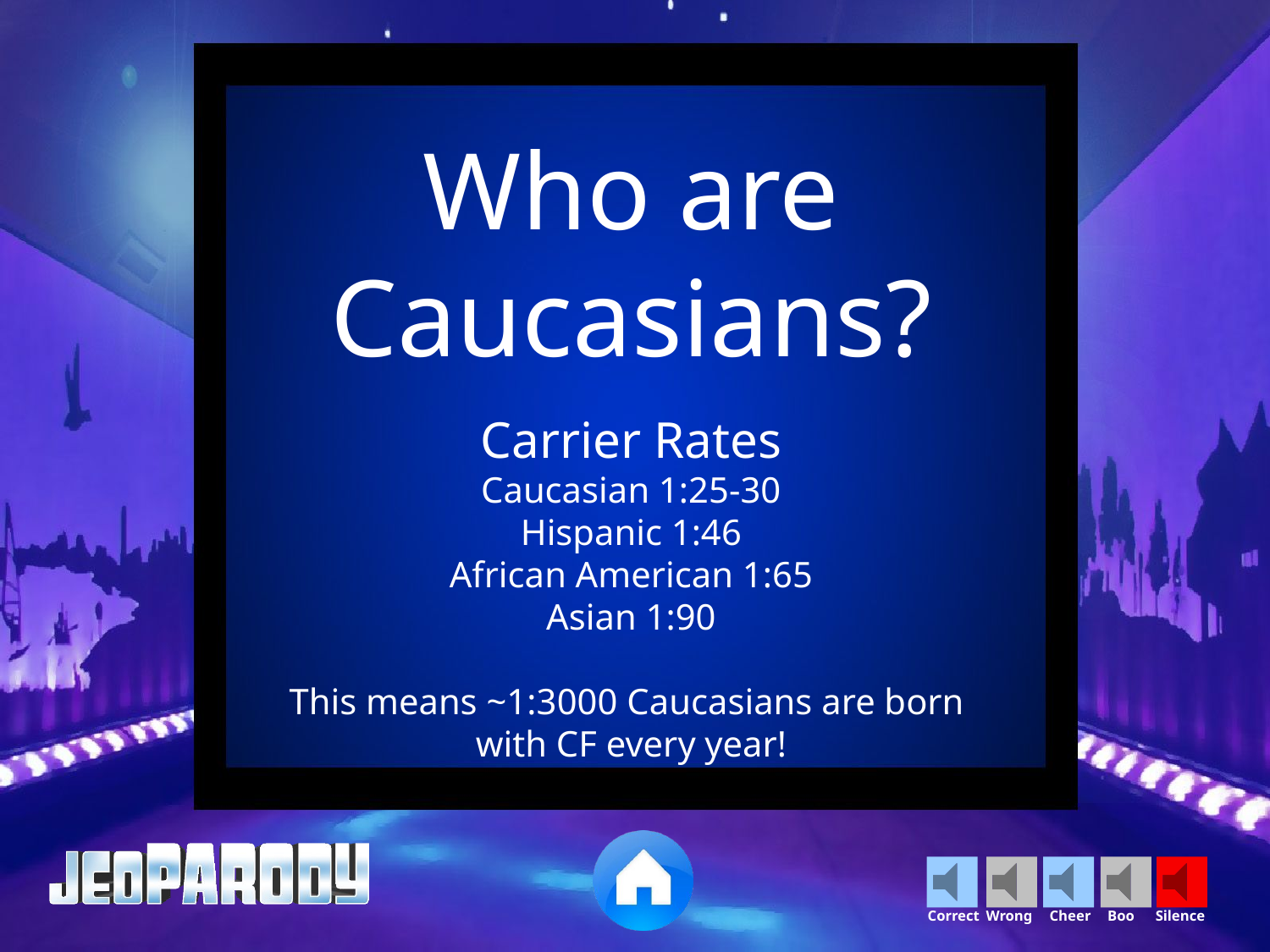

Who are Caucasians?
Carrier Rates
Caucasian 1:25-30
Hispanic 1:46
African American 1:65
Asian 1:90
This means ~1:3000 Caucasians are born
with CF every year!

## Slide 15
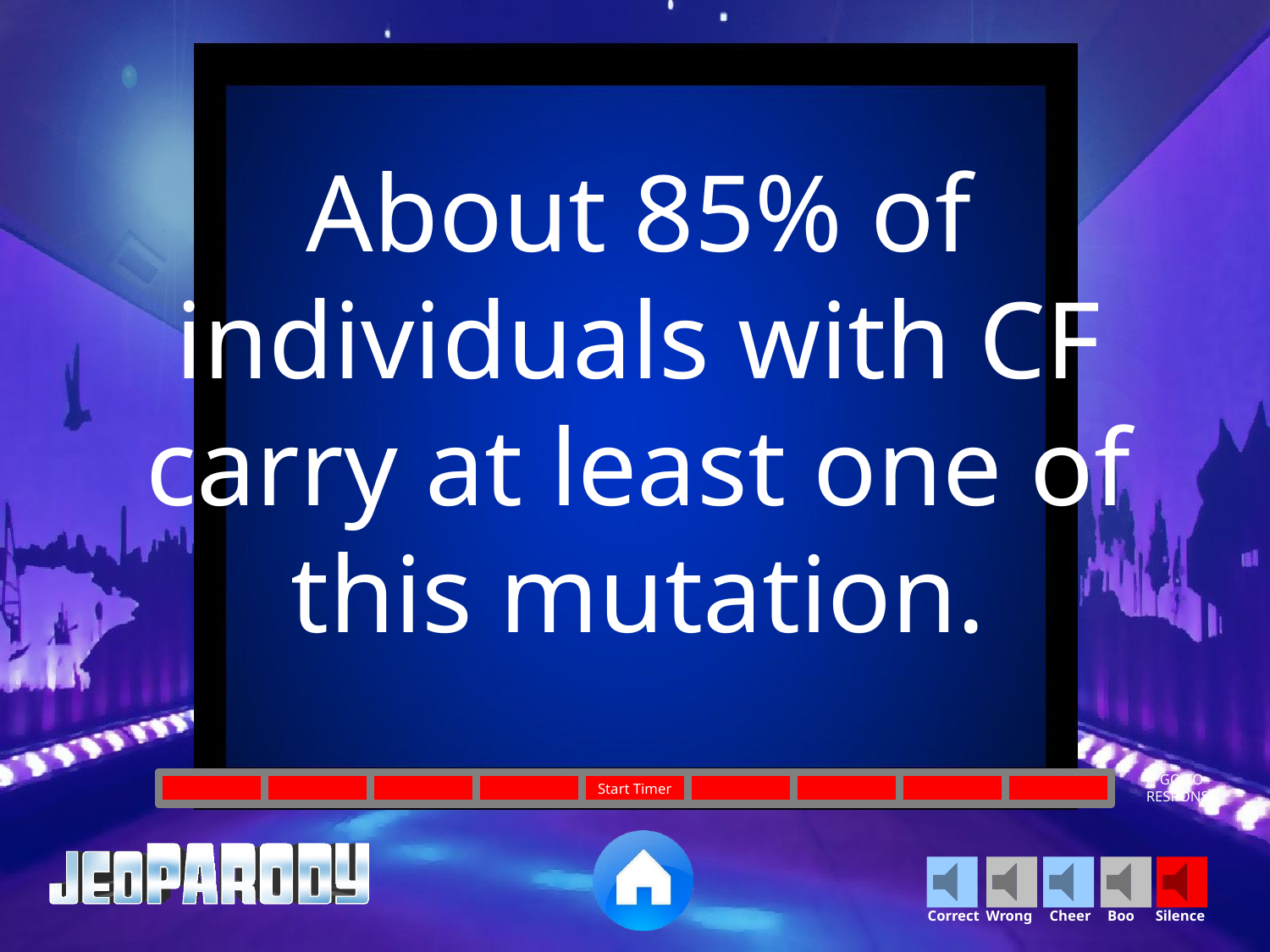

About 85% of individuals with CF carry at least one of this mutation.

## Slide 16
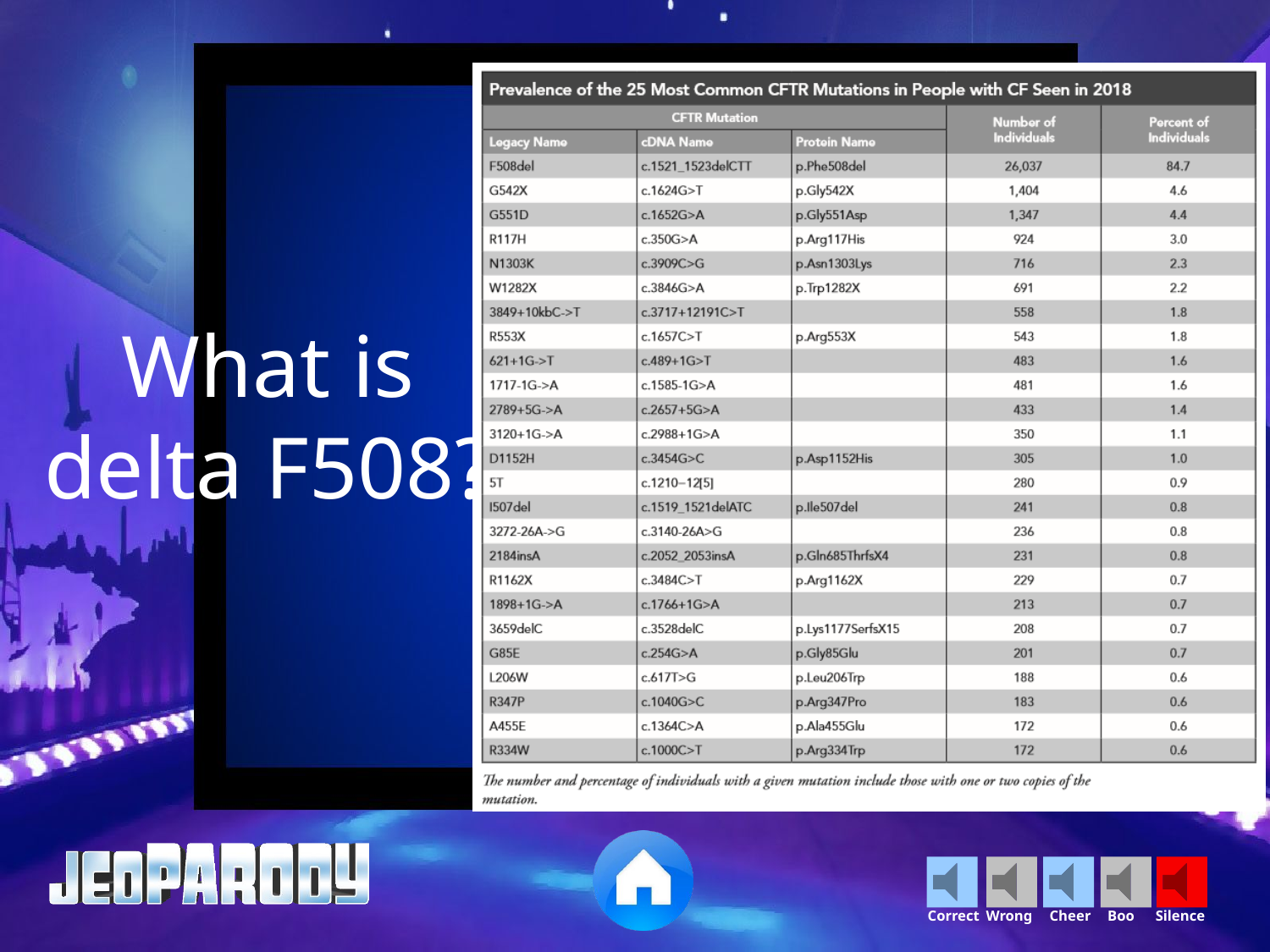

What is delta F508?

## Slide 17
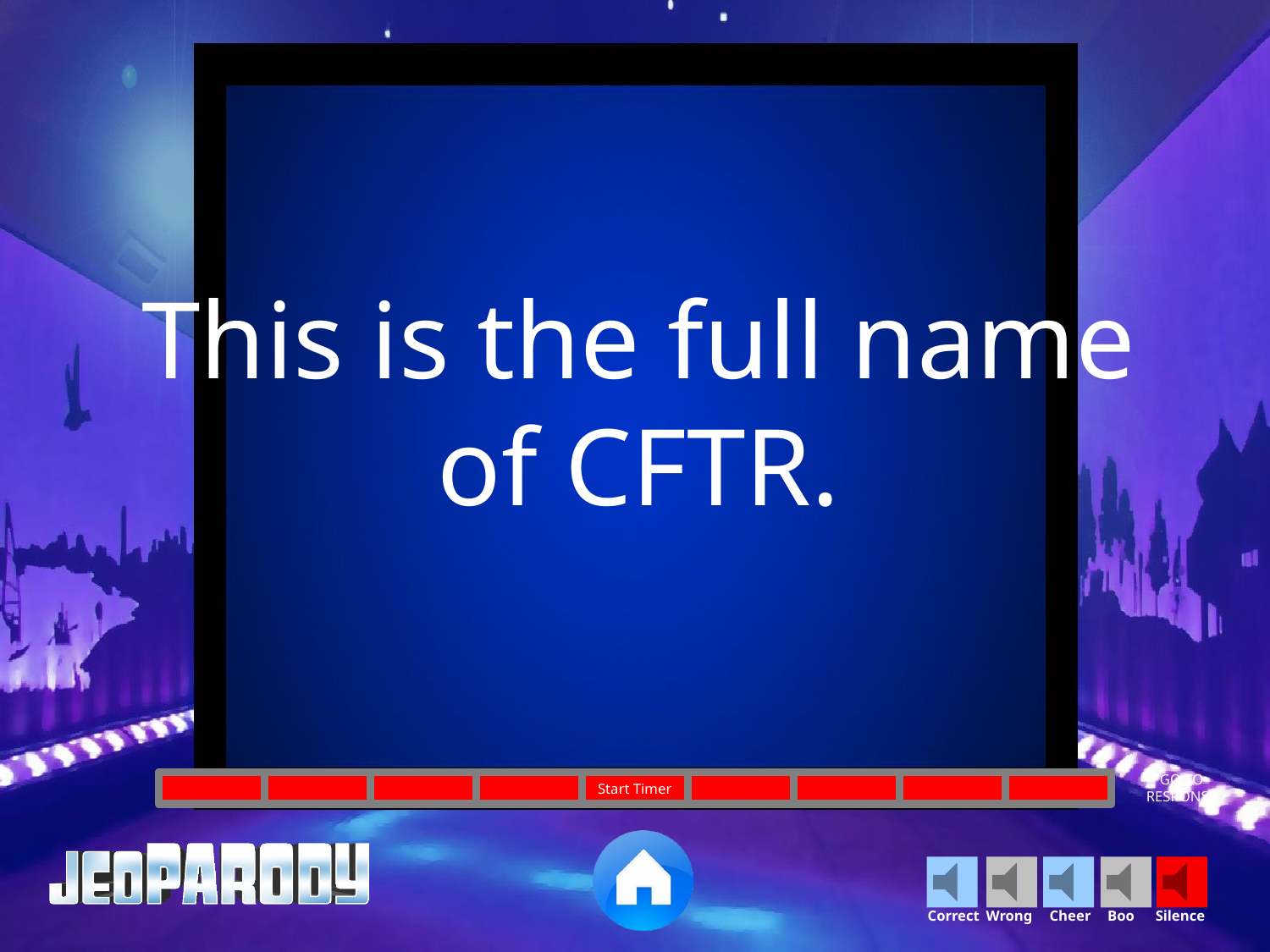

This is the full name of CFTR.

## Slide 18
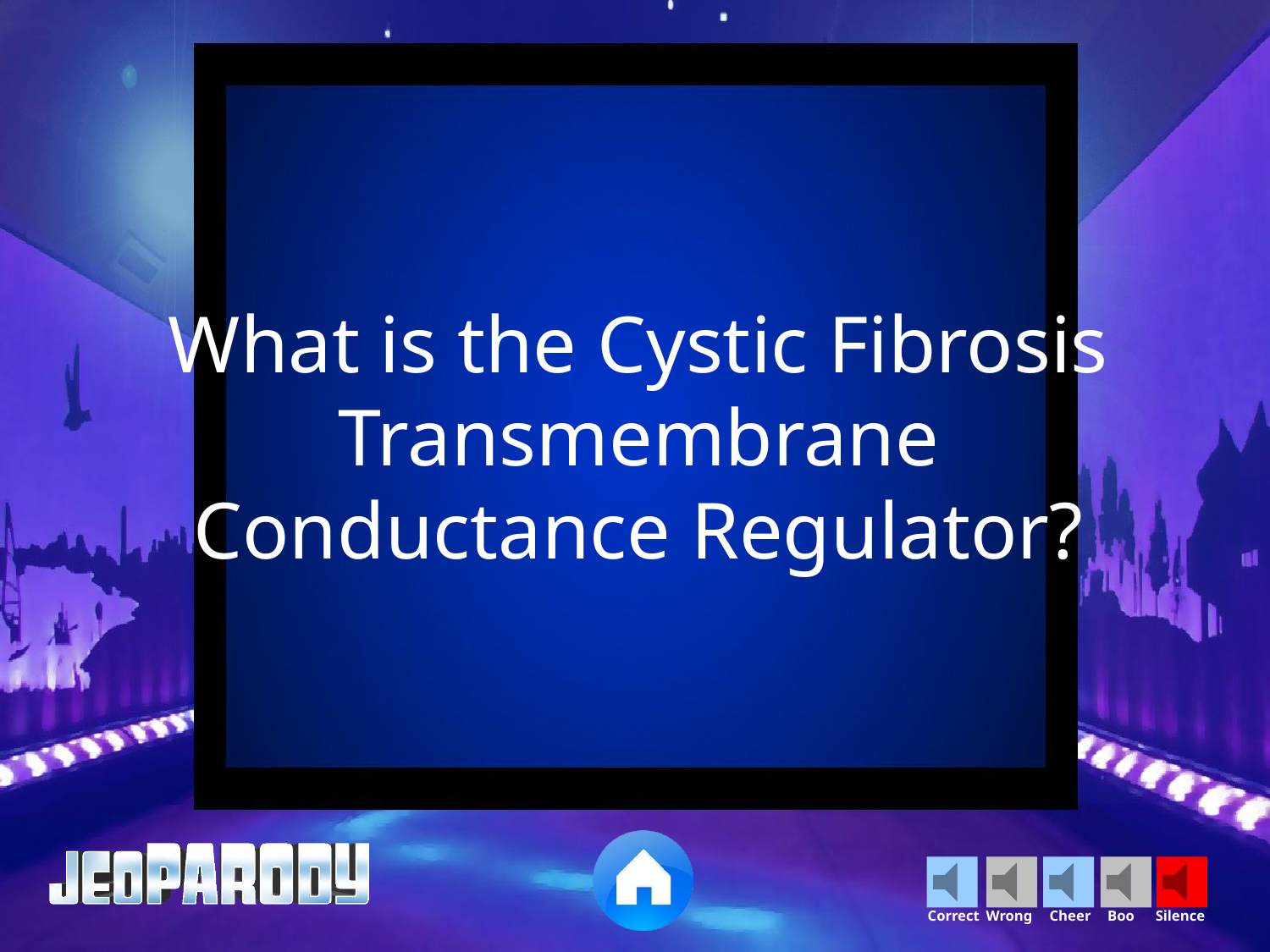

What is the Cystic Fibrosis Transmembrane Conductance Regulator?

## Slide 19
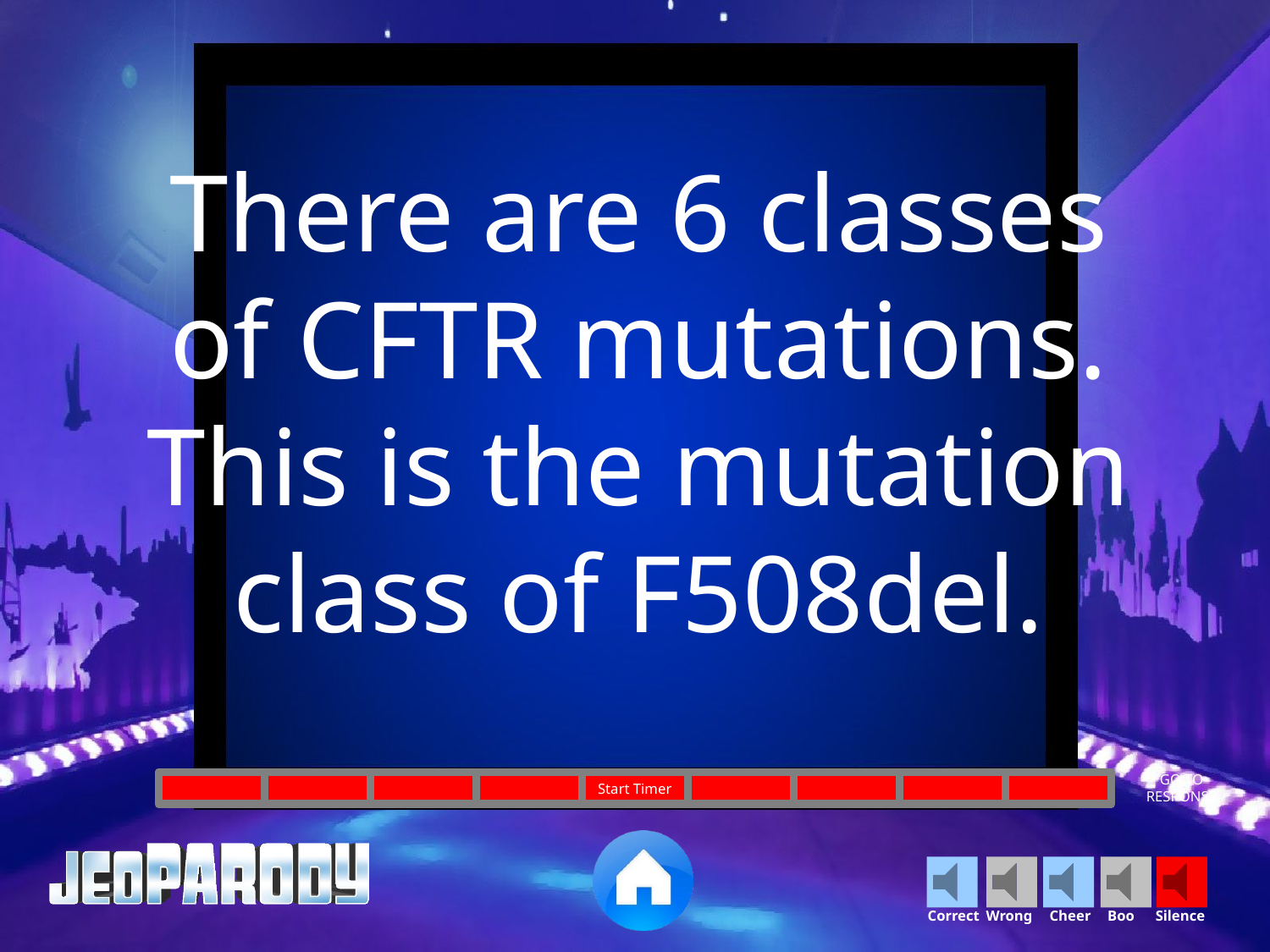

There are 6 classes of CFTR mutations. This is the mutation class of F508del.

## Slide 20
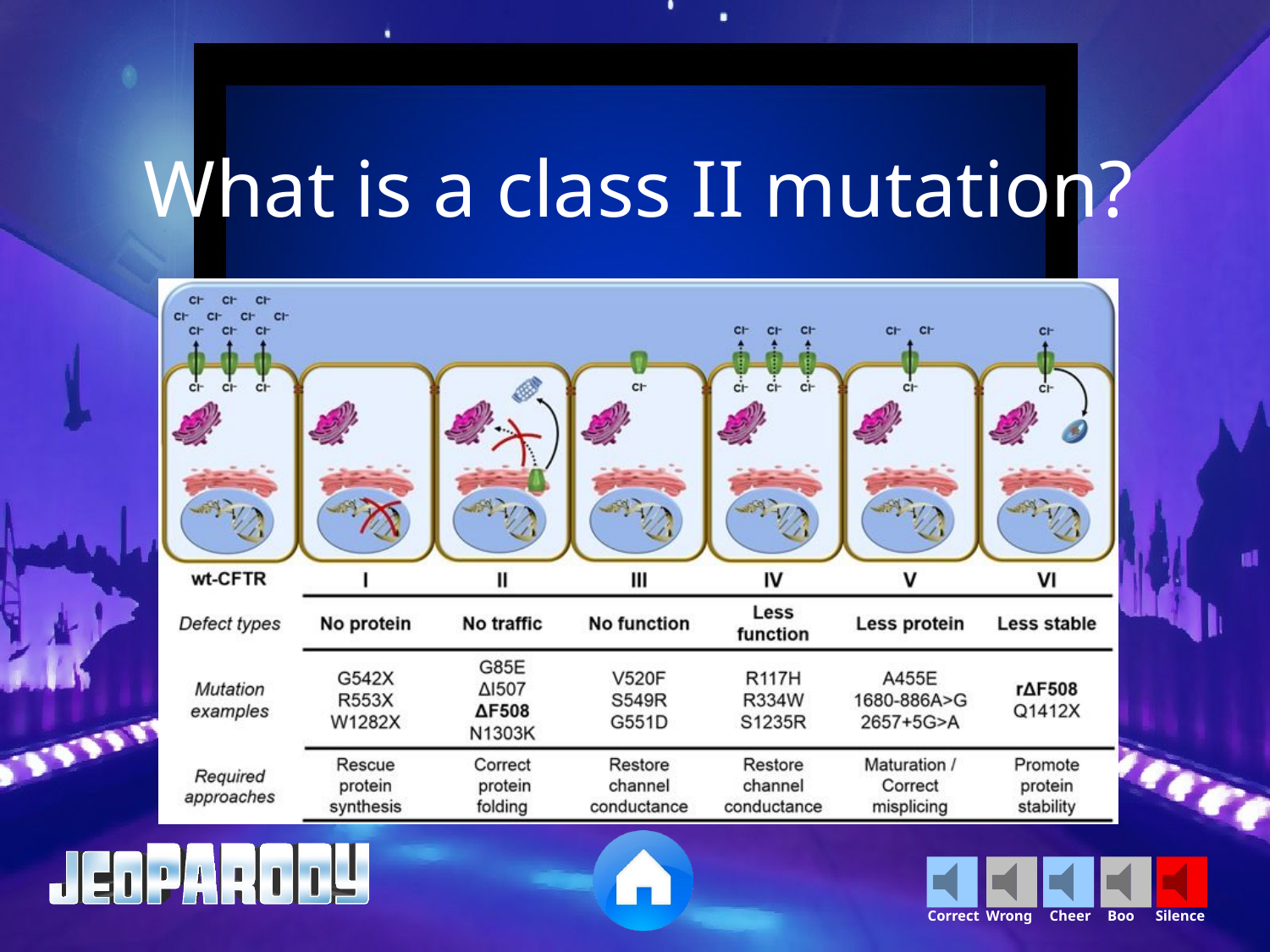

What is a class II mutation?

## Slide 21
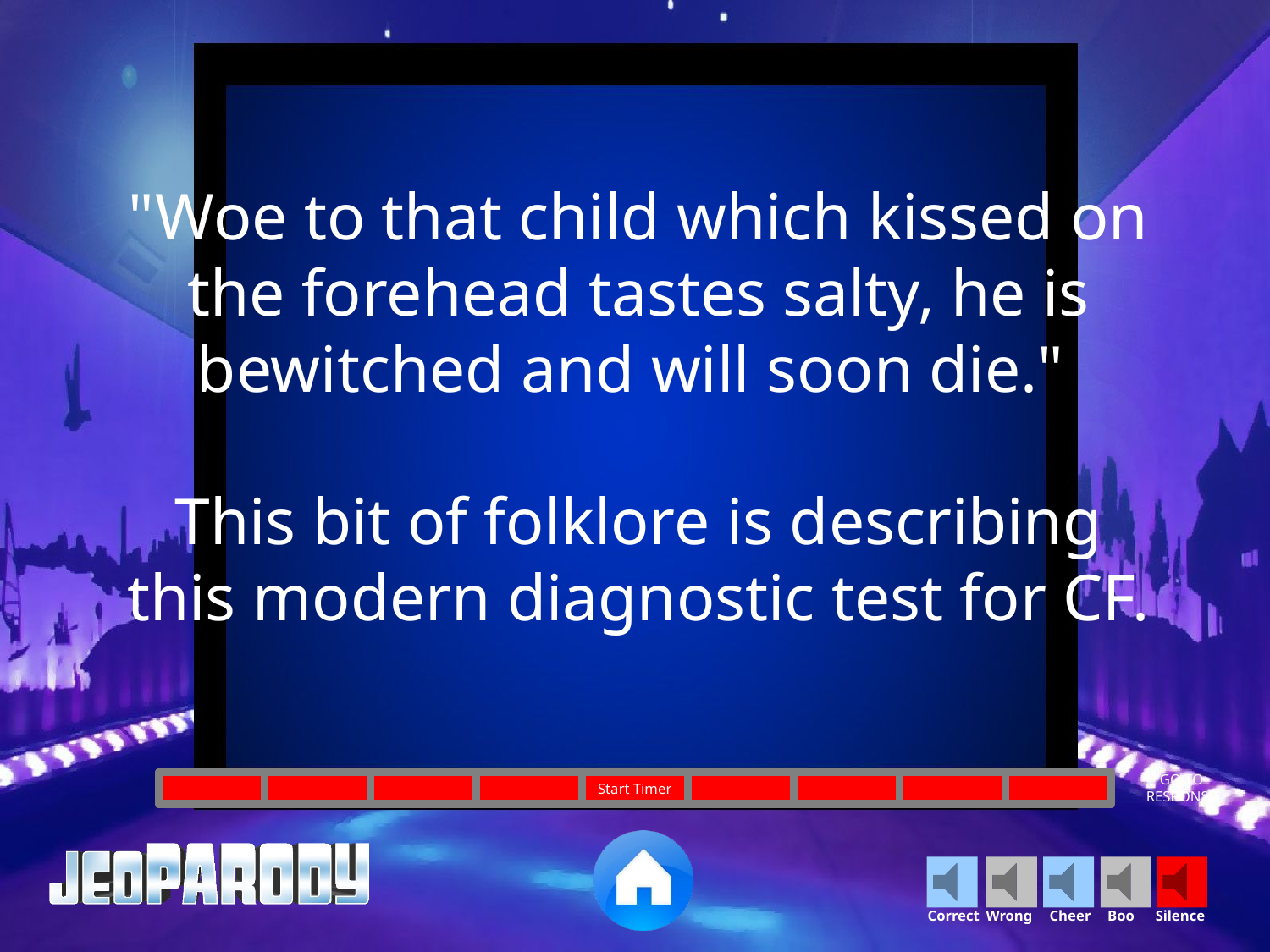

"Woe to that child which kissed on the forehead tastes salty, he is bewitched and will soon die."
This bit of folklore is describing this modern diagnostic test for CF.

## Slide 22
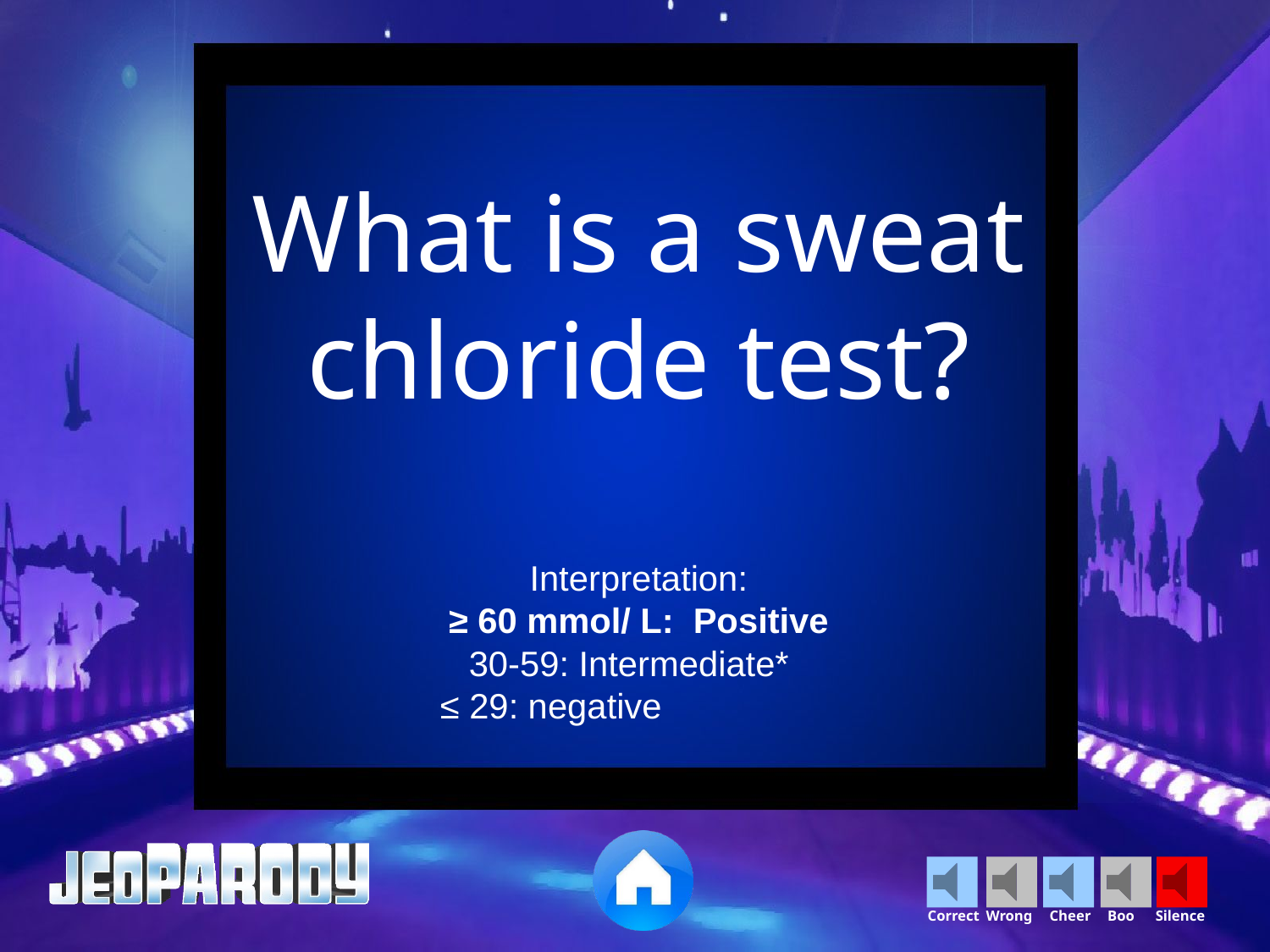

What is a sweat chloride test?
Interpretation:
≥ 60 mmol/ L: Positive
30-59: Intermediate*
≤ 29: negative

## Slide 23
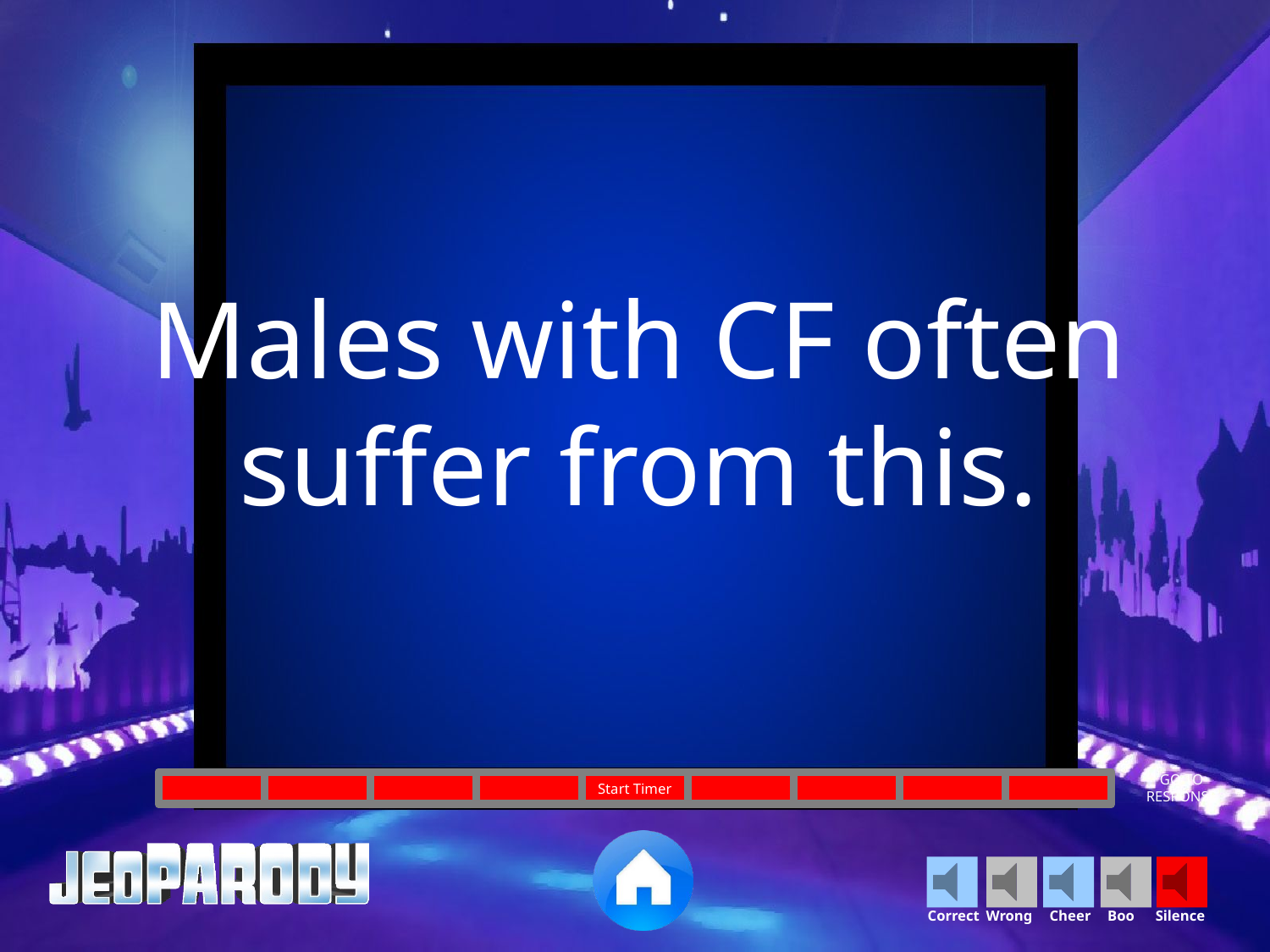

Males with CF often suffer from this.

## Slide 24
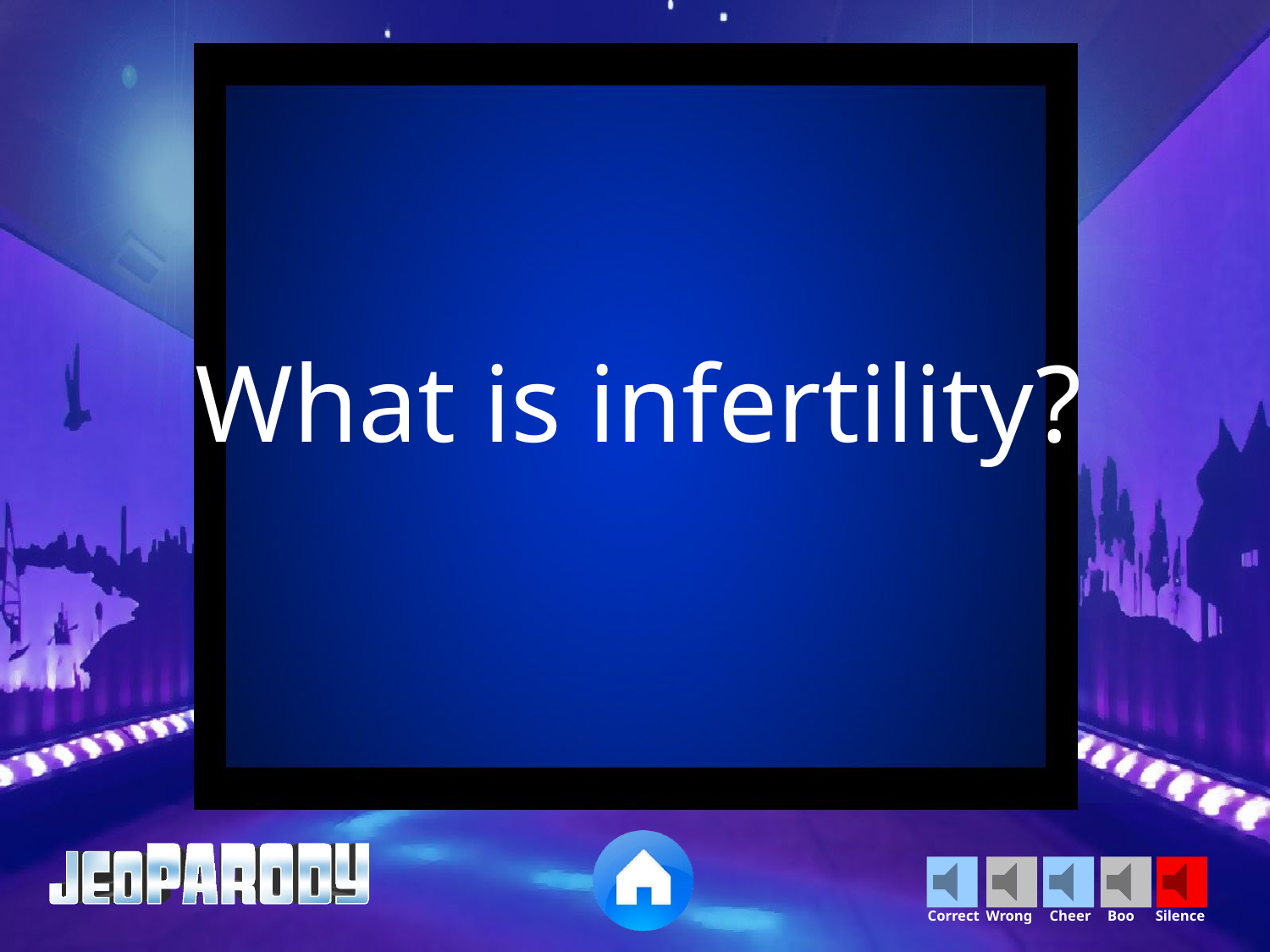

What is infertility?

## Slide 25
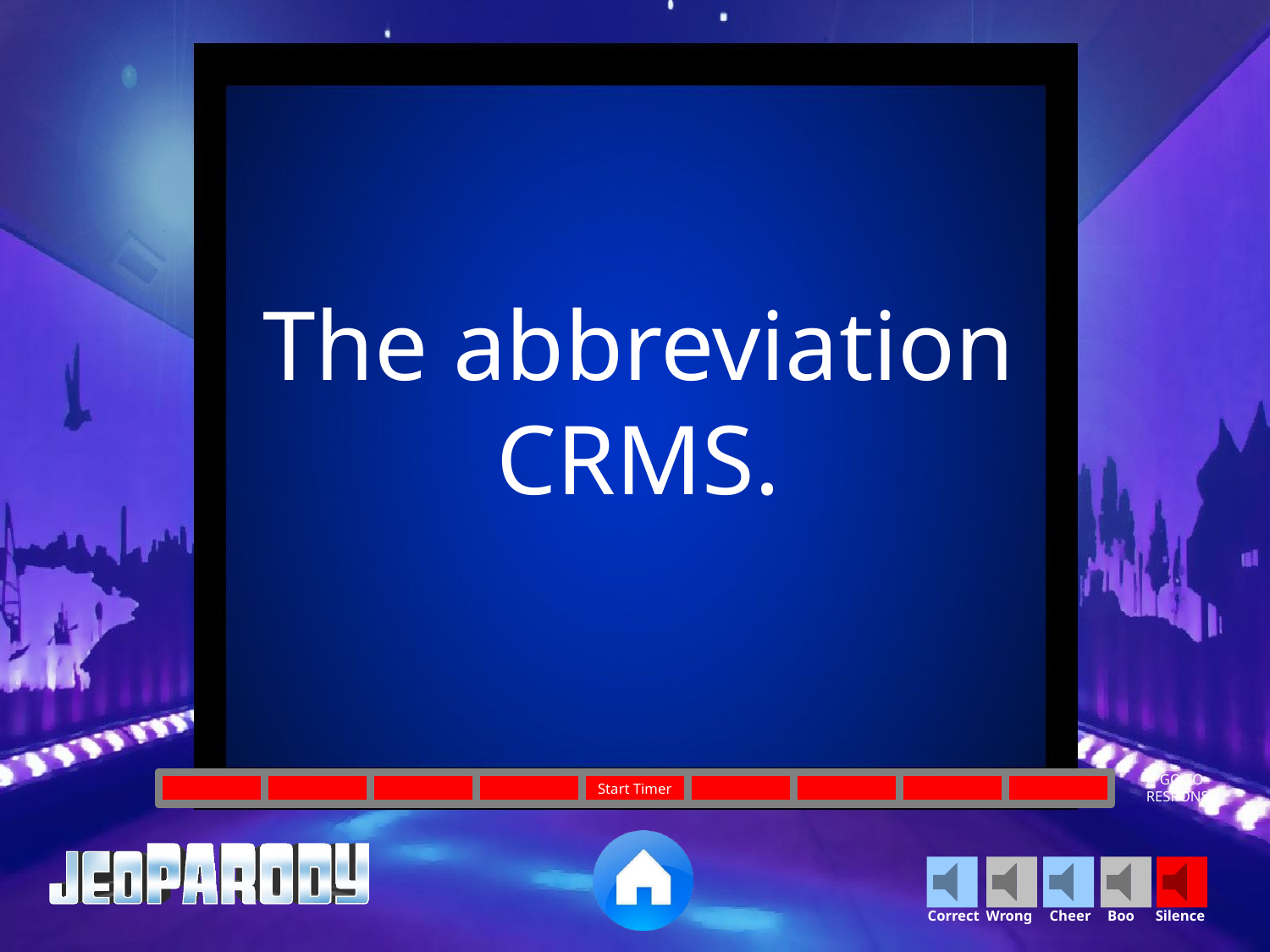

The abbreviation CRMS.

## Slide 26
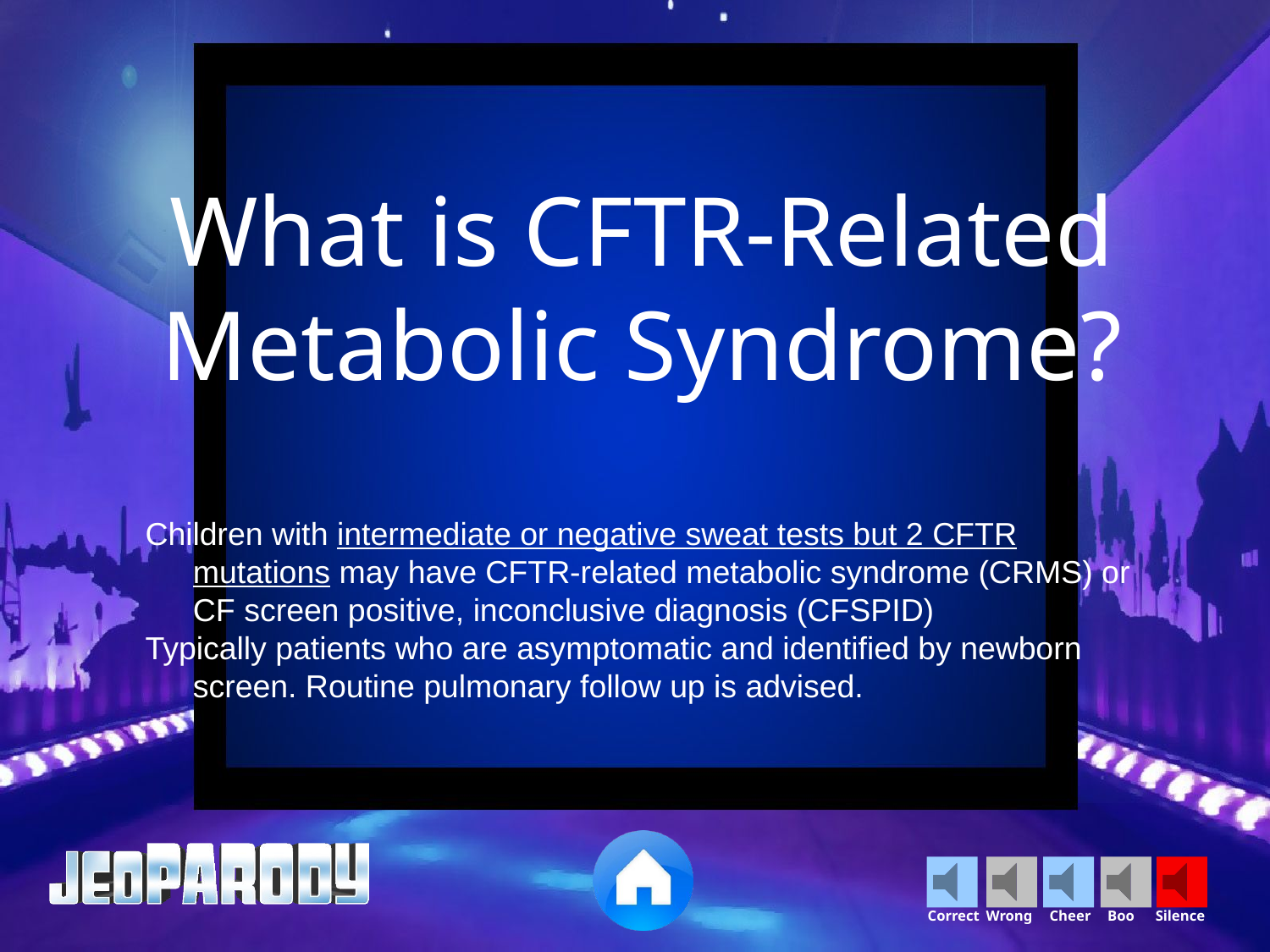

What is CFTR-Related Metabolic Syndrome?
Children with intermediate or negative sweat tests but 2 CFTR mutations may have CFTR-related metabolic syndrome (CRMS) or CF screen positive, inconclusive diagnosis (CFSPID)
Typically patients who are asymptomatic and identified by newborn screen. Routine pulmonary follow up is advised.

## Slide 27
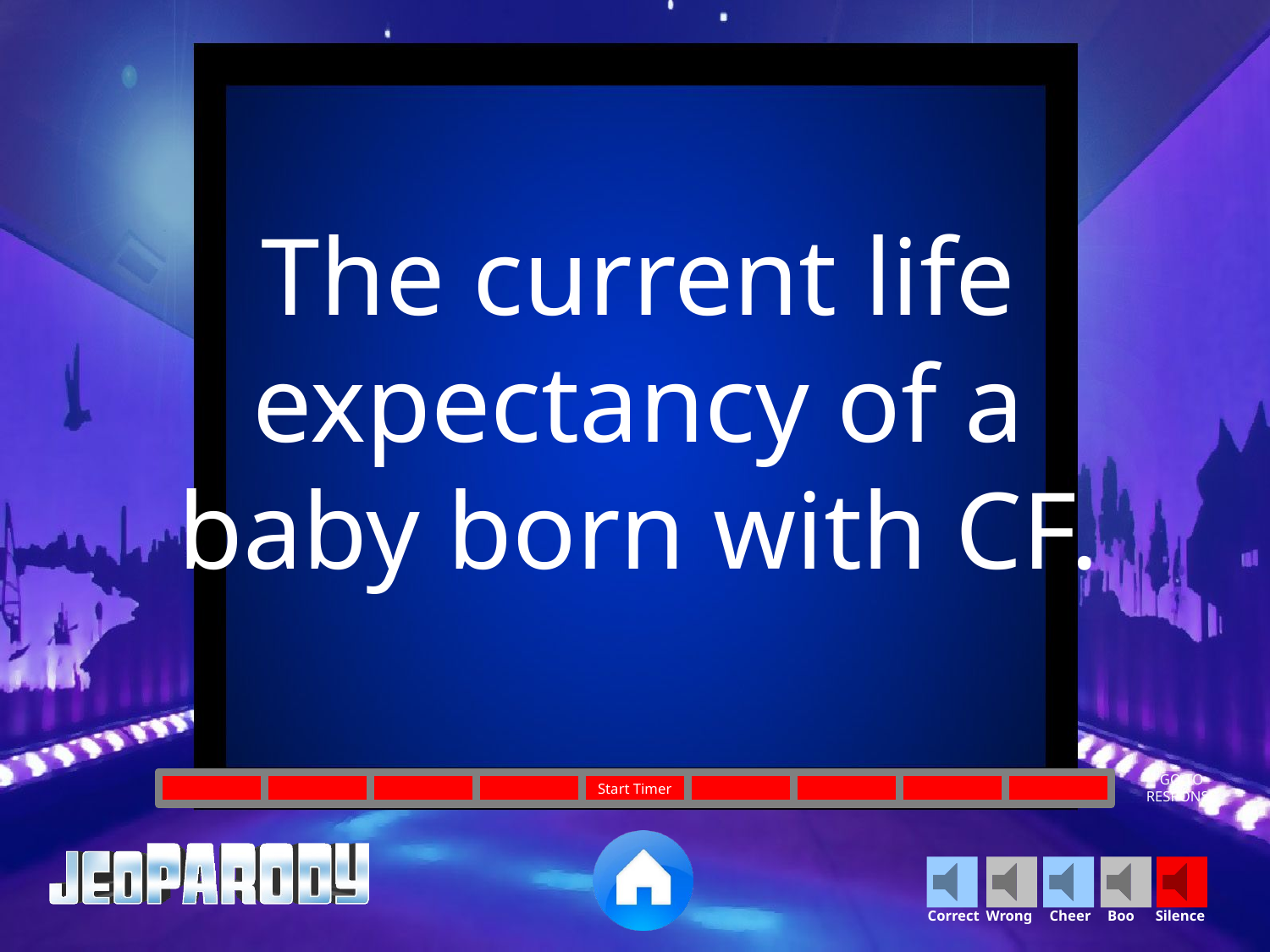

The current life expectancy of a baby born with CF.

## Slide 28
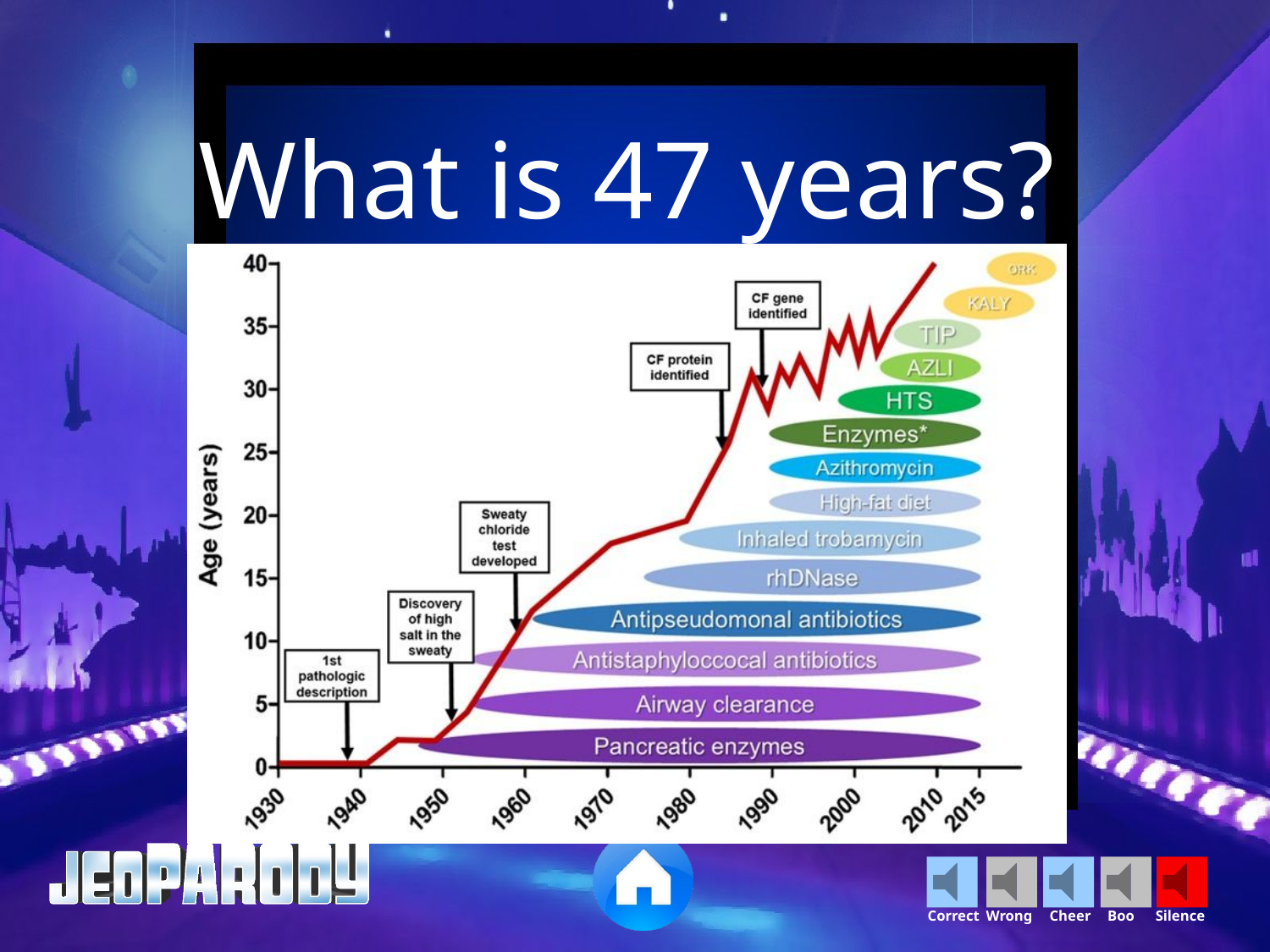

What is 47 years?

## Slide 29
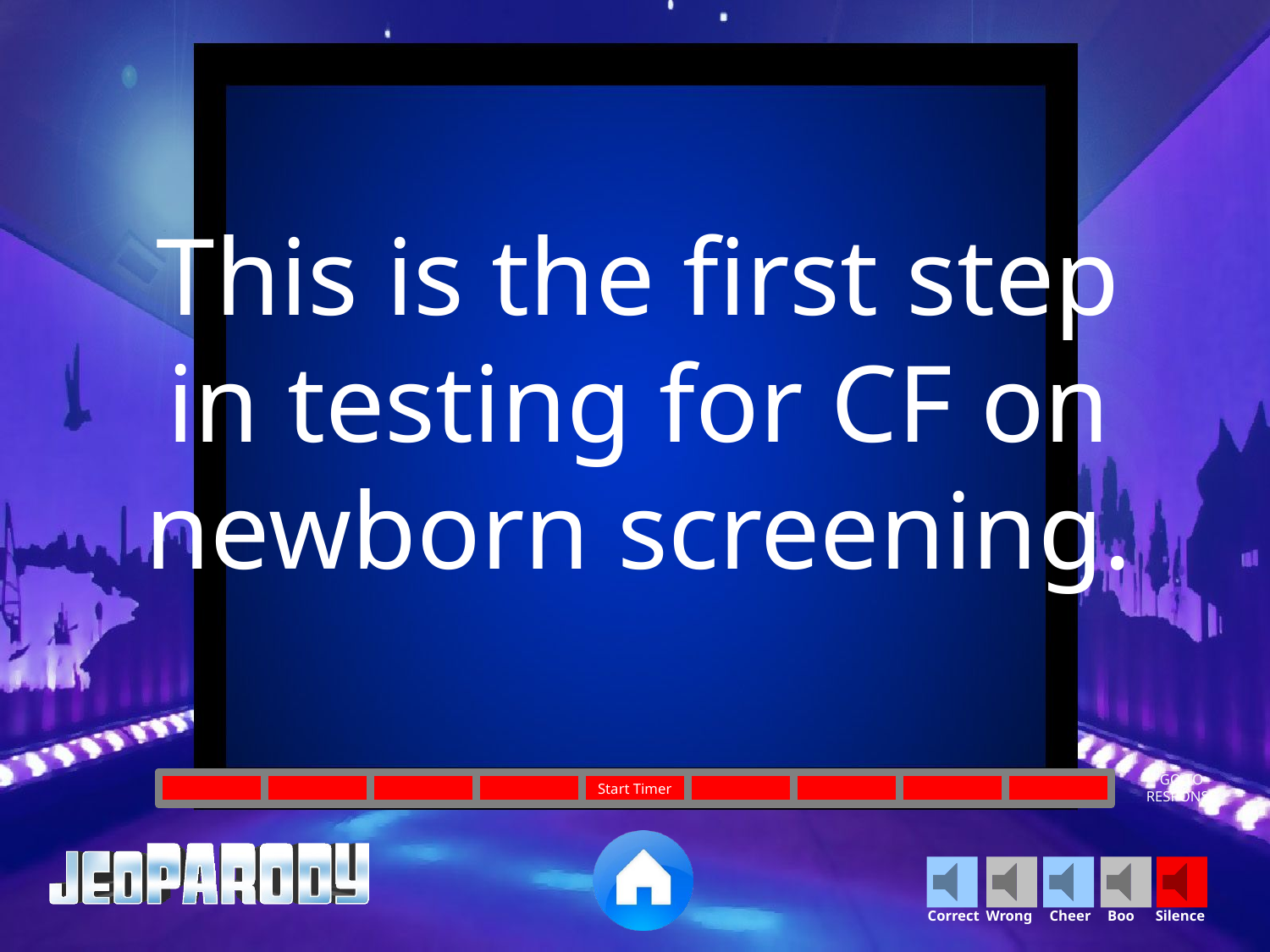

This is the first step in testing for CF on newborn screening.

## Slide 30
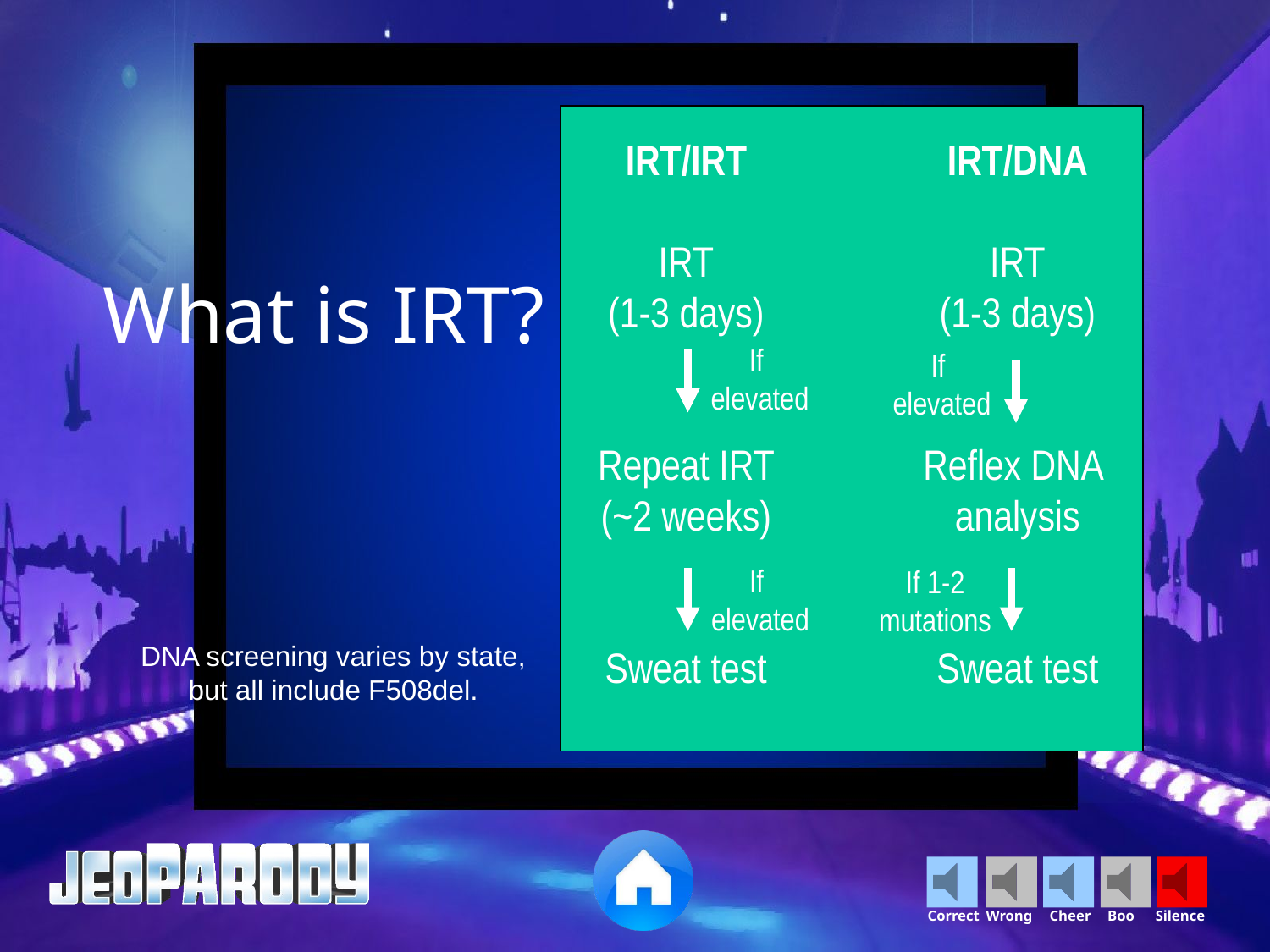

IRT/IRT
IRT
(1-3 days)
Repeat IRT
(~2 weeks)
Sweat test
IRT/DNA
IRT
(1-3 days)
Reflex DNA
analysis
Sweat test
What is IRT?
If
elevated
If
elevated
If
elevated
If 1-2
mutations
DNA screening varies by state, but all include F508del.

## Slide 31
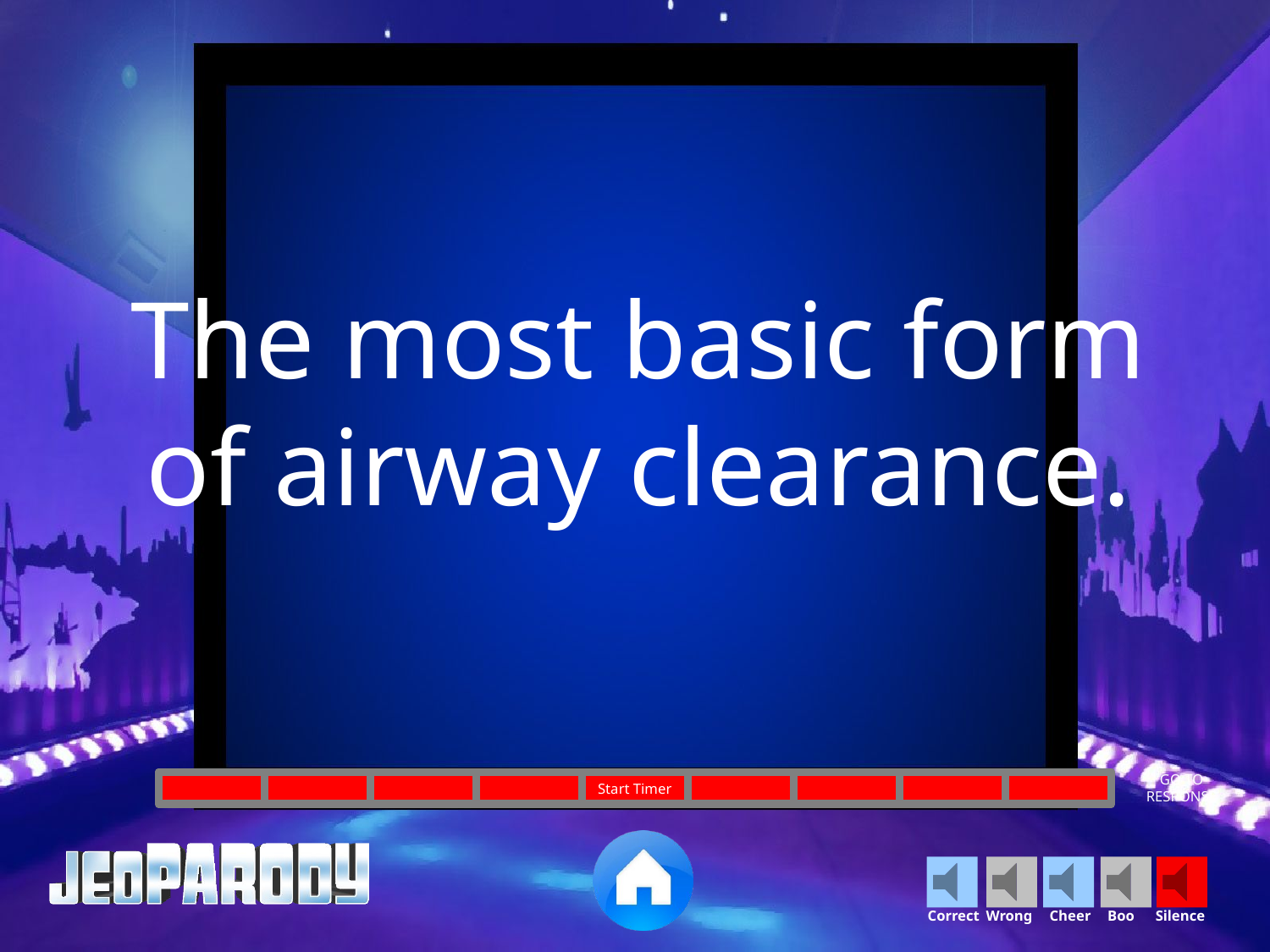

The most basic form of airway clearance.

## Slide 32
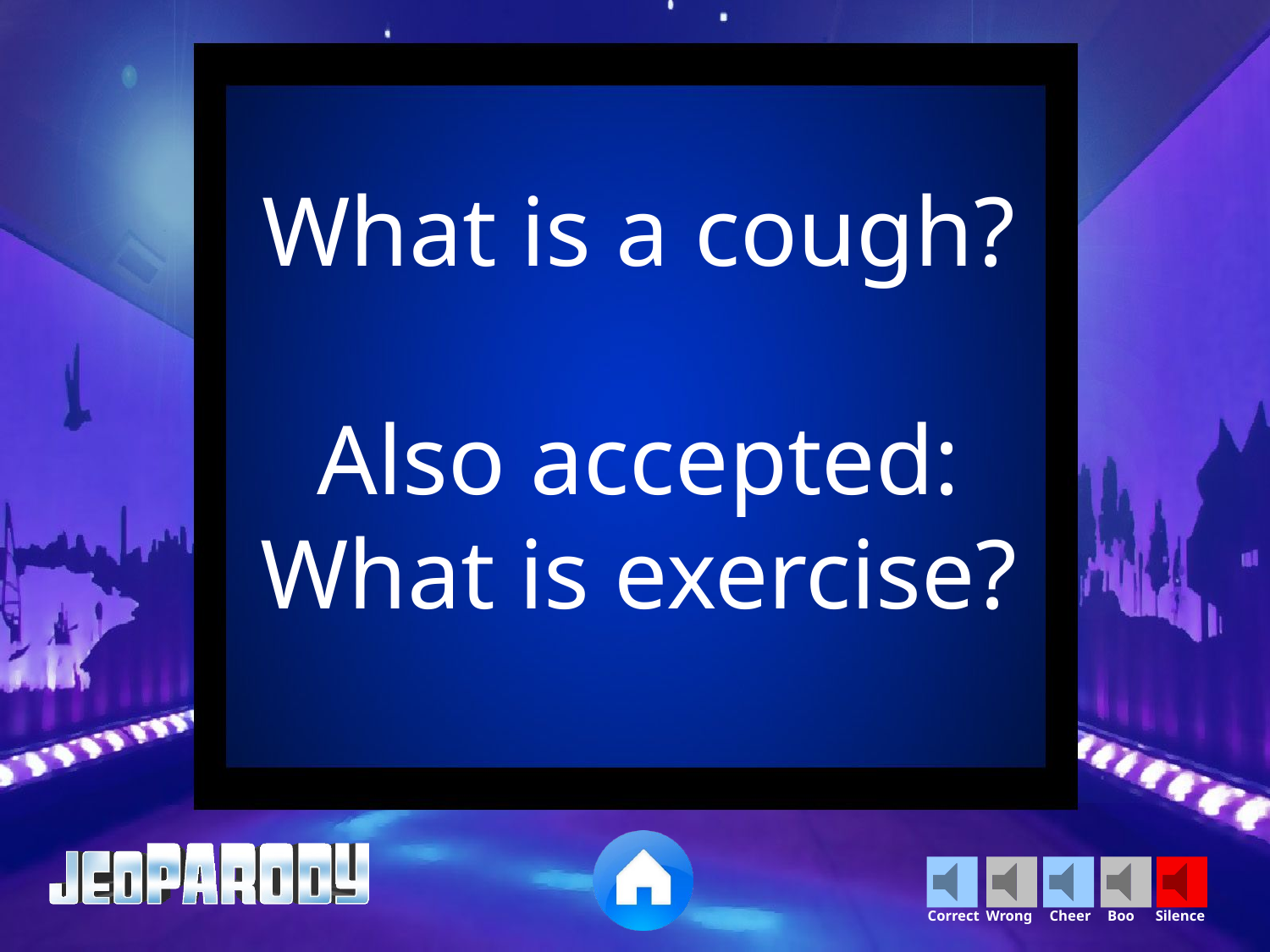

What is a cough?
Also accepted:
What is exercise?

## Slide 33
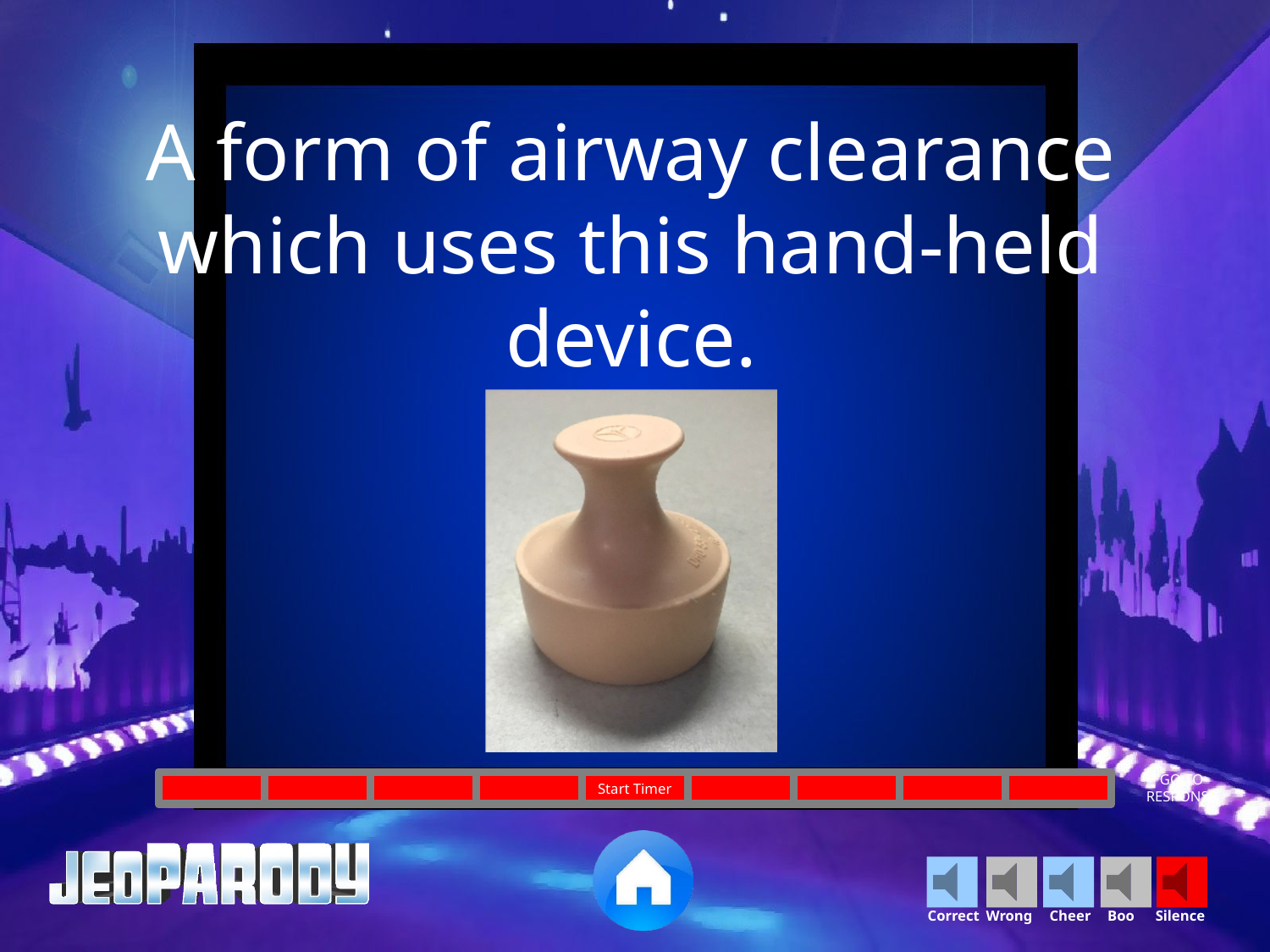

A form of airway clearance which uses this hand-held device.

## Slide 34
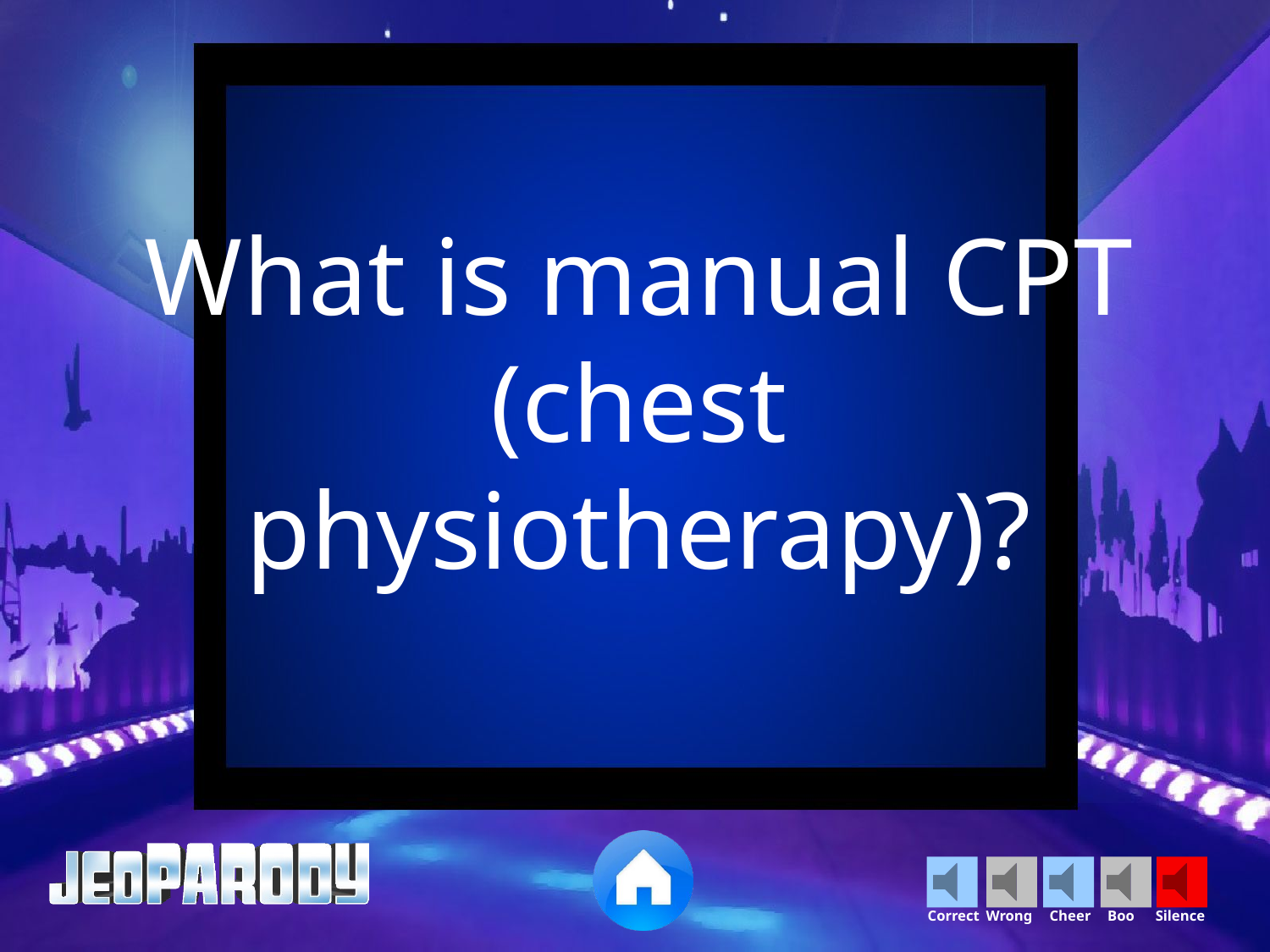

What is manual CPT (chest physiotherapy)?

## Slide 35
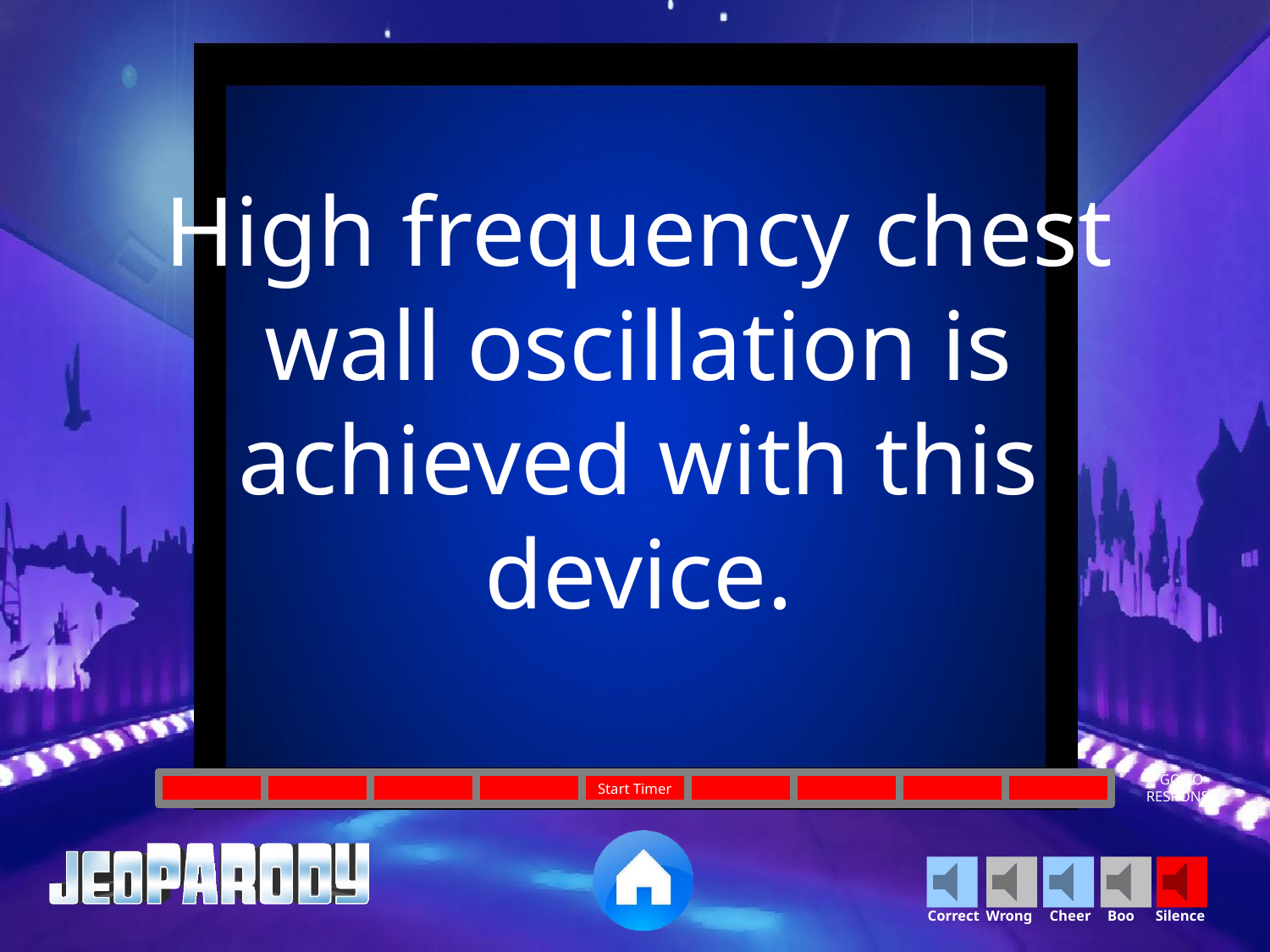

High frequency chest wall oscillation is achieved with this device.

## Slide 36
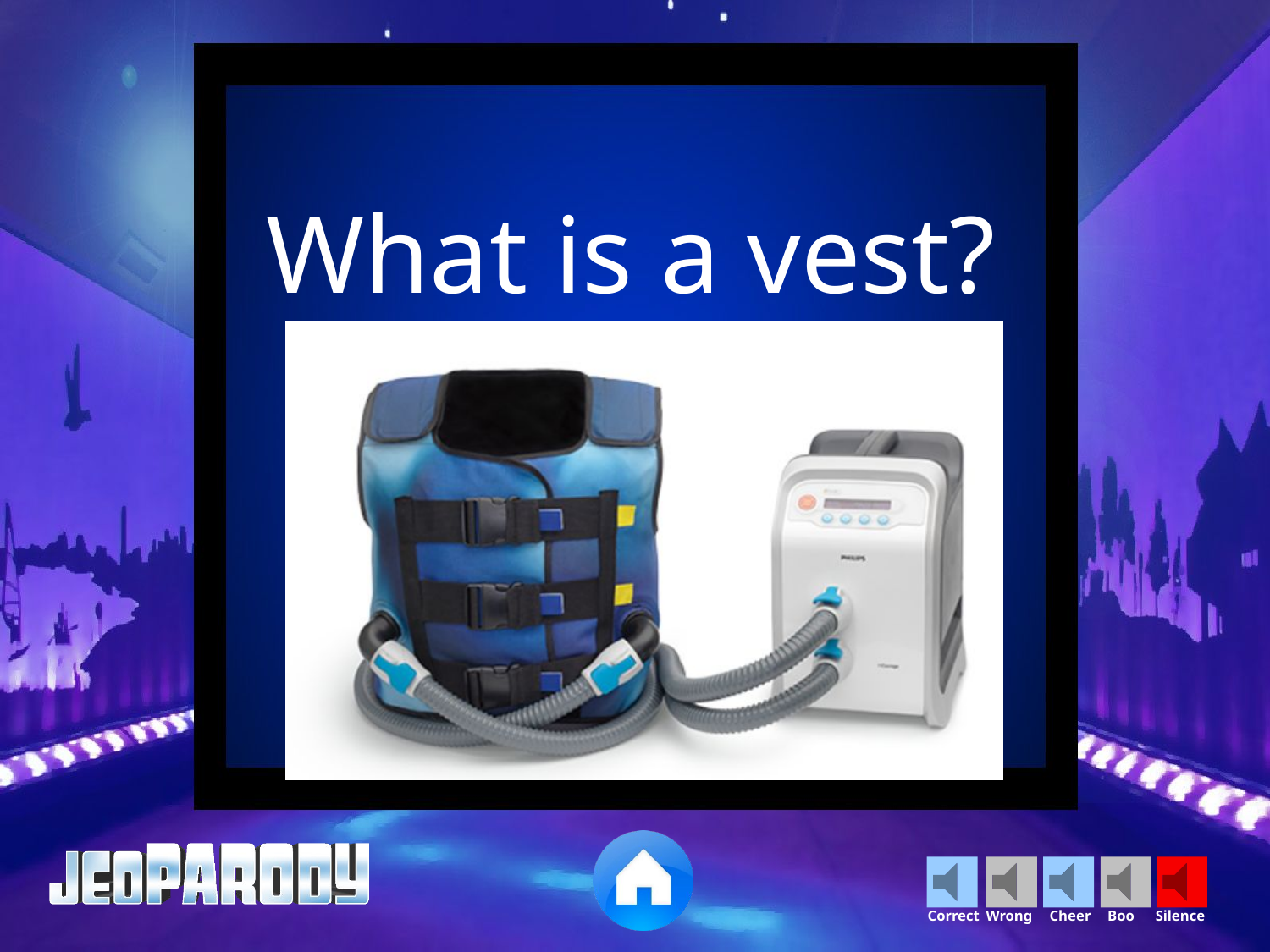

What is a vest?

## Slide 37
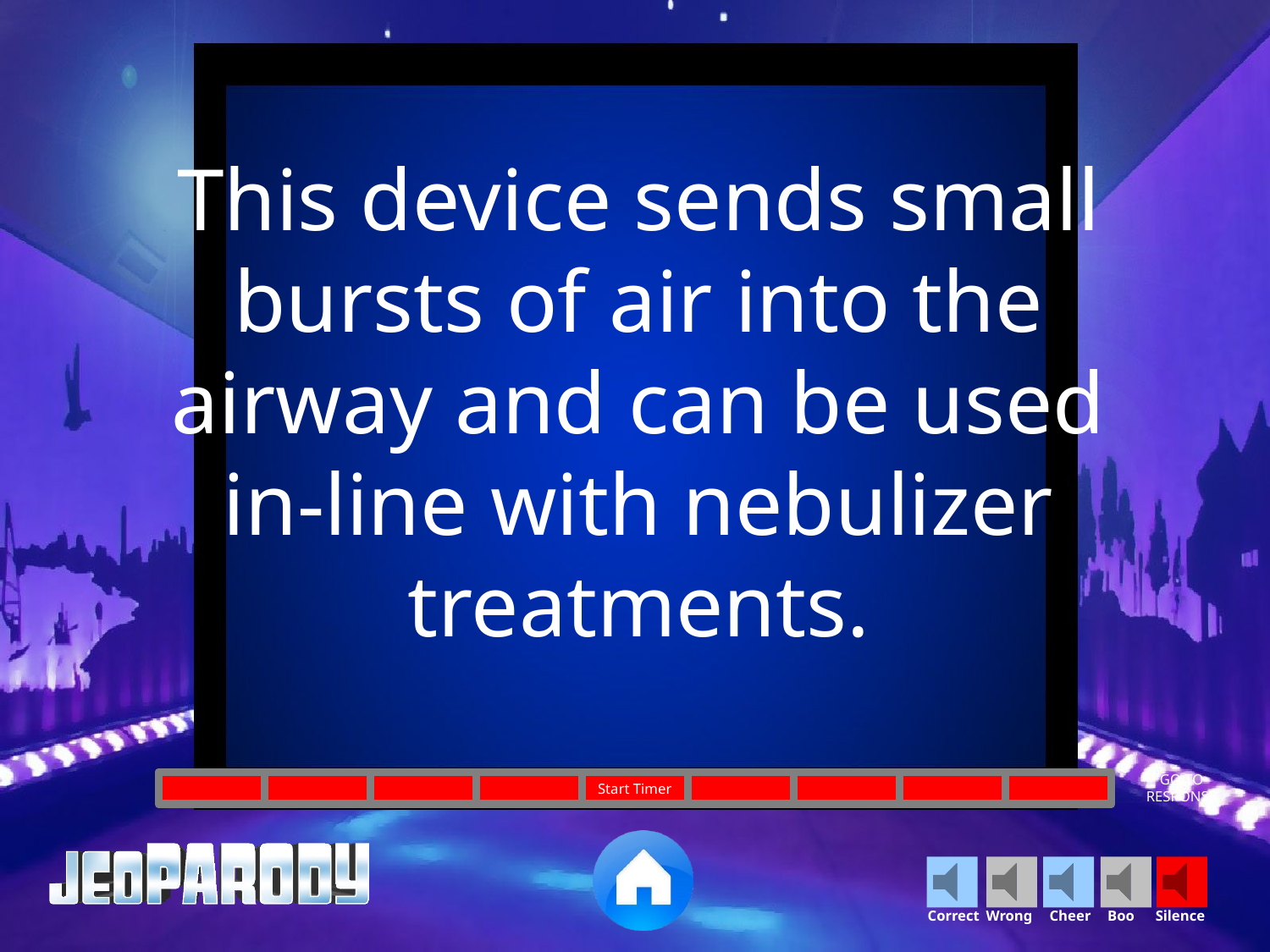

This device sends small bursts of air into the airway and can be used in-line with nebulizer treatments.

## Slide 38
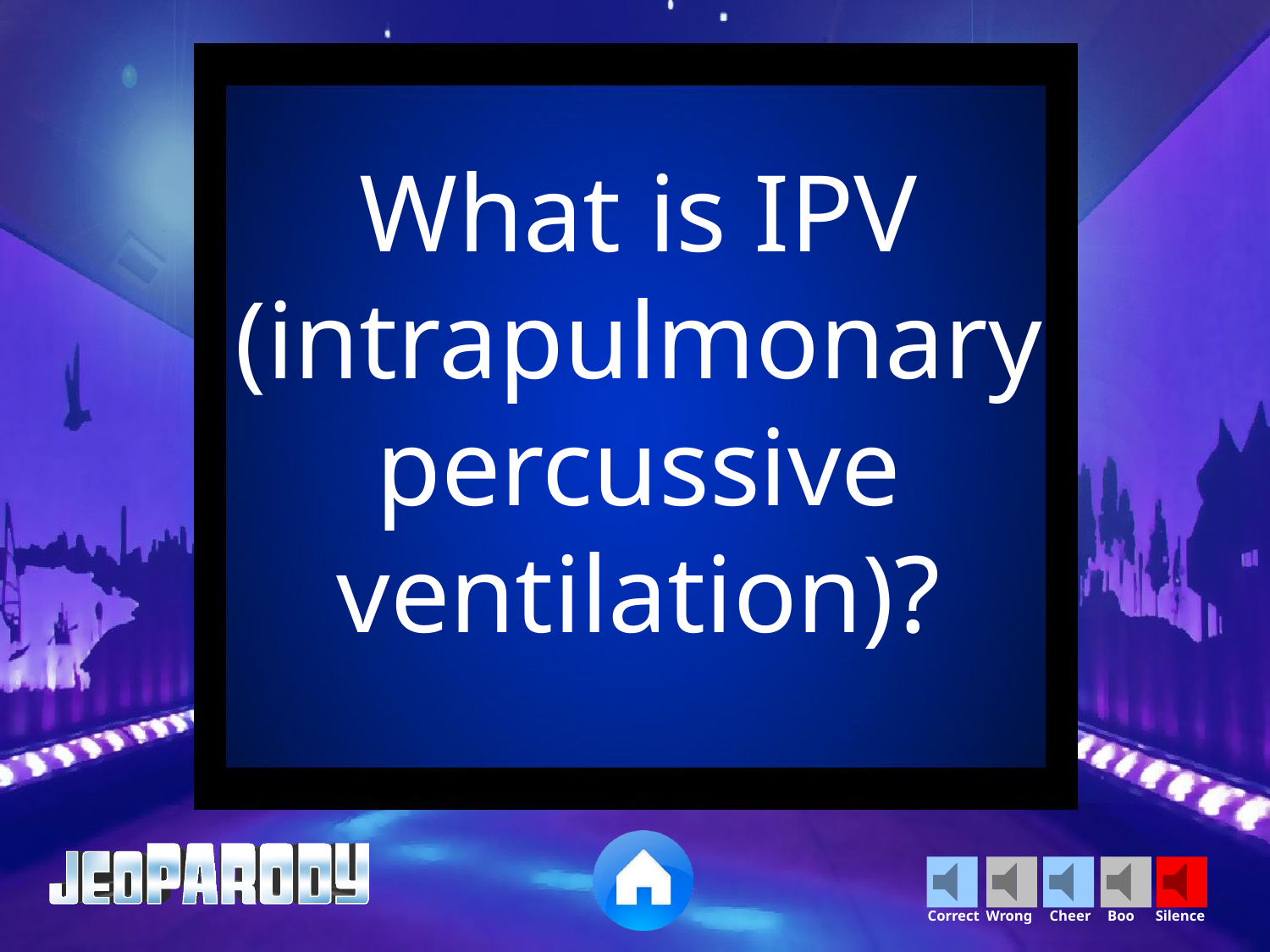

What is IPV (intrapulmonary percussive ventilation)?

## Slide 39
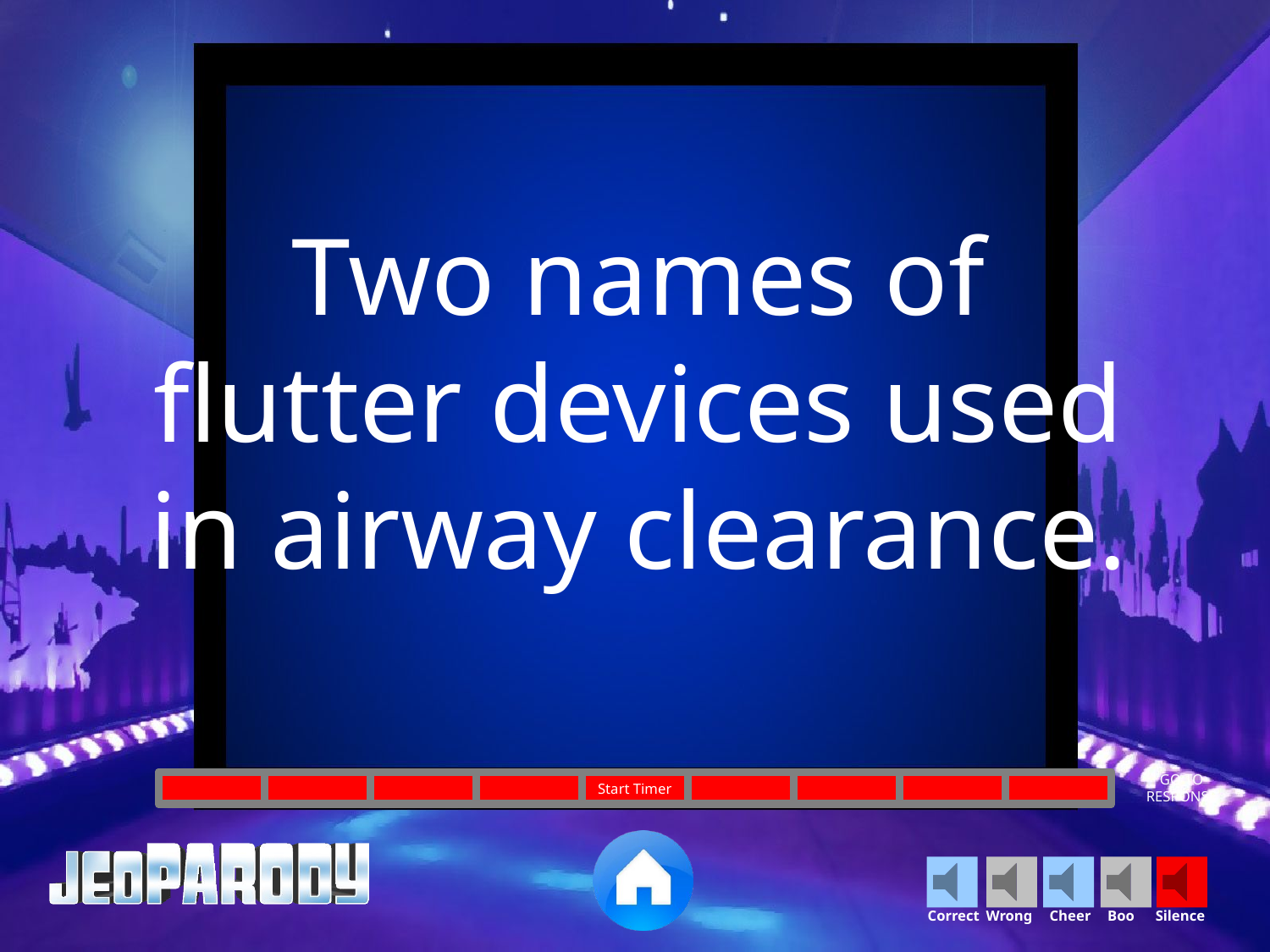

Two names of flutter devices used in airway clearance.

## Slide 40
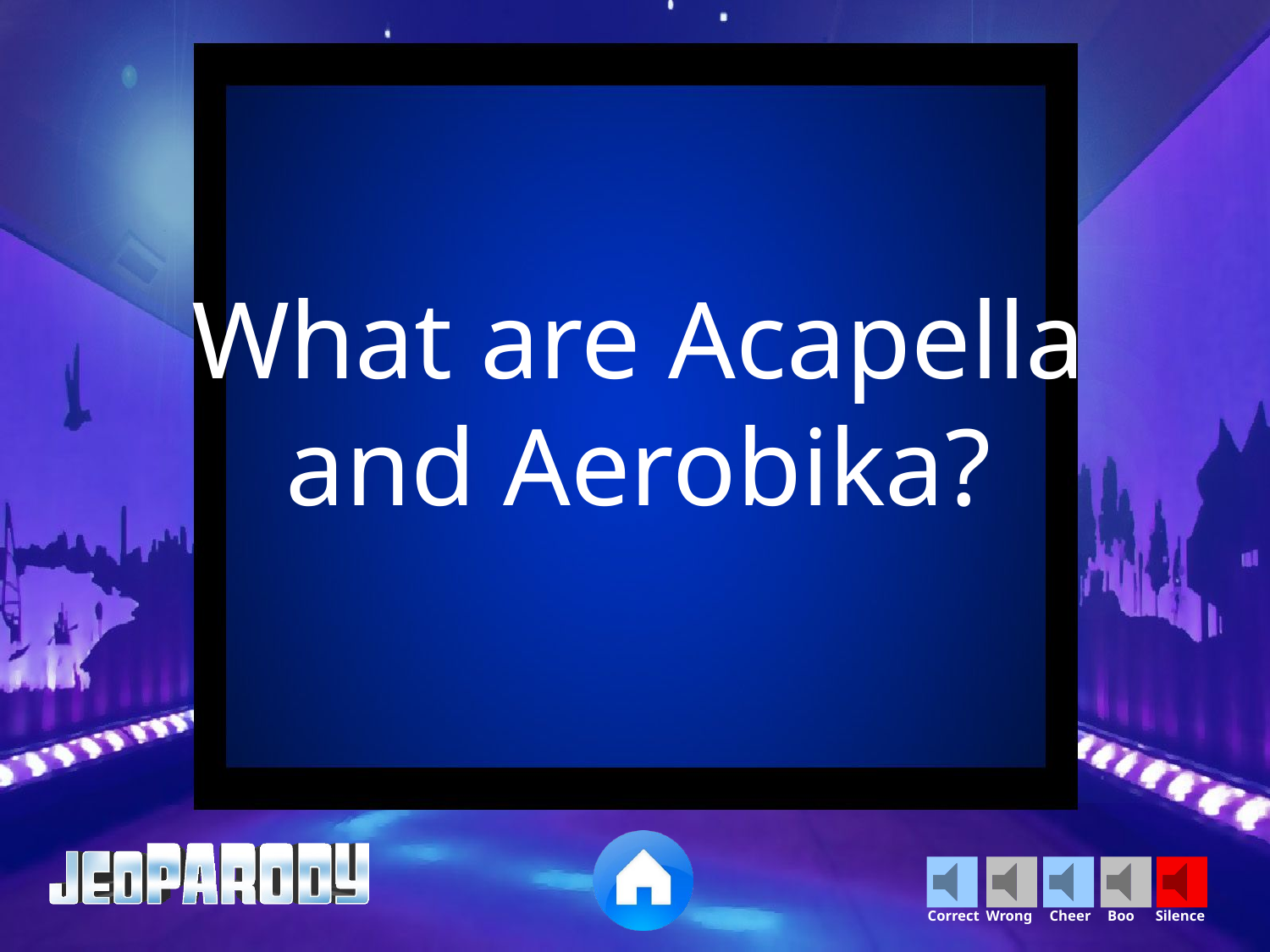

What are Acapella and Aerobika?

## Slide 41
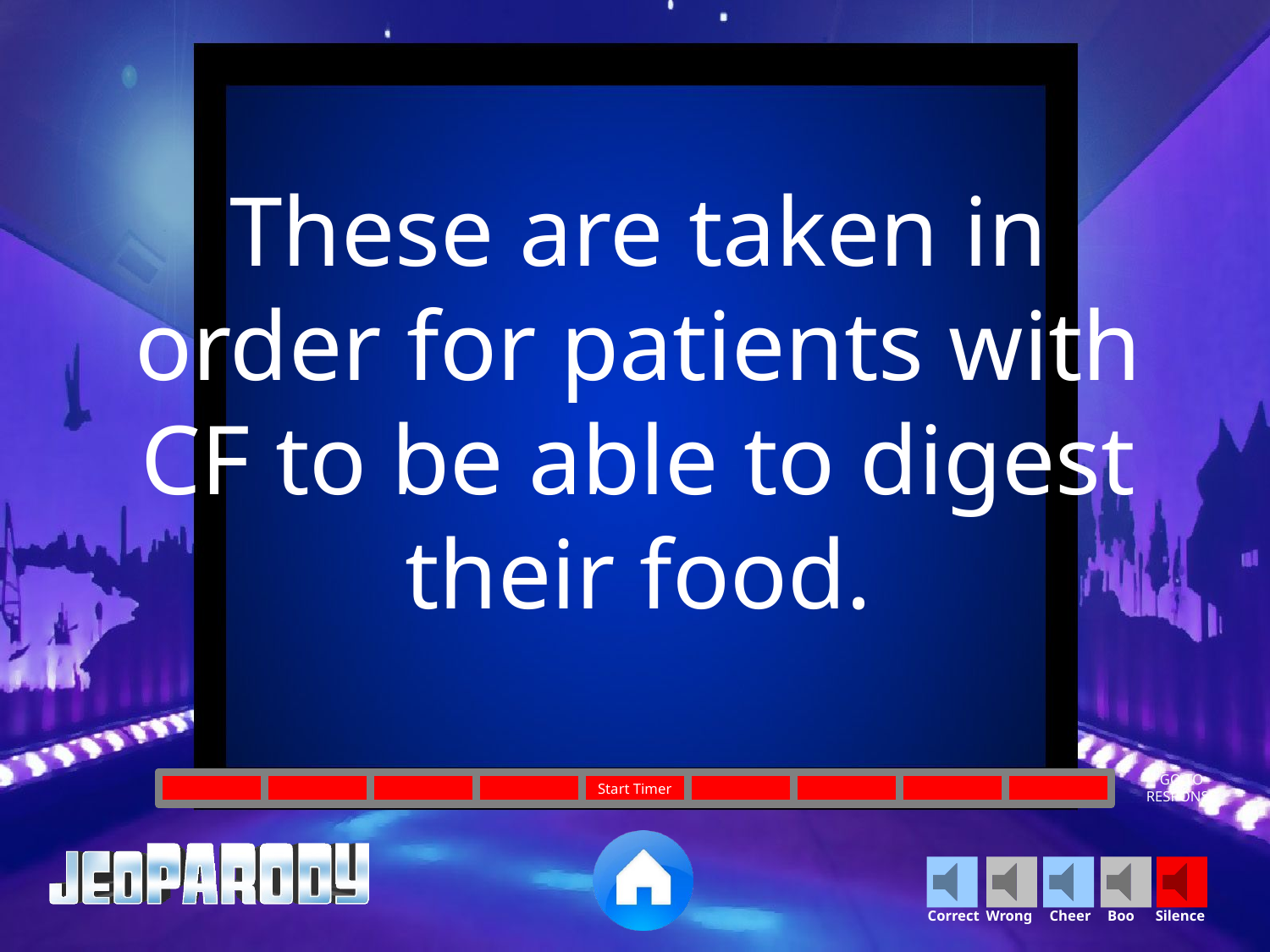

These are taken in order for patients with CF to be able to digest their food.

## Slide 42
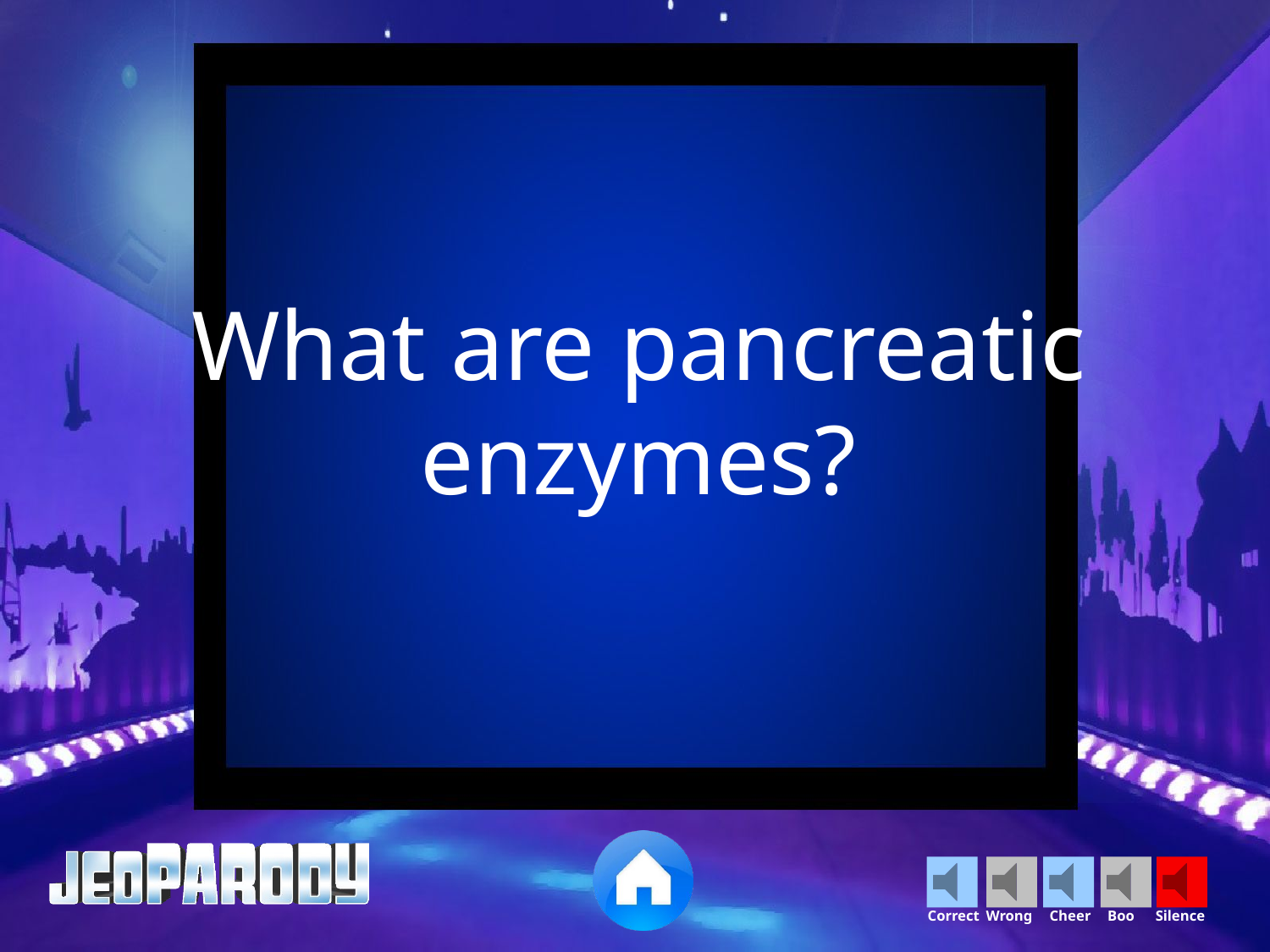

What are pancreatic enzymes?

## Slide 43
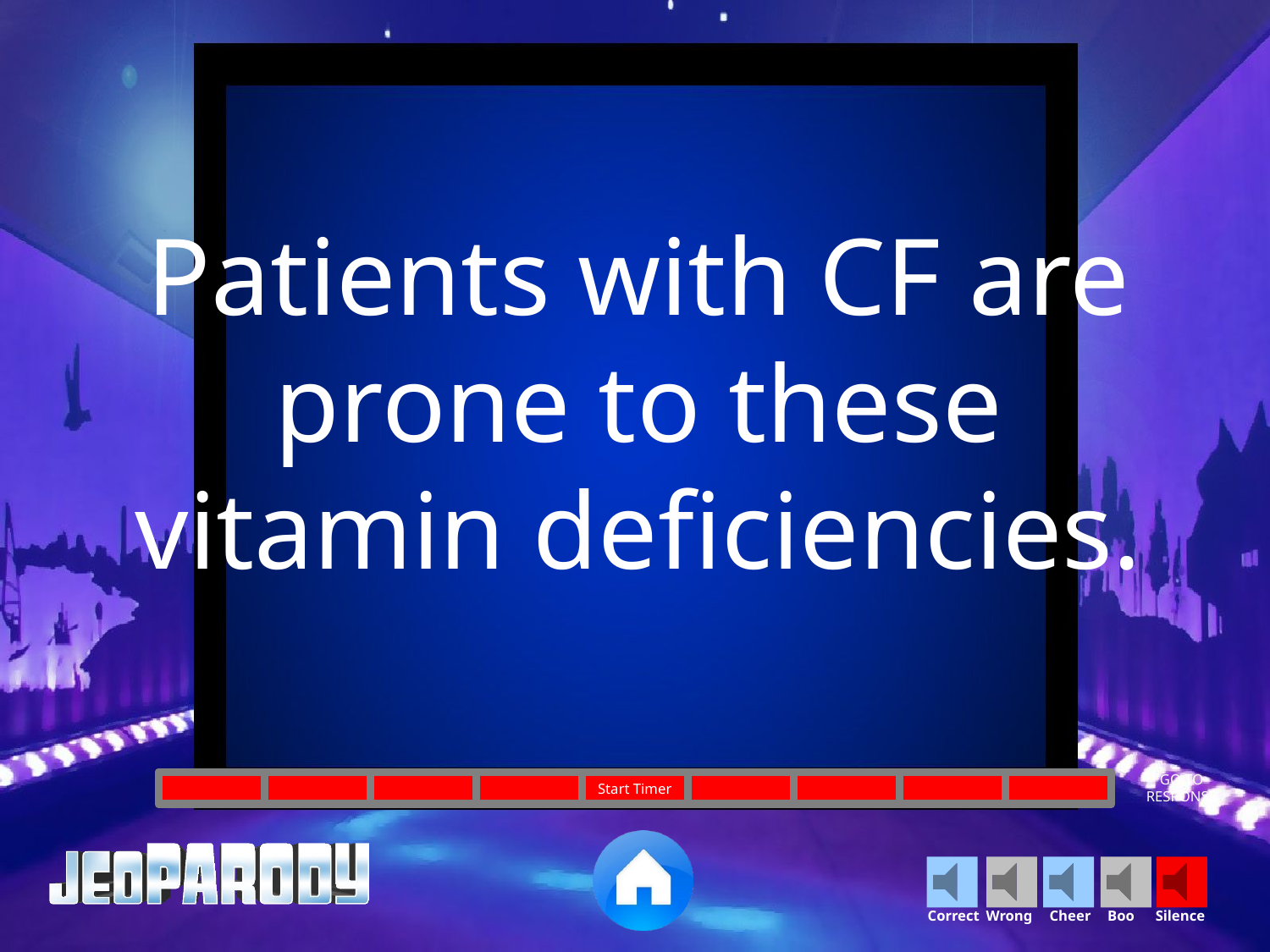

Patients with CF are prone to these vitamin deficiencies.

## Slide 44
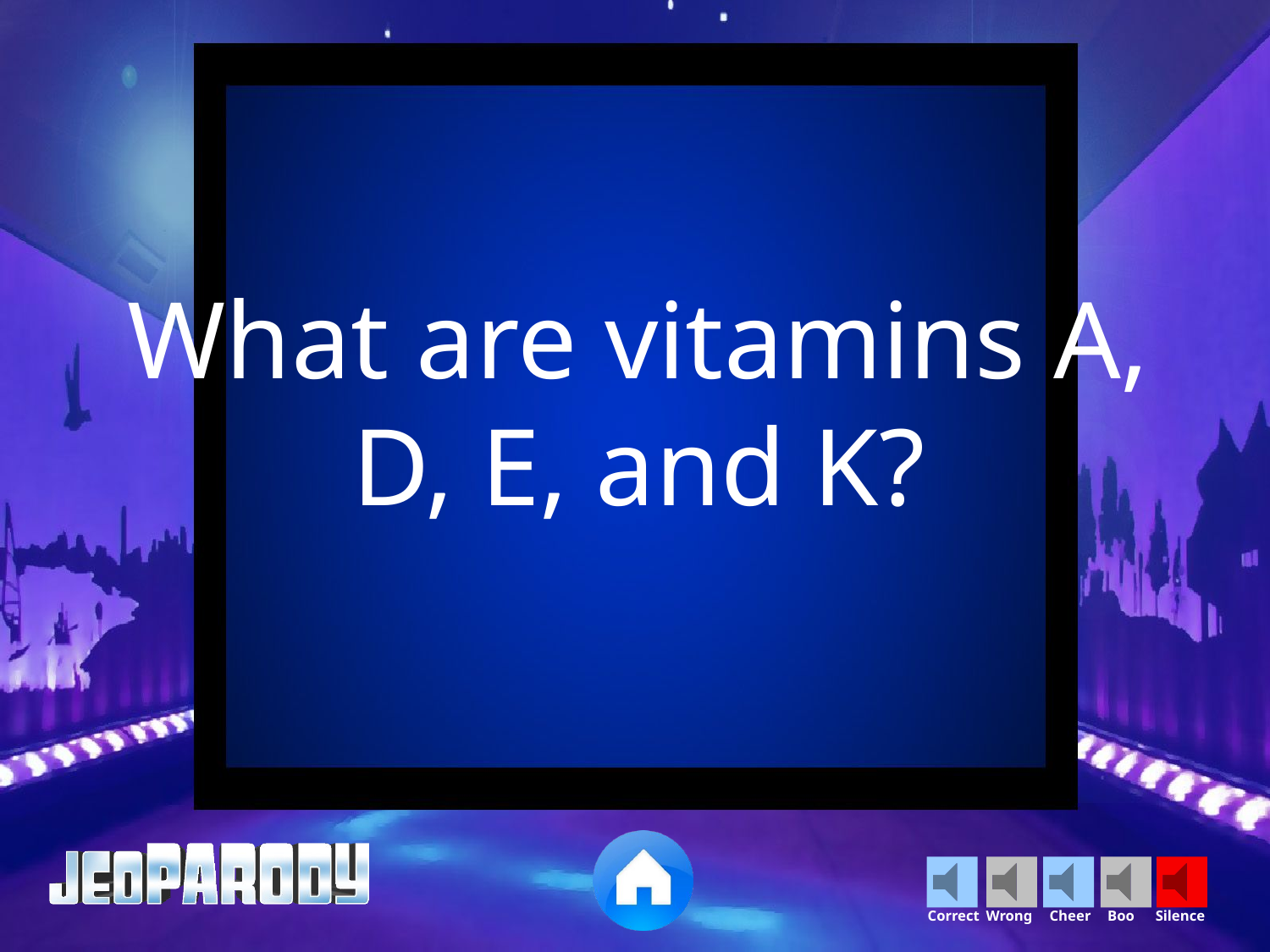

What are vitamins A, D, E, and K?

## Slide 45
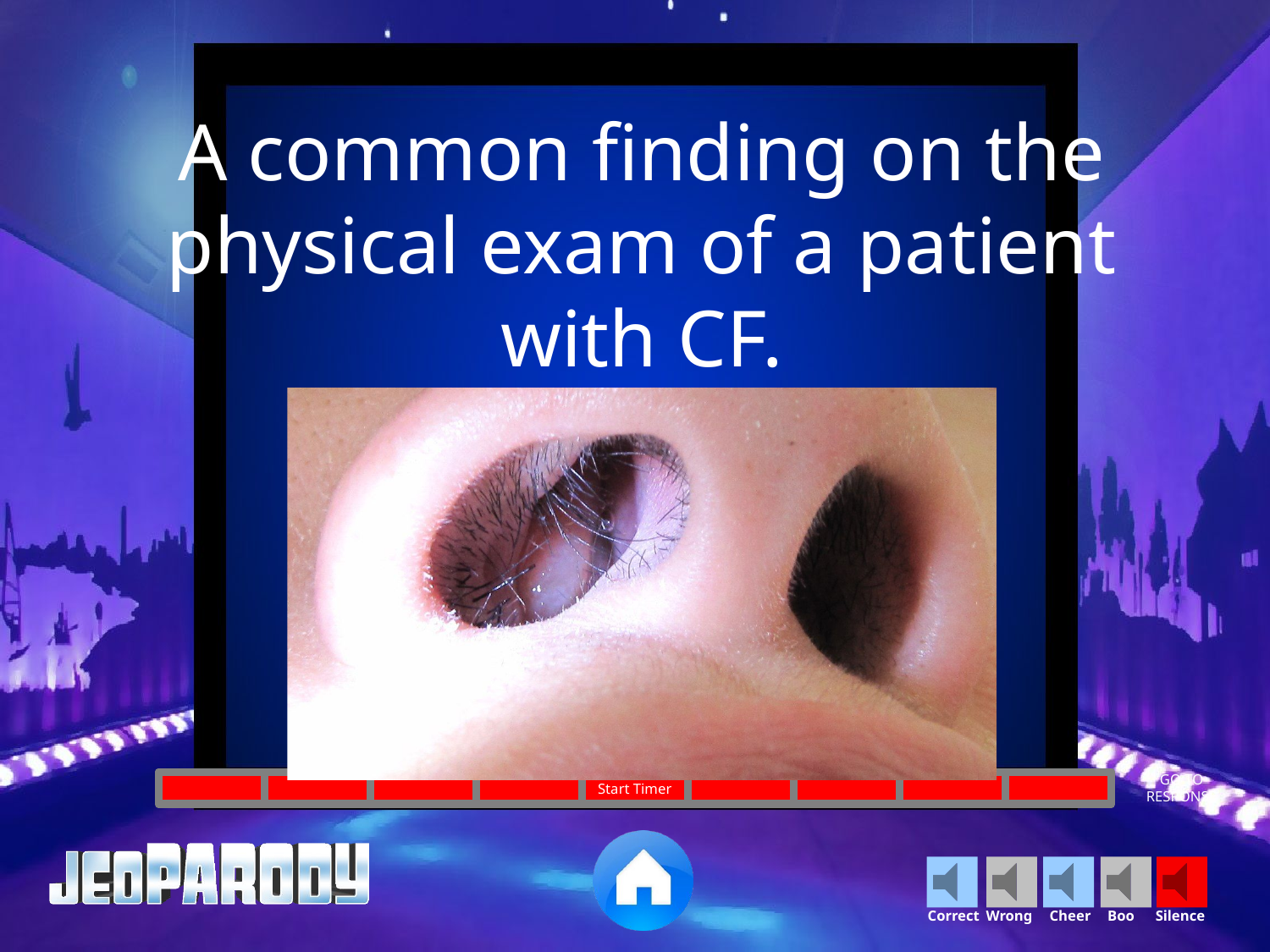

A common finding on the physical exam of a patient with CF.

## Slide 46
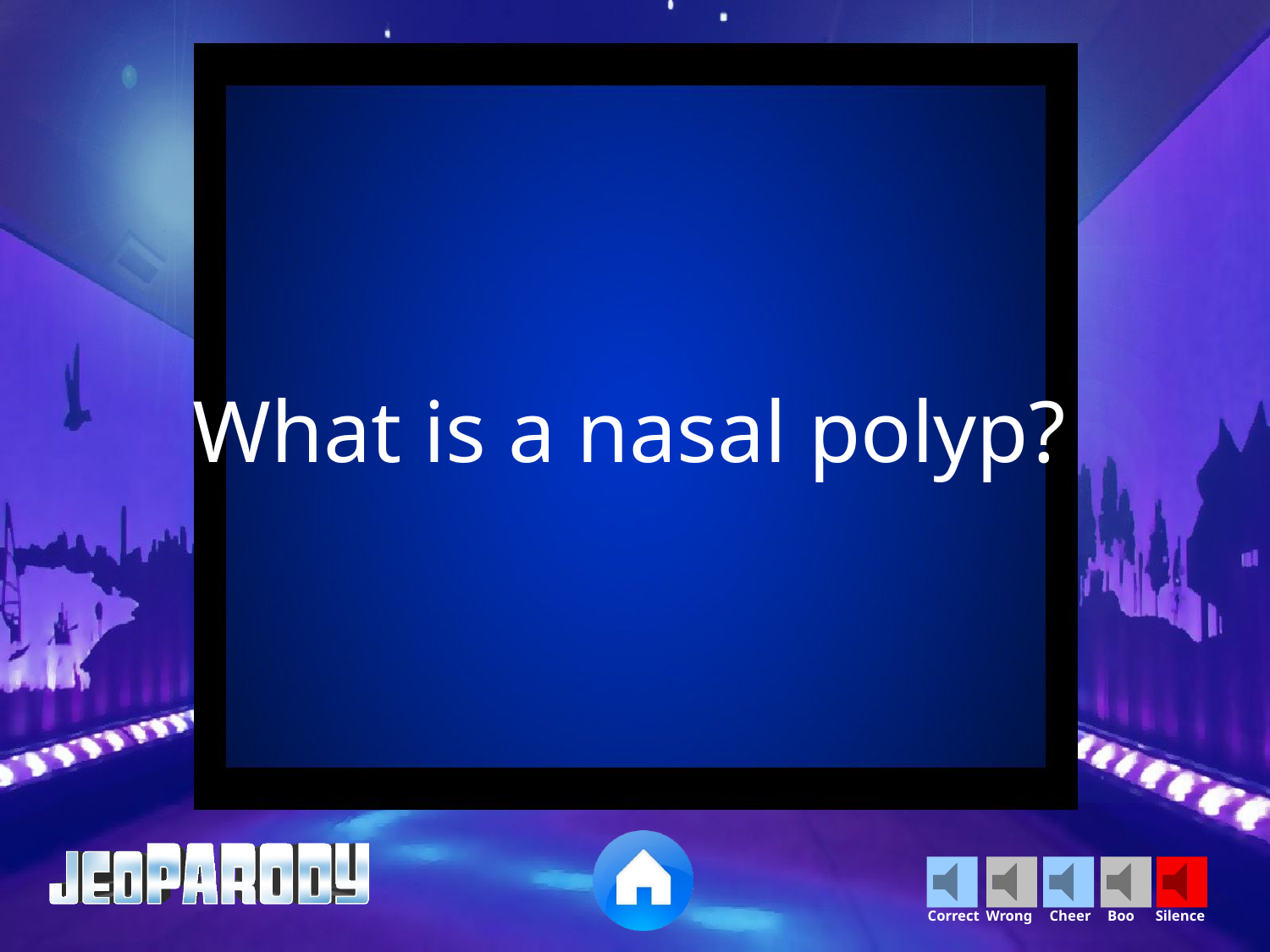

What is a nasal polyp?

## Slide 47
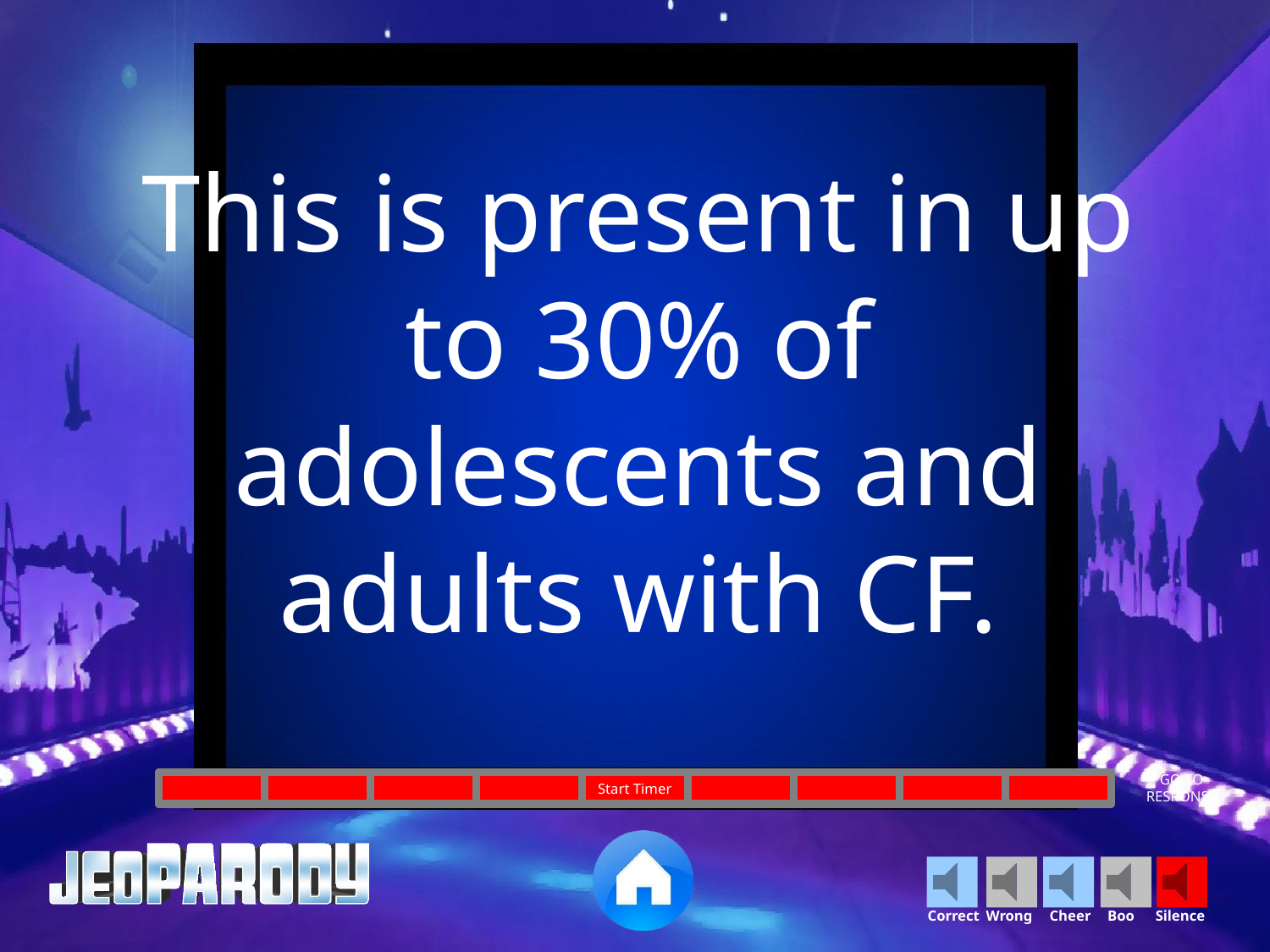

This is present in up to 30% of adolescents and adults with CF.

## Slide 48
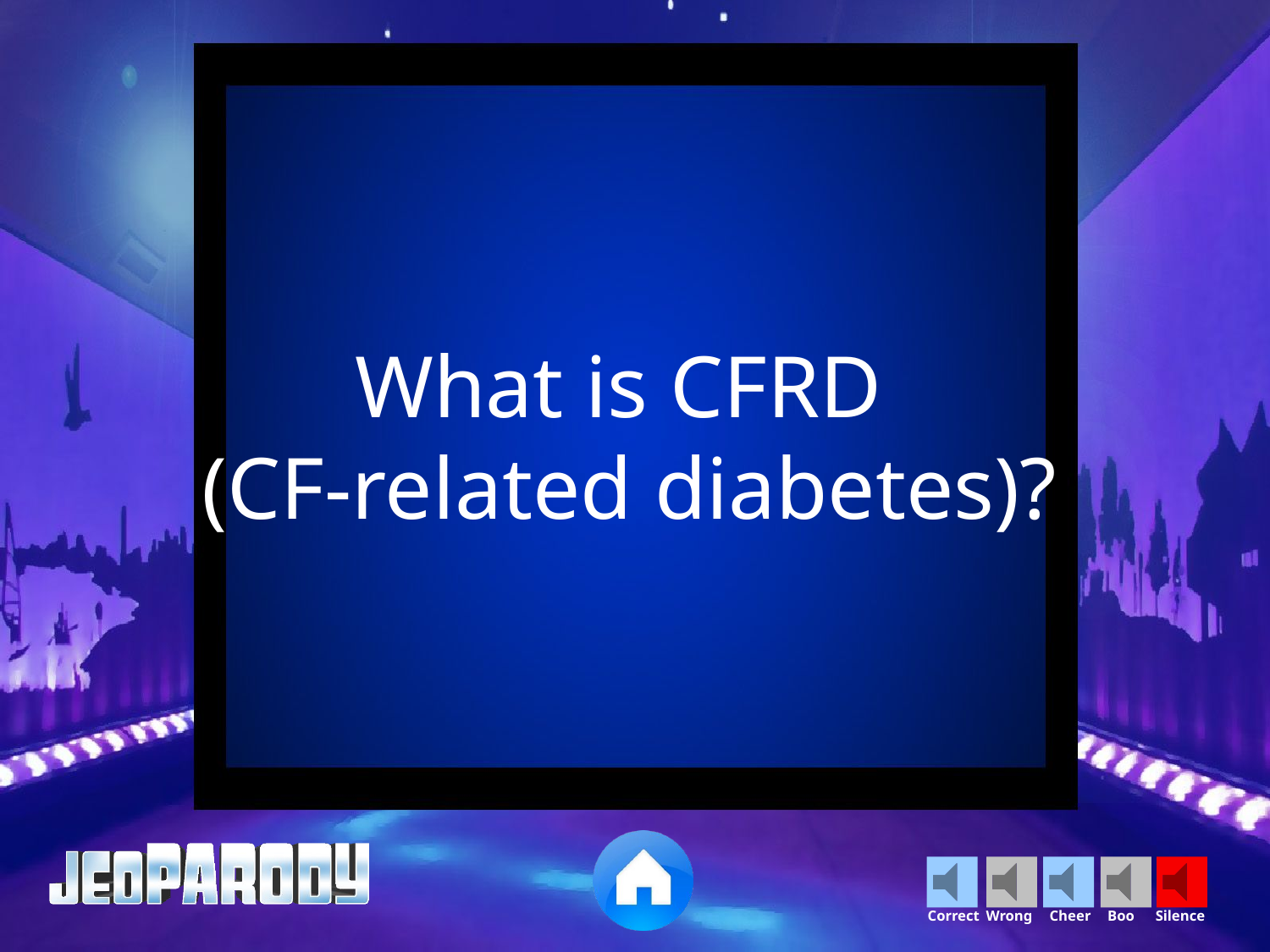

What is CFRD
(CF-related diabetes)?

## Slide 49
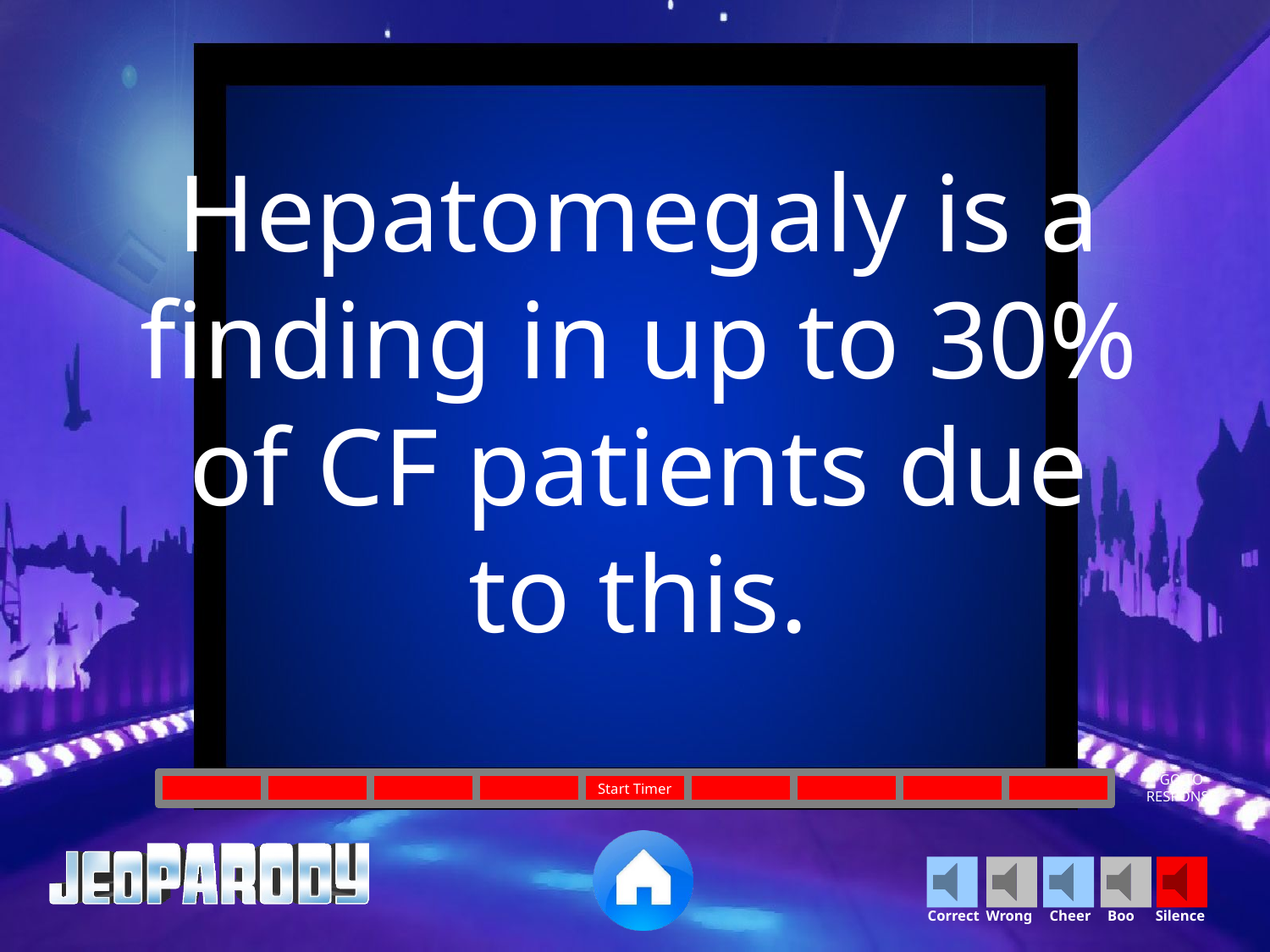

Hepatomegaly is a finding in up to 30% of CF patients due to this.

## Slide 50
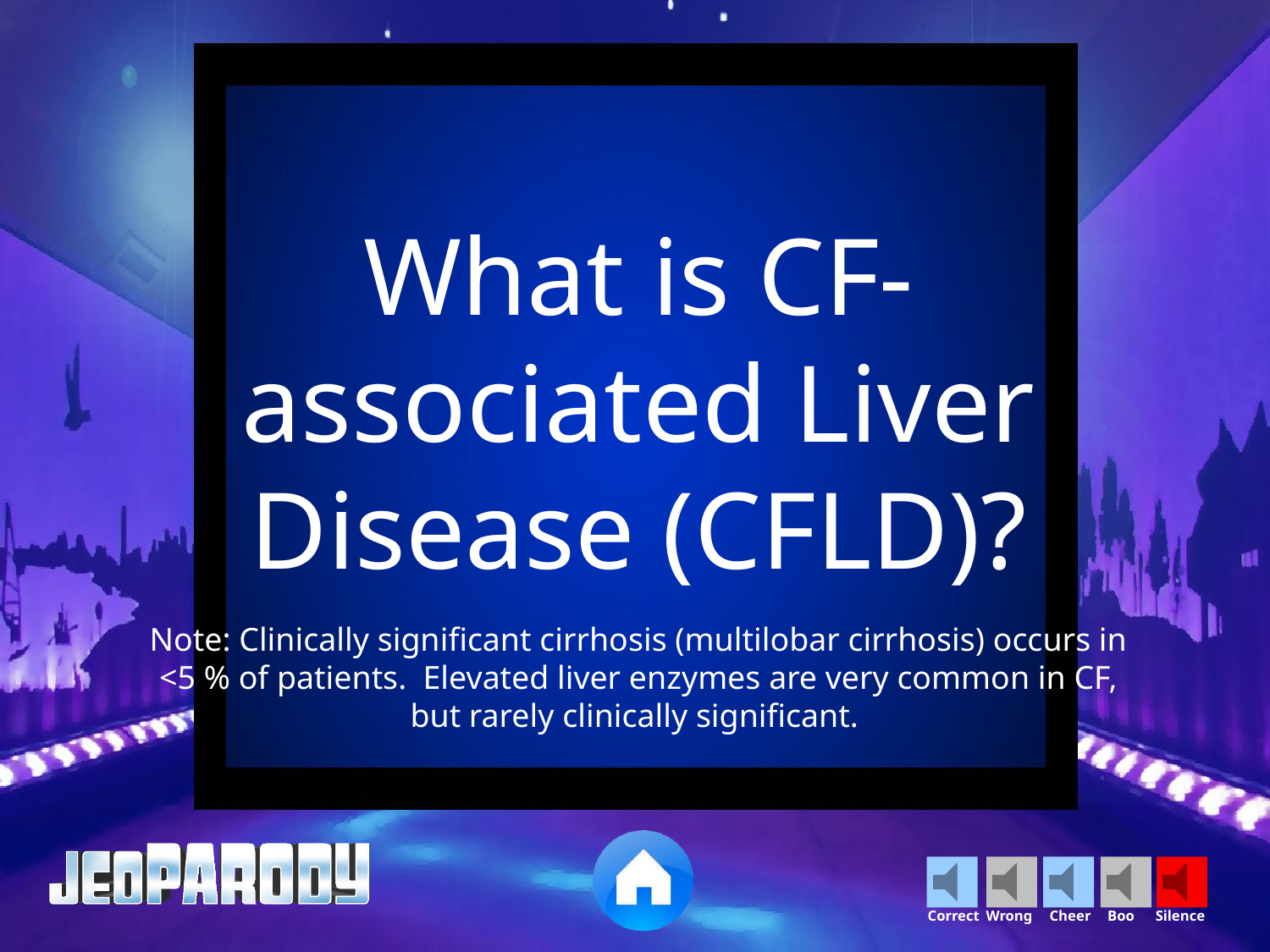

What is CF-associated Liver Disease (CFLD)?
Note: Clinically significant cirrhosis (multilobar cirrhosis) occurs in <5 % of patients. Elevated liver enzymes are very common in CF, but rarely clinically significant.

## Slide 51
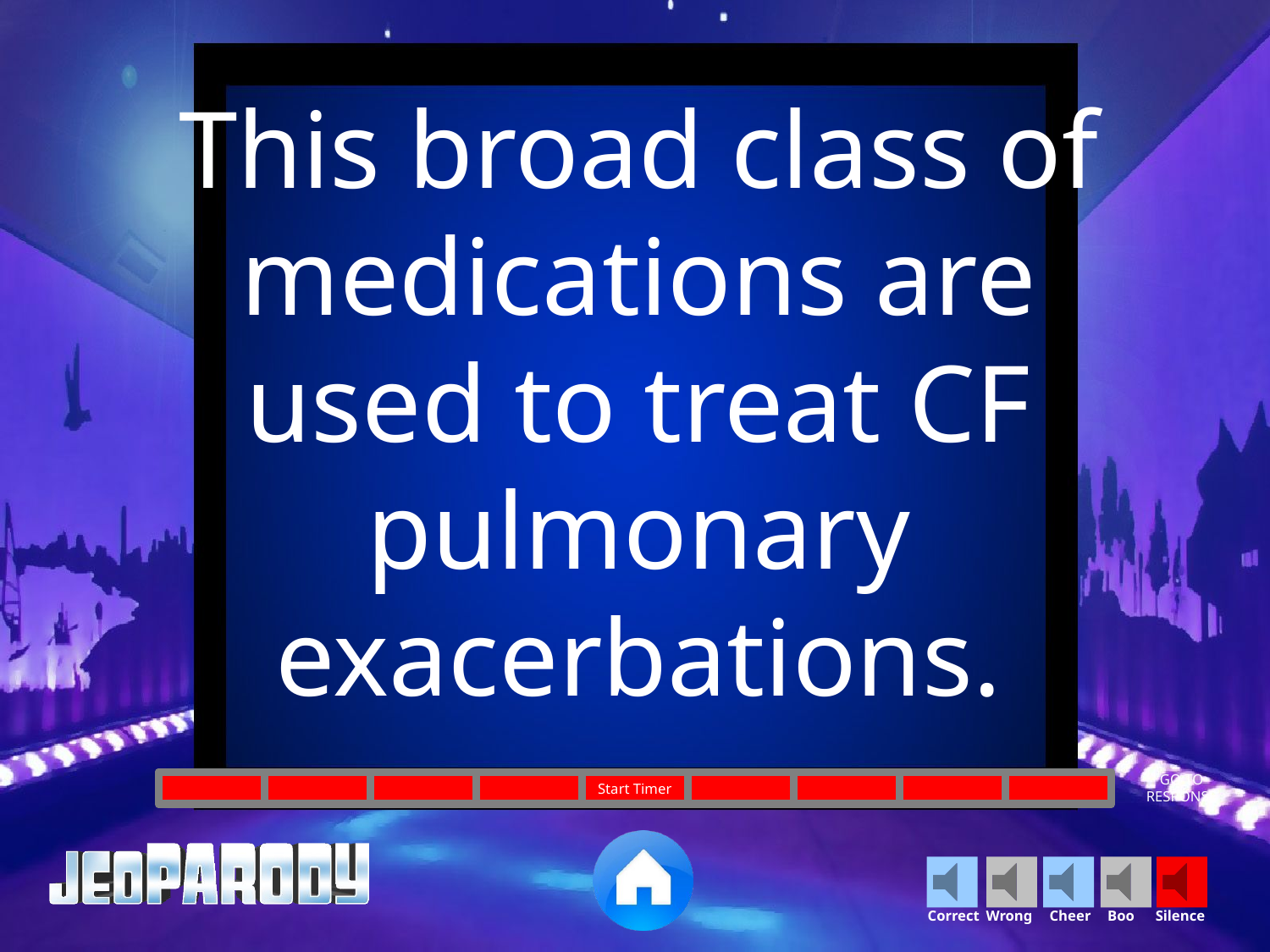

This broad class of medications are used to treat CF pulmonary exacerbations.

## Slide 52
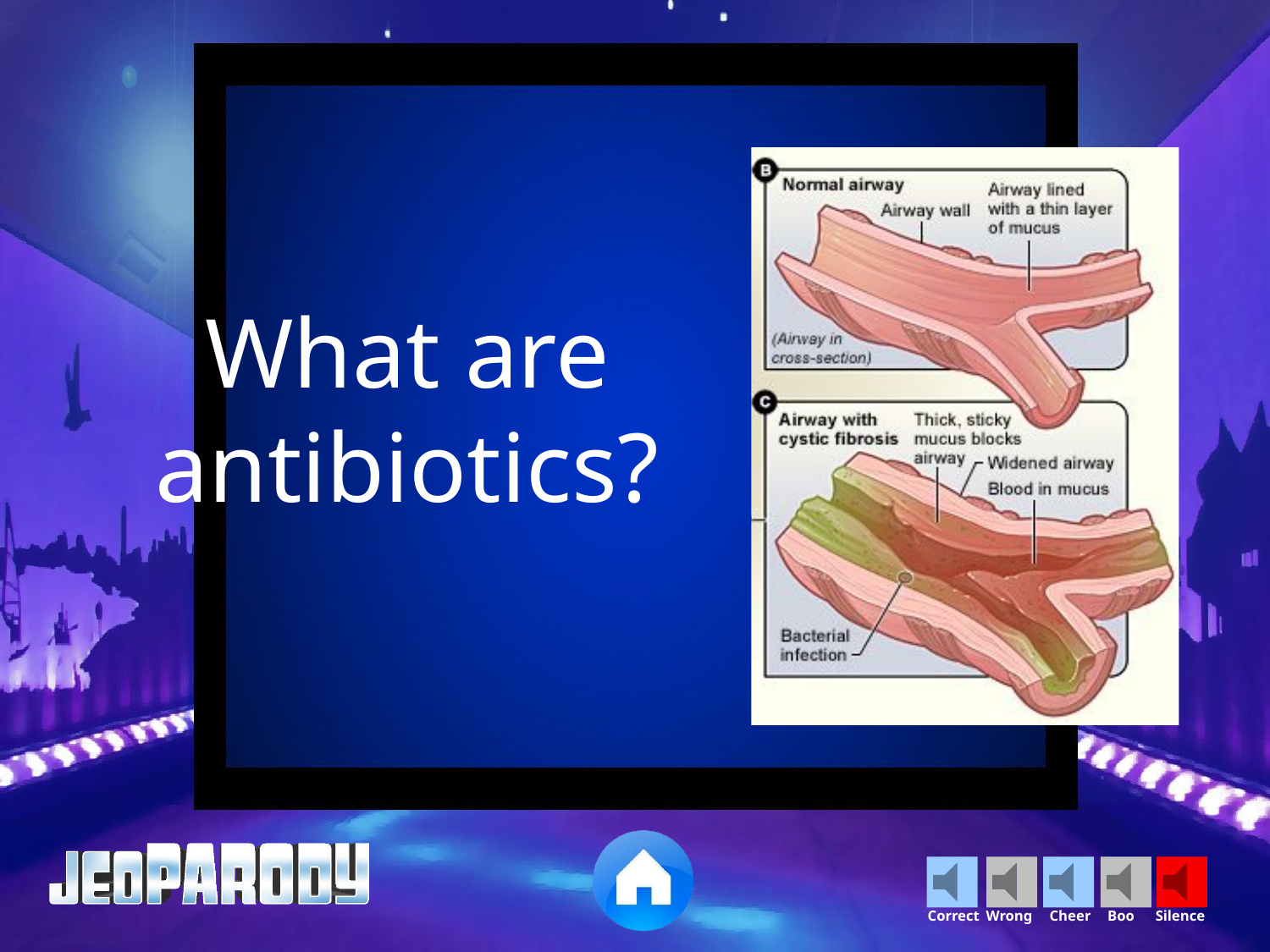

What are antibiotics?

## Slide 53
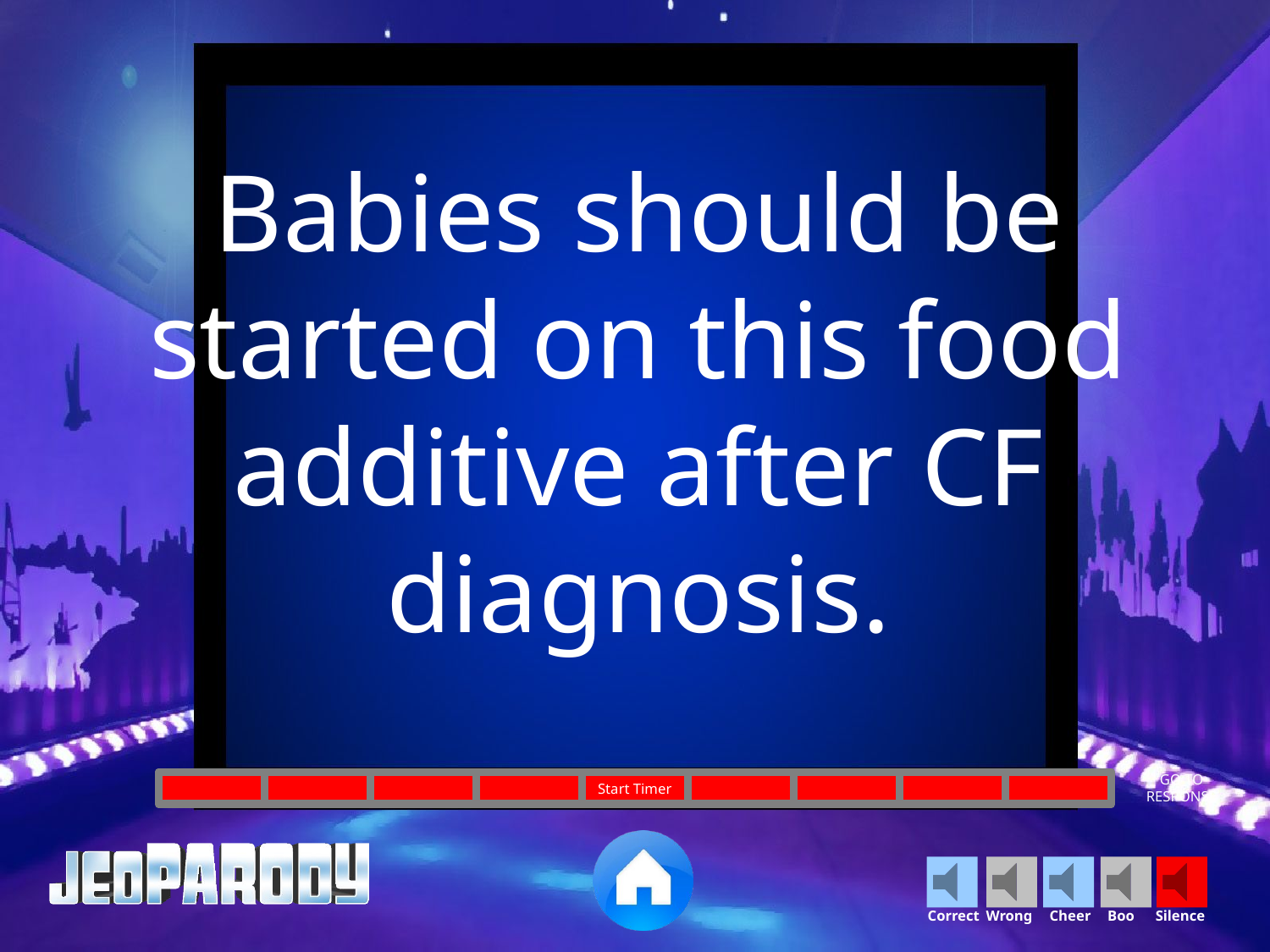

Babies should be started on this food additive after CF diagnosis.

## Slide 54
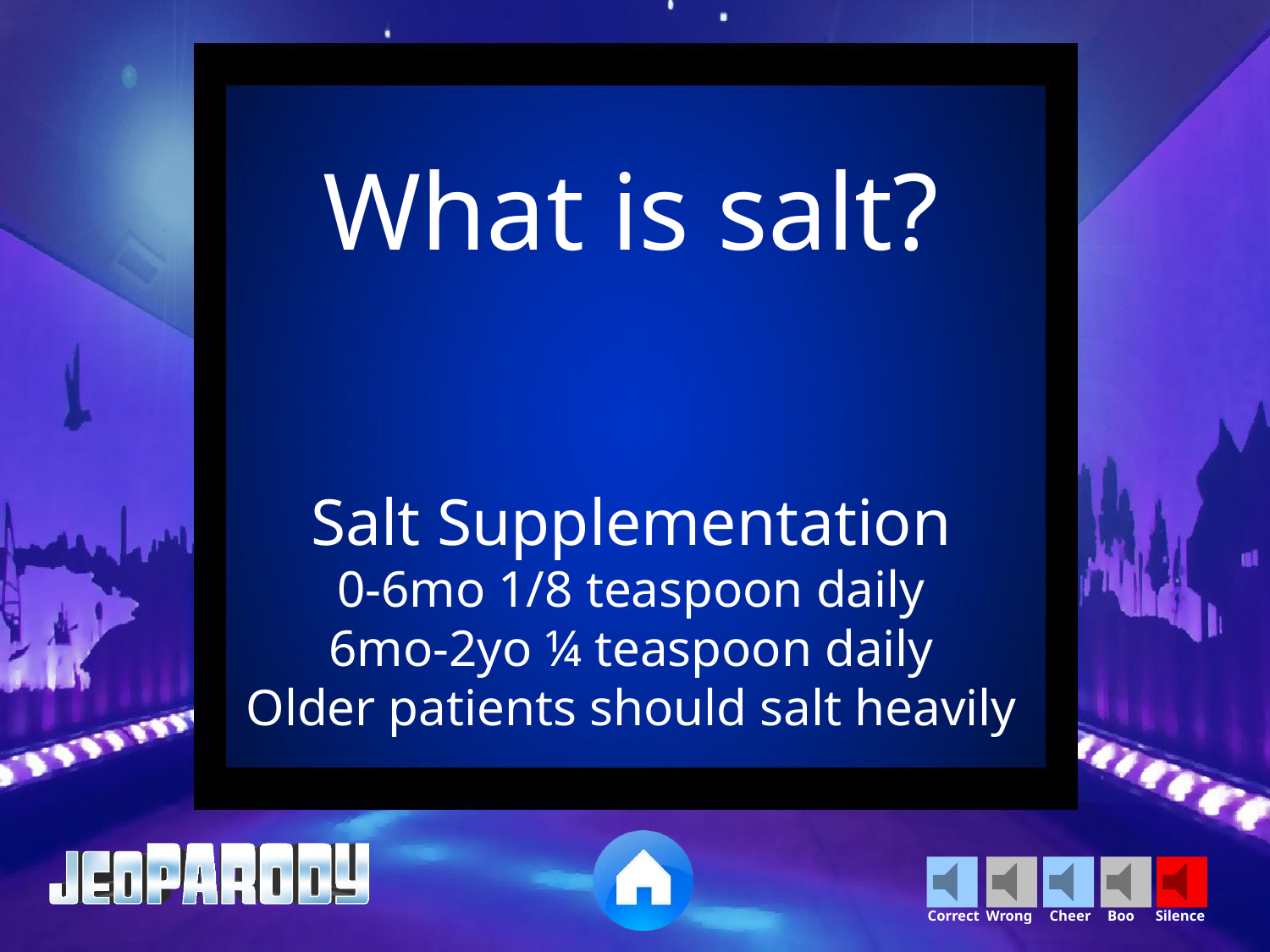

What is salt?
Salt Supplementation
0-6mo 1/8 teaspoon daily
6mo-2yo ¼ teaspoon daily
Older patients should salt heavily

## Slide 55
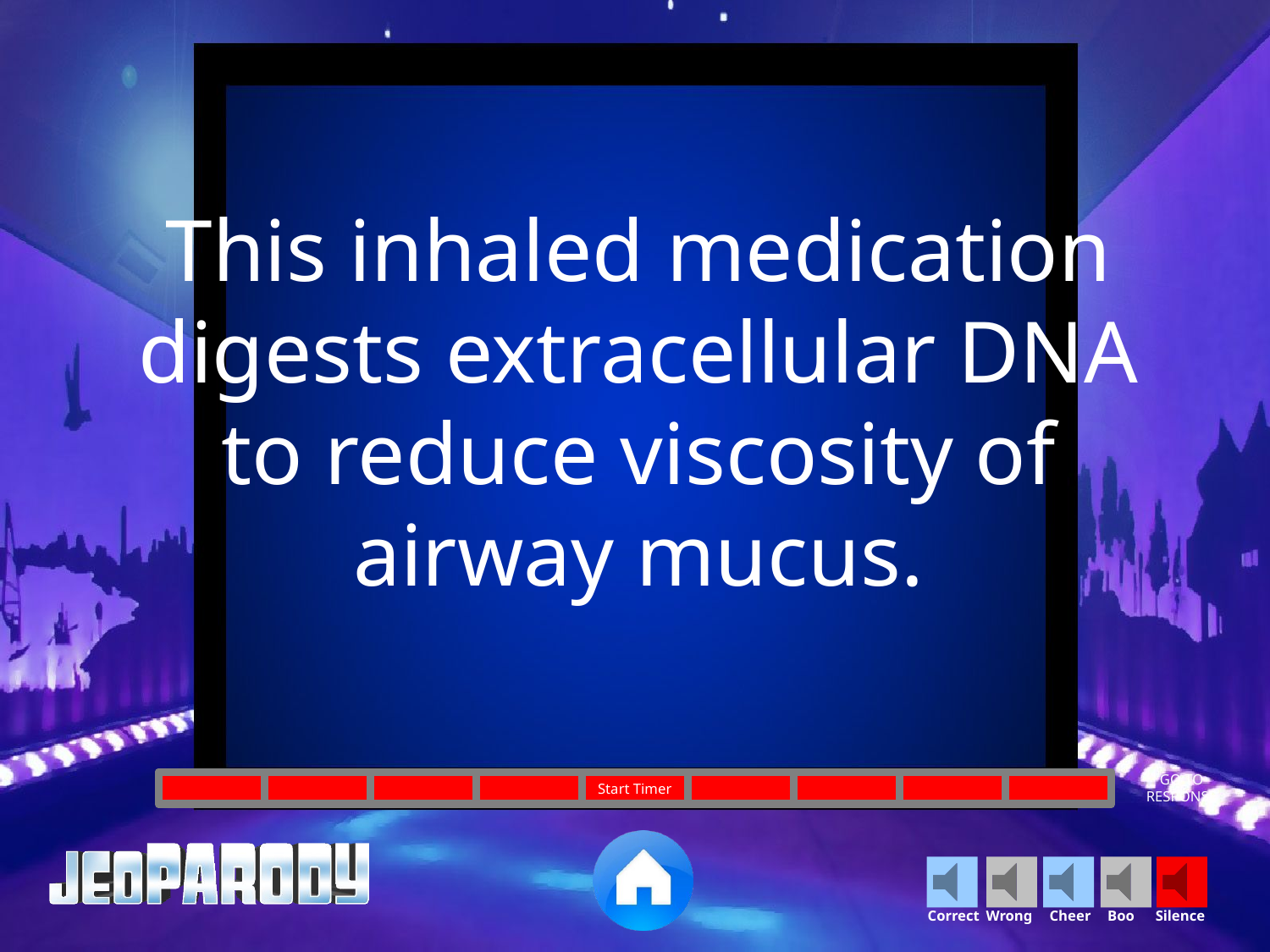

This inhaled medication digests extracellular DNA to reduce viscosity of airway mucus.

## Slide 56
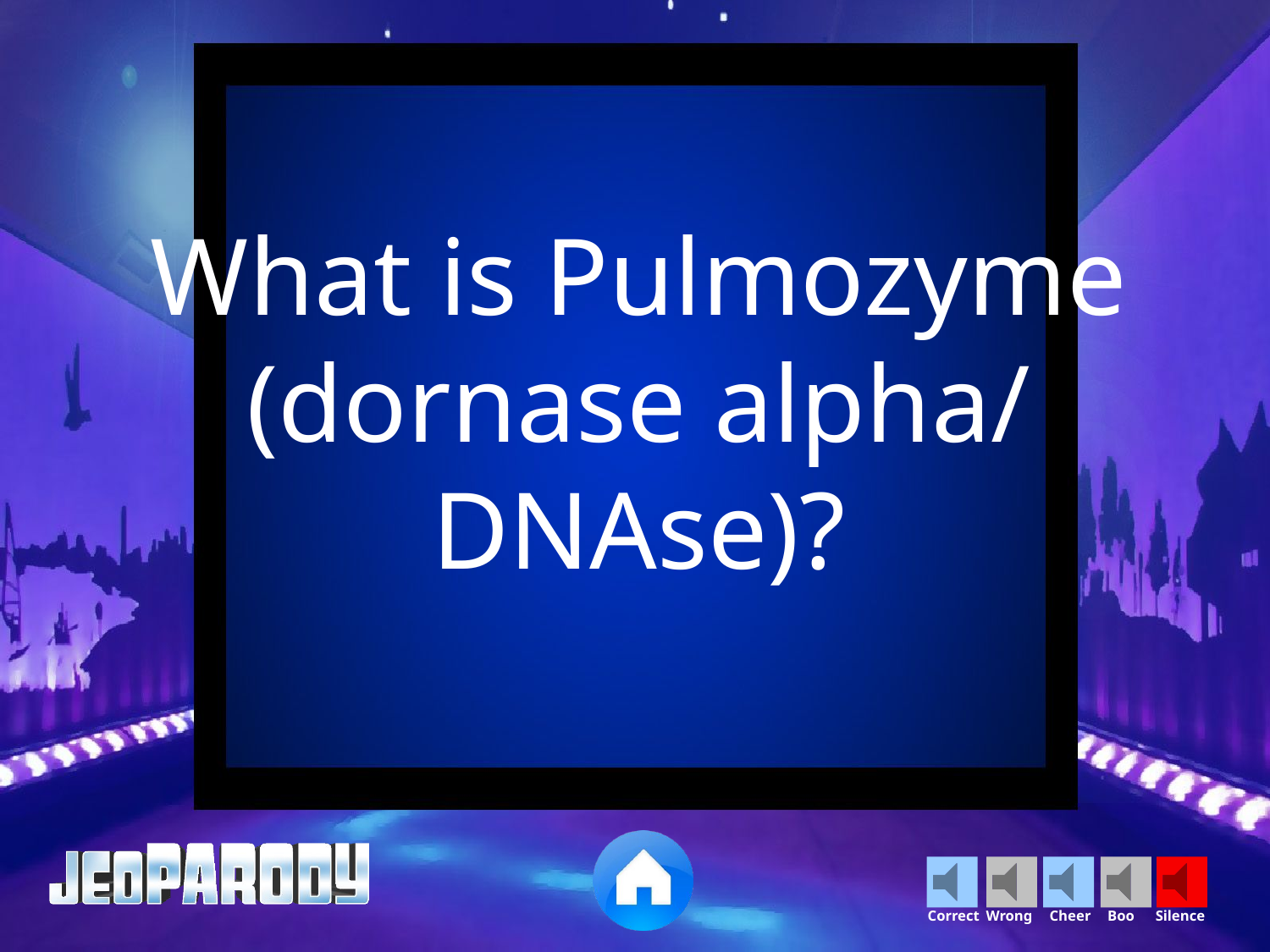

What is Pulmozyme (dornase alpha/ DNAse)?

## Slide 57
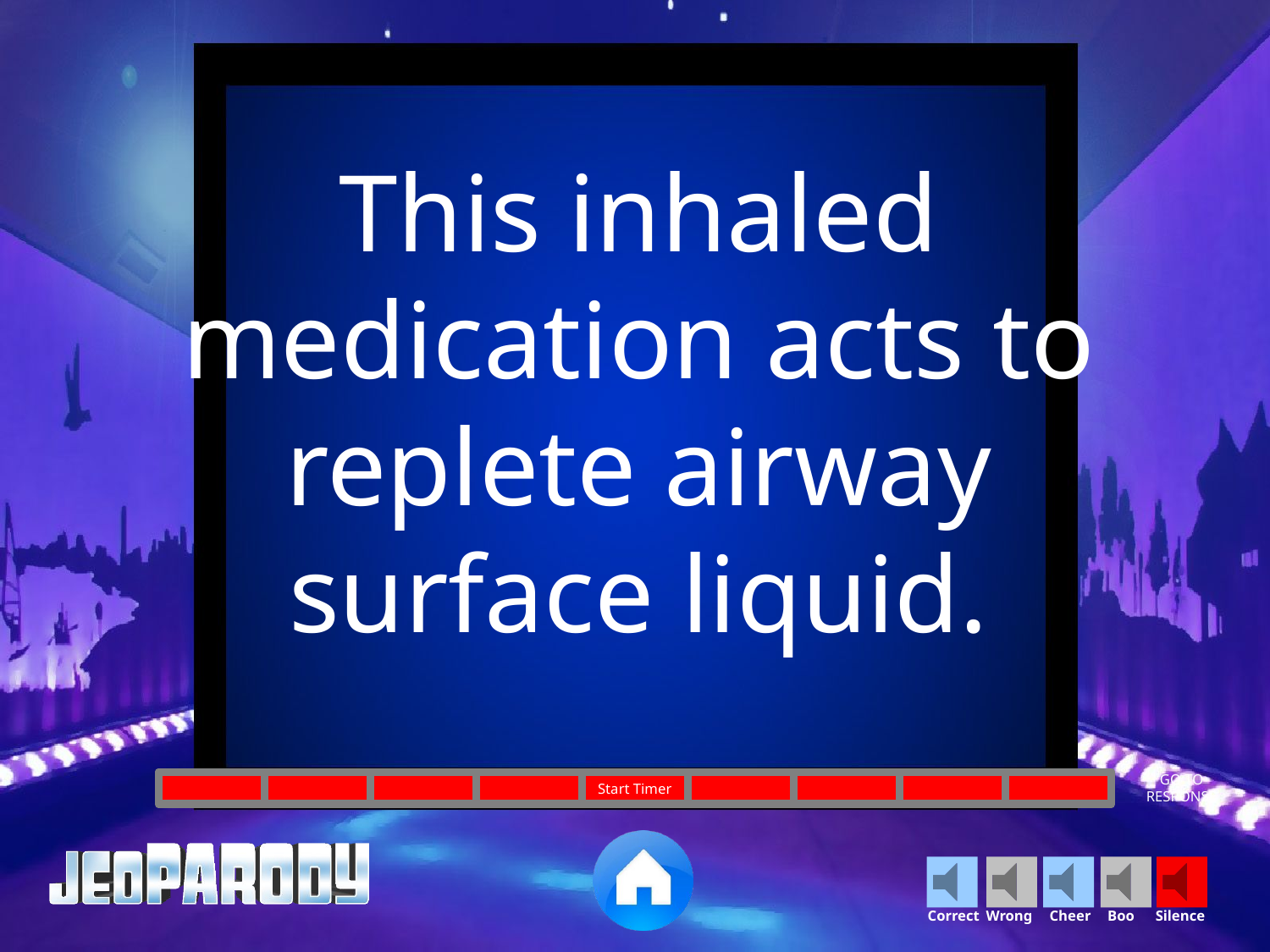

This inhaled medication acts to replete airway surface liquid.

## Slide 58
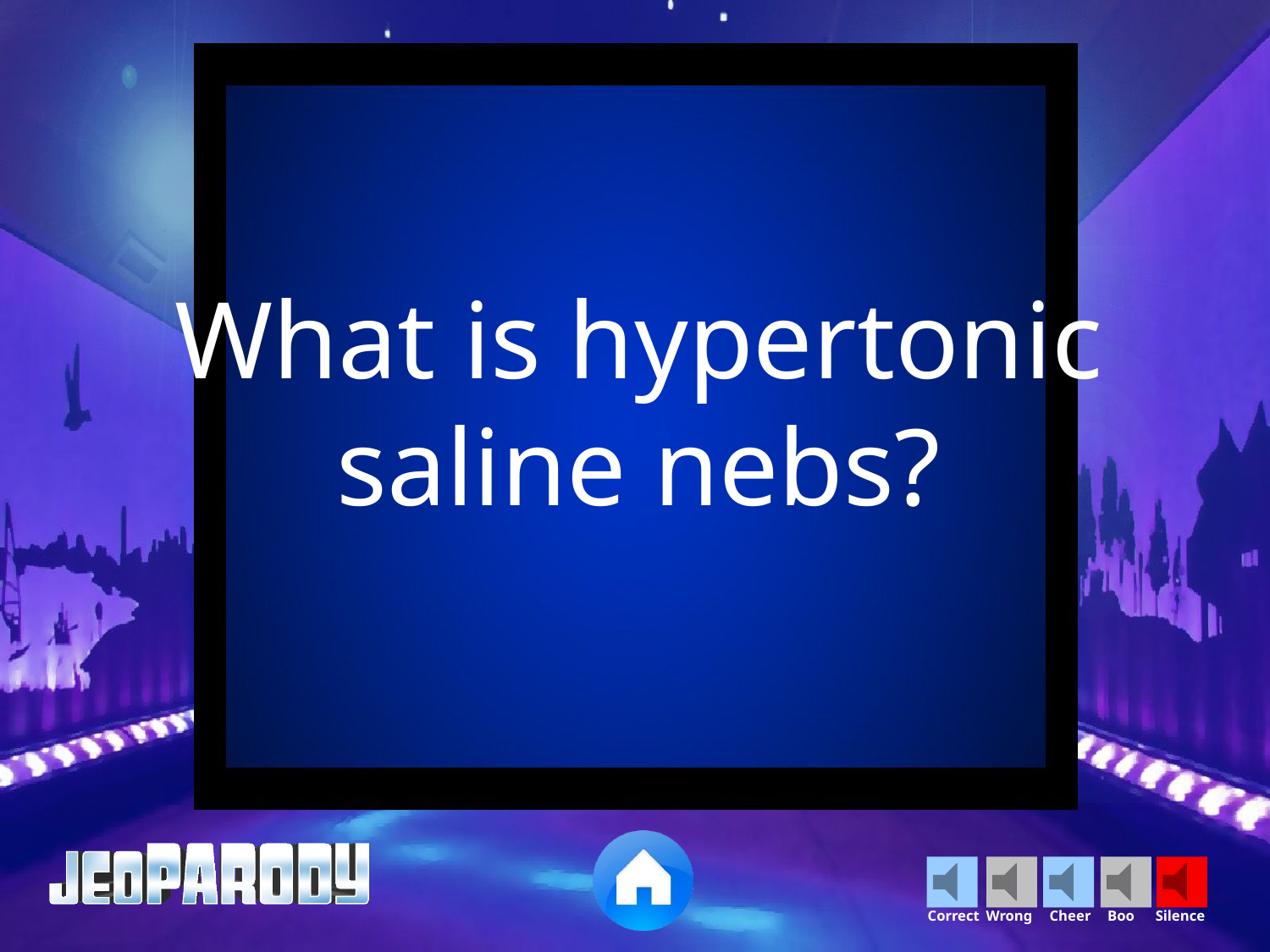

What is hypertonic saline nebs?

## Slide 59
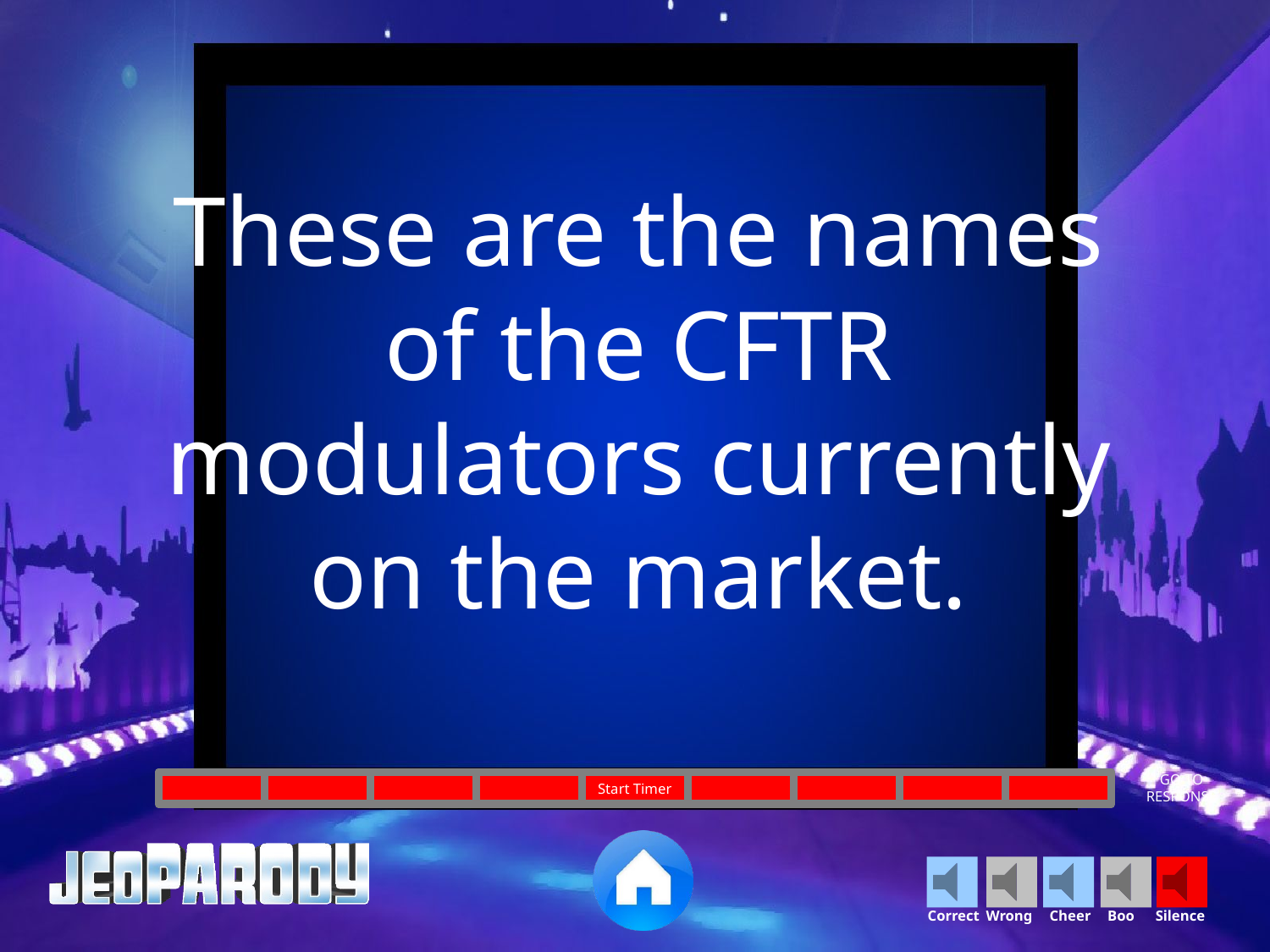

These are the names of the CFTR modulators currently on the market.

## Slide 60
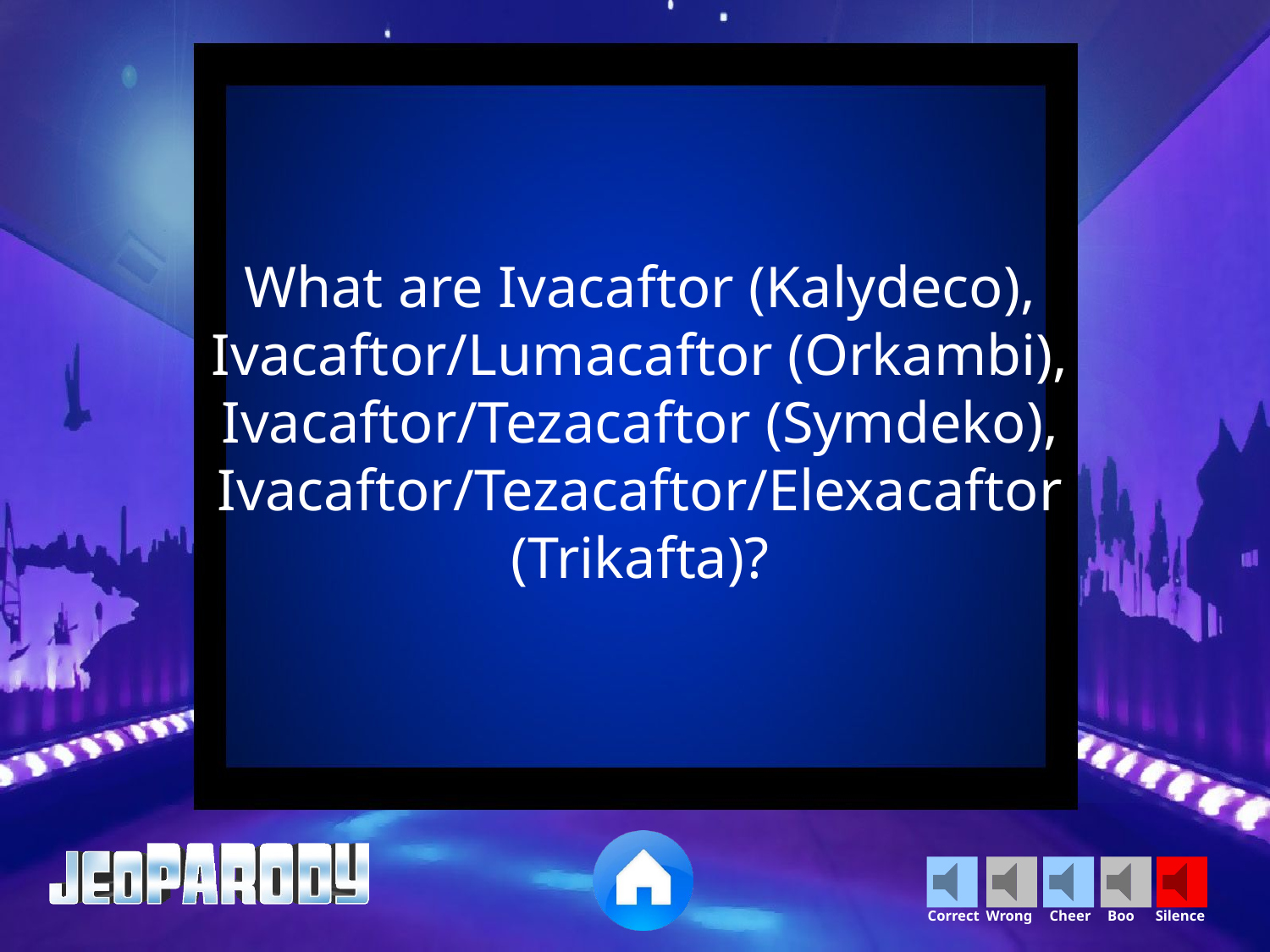

What are Ivacaftor (Kalydeco), Ivacaftor/Lumacaftor (Orkambi), Ivacaftor/Tezacaftor (Symdeko), Ivacaftor/Tezacaftor/Elexacaftor (Trikafta)?

## Slide 61
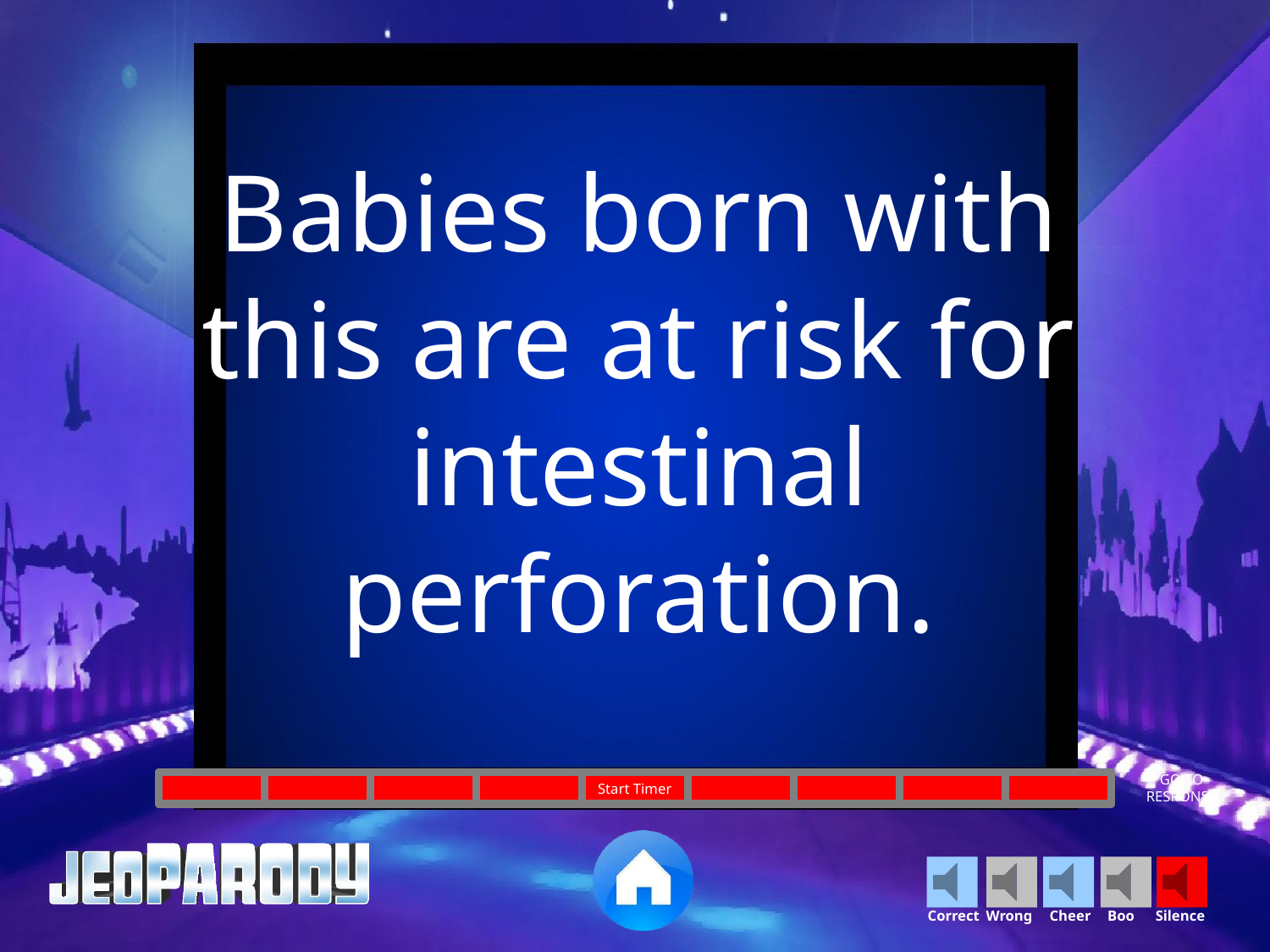

Babies born with this are at risk for intestinal perforation.

## Slide 62
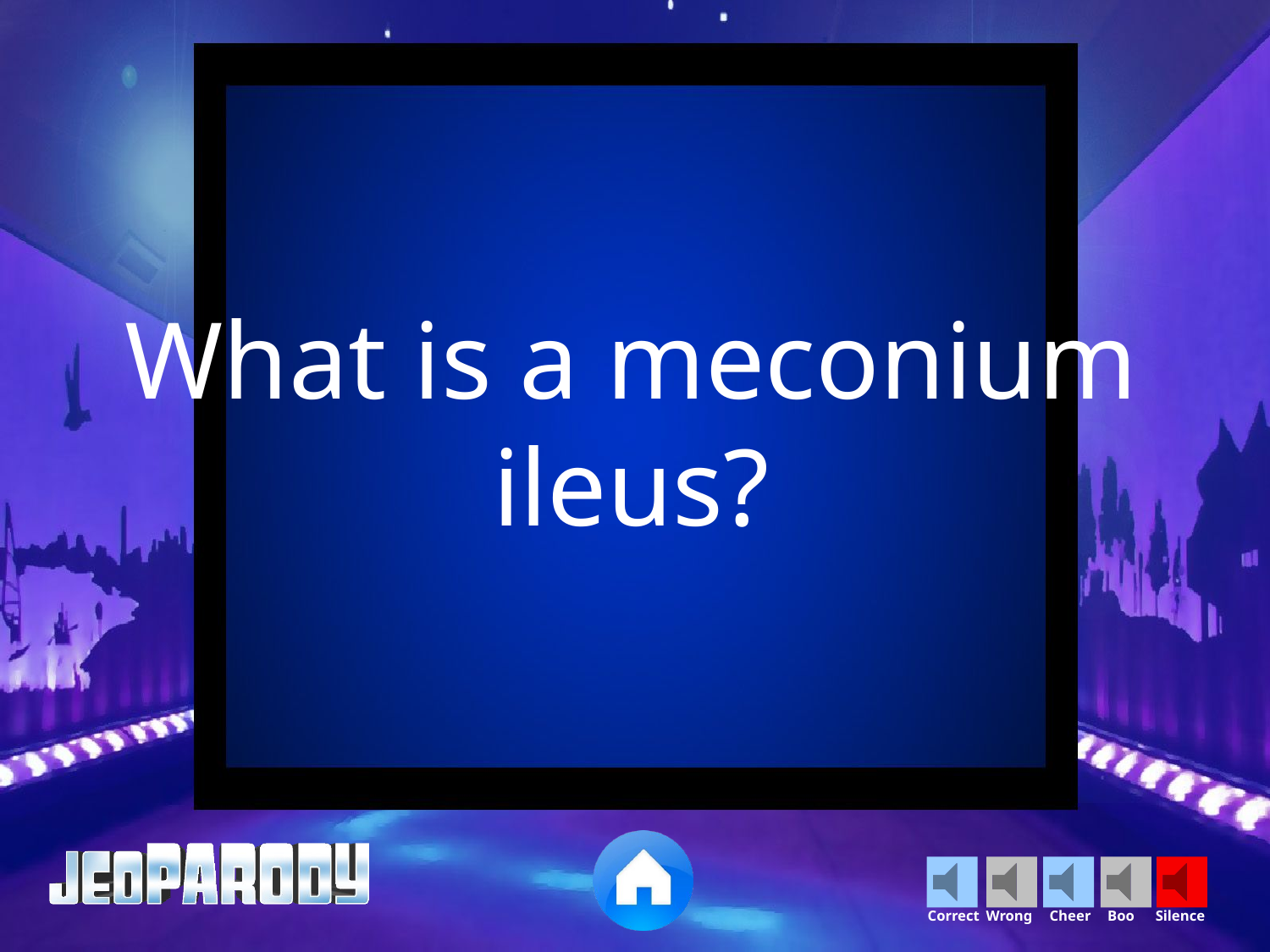

What is a meconium ileus?

## Slide 63
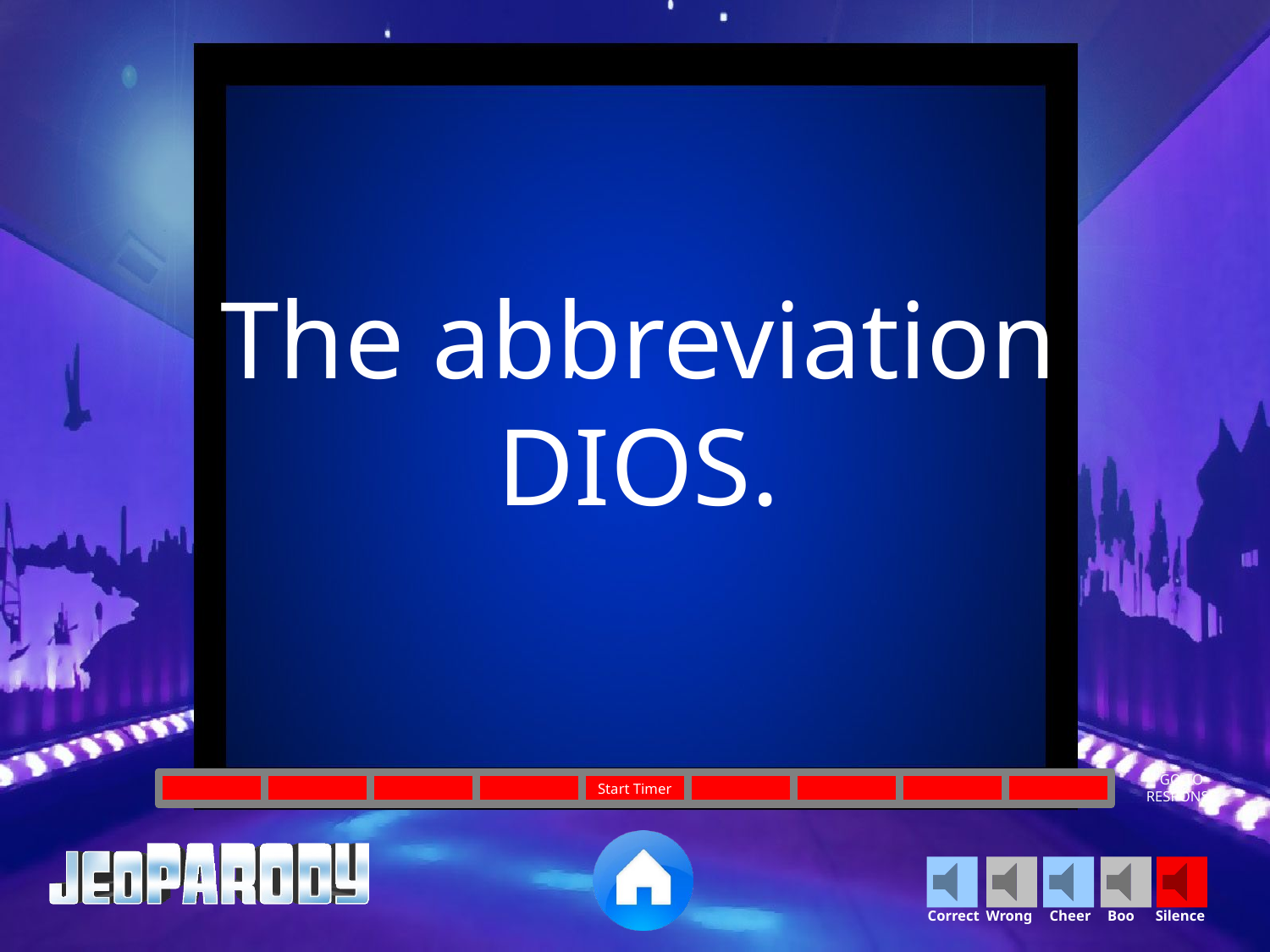

The abbreviation DIOS.

## Slide 64
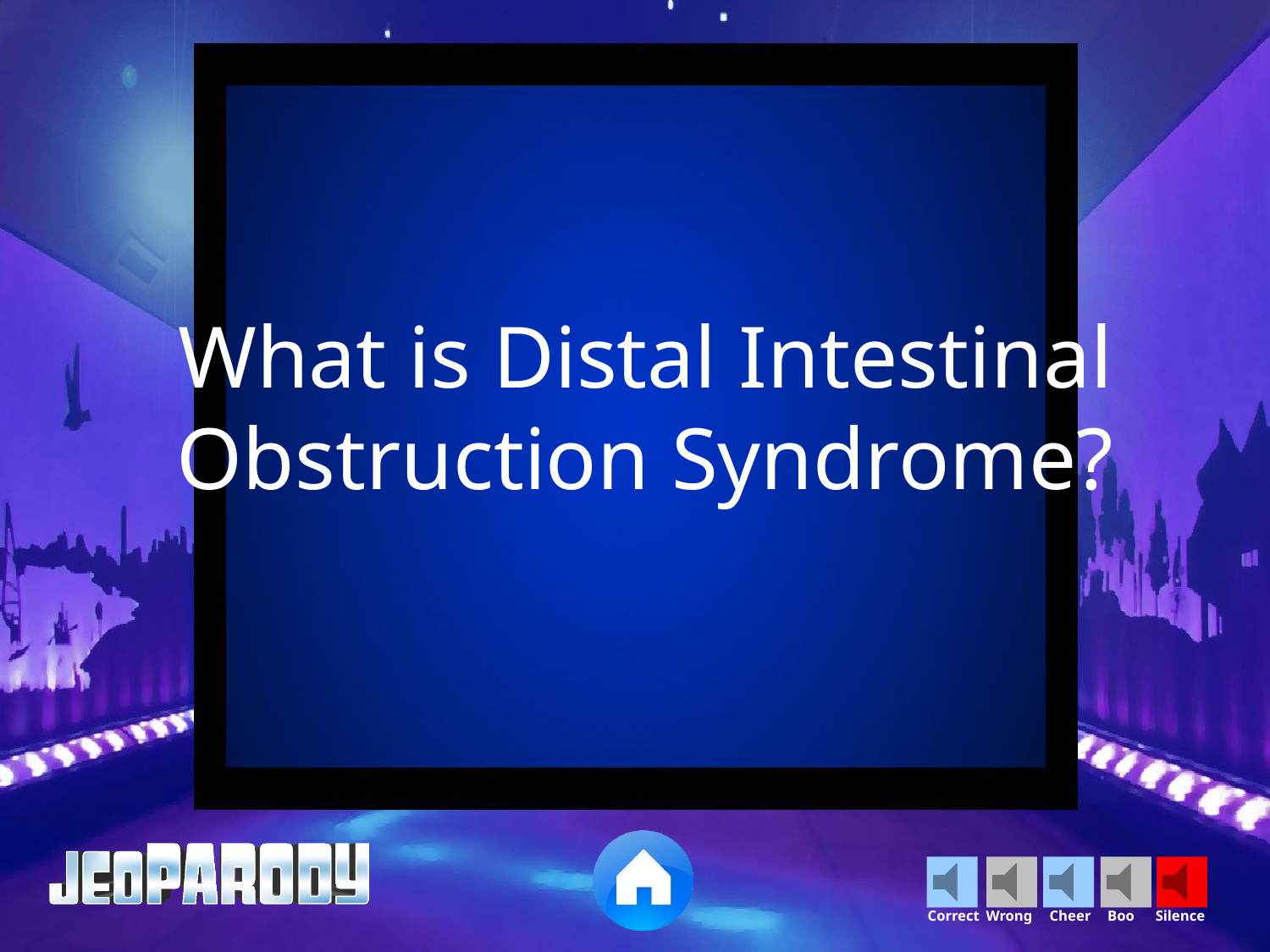

What is Distal Intestinal Obstruction Syndrome?

## Slide 65
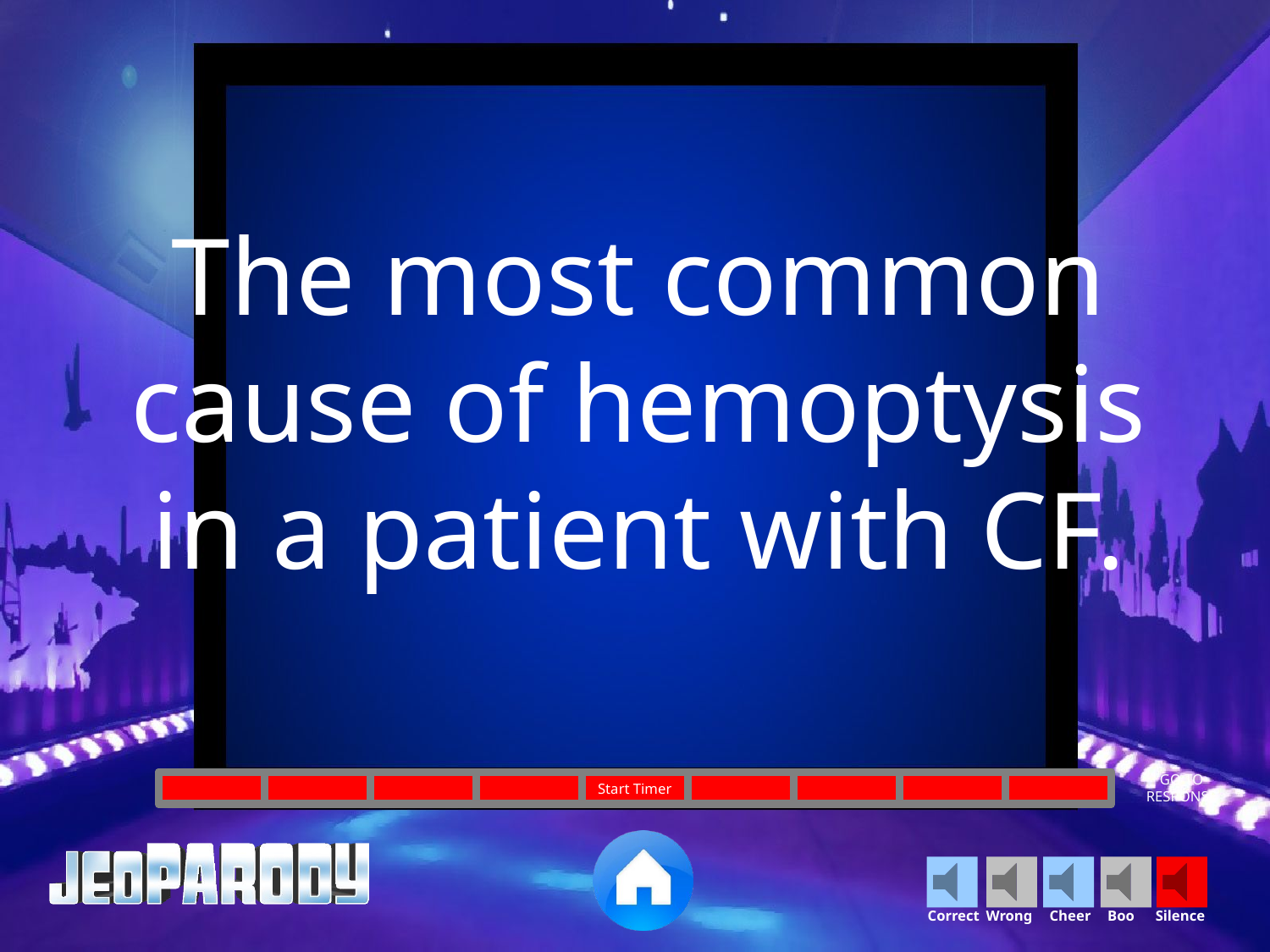

The most common cause of hemoptysis in a patient with CF.

## Slide 66
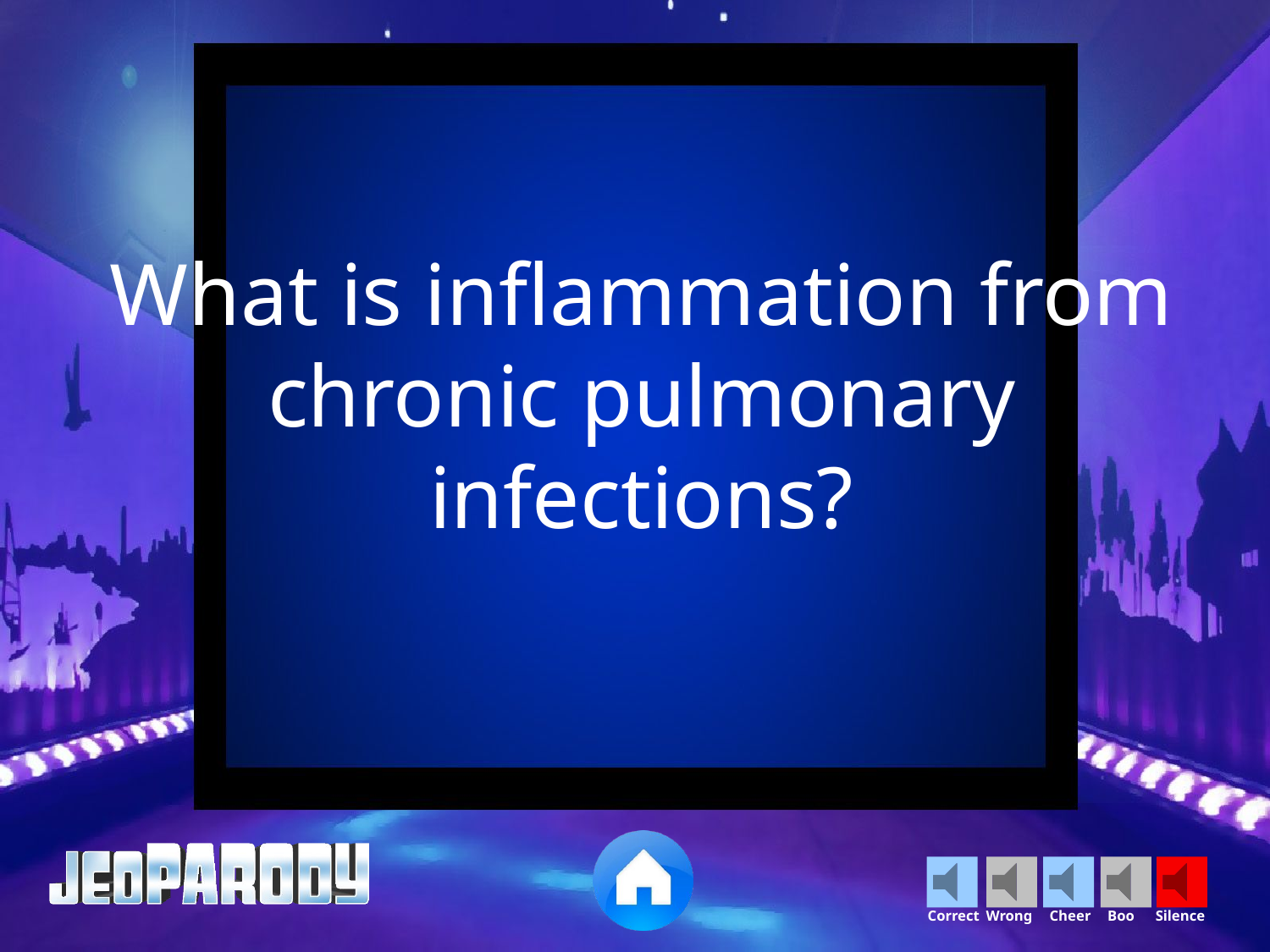

What is inflammation from chronic pulmonary infections?

## Slide 67
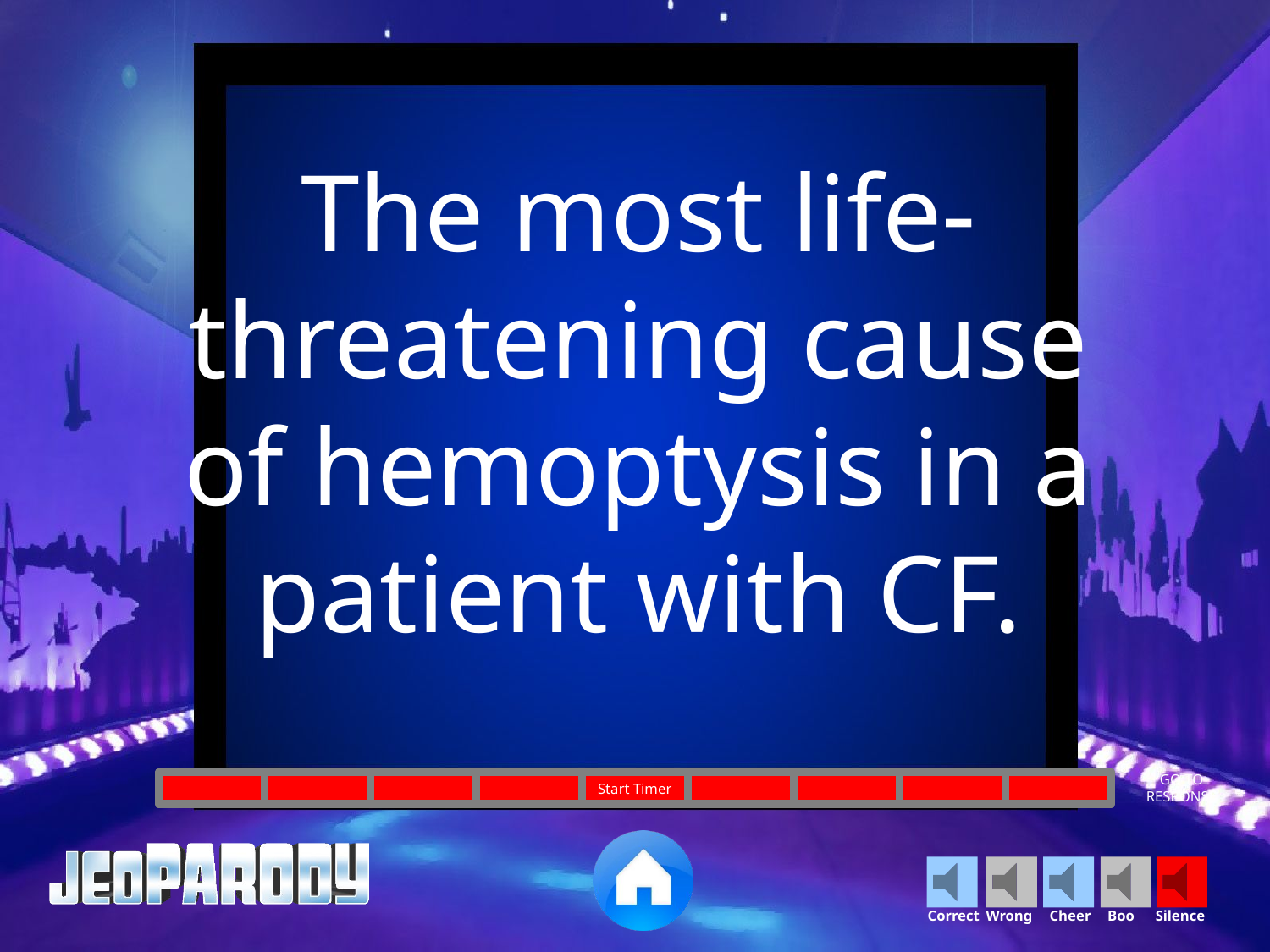

The most life-threatening cause of hemoptysis in a patient with CF.

## Slide 68
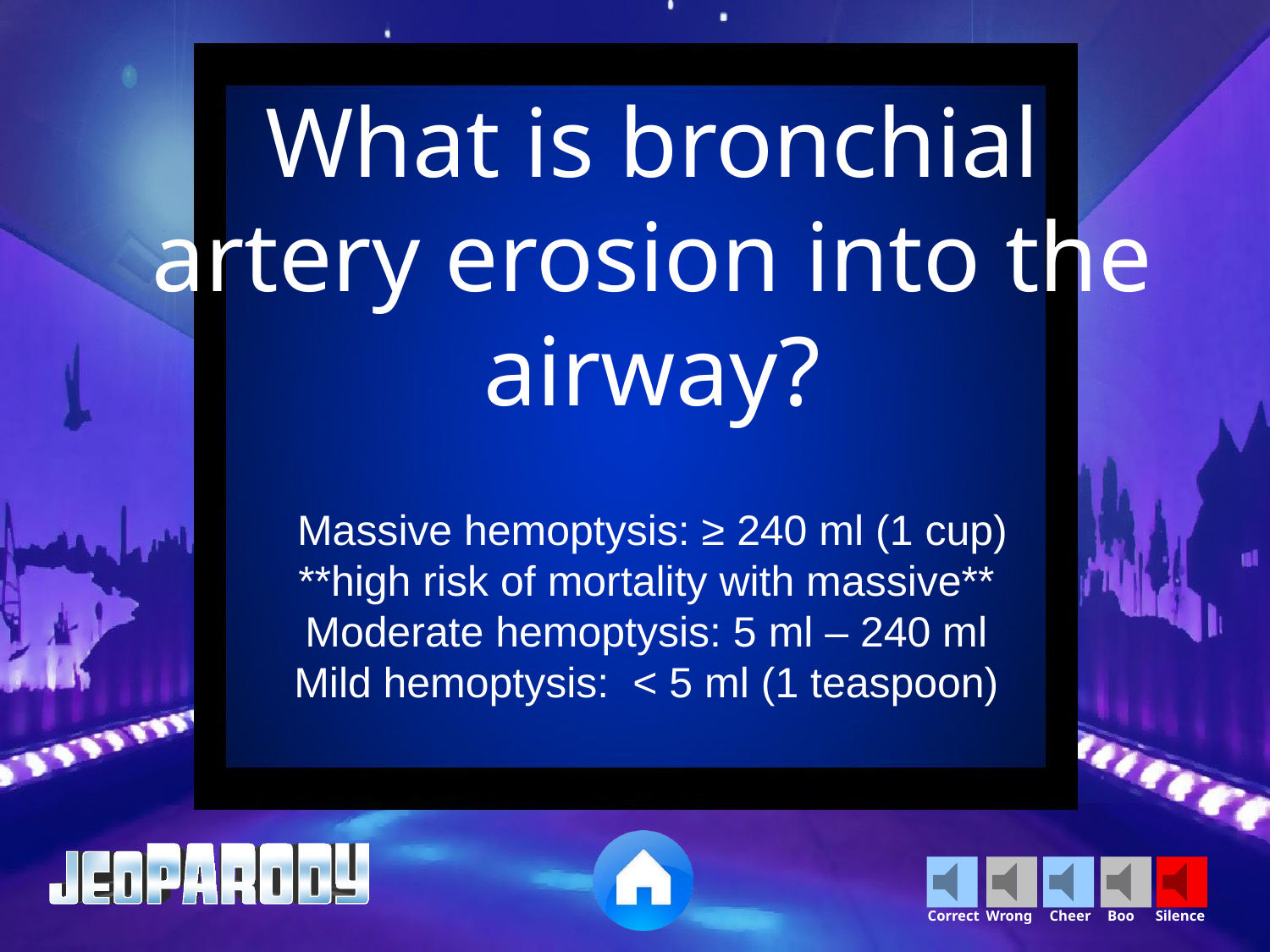

What is bronchial artery erosion into the airway?
Massive hemoptysis: ≥ 240 ml (1 cup)
**high risk of mortality with massive**
Moderate hemoptysis: 5 ml – 240 ml
Mild hemoptysis: < 5 ml (1 teaspoon)

## Slide 69
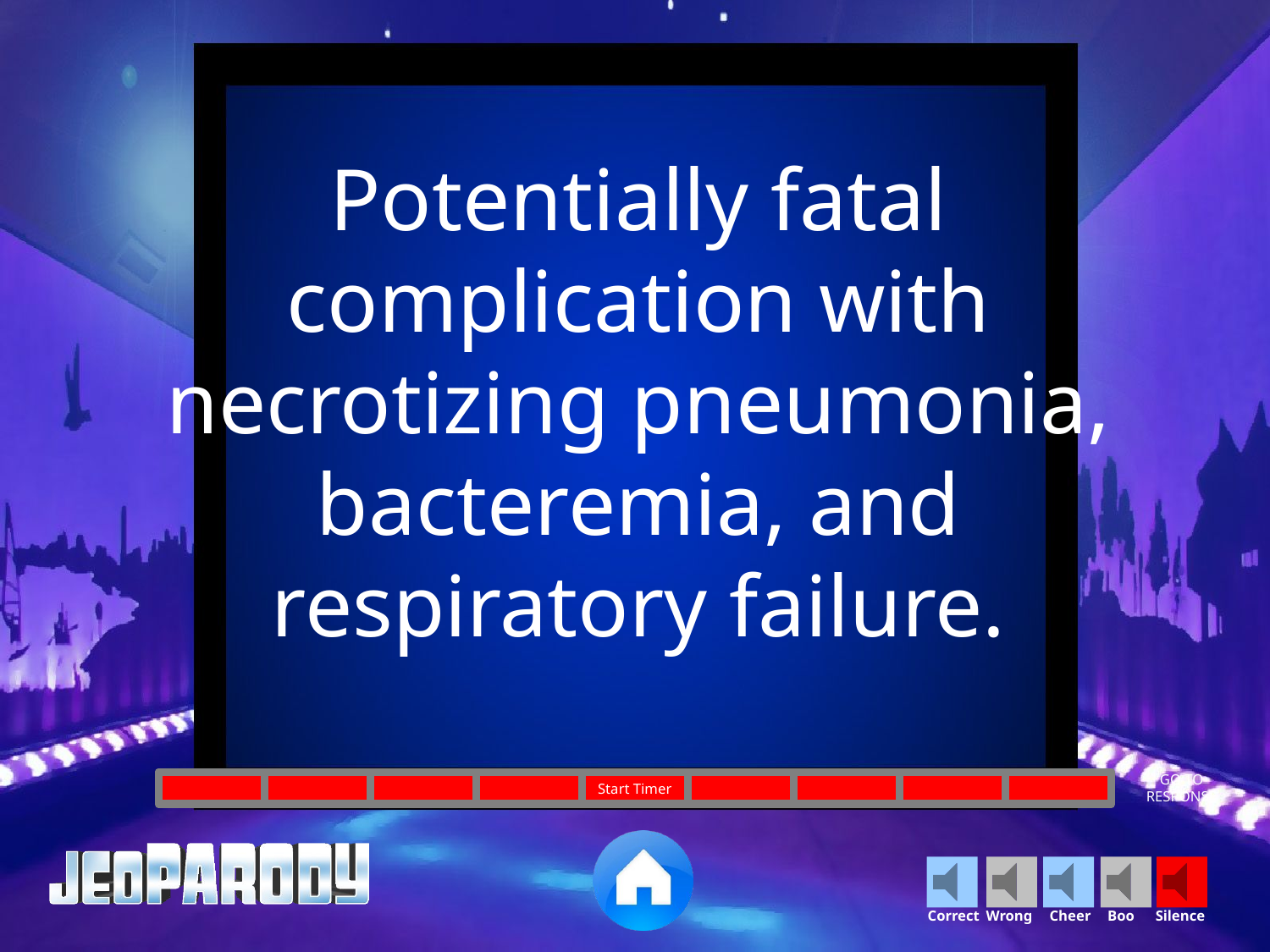

Potentially fatal complication with necrotizing pneumonia, bacteremia, and respiratory failure.

## Slide 70
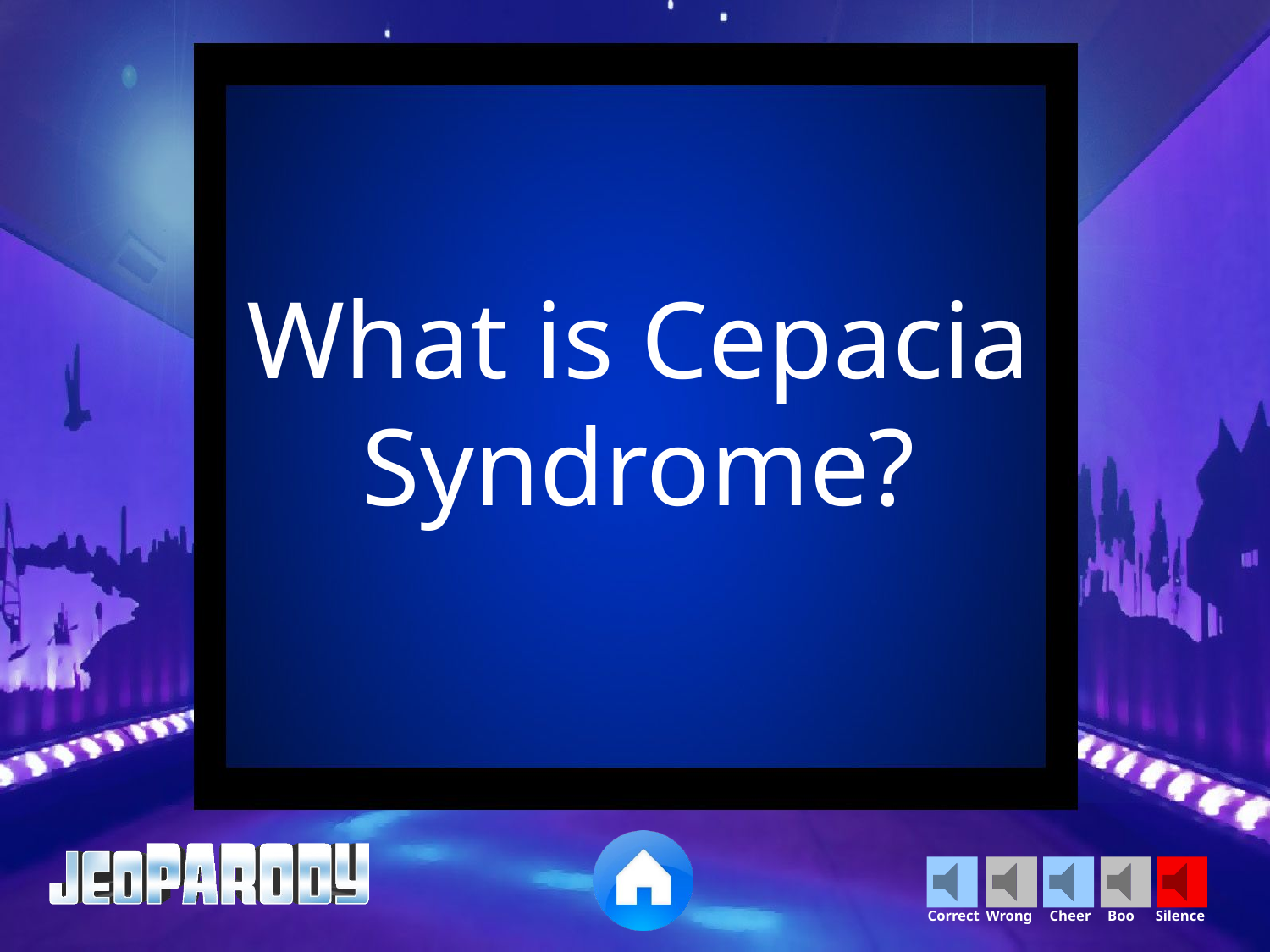

What is Cepacia Syndrome?

## Slide 71
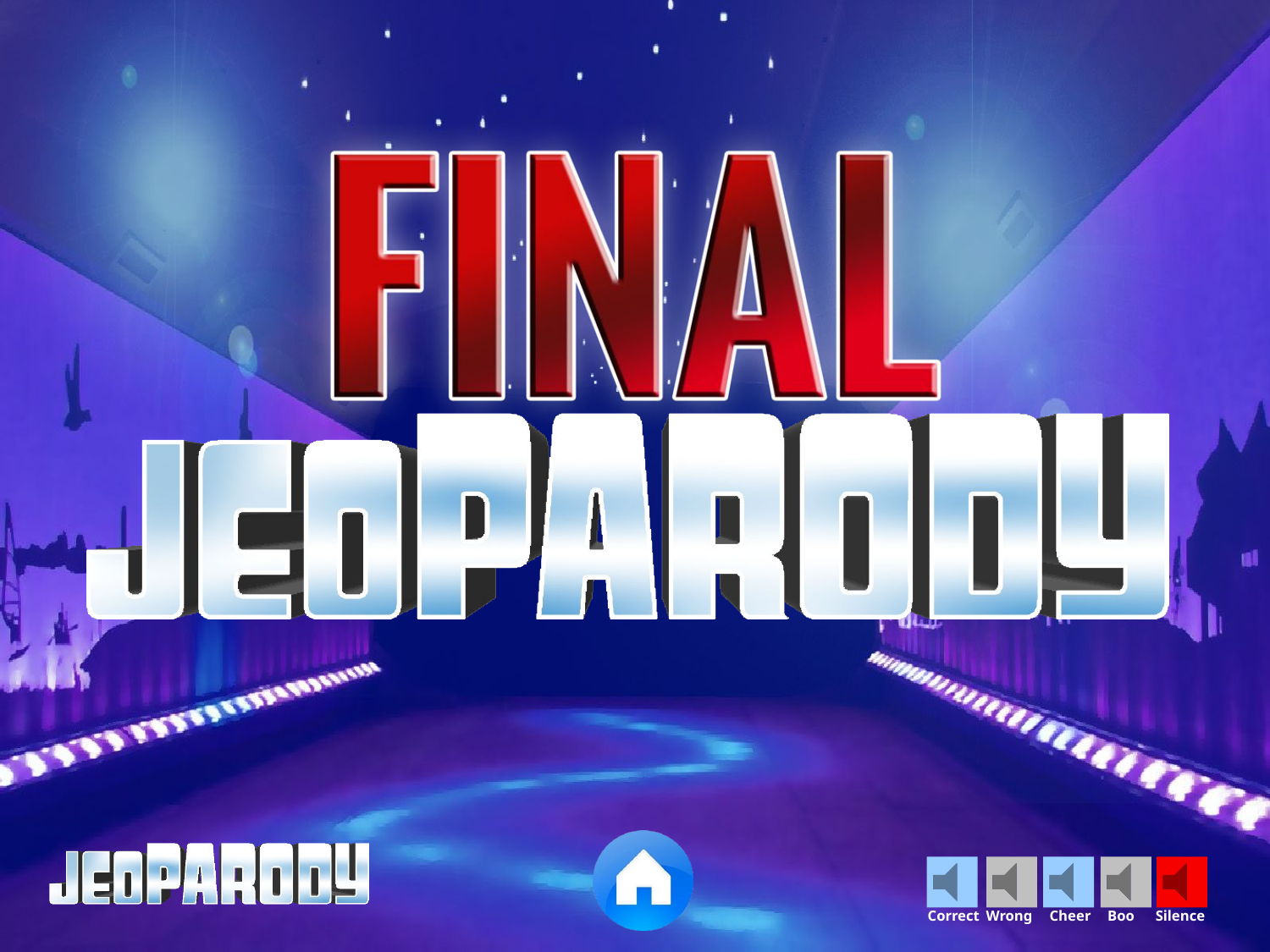

## Slide 72
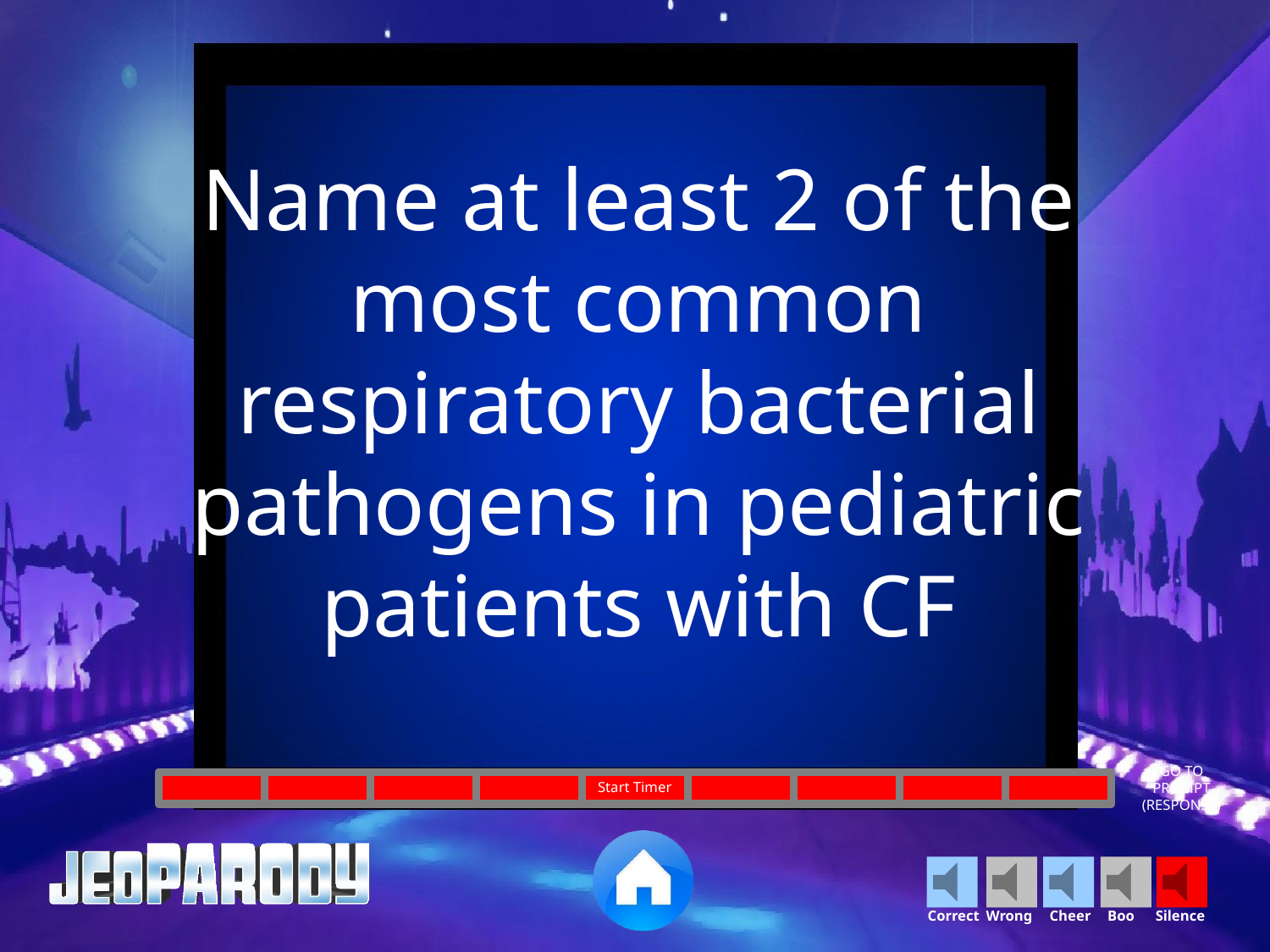

Name at least 2 of the most common respiratory bacterial pathogens in pediatric patients with CF
GO TO
PROMPT
(RESPONSE)
Start Timer

## Slide 73
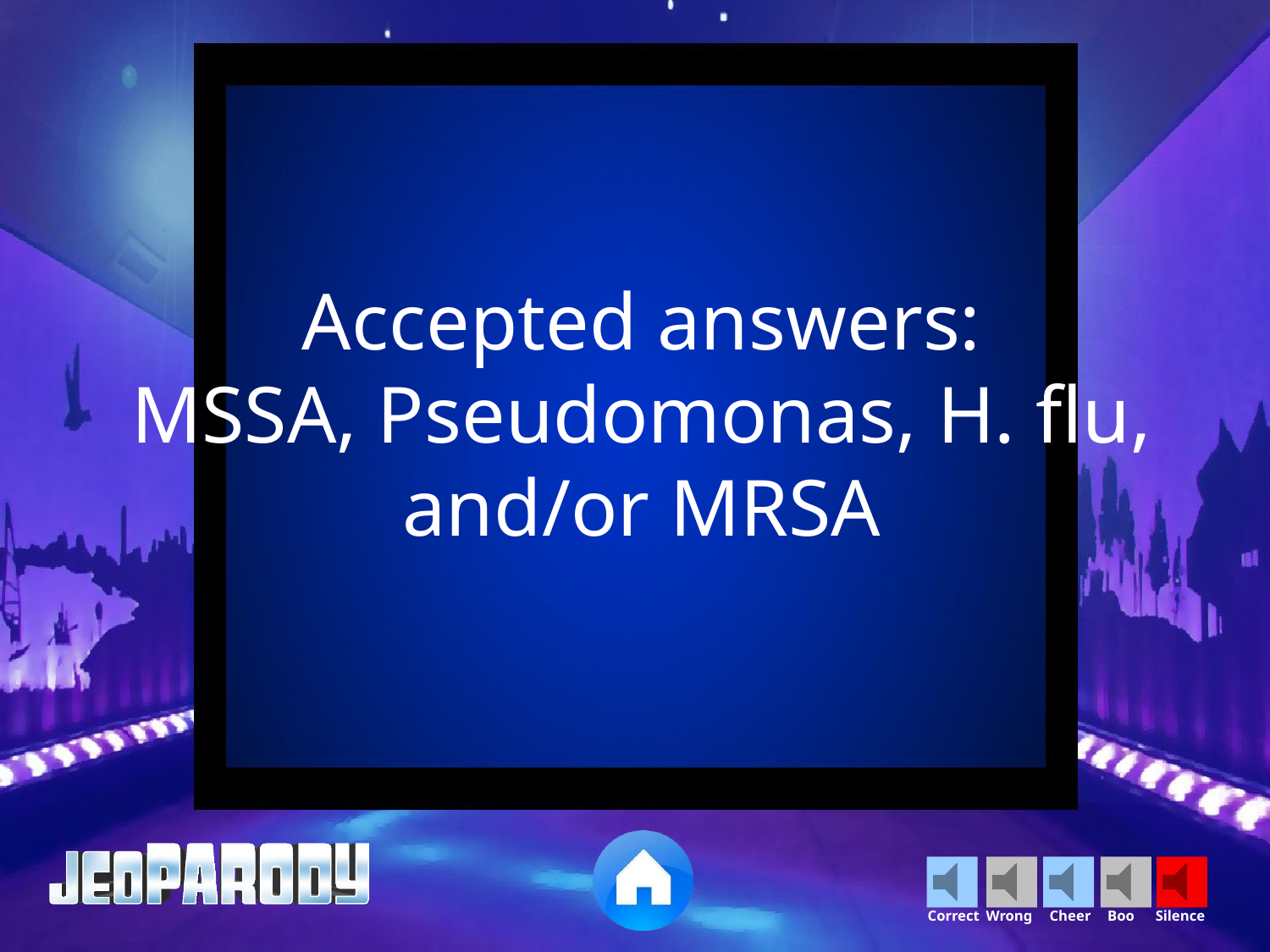

Accepted answers:
MSSA, Pseudomonas, H. flu, and/or MRSA

## Slide 74
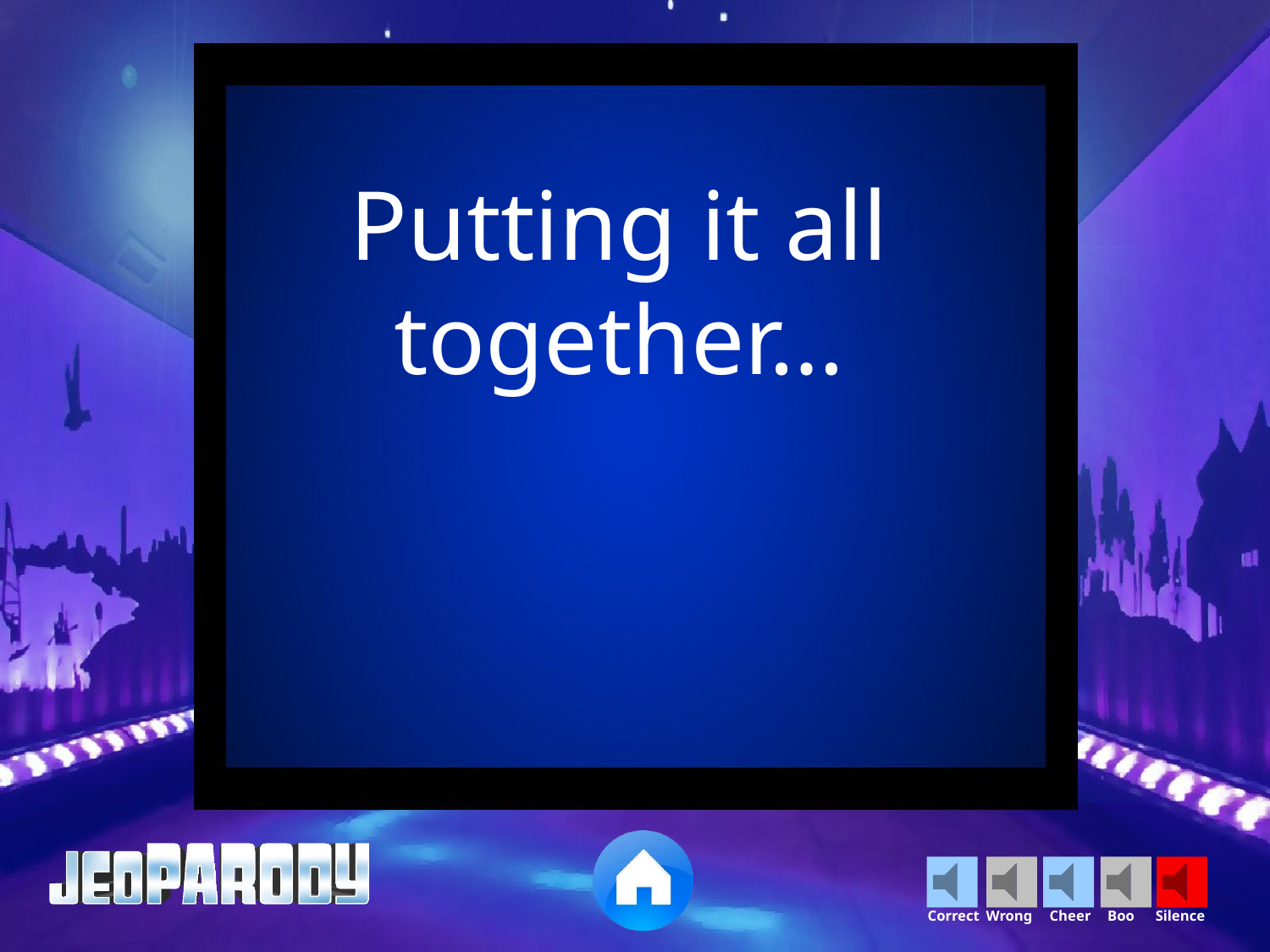

Putting it all together…

## Slide 75
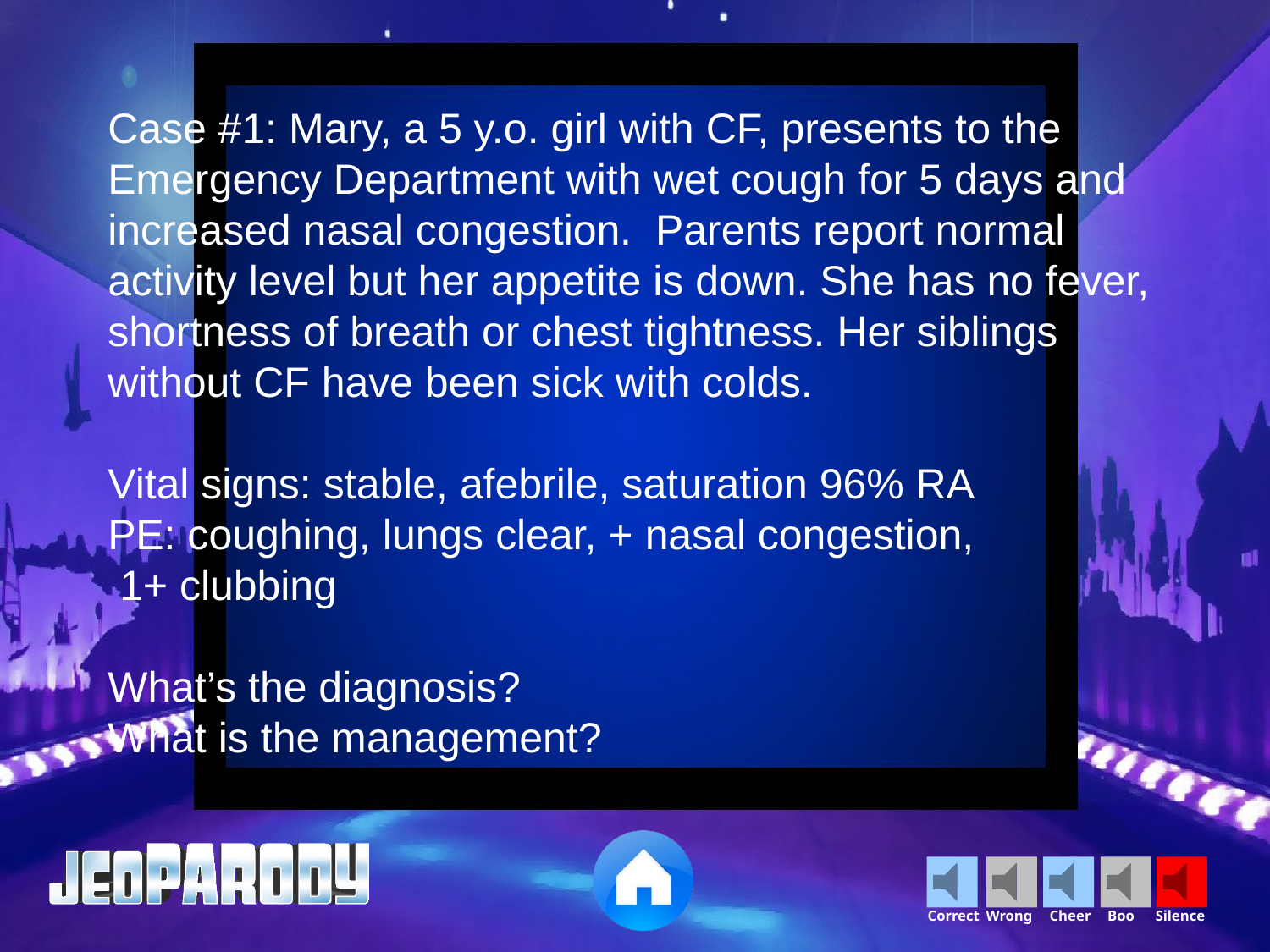

Case #1: Mary, a 5 y.o. girl with CF, presents to the Emergency Department with wet cough for 5 days and increased nasal congestion. Parents report normal activity level but her appetite is down. She has no fever, shortness of breath or chest tightness. Her siblings without CF have been sick with colds.
Vital signs: stable, afebrile, saturation 96% RA
PE: coughing, lungs clear, + nasal congestion,
 1+ clubbing
What’s the diagnosis?
What is the management?

## Slide 76
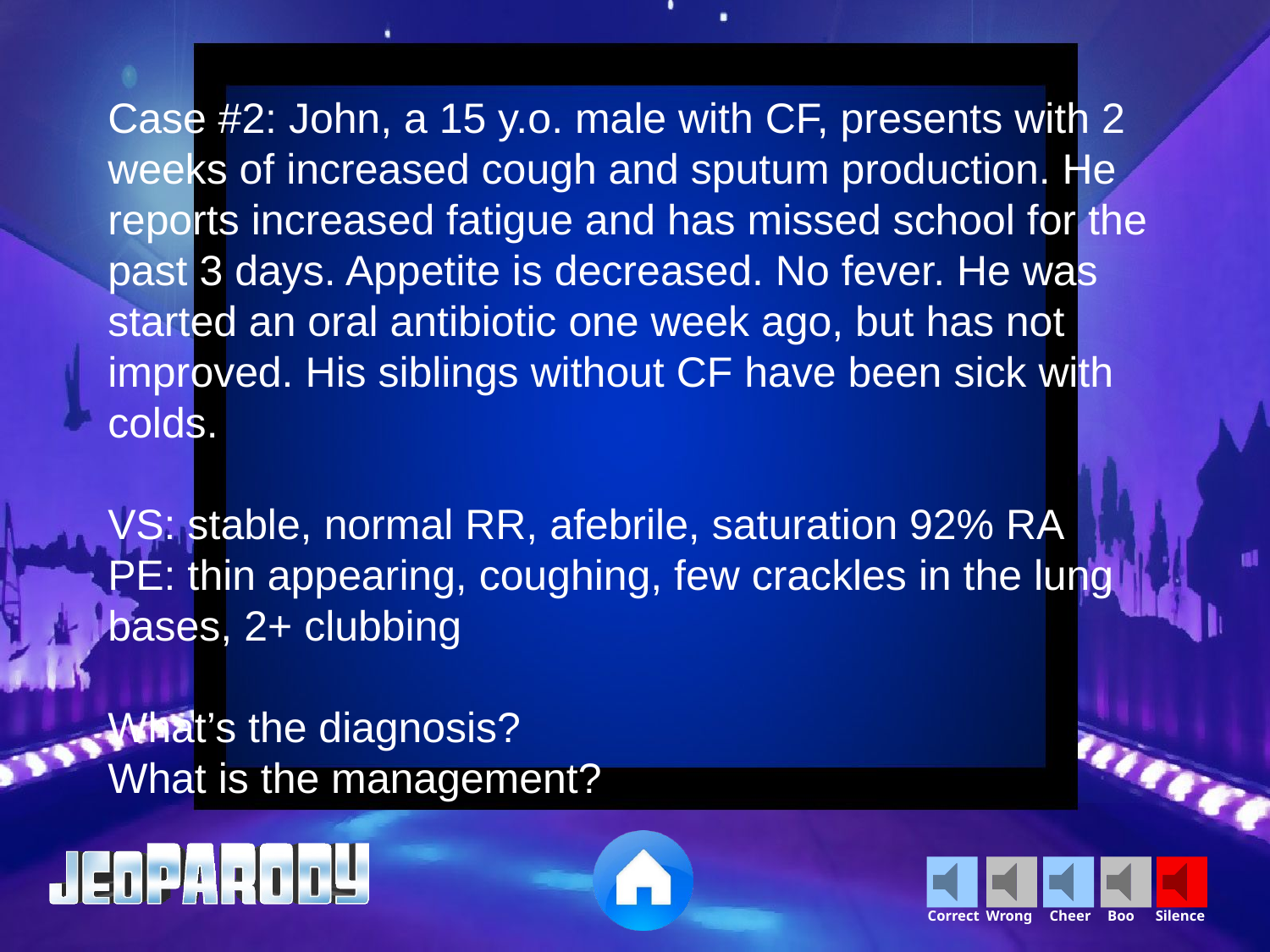

Case #2: John, a 15 y.o. male with CF, presents with 2 weeks of increased cough and sputum production. He reports increased fatigue and has missed school for the past 3 days. Appetite is decreased. No fever. He was started an oral antibiotic one week ago, but has not improved. His siblings without CF have been sick with colds.
VS: stable, normal RR, afebrile, saturation 92% RA
PE: thin appearing, coughing, few crackles in the lung bases, 2+ clubbing
What’s the diagnosis?
What is the management?

## Slide 77
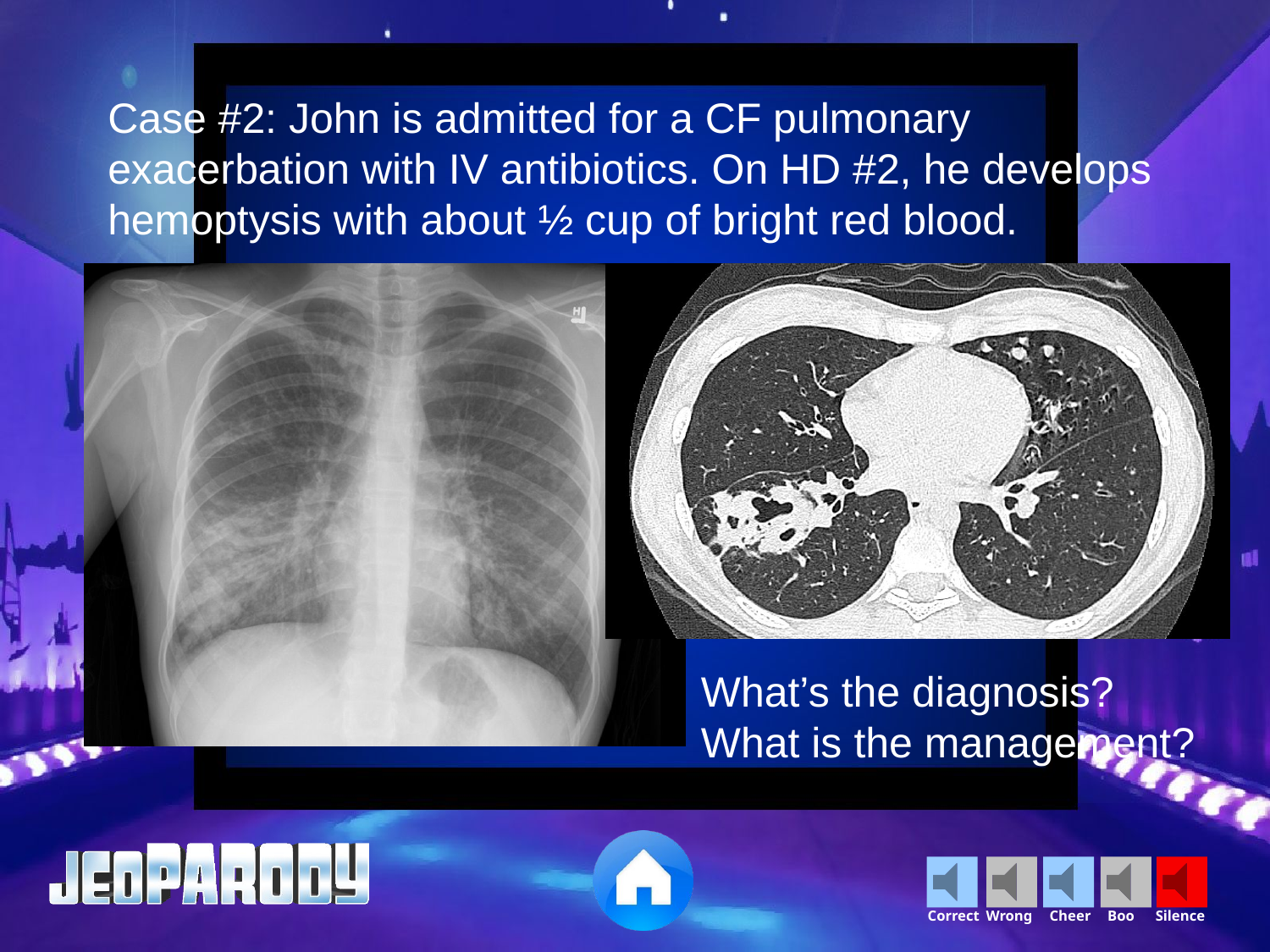

Case #2: John is admitted for a CF pulmonary exacerbation with IV antibiotics. On HD #2, he develops hemoptysis with about ½ cup of bright red blood.
What’s the diagnosis?
What is the management?

## Slide 78
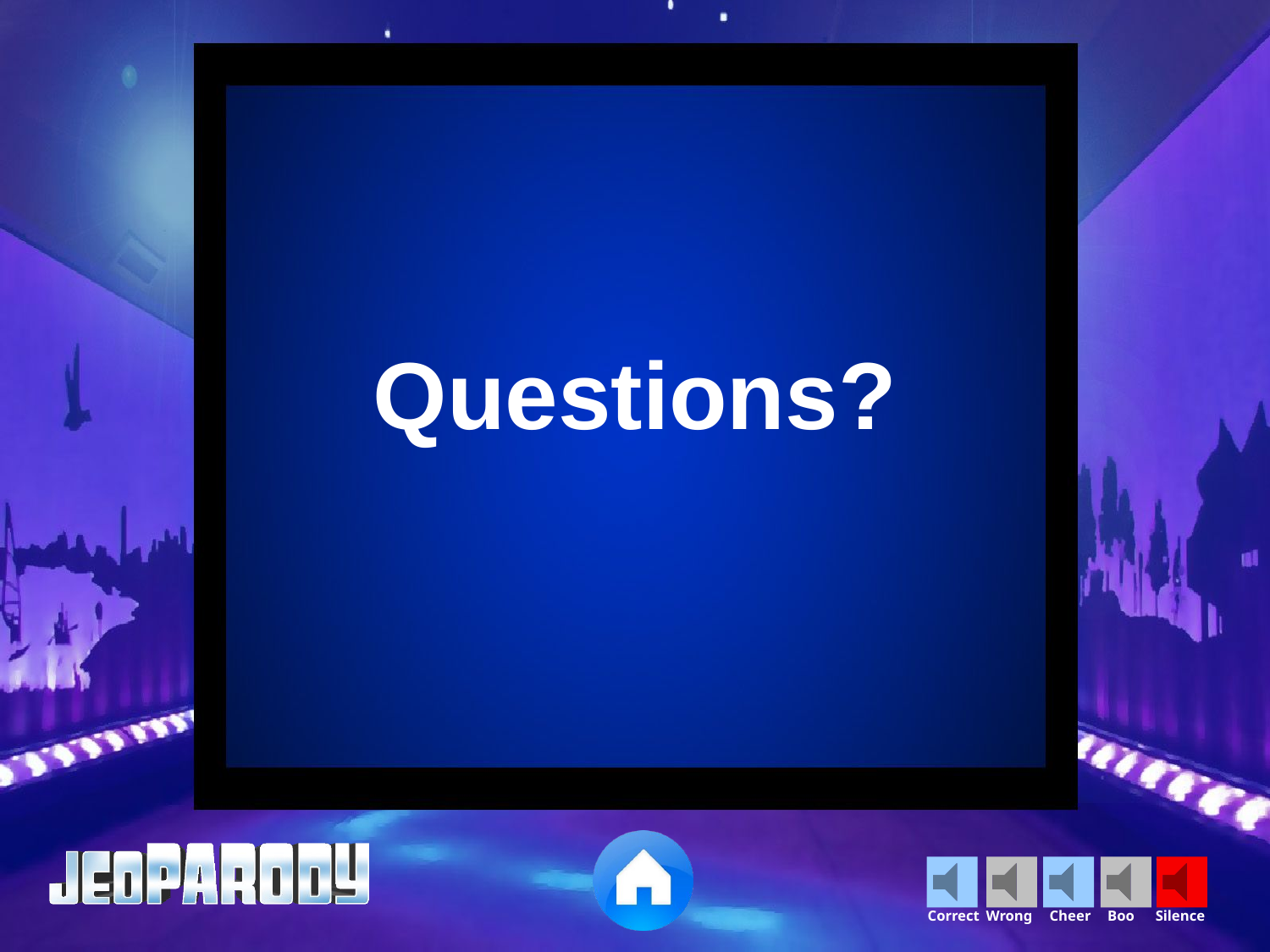

Questions?
