# Supplementary material for: Pediatric Resident Education in Pulmonary (PREP): A Subspecialty Preparatory Boot Camp Curriculum for Pediatric Residents
Source: MedEdPORTAL. 2021 Jan 7;17:11066. doi: 10.15766/mep_2374-8265.11066 (PMC7809931; doi:10.15766/mep_2374-8265.11066)
Supplement: Supplementary file 1 — Example Agenda.docxOrientation Template.pptxIntroduction to Tracheostomies and Ventilators.pptxCystic Fibrosis JeoPARODY.pptxIntroduction to Airway Clearance and Lung Expansion.pptxInstructor Guide CPT.docxInstructor Guide IS.docxInstructor Guide PEP.docxInstructor Guide PAP.docxInstructor Guide OPEP.docxInstructor Guide Insufflator Exsufflator.docxInstructor Guide HFCWO.docxInstructor Guide IPV.docxPREP Day of Evaluation.docxPREP End of Rotation Evaluation.docxPREP Faculty Feedback Survey.docxPREP Focus Group Guide.docx [file mep_2374-8265.11066-s001.zip › E. Intro to Airway Clearance and Lung Expansion.pptx]

## Slide 1
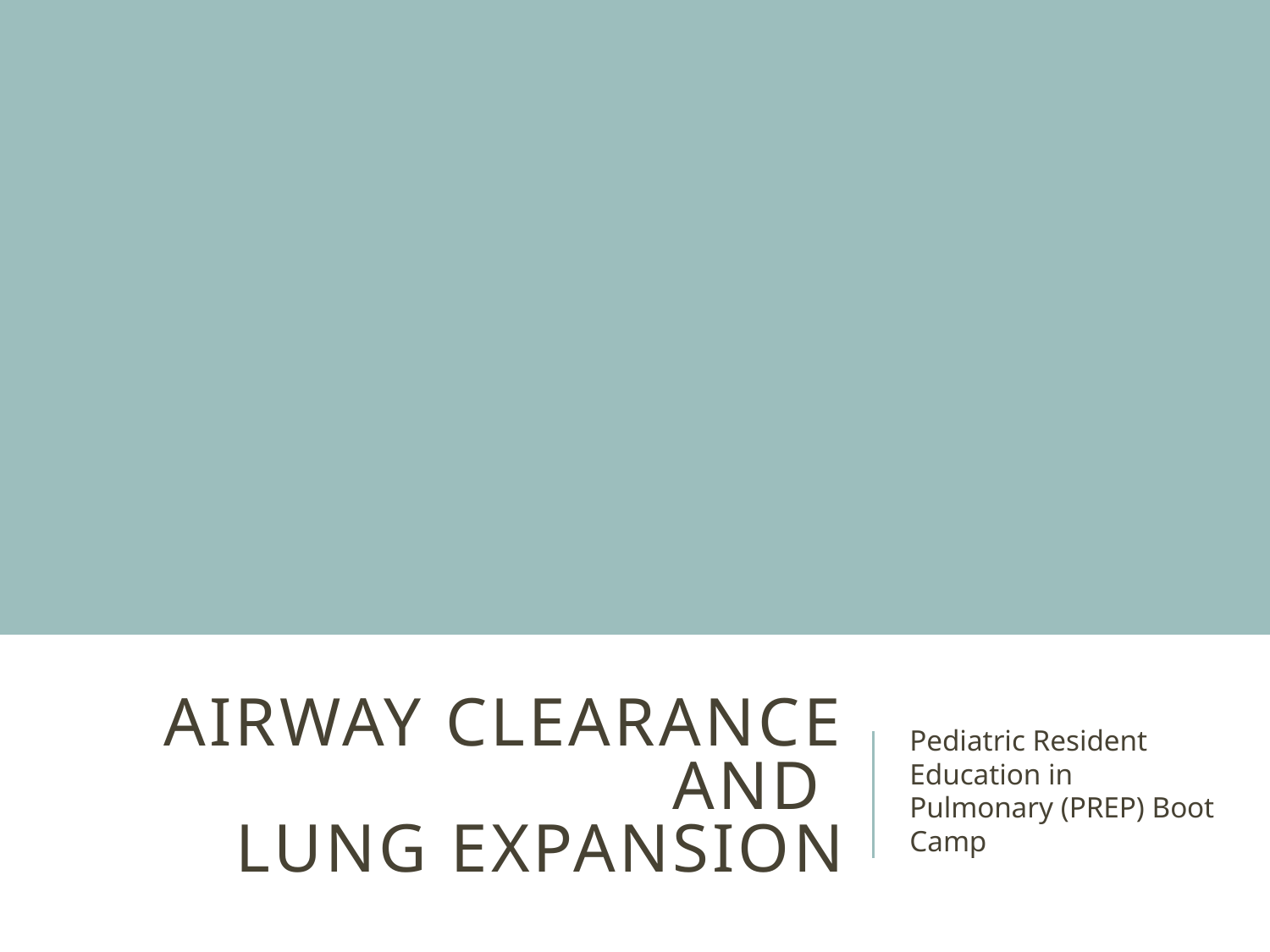

# Airway Clearance and Lung Expansion
Pediatric Resident Education in Pulmonary (PREP) Boot Camp

## Slide 2
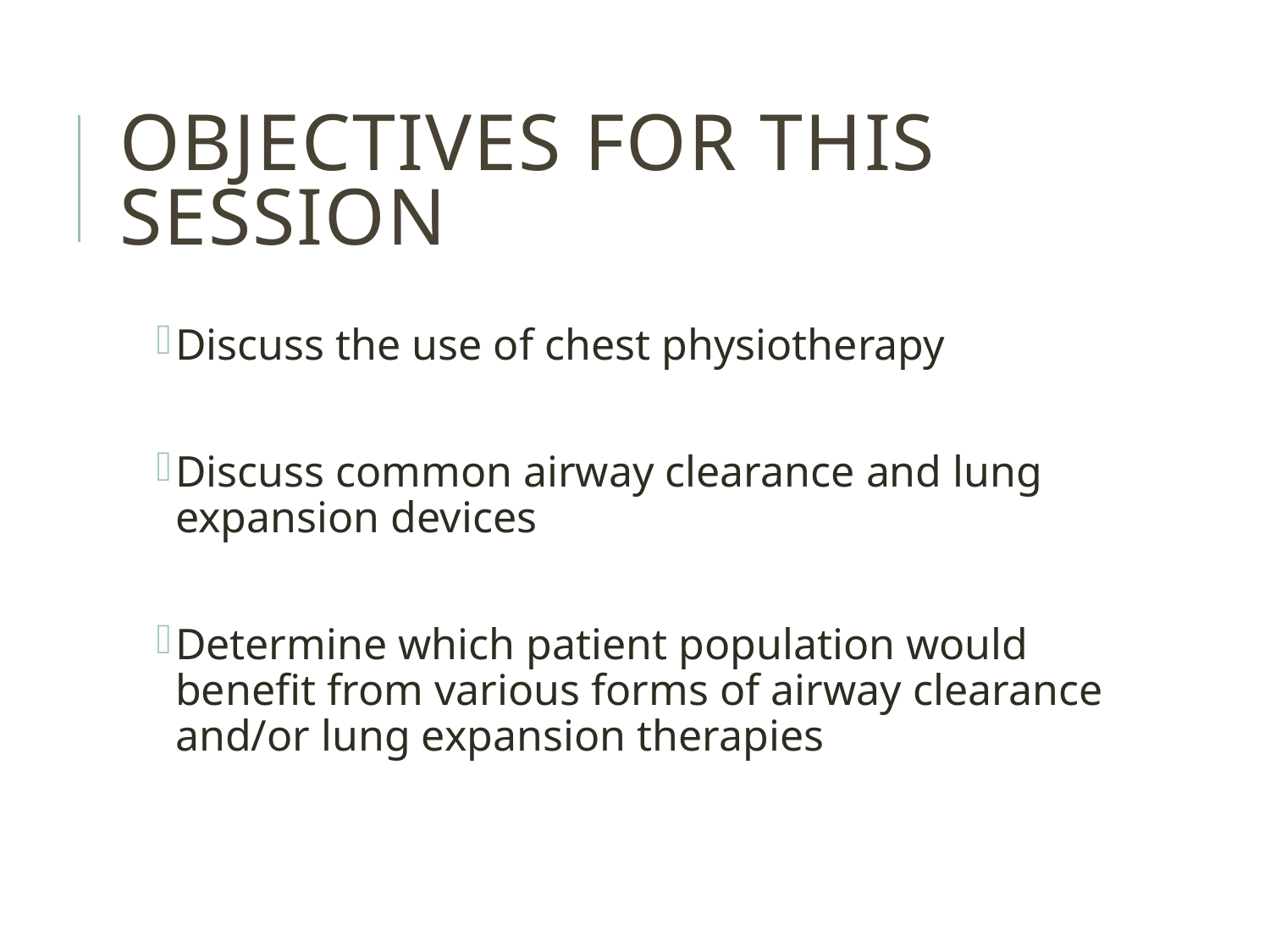

# Objectives for this session
Discuss the use of chest physiotherapy
Discuss common airway clearance and lung expansion devices
Determine which patient population would benefit from various forms of airway clearance and/or lung expansion therapies

## Slide 3
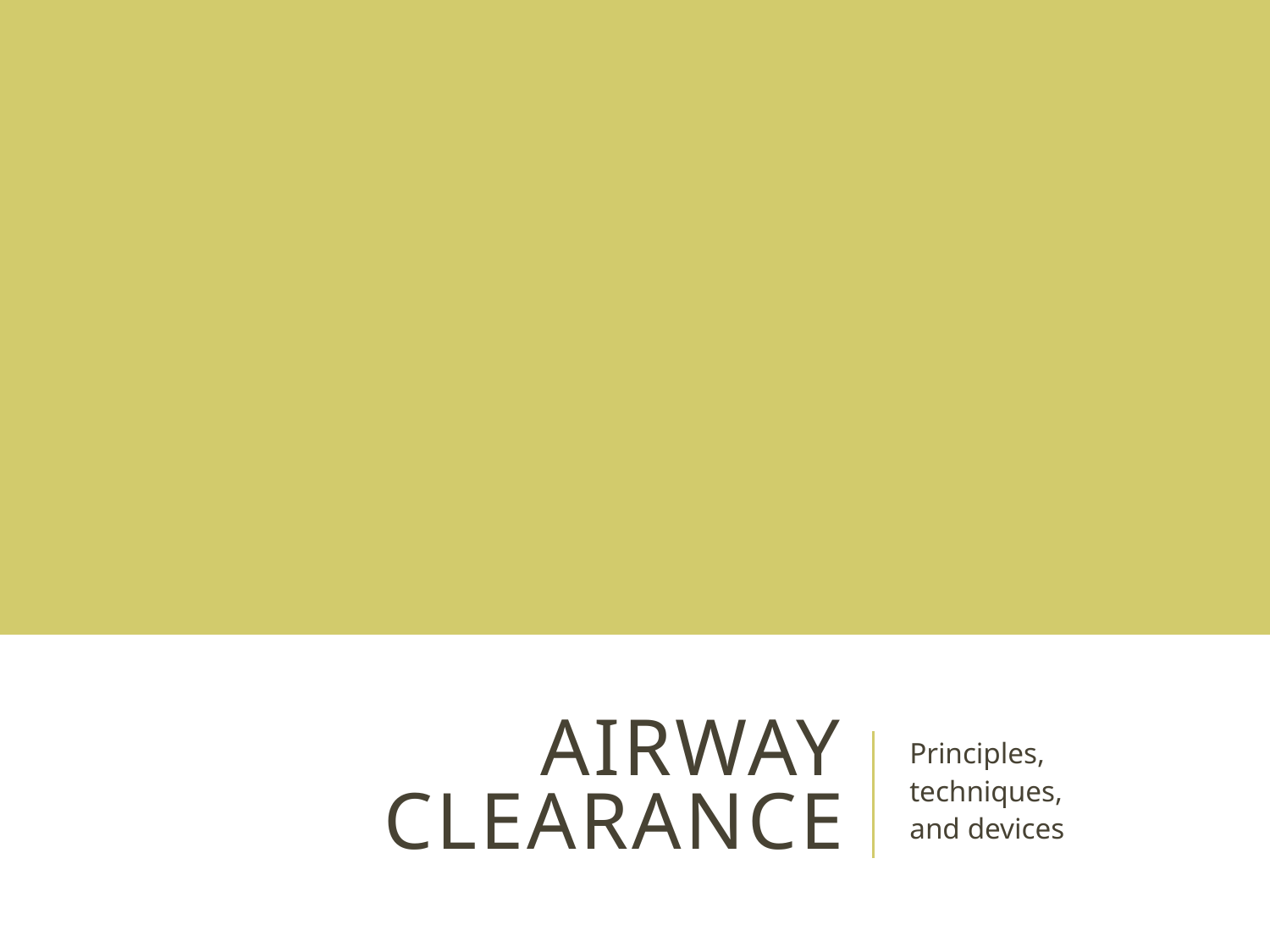

# Airway Clearance
Principles,
techniques,
and devices

## Slide 4
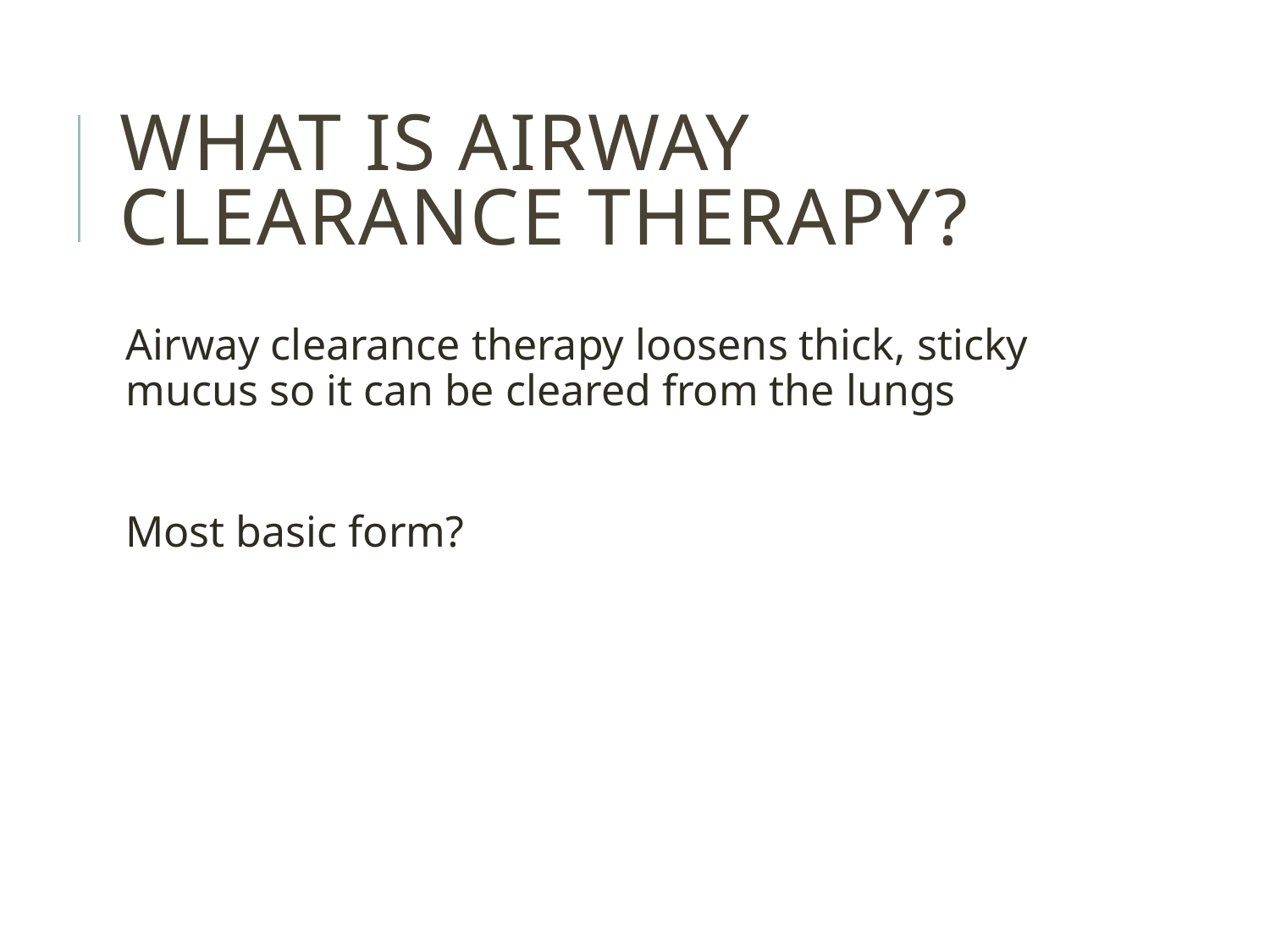

# What is Airway Clearance Therapy?
Airway clearance therapy loosens thick, sticky mucus so it can be cleared from the lungs
Most basic form?

## Slide 5
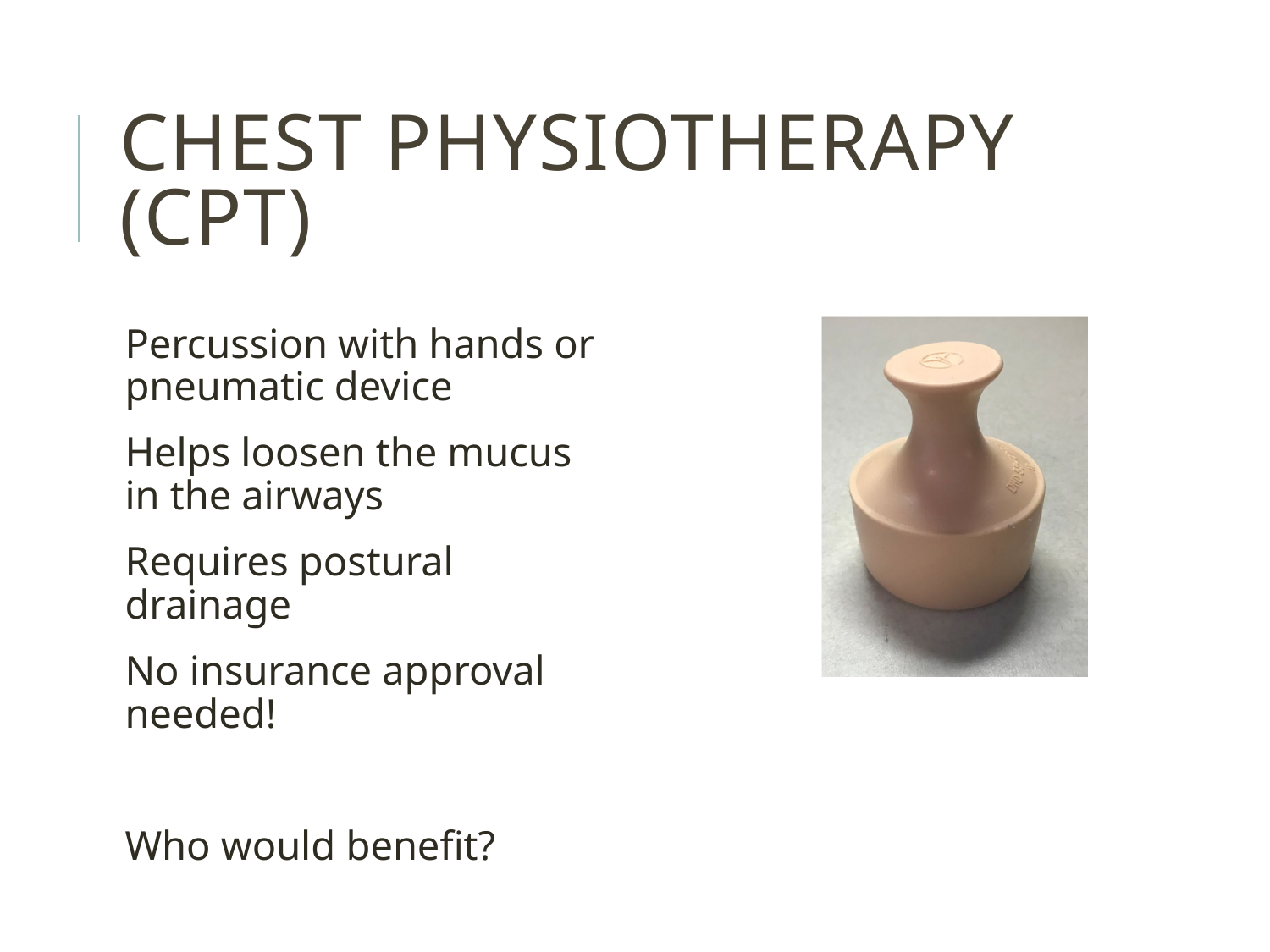

# Chest Physiotherapy (CPT)
Percussion with hands or pneumatic device
Helps loosen the mucus in the airways
Requires postural drainage
No insurance approval needed!
Who would benefit?

## Slide 6
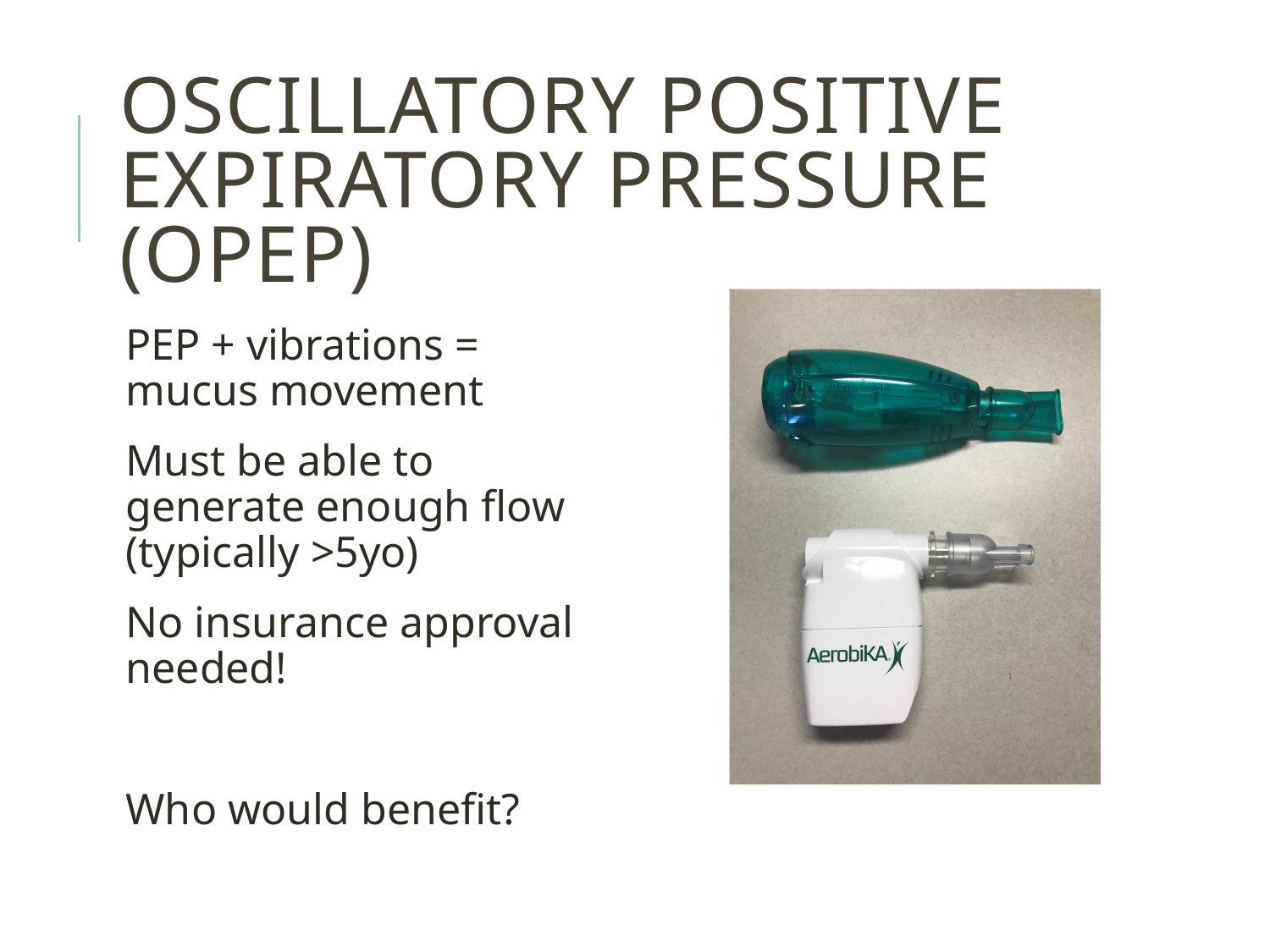

# Oscillatory Positive Expiratory Pressure (OPEP)
PEP + vibrations = mucus movement
Must be able to generate enough flow (typically >5yo)
No insurance approval needed!
Who would benefit?

## Slide 7
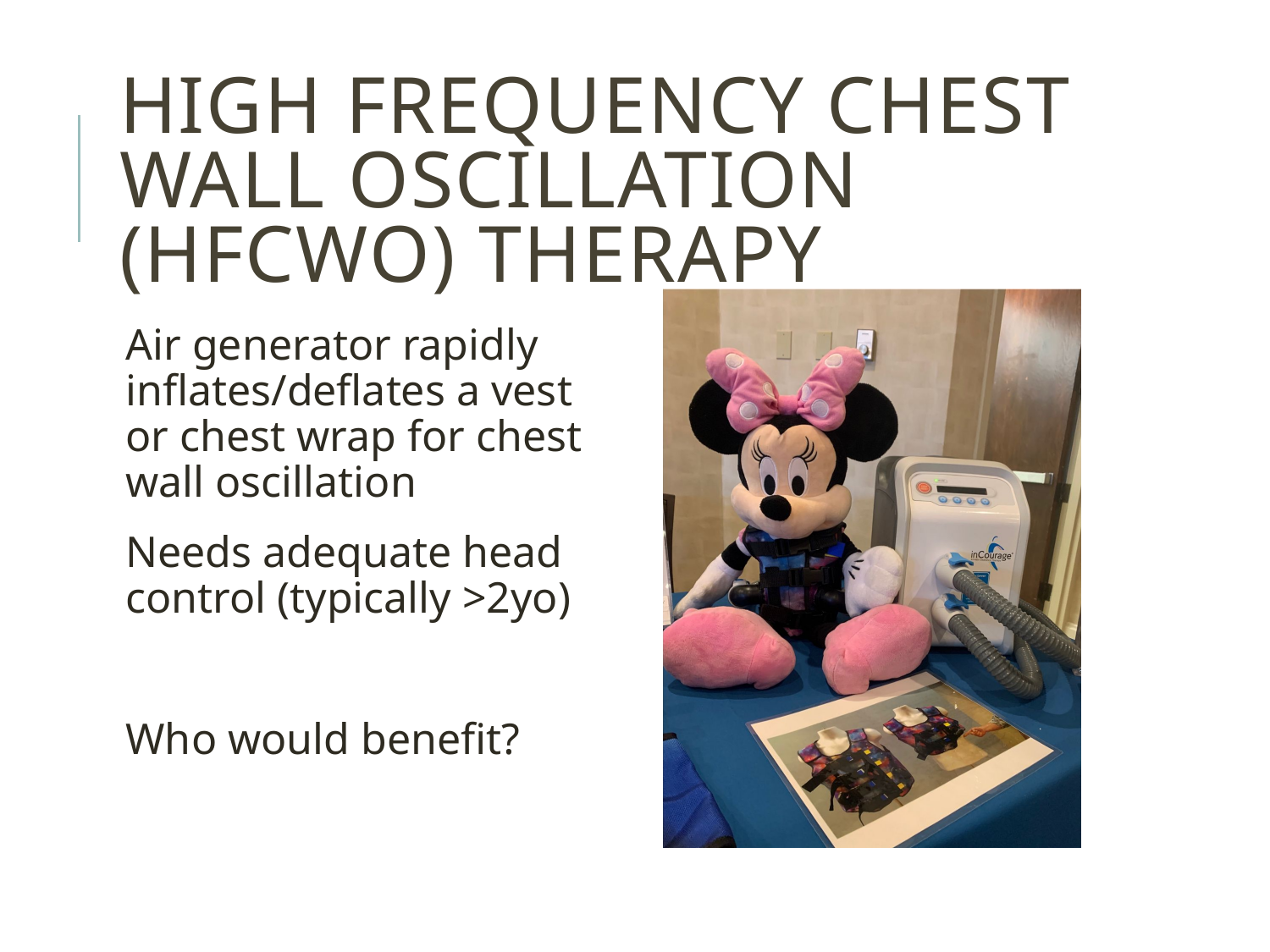

# High Frequency Chest Wall Oscillation (HFCWO) Therapy
Air generator rapidly inflates/deflates a vest or chest wrap for chest wall oscillation
Needs adequate head control (typically >2yo)
Who would benefit?

## Slide 8
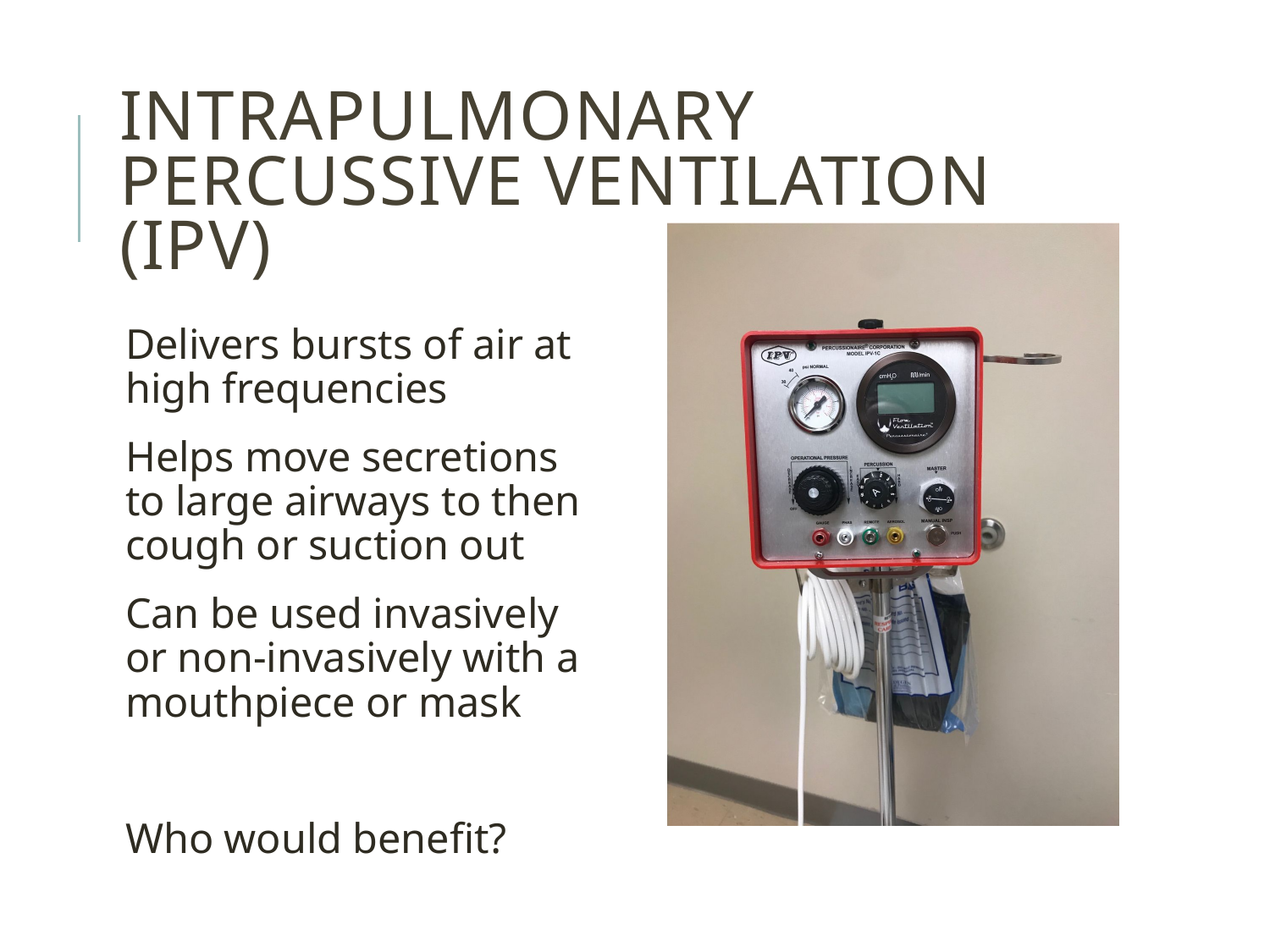

# Intrapulmonary Percussive Ventilation (IPV)
Delivers bursts of air at high frequencies
Helps move secretions to large airways to then cough or suction out
Can be used invasively or non-invasively with a mouthpiece or mask
Who would benefit?

## Slide 9
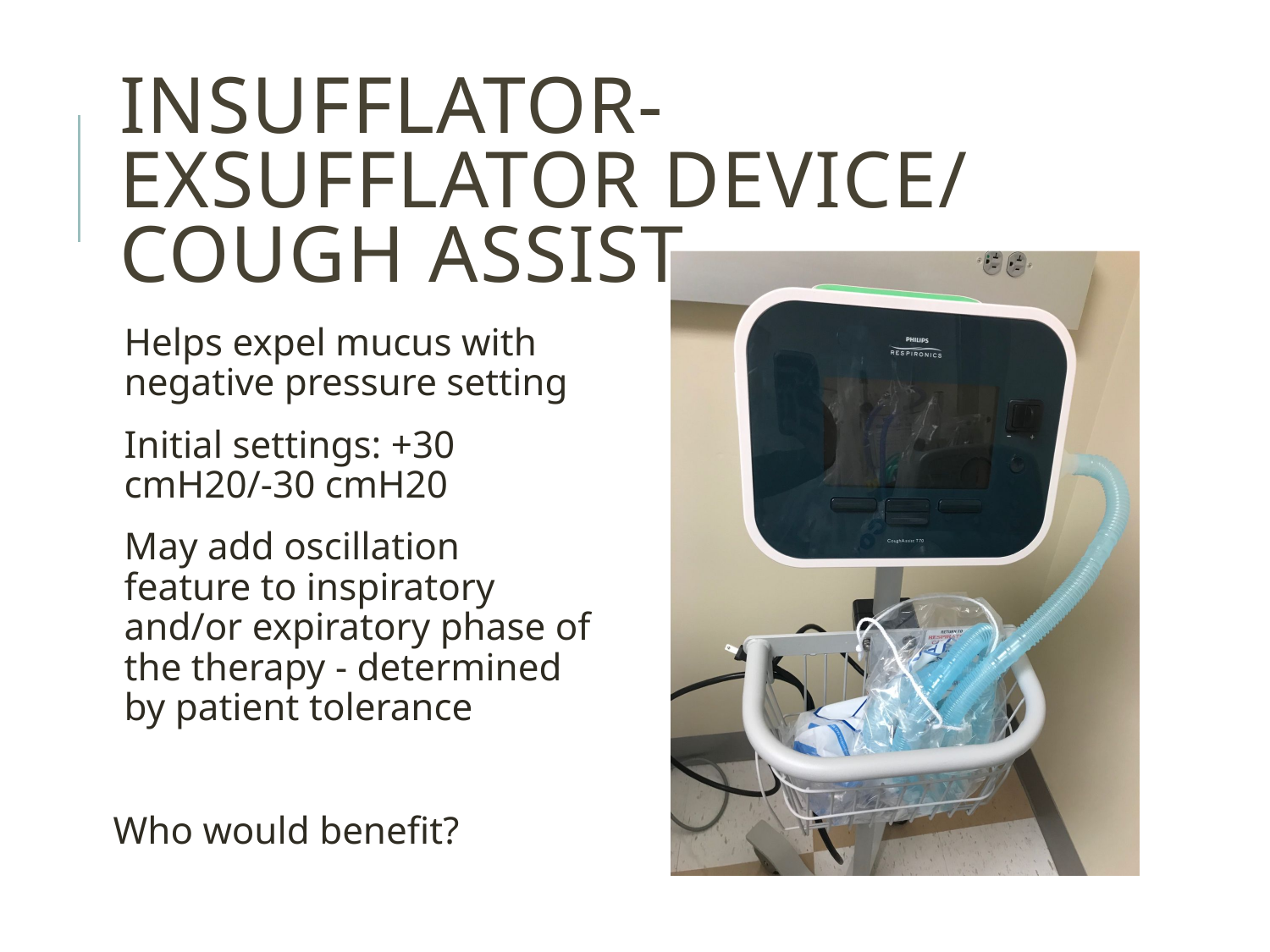

# Insufflator-Exsufflator Device/ Cough Assist
Helps expel mucus with negative pressure setting
Initial settings: +30 cmH20/-30 cmH20
May add oscillation feature to inspiratory and/or expiratory phase of the therapy - determined by patient tolerance
Who would benefit?

## Slide 10
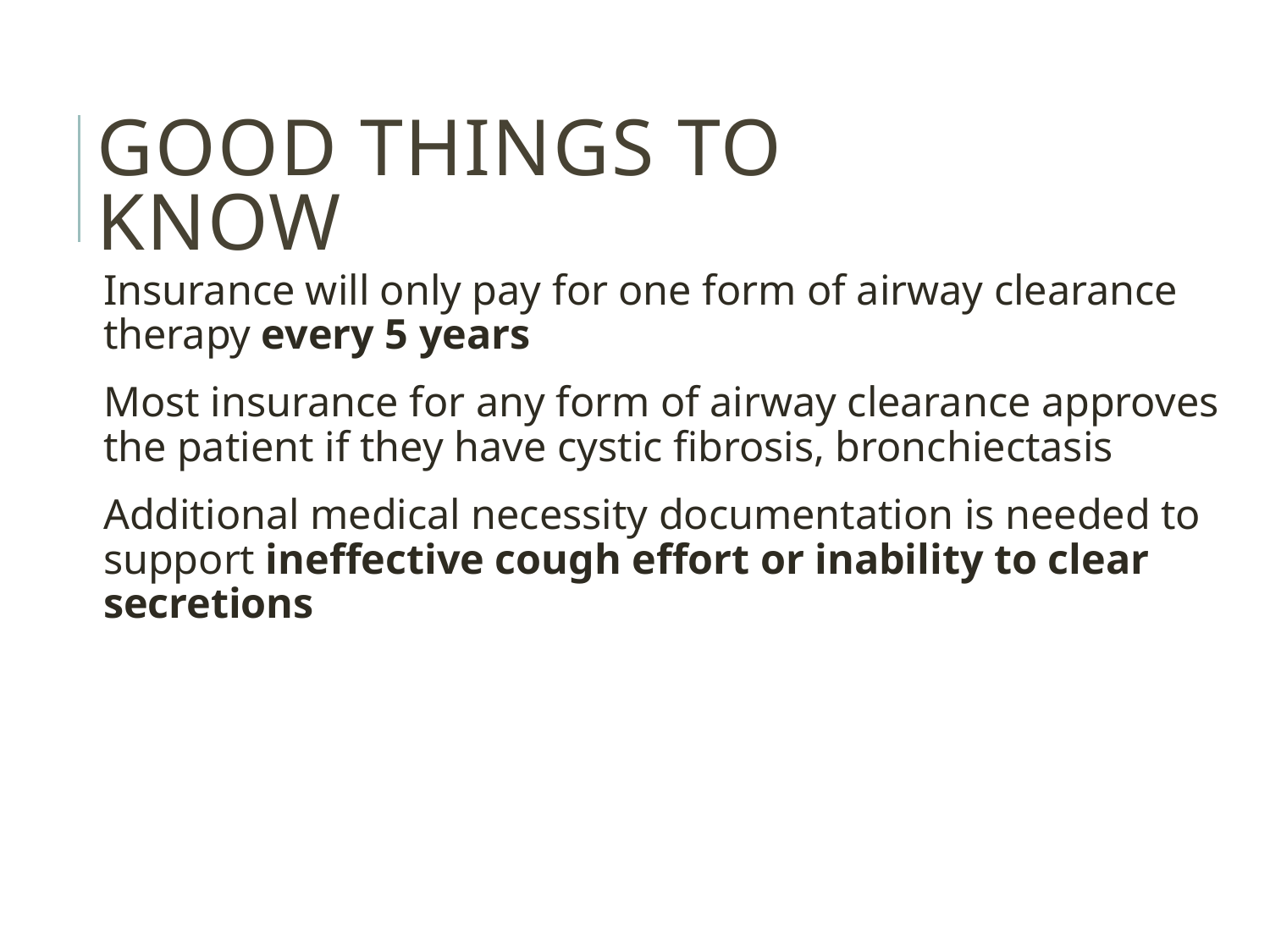

# Good Things To Know
Insurance will only pay for one form of airway clearance therapy every 5 years
Most insurance for any form of airway clearance approves the patient if they have cystic fibrosis, bronchiectasis
Additional medical necessity documentation is needed to support ineffective cough effort or inability to clear secretions

## Slide 11
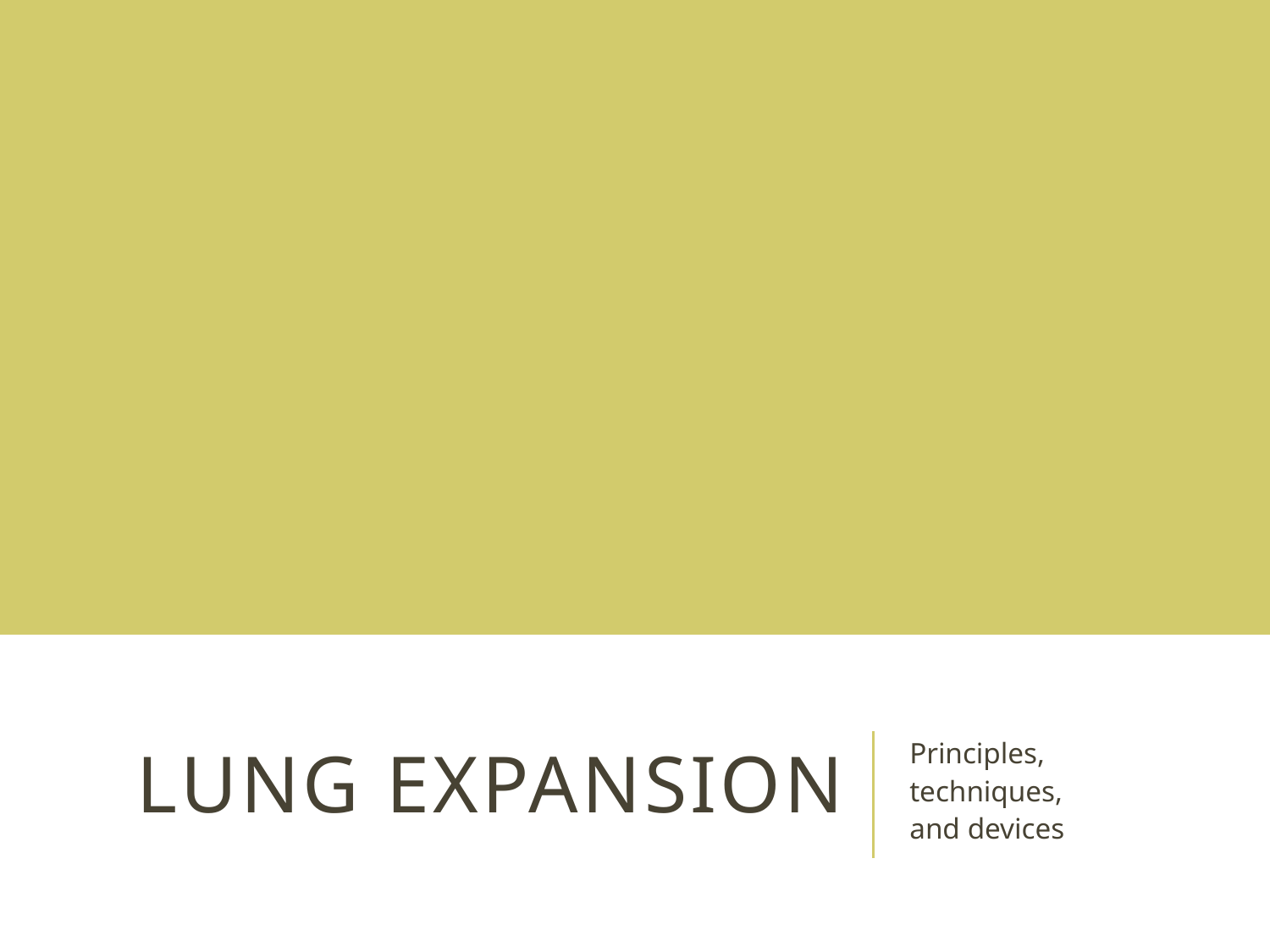

# Lung Expansion
Principles,
techniques,
and devices

## Slide 12
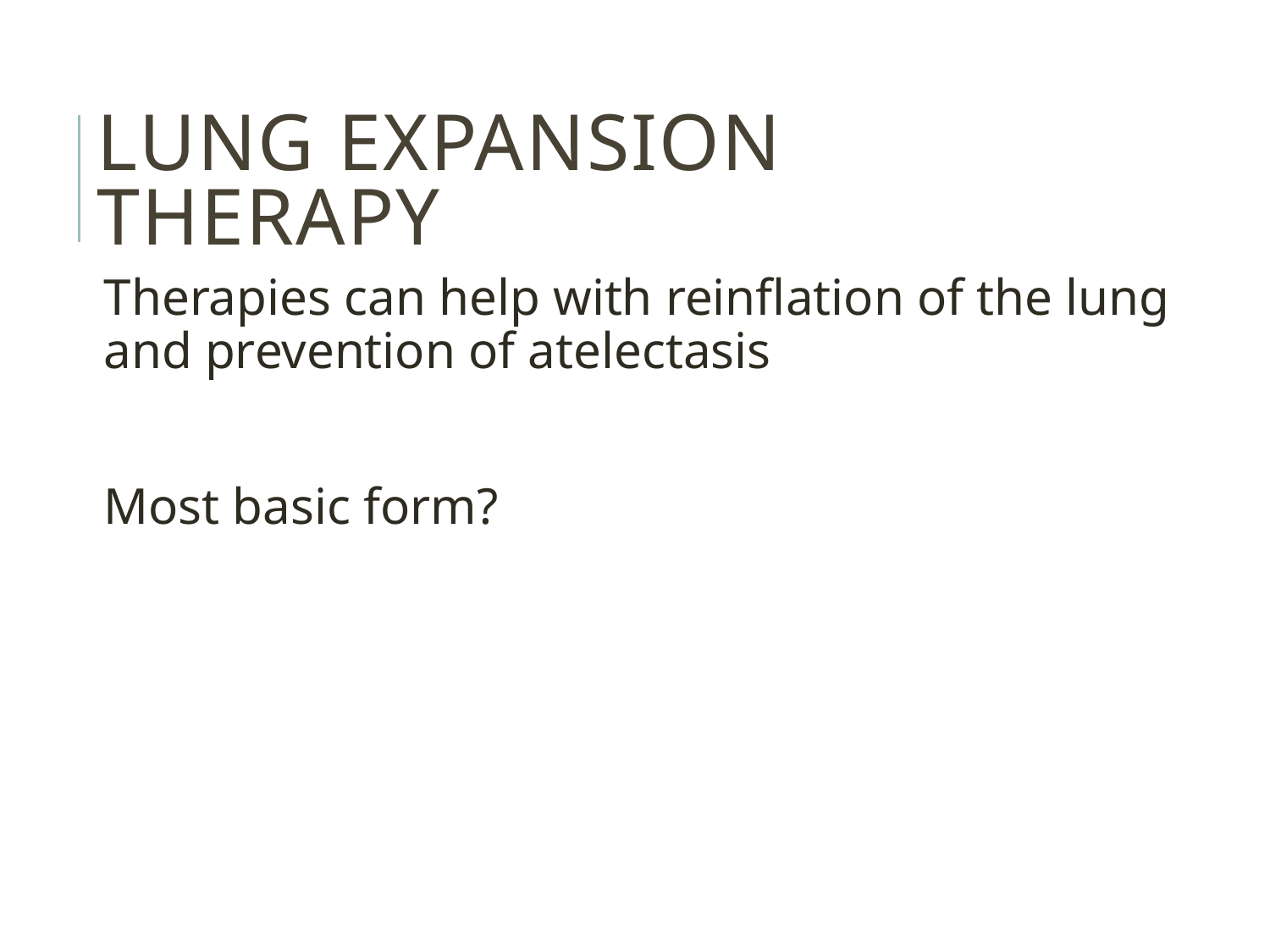

# Lung Expansion Therapy
Therapies can help with reinflation of the lung and prevention of atelectasis
Most basic form?

## Slide 13
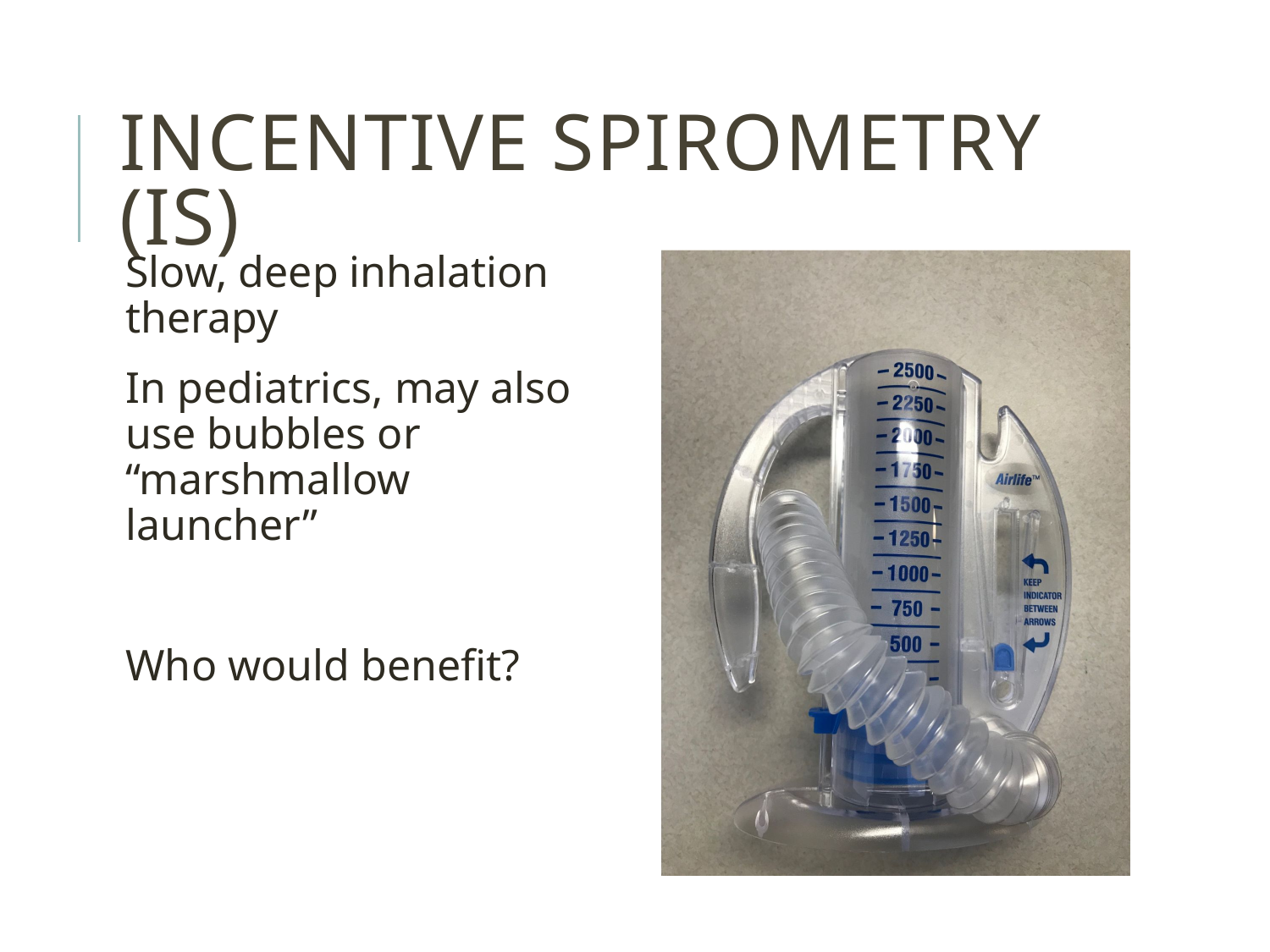

# Incentive Spirometry (IS)
Slow, deep inhalation therapy
In pediatrics, may also use bubbles or “marshmallow launcher”
Who would benefit?

## Slide 14
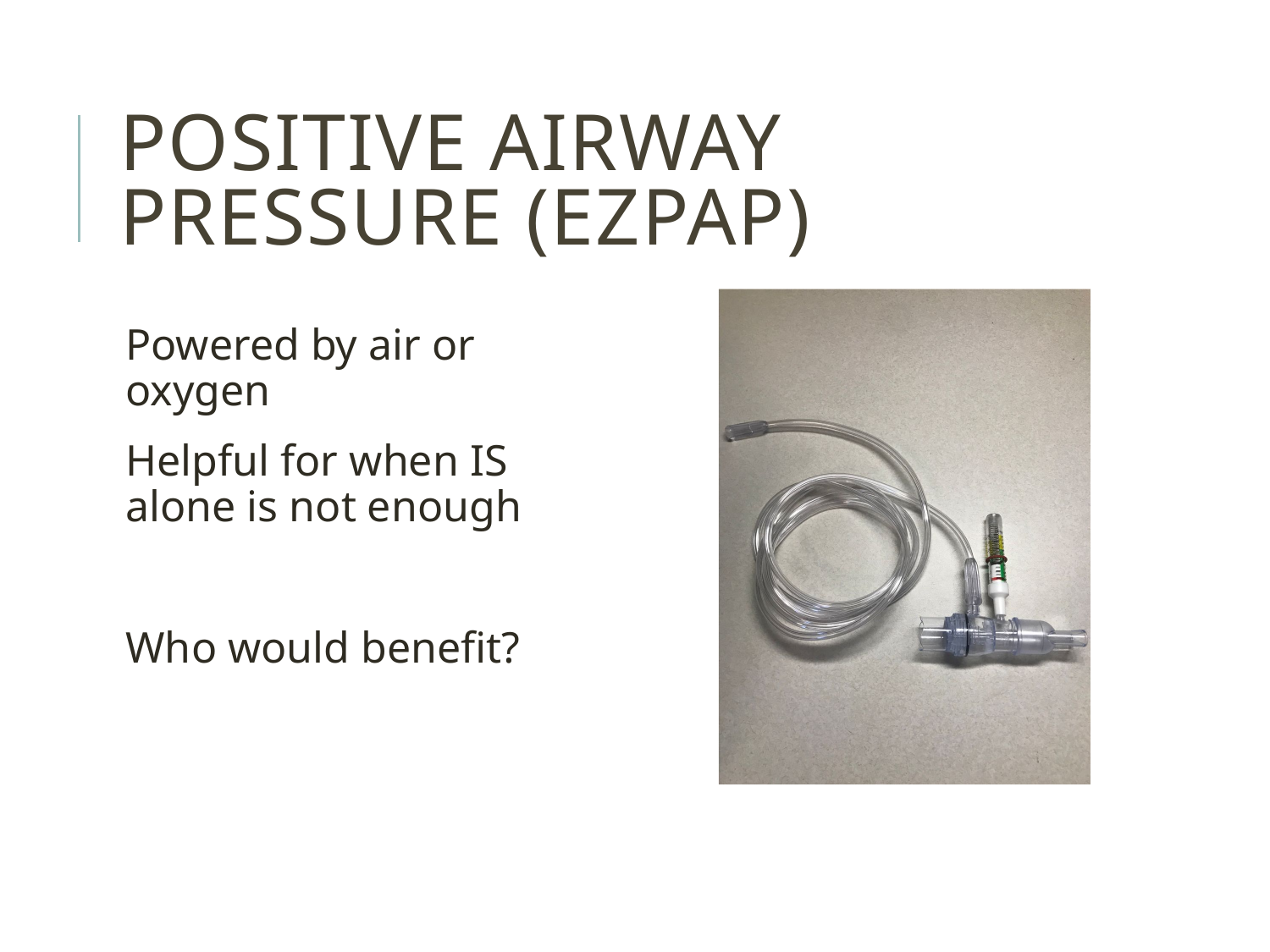

# Positive Airway Pressure (EzPAP)
Powered by air or oxygen
Helpful for when IS alone is not enough
Who would benefit?

## Slide 15
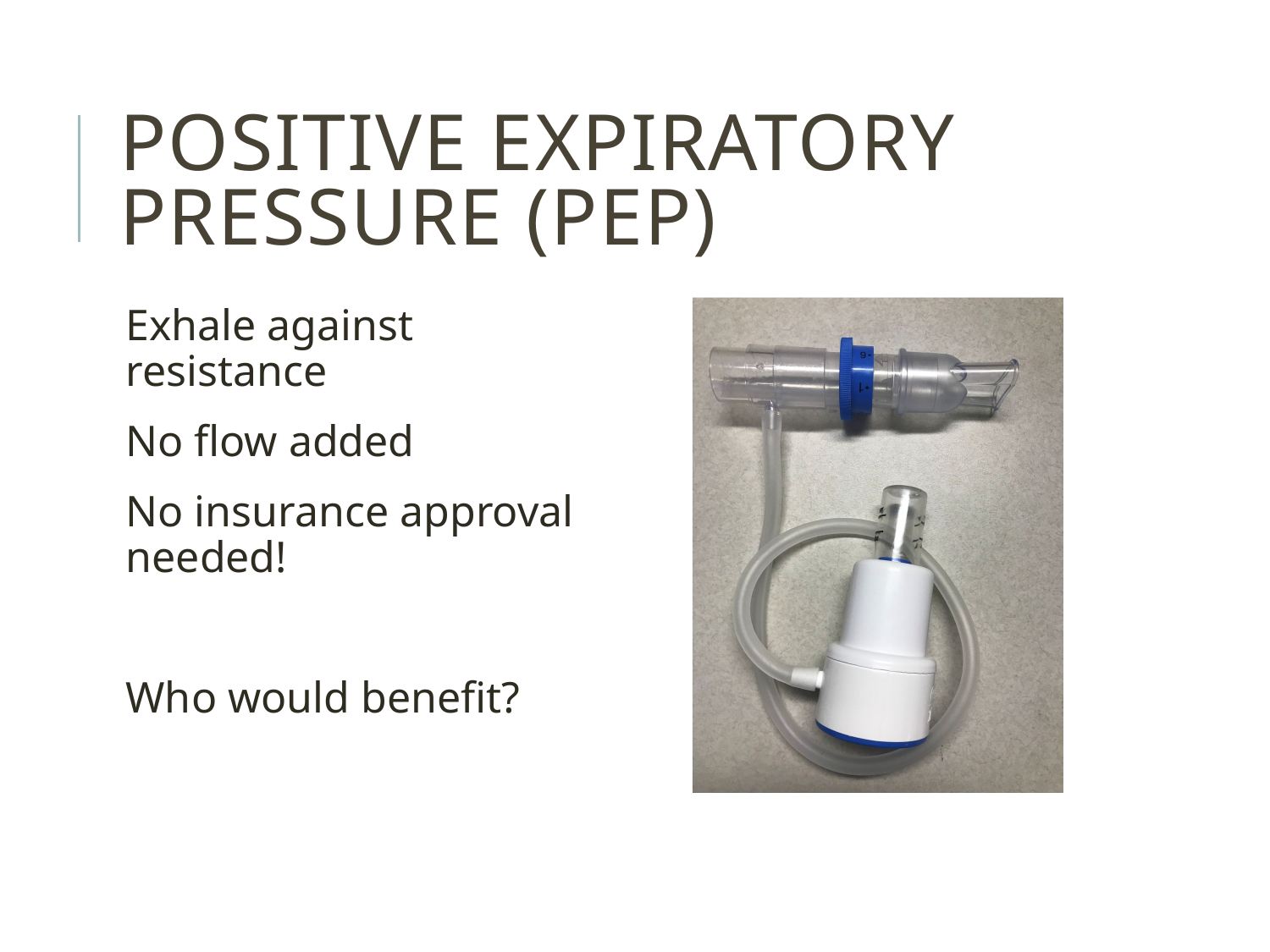

# Positive Expiratory Pressure (PEP)
Exhale against resistance
No flow added
No insurance approval needed!
Who would benefit?

## Slide 16
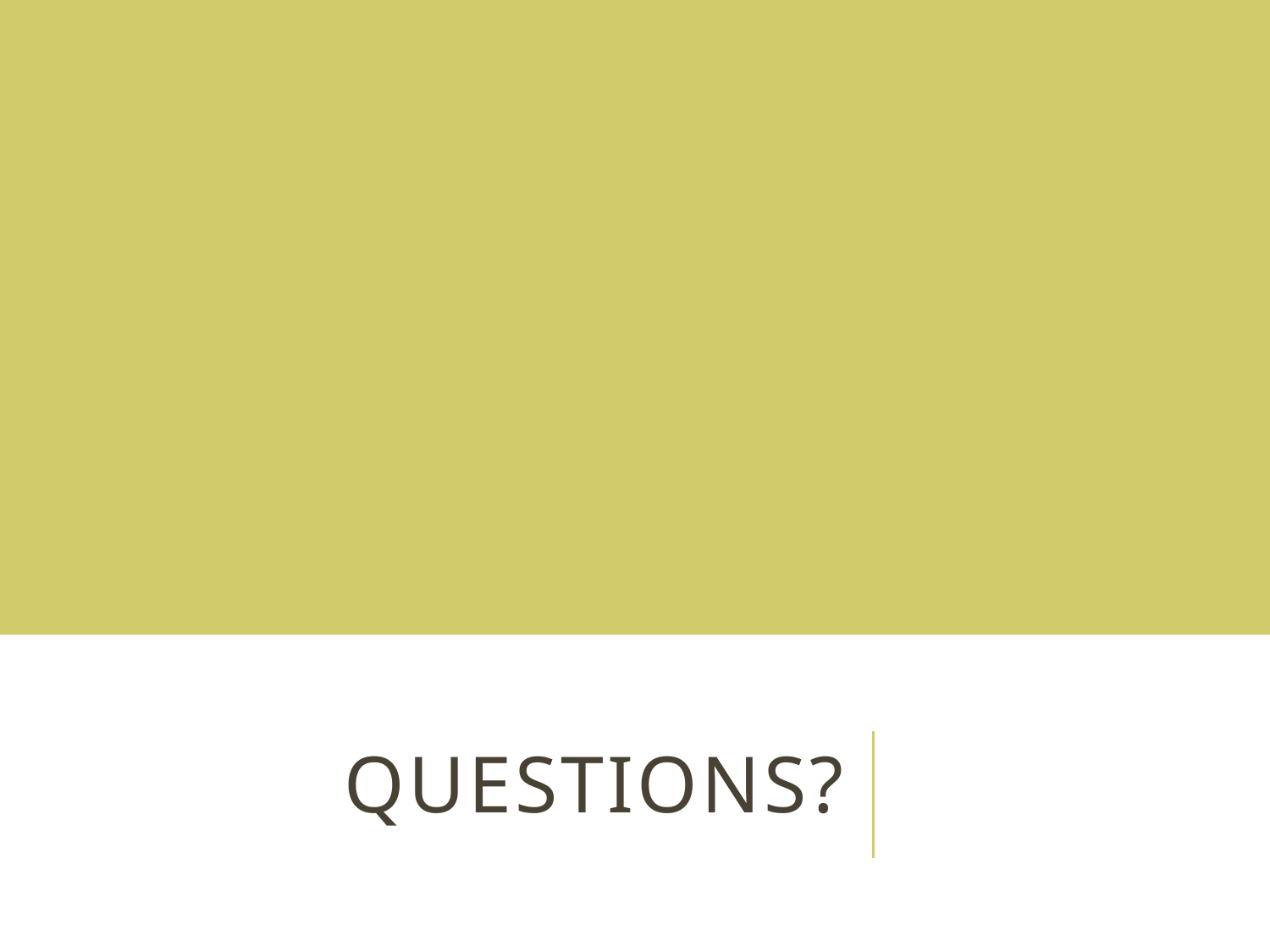

# Questions?
