# Supplementary material for: Pediatric Resident Education in Pulmonary (PREP): A Subspecialty Preparatory Boot Camp Curriculum for Pediatric Residents
Source: MedEdPORTAL. 2021 Jan 7;17:11066. doi: 10.15766/mep_2374-8265.11066 (PMC7809931; doi:10.15766/mep_2374-8265.11066)
Supplement: Supplementary file 1 — Example Agenda.docxOrientation Template.pptxIntroduction to Tracheostomies and Ventilators.pptxCystic Fibrosis JeoPARODY.pptxIntroduction to Airway Clearance and Lung Expansion.pptxInstructor Guide CPT.docxInstructor Guide IS.docxInstructor Guide PEP.docxInstructor Guide PAP.docxInstructor Guide OPEP.docxInstructor Guide Insufflator Exsufflator.docxInstructor Guide HFCWO.docxInstructor Guide IPV.docxPREP Day of Evaluation.docxPREP End of Rotation Evaluation.docxPREP Faculty Feedback Survey.docxPREP Focus Group Guide.docx [file mep_2374-8265.11066-s001.zip › F. Instructor Guide CPT.docx]

# PREP Boot Camp Hands-On Session Airway Clearance and Lung Expansion Devices Instructor Guide: Chest Physiotherapy (CPT)

## Learning Objectives:

1. Describe what is chest physiotherapy and how it works
2. Identify which patient population would benefit from chest physiotherapy
3. Discuss when to transition patient from chest physiotherapy to other airway clearance therapy

## Class Preparation:

### Equipment and Supplies:

- Percussive cup


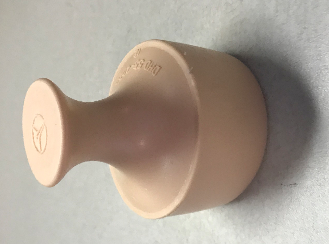


### Location:

- Conference room or unoccupied patient room

## Hands-On Learning Experience:

- Experience firsthand CPT therapy
- Each learner should attempt 1 minute of CPT therapy on each other
- Evaluate understanding and comprehension of the learner through discussion of key concepts

## Discussion of Key Concepts:

1. What are the different names for this type of therapy?
   - Chest physiotherapy
   - Chest physical therapy
   - Percussing
   - Percussion therapy
2. What is the goal of CPT?
   - Mobilizing secretions
3. What are indications and contraindications for CPT?
   - Indications: cystic fibrosis, bronchiectasis, recurring pneumonia, neuromuscular disease with effective cough, any other need for enhanced airway clearance
   - Contraindications: fractured ribs, unstable chest, chest tube, acute head/spine injury, pulmonary hemorrhage, pleural effusions/empyema, pneumothorax, pneumomediastinum, pulmonary embolism, burns, open wounds, foreign body aspiration
4. What are complications and consequences of CPT?
   - Newborns have very compliant chest and percussion too hard can result in lung volume loss during therapy
   - Hypoxemia secondary to mobilizing secretions causing V/Q mismatch.
   - Tolerance
   - Aspiration – CPT should be done prior to eating/feeding. Feeds should be stopped at least 30 min prior to therapy
   - Increase in intracranial and intraocular pressure with Trendelenburg positioning. We discourage Trendelenburg positioning and instead recommend modified lower lobe therapy which is supine right/left side up with a pillow under the hip
5. Treatment Pearls:
   - *Positioning* utilizes gravity to assist in effectively draining secretions from the smaller airways into the central airway where they can either be coughed up or suctioned out
   - *Turning* assists in secretion mobilization
   - *Coughing* breaks up and expectorates secretions in the lungs
   - *Deep breathing* helps expand the lungs and forces an improved distribution of the air into all sections of the lungs
   - *Percussion* (rhythmically striking the chest wall with cupped hands or percussor) breaks up thick secretions in the lungs
   - Treatment time – 3-5 minutes per lung segment
   - CPT requires skill and constant assessment
6. What positioning for CPT would best isolate specific lung lobes?
   - Upper lobes: sitting, percussing anterior or posterior upper chest
   - Right middle lobe: supine, right side up
   - Lower lobes: Trendelenburg, right/left side up
7. What is the correct technique to provide CPT?
   - Clap with a cupped hand or rubber palm cup over the designated lung field. This vibrates the lungs and loosens secretions.
   - Avoid spine and sternum
   - Never percuss on floating ribs or below where you can potentially injure the stomach, liver, spleen, kidneys, etc.
   - CPT is typically used for kids < 2 years of age; high frequency chest wall oscillation therapy may be the preferred inpatient therapy in children > 2 years of age
8. Considerations for ordering CPT for home use:
   - First option for airway clearance
   - Insurance does not typically cover percussors
   - Patients on other airway clearance devices (high frequency chest wall oscillation and cough assist) should still be educated on CPT in the event their equipment fails or not readily available

## References

Bylander LL. Foundations in Neonatal and Pediatric Respiratory Care: Airway clearance and lung expansion therapy. Burlington, MA: Jones & Bartlett Learning; 2019.

Walsh BK. Perinatal and Pediatric Respiratory Care: Airway clearance techniques and lung expansion. 3^rd^ ed. St. Louis, MO: Saunders Elsevier; 2010. 196-219 p.

Wright S, Wakeman R, Collins N, Chatwin M. Disorders of the Respiratory Tract in Children: Physical therapies in pediatric respiratory disease. 9^th^ ed. Philidelphia, PA: Elsevier; 2019. 273-288 p.
