# Supplementary material for: Pediatric Resident Education in Pulmonary (PREP): A Subspecialty Preparatory Boot Camp Curriculum for Pediatric Residents
Source: MedEdPORTAL. 2021 Jan 7;17:11066. doi: 10.15766/mep_2374-8265.11066 (PMC7809931; doi:10.15766/mep_2374-8265.11066)
Supplement: Supplementary file 1 — Example Agenda.docxOrientation Template.pptxIntroduction to Tracheostomies and Ventilators.pptxCystic Fibrosis JeoPARODY.pptxIntroduction to Airway Clearance and Lung Expansion.pptxInstructor Guide CPT.docxInstructor Guide IS.docxInstructor Guide PEP.docxInstructor Guide PAP.docxInstructor Guide OPEP.docxInstructor Guide Insufflator Exsufflator.docxInstructor Guide HFCWO.docxInstructor Guide IPV.docxPREP Day of Evaluation.docxPREP End of Rotation Evaluation.docxPREP Faculty Feedback Survey.docxPREP Focus Group Guide.docx [file mep_2374-8265.11066-s001.zip › G. Instructor Guide IS.docx]

# PREP Boot Camp Hands-On Session Airway Clearance and Lung Expansion Devices Instructor Guide: Incentive Spirometer (IS)

## Learning Objectives:

1. Describe what is an incentive spirometer and how it works
2. Identify which patient population benefits from incentive spirometer therapy
3. Discuss how to evaluate the effectiveness of incentive spirometer therapy

Class Preparation:

### Equipment and Supplies:

- Incentive spirometer


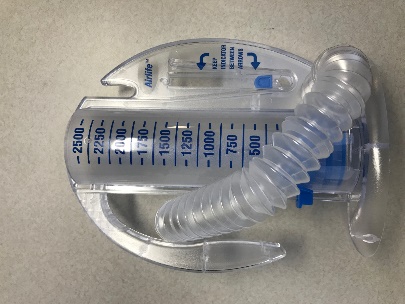


- Bacteria filter for each learner (required, can be used with or without mouthpiece)


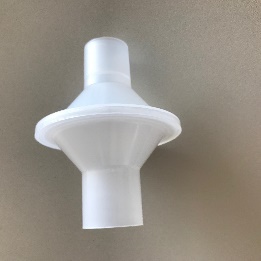


- Mouthpiece for each learner (optional)


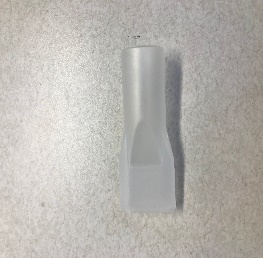


### Location:

- Conference room or unoccupied patient room

## Hands-On Learning Experience:

- Experience firsthand incentive spirometry
- Have learner stand, take 10 slow deep breaths keeping the indicator between the arrows, striving to achieve at least 1000 ml using
- Instructor to evaluate understanding and comprehension of the learner through discussion of key concepts

## Discussion of Key Concepts:

1. What are the different names for this type of therapy?

- IS therapy
- Incentive spirometer
- Maximal inspiration therapy
- Sustained maximal inspiration therapy

1. What are the goals of IS therapy?
   - Lung expansion and recruitment
   - Improved oxygenation
   - Good aeration across all lung fields
   - Aids in mobilizing secretions

| 1. What are indications and contraindications for IS therapy?    - Indications: abdominal surgery, thoracic surgery, atelectasis, restrictive lung disease limiting inspiratory effort, limited activity with risk of developing atelectasis or pneumonia    - Contraindications: Patient who are not able to coordinate slow, deep breathing through a mouthpiece with or without nose clips 2. Review initial treatment settings:    - Target volume should be around 14ml/kg    - Take slow deep breaths through mouth, trying to keep the indicator between the arrows, hold breath for 2-3 seconds then exhale    - Repeat for a total of 10 breaths    - May need to have patient pinch nose or use nose clips to ensure deep breathing is being coordinated through the mouth    - Encourage caregiver/parent to have patient do therapy in-between scheduled therapy 3. How to evaluate implementation and effectiveness of therapy  - Improved oxygenation - Improved CXR - Good breath sounds across lung fields  References Bylander LL. Foundations in Neonatal and Pediatric Respiratory Care: Airway clearance and lung expansion therapy. Burlington, MA: Jones & Bartlett Learning; 2019.  Walsh BK. Perinatal and Pediatric Respiratory Care: Airway clearance techniques and lung expansion. 3^rd^ ed. St. Louis, MO: Saunders Elsevier; 2010. 196-219 p. |
| --- |
