# Supplementary material for: Pediatric Resident Education in Pulmonary (PREP): A Subspecialty Preparatory Boot Camp Curriculum for Pediatric Residents
Source: MedEdPORTAL. 2021 Jan 7;17:11066. doi: 10.15766/mep_2374-8265.11066 (PMC7809931; doi:10.15766/mep_2374-8265.11066)
Supplement: Supplementary file 1 — Example Agenda.docxOrientation Template.pptxIntroduction to Tracheostomies and Ventilators.pptxCystic Fibrosis JeoPARODY.pptxIntroduction to Airway Clearance and Lung Expansion.pptxInstructor Guide CPT.docxInstructor Guide IS.docxInstructor Guide PEP.docxInstructor Guide PAP.docxInstructor Guide OPEP.docxInstructor Guide Insufflator Exsufflator.docxInstructor Guide HFCWO.docxInstructor Guide IPV.docxPREP Day of Evaluation.docxPREP End of Rotation Evaluation.docxPREP Faculty Feedback Survey.docxPREP Focus Group Guide.docx [file mep_2374-8265.11066-s001.zip › H. Instructor Guide PEP.docx]

# PREP Boot Camp Hands-On Session Airway Clearance and Lung Expansion Devices Instructor Guide: Positive Expiratory Pressure (PEP)

## Learning Objectives:

1. Describe what is a positive expiratory pressure therapy and how it works
2. Identify which patient population benefits from positive expiratory pressure therapy
3. Discuss how to evaluate the effectiveness of positive expiratory pressure therapy

Class Preparation:

### Equipment and Supplies:

- TheraPEP^TM^


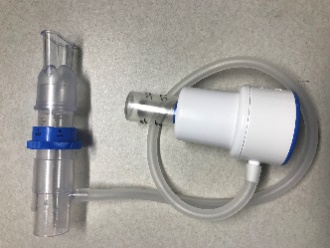


- Bacteria filter for each learner (required, can be used with or without mouthpiece)


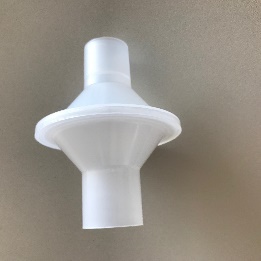


- Mouthpiece for each learner (optional)


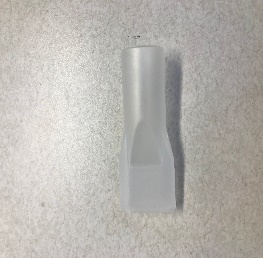


### Location:

- Conference room or unoccupied patient room

## Hands-On Learning Experience:

- Experience firsthand positive expiratory pressure therapy (TheraPEP)
- Have learner take 10 slow deep breaths with the PEP device, keeping the bellows between the two lines/arrows
- Instructor to evaluate understanding and comprehension of the learner through discussion of key concepts

## Discussion of Key Concepts:

1. What are the different names for this type of therapy?
   - Therapep^TM^
   - ThresholdPEP^TM^
   - PEP
   - Positive expiratory pressure therapy
2. What are the goals of PEP therapy?

- Lung expansion and recruitment
- Improved oxygenation
- Good aeration across all lung fields
- Aids in mobilizing secretions

| 1. What are indications and contraindications for PEP therapy?    - Able to coordinate deep breathing and adequate and consistent flow through the device (typically 3 years and older)    - Indications: abdominal surgery, thoracic surgery, atelectasis, patients who have failed to respond to incentive spirometry, chest physiotherapy, high frequency chest wall oscillation due to V/Q mismatching    - Contraindications: bullous emphysema, pneumothorax or pneumomediastinum, recent barotrauma 2. What are complications of PEP therapy?    - Hyperventilation    - Dizziness 3. Treatment Pearls:    - Sitting or standing    - Adjust dial for preferred resistance    - Place mouthpiece in mouth    - Take a deep breath    - Breath out slowly, keeping the bellows between the two lines/arrows    - 10 breaths per multiple cycles (no more than 5 cycles)    - Encourage caregiver/parent to have patient do therapy in-between scheduled therapy 4. How to evaluate implementation and effectiveness of therapy    - Achieve adequate airway clearance    - Improved CXR and breath sounds    - Improved oxygenation  References Bylander LL. Foundations in Neonatal and Pediatric Respiratory Care: Airway clearance and lung expansion therapy. Burlington, MA: Jones & Bartlett Learning; 2019.  Walsh BK. Perinatal and Pediatric Respiratory Care: Airway clearance techniques and lung expansion. 3^rd^ ed. St. Louis, MO: Saunders Elsevier; 2010. 196-219 p. |
| --- |
|  |
