# Supplementary material for: Pediatric Resident Education in Pulmonary (PREP): A Subspecialty Preparatory Boot Camp Curriculum for Pediatric Residents
Source: MedEdPORTAL. 2021 Jan 7;17:11066. doi: 10.15766/mep_2374-8265.11066 (PMC7809931; doi:10.15766/mep_2374-8265.11066)
Supplement: Supplementary file 1 — Example Agenda.docxOrientation Template.pptxIntroduction to Tracheostomies and Ventilators.pptxCystic Fibrosis JeoPARODY.pptxIntroduction to Airway Clearance and Lung Expansion.pptxInstructor Guide CPT.docxInstructor Guide IS.docxInstructor Guide PEP.docxInstructor Guide PAP.docxInstructor Guide OPEP.docxInstructor Guide Insufflator Exsufflator.docxInstructor Guide HFCWO.docxInstructor Guide IPV.docxPREP Day of Evaluation.docxPREP End of Rotation Evaluation.docxPREP Faculty Feedback Survey.docxPREP Focus Group Guide.docx [file mep_2374-8265.11066-s001.zip › I. Instructor Guide PAP.docx]

# PREP Boot Camp Hands-On Session Airway Clearance and Lung Expansion Devices Instructor Guide: Intermittent Positive Airway Pressure Therapy (PAP) Therapy

## Learning Objectives:

1. Describe what is a positive airway pressure therapy and how it works
2. Identify which patient population benefits from positive airway pressure therapy
3. Discuss how to evaluate the effectiveness of positive airway pressure therapy

Class Preparation:

### Equipment and Supplies:

- EzPAP^TM^


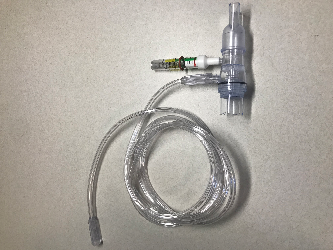


- Bacteria filter for each learner (required, can be used with or without mouthpiece)


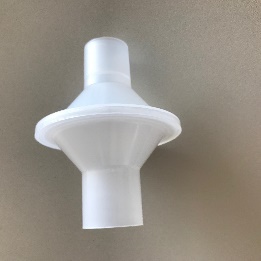


- Mouthpiece for each learner (optional)


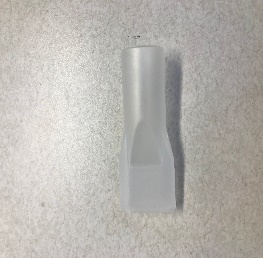


### Location:

- Conference room or unoccupied patient room

## Hands-On Learning Experience:

- Experience firsthand positive airway pressure (PAP) therapy
- Have learner take 10 slow deep breaths with EzPAP at 6 lpm air/oxygen then increase to 10 lpm air/oxygen and repeat the 10 breaths
- Instructor to evaluate understanding and comprehension of the learner through discussion of key concepts

## Discussion of Key Concepts:

1. What are the different names this type of therapy?
   - EzPAP^TM^
   - Intermittent positive airway pressure therapy
2. What are the goals of PAP therapy?

- Lung expansion and recruitment
- Improved oxygenation
- Aids in mobilizing secretions

| 1. How does PAP therapy work?    - On inspiration PAP provides additional flow and volume to enhance lung expansion    - On expiration continuous flow creates resistance to increase functional residual capacity and lung recruitment    - Studies have shown PAP therapy has greater effect on improving atelectasis than incentive spirometry 2. What are indications and contraindications for PAP therapy?    - Indications: abdominal surgery, thoracic surgery, atelectasis, patients who have failed to respond to incentive spirometry, chest physiotherapy, high frequency chest wall oscillation due to V/Q mismatching    - Contraindications: bullous emphysema, pneumothorax or pneumomediastinum, recent barotrauma, skull or facial trauma/surgery 3. What are complications of PAP therapy?    - Bronchospasms    - Gastric distention    - Hyperventilation    - Hypoventilation    - Baro/volutrauma with higher pressure    - Patient tolerance 4. Review initial treatment settings:    - Can be done with mouthpiece or mask    - Start at low flows of air/oxygen    - Gradually increase pressure to achieve desired expiratory pressures and patient’s tolerance    - 10 breaths with every cycle (3 is the standard) or 3 minutes of continual breathing    - Can be used as a standalone treatment typically every 4 hours; used in conjunction with airway clearance therapy; should not take a patient on non-invasive positive pressure ventilation    - Cannot be used in the home because it requires a high flow gas source and incorrect use can have clinical risks 5. How to evaluate implementation and effectiveness of therapy:    - Improved CXR and breath sounds    - Improved oxygenation  References Bylander LL. Foundations in Neonatal and Pediatric Respiratory Care: Airway clearance and lung expansion therapy. Burlington, MA: Jones & Bartlett Learning; 2019.  Walsh BK. Perinatal and Pediatric Respiratory Care: Airway clearance techniques and lung expansion. 3^rd^ ed. St. Louis, MO: Saunders Elsevier; 2010. 196-219 p.  Elliot S. A study to investigate the clinical use and outcomes of EzPAP positive pressure device. Thorax. 2011;66(A):96. |
| --- |
