# Supplementary material for: Pediatric Resident Education in Pulmonary (PREP): A Subspecialty Preparatory Boot Camp Curriculum for Pediatric Residents
Source: MedEdPORTAL. 2021 Jan 7;17:11066. doi: 10.15766/mep_2374-8265.11066 (PMC7809931; doi:10.15766/mep_2374-8265.11066)
Supplement: Supplementary file 1 — Example Agenda.docxOrientation Template.pptxIntroduction to Tracheostomies and Ventilators.pptxCystic Fibrosis JeoPARODY.pptxIntroduction to Airway Clearance and Lung Expansion.pptxInstructor Guide CPT.docxInstructor Guide IS.docxInstructor Guide PEP.docxInstructor Guide PAP.docxInstructor Guide OPEP.docxInstructor Guide Insufflator Exsufflator.docxInstructor Guide HFCWO.docxInstructor Guide IPV.docxPREP Day of Evaluation.docxPREP End of Rotation Evaluation.docxPREP Faculty Feedback Survey.docxPREP Focus Group Guide.docx [file mep_2374-8265.11066-s001.zip › J. Instructor Guide OPEP.docx]

# PREP Boot Camp Hands-On Session Airway Clearance and Lung Expansion Devices Instructor Guide: Oscillatory Positive Expiratory Pressure (OPEP) Devices

## Learning Objectives:

1. Describe what is oscillatory positive expiratory pressure therapy and how it works
2. Identify which patient population benefits from oscillatory positive expiratory pressure therapy
3. Discuss when to transition patient from oscillatory positive expiratory pressure therapy to other airway clearance devices

## Class Preparation:

### Equipment and Supplies:

- OPEP devices: Acapella^TM^ and/or Aerobika ^TM^

^
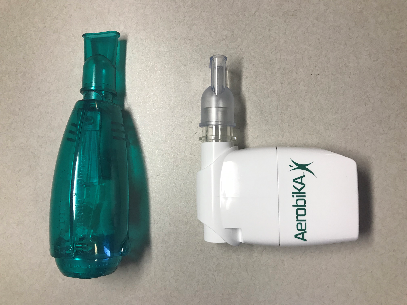
^

- Bacteria filter for each learner (required, can be reused on various equipment)


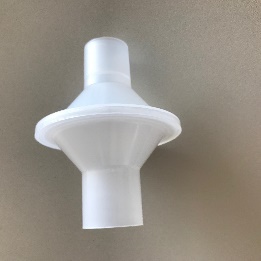


### Location:

- Conference room or unoccupied patient room

## Hands-On Learning Experience:

- This is where the learners can experience firsthand OPEP therapy
- Each learner should attempt 10 breaths on each of the OPEP therapy devices
- Instructor to evaluate understanding and comprehension of the learner through discussion of key concepts

## Discussion of Key Concepts:

1. What are the different names for this type of therapy?
   - Acapella^TM^
   - Aerobika^TM^
   - OPEP
   - Flutter^TM^
   - Oscillatory positive expiratory pressure therapy
   - Oscillatory therapy
2. What are the goals of OPEP?

- Lung expansion and recruitment
- Improved oxygenation
- Good aeration across all lung fields
- Aids in mobilizing secretions

1. How does OPEP work in assisting in airway clearance and lung recruitment?
   - Provides back pressure to increase airway resistance on expiration and improve lung expansion
   - Use oscillation to sheer mucus from the airways and aid in the mobilization into larger airways
2. What are indications and contraindications for OPEP?
   - Able to coordinate deep breathing and adequate flow through the device (typically 5 years and older)
   - Indications: cystic fibrosis, bronchiectasis, pneumonia, hypoxemia in the face of V/Q mismatch
   - Contraindications: sinusitis, pneumothorax, pneumomediastinum, lobectomy, lung abscess, hemodynamic instability, middle ear infection, acute increased work of breathing
3. What are adverse consequences of OPEP?
   - Shortness of breath
   - Dizziness
   - Hyperventilating
4. What is the therapy cycle?
   - Sitting or standing
   - Adjust dial for preferred resistance
   - Take a deep breath
   - Breath out through the mouth at moderate rate
   - 10 breaths per multiple cycles (no more than 5 cycles)
   - Encourage caregiver/parent to have child do therapy in-between scheduled therapy
5. How to evaluate implementation and effectiveness of therapy
   - Achieve adequate airway clearance
   - Improved CXR
   - Improved oxygenation
   - Improved aeration across lung fields
6. Considerations for ordering for home use
   - OPEP is typically not covered by insurance
   - Families can purchase products through online medical stores

## References

Bylander LL. Foundations in Neonatal and Pediatric Respiratory Care: Airway clearance and lung expansion therapy. Burlington, MA: Jones & Bartlett Learning; 2019.

Walsh BK. Perinatal and Pediatric Respiratory Care: Airway clearance techniques and lung expansion. 3^rd^ ed. St. Louis, MO: Saunders Elsevier; 2010. 196-219 p.
