# Supplementary material for: Pediatric Resident Education in Pulmonary (PREP): A Subspecialty Preparatory Boot Camp Curriculum for Pediatric Residents
Source: MedEdPORTAL. 2021 Jan 7;17:11066. doi: 10.15766/mep_2374-8265.11066 (PMC7809931; doi:10.15766/mep_2374-8265.11066)
Supplement: Supplementary file 1 — Example Agenda.docxOrientation Template.pptxIntroduction to Tracheostomies and Ventilators.pptxCystic Fibrosis JeoPARODY.pptxIntroduction to Airway Clearance and Lung Expansion.pptxInstructor Guide CPT.docxInstructor Guide IS.docxInstructor Guide PEP.docxInstructor Guide PAP.docxInstructor Guide OPEP.docxInstructor Guide Insufflator Exsufflator.docxInstructor Guide HFCWO.docxInstructor Guide IPV.docxPREP Day of Evaluation.docxPREP End of Rotation Evaluation.docxPREP Faculty Feedback Survey.docxPREP Focus Group Guide.docx [file mep_2374-8265.11066-s001.zip › K. Instructor Guide Insufflator Exsufflator.docx]

# Pediatric Resident Education in Pulmonary (PREP) Boot Camp Hands-On Session: Airway Clearance and Lung Expansion Devices Instructor Guide: Insufflator-Exsufflator (Cough Assist)

## Learning Objectives:

1. Describe what is an insufflator-exsufflator device and how it works
2. Identify which patient population benefits from insufflator-exsufflator therapy
3. Discuss appropriate treatment settings and modifications to support adequate cough augmentation

Class Preparation:

### Equipment and Supplies:

- Insufflator-exsufflator machine (Cough Assist T70 device)

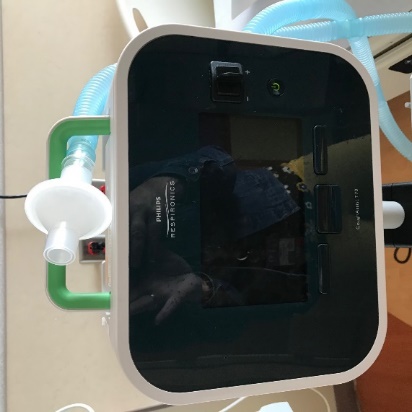

- Tubing for machine
- Bacterial filter for each learner (required, can be used with or without mouthpiece)

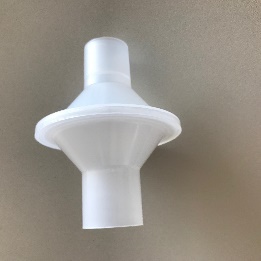

- Oxygen bleed in adapter
- Mouthpiece for each learner (optional)


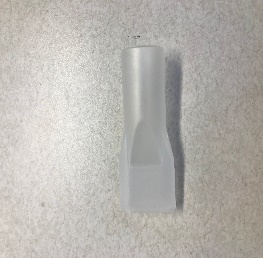

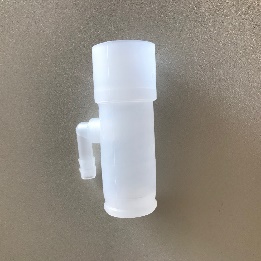


### Location: Conference room or unoccupied patient room

### Learner Settings:

Mode: Auto

Cough-Trak: On

Inhale Pressure: 20 cmH20

Inhale Flow: Medium

Inhale Time: 3.0 sec

Exhale Pressure: -20 cmH20

Exhale Time: 2.0 sec

Oscillation: Exhale

Frequency: 10 Hz

Amplitude: 5 cmH20

## Hands-On Learning Experience:

- This is where the learners can experience firsthand insufflator-exsufflator therapy
- Have learner pinch their nose, place the bacteria filter attached to the circuit in their mouth, relax as much as possible, and breath in (triggering the device)
- Each learner should attempt one therapy cycle (5 breaths)
- Instructor to evaluate understanding and comprehension of the learner through discussion of key concepts

## Discussion of Key Concepts:

1. What are the different names for this type of therapy?
   - Cough assist^TM^
   - Insufflator-exsufflator
   - Cough therapy
   - Cough augmentation
   - Mechanical insufflator/exsufflator (MI-E)
2. What are the goals of cough assist?
   - Prevent accumulation of secretions
   - Mobilizing secretions
   - Mimic cough
3. What is the benefit of a cough?
   - Reflex that helps protect the airway and lungs against irritants
   - Key way to remove secretions from the airway
4. What are the different cycles of a cough?

- Inspiration up to 85–90% of total lung capacity
- Rapid closure of the glottis for ~0.2 sec
- Forced expiration by the abdominal muscles, intercostal muscles contracting forcibly, pushing against the diaphragm resulting intrapleural pressures of >190 cmH20 or expiratory force of 75-100 miles per hour
- Peak cough flows 360-1,200 LPM
- Inspiration
- Example white board drawing demonstrating this concept here:


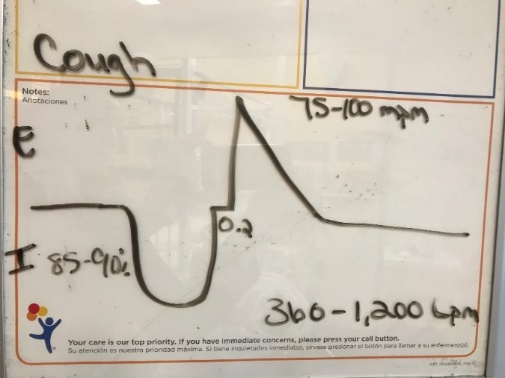


| 1. What are indications and contraindications for cough augmentation with the in-exsufflator?    - Indications: patient with ineffective cough due to neuromuscular disease, restrictive lung disease with ineffective cough, need for bronchial hygiene in an invasively ventilated patient with poor cough effort.    - Contraindications: bullous emphysema, pneumothorax or pneumomediastinum, recent barotrauma, skull or facial trauma/surgery, known or suspected tympanic membrane rupture 2. What are complication consequences from cough augmentation?    - Baro/volutrauma    - Tympanic membrane rupture    - Gastric insufflation 3. Review initial treatment settings:    - Treatment is done with appropriate size full face mask to achieve a good seal    - Presets: allow for different settings under each number    - Mode: auto cycles with the patient’s inspiratory effort; manual requires health care provider to initiate inspiration    - Cough Trak: allows synchronization with patient effort    - Inhale pressure-starting pressure at 30 cmH20    - Inhale Flow: adjustment of inspiratory flow, used for patient comfort    - Inhale time:1-3 sec (children, adolescent can be 3-6 sec) based on effectiveness of therapy and patient tolerance; should be slightly longer than exhalation to maintain Functional Residual Capacity    - Exhale pressure-starting pressure at -30cmH20    - Exhale time 1-3 sec (children, adolescent can be 3-6 sec) based on effectiveness of therapy and patient tolerance    - Oscillation: enhances mobilization of secretions    - Frequency: how rapidly the oscillations occur (15 Hz=900 per minute)    - Amplitude: change in pressure within each oscillation    - Oxygen: Bleed in distal or proximal to the patient 4. Recognize inspiration and expiration of breath from main screen    - Inspiration demonstrated here:   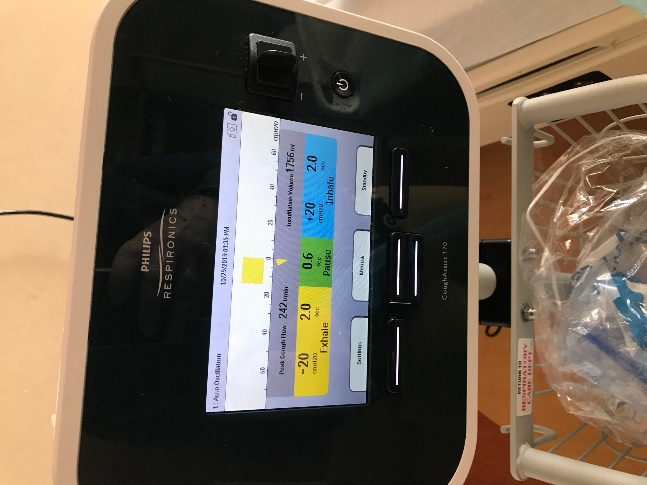 |
| --- |
| \| 1. What is Cough Peak Flow (CPF)    - Directly assess bulbar-innervated muscle function    - Should be based on gender, height, and body mass    - CPF <270L/m suggest use of cough assist device    - CPF<160lpm can be associated with extubation failure 2. Understands the sequence and frequency of therapy regiment    - Initiation of first breath cycle (5 breaths, end on inspiration, suction naso-pharynx/mouth/airway)    - Allow time between therapy for hyper oxygenation and ventilation    - Repeat for a total of three to six breath cycles    - Frequency for hospital should be no more often Q2 (in the ICU setting) and no less than Q6    - Home regiment should be BID when healthy, increase to TID or QID when sick 3. How to evaluate implementation and effectiveness of therapy    - Cough peak flow ≥300 lpm    - Achieve adequate airway clearance    - Improved CXR and breath sounds    - Appropriate oxygenation-ensure oxygen is being bled in during therapy 4. Discuss options for adjusting settings for optimal therapy    - Bedside respiratory therapist should be comfortable assessing then providing follow up to the medical team    - Hypoxia-ensure oxygen is being bled into the cough assist circuit    - Not getting enough secretions with minimal to no change to clinical presentation-consider increasing pressure, starting mucolytic    - Asynchrony-consider changing a different mode, adjust inspiration or expiration time 5. Understand limitations to therapy in patients with artificial airway (endotracheal tube or tracheostomy tube)    - Patient is on high levels of Peep    - Should not be placed in line with ventilator circuit    - Differences in airway clearance between cuffed and uncuffed artificial airway    - Do not use in line with ventilator circuit 6. Considerations for ordering cough assist for home use  - Indication: Cough Assist device is indicated for any patients unable to cough or clear secretions effectively due to reduced peak cough expiratory flow, resulting from high spinal cord injuries, neuromuscular deficits or severe fatigue associated with intrinsic lung diseases where other airway clearance devices have failed (defined by hospitalizations or reoccurring pneumonias) - Insurance companies will only rent/purchase on airway clearance device every five year - Options for multiple presets for home management. Example: Preset 1: Inspiratory pressure with suction. Preset 2: inspiratory and expiratory pressures. Preset 3: Inspiratory and expiratory pressure with oscillation. \| \| --- \| |

## *All photos courtesy of Joyce Baker, MBA RRT-NPS

## References

Bylander LL. Foundations in Neonatal and Pediatric Respiratory Care: Airway clearance and lung expansion therapy. Burlington, MA: Jones & Bartlett Learning; 2019.

Walsh BK. Perinatal and Pediatric Respiratory Care: Airway clearance techniques and lung expansion. 3^rd^ ed. St. Louis, MO: Saunders Elsevier; 2010. 196-219 p.

Abusamra R, Russell RR. Management of respiratory disease in children with muscular weakness. Paediatrics and Child Health. 2015 Jul;25(11):515-521.

Bianchi C, Baiardi P. Cough peak flows: Standard values for children and adolescents. Am J Phys Med Rehabil. 2008 Jun;87(6):461-7.

Rafiq MK, Bradburn M, Mustfa N, Mcdermott CJ, Annane D. Mechanical cough augmentation techniques in amyotrophic lateral sclerosis/motor neuron disease. Cochrane Database of Systematic Reviews. 2016 Dec 23;12.

Rokadia HK, Adams JR, McCarthy K, Aboussousan LS, Mireles-Cabodevila E. Cough augmentation in a patient with neuromuscular disease. Ann Am Thorac Soc. 2015 Dec;12(12):1888-91.

Siriwat R, Deerojanawong J, Sritippayawan S, Hantragool S, Cheanprapai P. Mechanical insufflation-exsufflation versus conventional chest physiotherapy in children with cerebral palsy. Respir Care. 2018 Feb;63(2):187-193.
