# Supplementary material for: Pediatric Resident Education in Pulmonary (PREP): A Subspecialty Preparatory Boot Camp Curriculum for Pediatric Residents
Source: MedEdPORTAL. 2021 Jan 7;17:11066. doi: 10.15766/mep_2374-8265.11066 (PMC7809931; doi:10.15766/mep_2374-8265.11066)
Supplement: Supplementary file 1 — Example Agenda.docxOrientation Template.pptxIntroduction to Tracheostomies and Ventilators.pptxCystic Fibrosis JeoPARODY.pptxIntroduction to Airway Clearance and Lung Expansion.pptxInstructor Guide CPT.docxInstructor Guide IS.docxInstructor Guide PEP.docxInstructor Guide PAP.docxInstructor Guide OPEP.docxInstructor Guide Insufflator Exsufflator.docxInstructor Guide HFCWO.docxInstructor Guide IPV.docxPREP Day of Evaluation.docxPREP End of Rotation Evaluation.docxPREP Faculty Feedback Survey.docxPREP Focus Group Guide.docx [file mep_2374-8265.11066-s001.zip › L. Instructor Guide HFCWO.docx]

# PREP Boot Camp Hands-On Session Airway Clearance and Lung Expansion Devices Instructor Guide: High Frequency Chest Wall Oscillation (HFCWO)

## Learning Objectives:

1. Describe what is high frequency chest wall oscillation therapy and how it works
2. Identify which patient population benefits from high frequency chest wall oscillation therapy
3. Discuss appropriate treatment settings and modifications for adequate high frequency chest wall oscillation therapy

## Class Preparation:

### Equipment and Supplies:

### High frequency chest wall oscillation (HFCWO) device

###
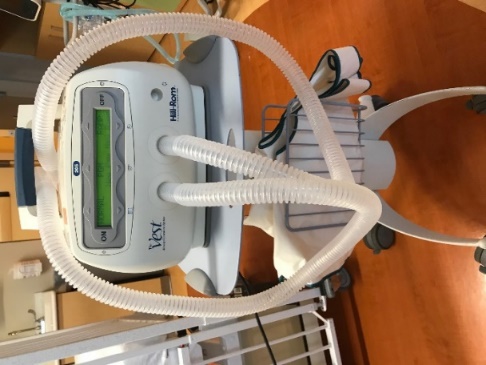


- Air hose specific to the HFCWO device
- Medium size vest or wrap

### Location:

- Conference room or unoccupied patient room

### Learner Settings (Hill-Rom 205):

- - Mode: Normal
  - Frequency: 10 Hz
  - Pressure: 5 cmH20
  - Time: 1 minute


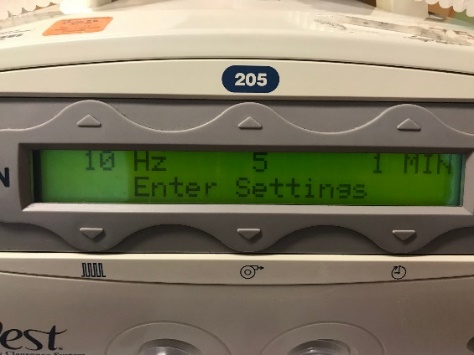


## Hands-On Learning Experience:

- Experience firsthand HFCWO therapy
- Each learner should attempt 1 minute of HFCWO therapy using a vest or wrap device
- Instructor to evaluate understanding and comprehension of the learner through discussion of key concepts

## Discussion of Key Concepts:

1. What is HFCWO?

- Airway clearance technique using external chest wall oscillations via inflatable vest or wrap worn on the torso
- Create indirect positive and negative pressure changes in the lungs helping to mobilize secretions
- Loosens secretions in the smaller airways, oscillating them towards the larger airways

1. What are the different names for this type of therapy?

- Vest therapy
- High frequency chest wall oscillation therapy
- HFCWO
- Vest Airway Clearance System ^TM^
- inCourage System ^TM^
- SmartVest ^TM^
- AffloVest ^TM^

1. What is the goal of HFCWO?
   - Mobilizing secretions
2. What are indications and contraindications for HFCWO?
   - Indications: patient with ineffective secretion clearance due to bronchitis, bronchiectasis, cystic fibrosis, neuromuscular disorder which produce pulmonary symptoms
   - Contraindications: pneumothorax, hemoptysis, elevated intracranial pressures, hemodynamically unstable, pulmonary embolism, active tuberculosis, fractured ribs or unstable chest, head or neck injury not stabilized
3. What are complications from HFCWO?
   - Tolerance
   - Discomfort
   - G-Tube pressure injury

| 1. Review initial HFCWO treatment settings:    - Cystic Fibrosis      1. Mode: Normal      2. Treatment Regiment: 4 cycles of 5 minutes’ increments per cycle. Increasing Hz with each cycle 10, 11, 12, 13      3. Huff coughing after each cycle      4. Can be taking in conjunction with Inhaled hypertonic treatment      5. Treatment frequency:         1. Home: BID or increase TID if sick         2. Hospital: TID or QID    - Other patients      1. Mode: Normal      2. Treatment Regiment: 1 cycle, 10-20 minutes      3. Oral, nasal, or deep suctioning after each treatment      4. Treatment frequency: QID, Q4, Q6 |
| --- |
| \| 1. Understand limitations to ordering HFCWO for home use:    - - Cystic fibrosis    - Documentation of failure of standard treatments (CPT, OPEP)      - Bronchiectasis confirmed with CT    - Daily cough for at least 6 months    - Frequency exacerbations requiring antibiotics within the year    - Documentation of failure of standard treatments (CPT, OPEP)  - Other diagnosis - Ineffective airway clearance - Documentation of multiple hospitalizations/ED visits - Recurrent pneumonias   - Documentation of failure of standard treatments (CPT, OPEP) \| \| --- \| \| References Bylander LL. Foundations in Neonatal and Pediatric Respiratory Care: Airway clearance and lung expansion therapy. Burlington, MA: Jones & Bartlett Learning; 2019.  Walsh BK. Perinatal and Pediatric Respiratory Care: Airway clearance techniques and lung expansion. 3^rd^ ed. St. Louis, MO: Saunders Elsevier; 2010. 196-219 p.  Mcllwaine M, Bradley J, Elborn JS, Moran F. Personalising airway clearance in chronic lung disease. Eur Respir Rev. 2017; 26(143):160086.  Snijders D, Fernandez Dominguez B, Calgaro S, Bertozzi I, Escribano Montaner A, erilongo G, Barbato A. Mucocilliary clearance techniques for treating non-cystic fibrosis bronchiectasis: Is there evidence? Int J Immunopathol Pharmacol. 2015;28(2):150-9. \| |
