# Supplementary material for: Pediatric Resident Education in Pulmonary (PREP): A Subspecialty Preparatory Boot Camp Curriculum for Pediatric Residents
Source: MedEdPORTAL. 2021 Jan 7;17:11066. doi: 10.15766/mep_2374-8265.11066 (PMC7809931; doi:10.15766/mep_2374-8265.11066)
Supplement: Supplementary file 1 — Example Agenda.docxOrientation Template.pptxIntroduction to Tracheostomies and Ventilators.pptxCystic Fibrosis JeoPARODY.pptxIntroduction to Airway Clearance and Lung Expansion.pptxInstructor Guide CPT.docxInstructor Guide IS.docxInstructor Guide PEP.docxInstructor Guide PAP.docxInstructor Guide OPEP.docxInstructor Guide Insufflator Exsufflator.docxInstructor Guide HFCWO.docxInstructor Guide IPV.docxPREP Day of Evaluation.docxPREP End of Rotation Evaluation.docxPREP Faculty Feedback Survey.docxPREP Focus Group Guide.docx [file mep_2374-8265.11066-s001.zip › M. Instructor Guide IPV.docx]

# PREP Boot Camp Hands-On Session Airway Clearance and Lung Expansion Devices Instructor Guide: Intrapulmonary Percussive Ventilation (IPV)

## Learning Objectives:

1. Describe what is intrapulmonary percussive ventilation therapy and how it works
2. Identify which patient population benefits from intrapulmonary percussive ventilation therapy
3. Discuss appropriate treatment settings and modifications for adequate intrapulmonary percussive ventilation therapy

Class Preparation:

### Equipment and Supplies:

- Intrapulmonary percussive ventilation machine


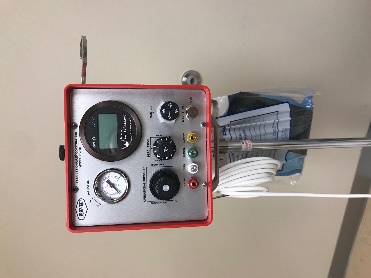


- Tubing/circuit for IPV
- Bacterial filter for each learner (required, can be used with or without mouthpiece)


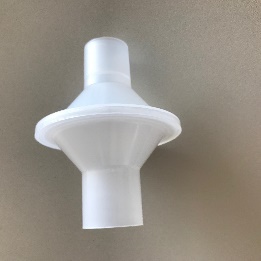


- Mouthpiece for each learner (optional)


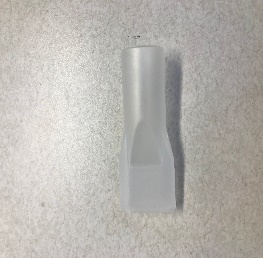


### Location: Conference room or unoccupied patient room

### Learner Settings (Percussionaire ^TM^ IPV-C)

Operational Pressure: 20 cmH20

Percussion: 5 Hz


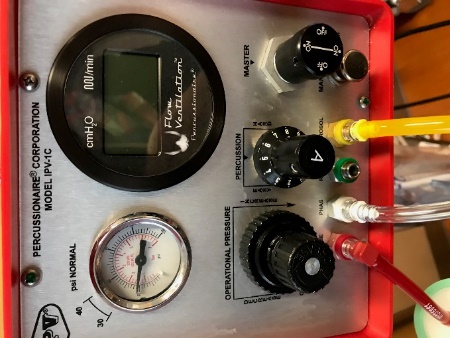


## Hands-On Learning Experience:

- Experience firsthand IPV therapy
- Use proper IPV technique: pinch their nose, place the bacteria filter attached to the circuit in their mouth, and breathe in and out through their mouth
- Each learner should attempt 1 minute of IPV therapy
- Instructor to evaluate understanding and comprehension of the learner through discussion of key concepts

## Discussion of Key Concepts:

1. What is IPV?
   - Intermittent pneumatic positive pressure device
   - Assists in airway clearance
   - Delivers small bursts of flow at a high rate
   - Mobilizes endobronchial secretions and diffuse patchy atelectasis

| 1. What are different names or devices to deliver IPV?    - Intermittent percussive ventilation    - Meta-Neb ^TM^    - Intrapulmonary percussive ventilation 2. What are indications and contraindications for IPV?    - Indications: spontaneously breathing patient with ineffective secretion clearance due to bronchitis, bronchiectasis, cystic fibrosis, neuromuscular disorder    - Contraindications: pneumothorax, hemoptysis, active tuberculosis, fractured ribs or unstable chest, patient is on inhaled nitric oxide 3. What are possible complications from IPV?    - Hyperventilation    - Gastric insufflation    - Decrease cardiac output    - Increased intracranial pressures    - Barotrauma or volutrauma 4. Treatment Pearls:    - Treatment is done with mouthpiece and nose clips, or airway adapter    - Hypertonic saline is often administered through IPV    - Patient controls variables such as inspiratory time, delivery rates and peak pressure    - Initial treatment – start at low operational pressure (20 cH20) and gradually increase to obtain good chest wiggle (no more than 40 cmH20)    - Adjust percussion (Hz) to patient comfort, chest wiggle, and optimal secretion removal    - Treatment cycle is 3-5 minutes for a total 3-4 cycles |
| --- |
| 1. Special considerations in patients with artificial airway (endotracheal tube or tracheostomy tube):    - Can be done either as a standalone treatment (taking patient off the ventilator) or in line with the ventilator if using a one-way valve    - Patients requiring higher levels of PEEP and Fi02 may not tolerating being taken off the ventilator for IPV therapy    - If artificial airway has a cuff, it needs to be deflated. This allows for the airway to remain patent during the IPV treatment. 2. Consideration for home use:    - - Rarely approved by insurance, and if so, only in bronchiectasis (i.e. cystic fibrosis) and after documentation why other therapy modalities have failed  References Bylander LL. Foundations in Neonatal and Pediatric Respiratory Care: Airway clearance and lung expansion therapy. Burlington, MA: Jones & Bartlett Learning; 2019.  Walsh BK. Perinatal and Pediatric Respiratory Care: Airway clearance techniques and lung expansion. 3^rd^ ed. St. Louis, MO: Saunders Elsevier; 2010. 196-219 p.  Mcllwaine M, Bradley J, Elborn JS, Moran F. Personalising airway clearance in chronic lung disease. Eur Respir Rev. 2017;26(143):160086.  [Dingemans J](https://www.ncbi.nlm.nih.gov/pubmed/?term=Dingemans%20J%5BAuthor%5D&cauthor=true&cauthor_uid=29560543), Eyns H, Willekens J, Monsieurs P, Van Houdt R, Cornelis P, Malfroot A, Crabbe, A. Intrapulmonary percussive ventilation improves lung in cystic fibrosis patients chronically colonized with Pseudomonas aeruginosa: a pilot cross-over study. Eur J Clin Microbiol Infect Dis. 2018 Jun;37(6):1143-1151. |
