# Supplementary material for: Pediatric Resident Education in Pulmonary (PREP): A Subspecialty Preparatory Boot Camp Curriculum for Pediatric Residents
Source: MedEdPORTAL. 2021 Jan 7;17:11066. doi: 10.15766/mep_2374-8265.11066 (PMC7809931; doi:10.15766/mep_2374-8265.11066)
Supplement: Supplementary file 1 — Example Agenda.docxOrientation Template.pptxIntroduction to Tracheostomies and Ventilators.pptxCystic Fibrosis JeoPARODY.pptxIntroduction to Airway Clearance and Lung Expansion.pptxInstructor Guide CPT.docxInstructor Guide IS.docxInstructor Guide PEP.docxInstructor Guide PAP.docxInstructor Guide OPEP.docxInstructor Guide Insufflator Exsufflator.docxInstructor Guide HFCWO.docxInstructor Guide IPV.docxPREP Day of Evaluation.docxPREP End of Rotation Evaluation.docxPREP Faculty Feedback Survey.docxPREP Focus Group Guide.docx [file mep_2374-8265.11066-s001.zip › N. PREP Day of Evaluation.docx]

**Pediatric Resident Education in Pulmonary (PREP) Boot Camp Evaluation**

We’re glad you’re here! This survey will have no impact on your evaluations. It is anonymous and completely voluntary. Please answer honestly!

**How prepared do you feel to take your first night of call on inpatient pulmonary?**

Extremely prepared Very prepared Somewhat prepared Not so prepared Not at all prepared

5 4 3 2 1

**Pediatric Resident Education in Pulmonary (PREP) Boot Camp Evaluation**

We’re glad you’re here! This survey will have no impact on your evaluations. It is anonymous and completely voluntary. Please answer honestly!

**How prepared do you feel to take your first night of call on inpatient pulmonary?**

Extremely prepared Very prepared Somewhat prepared Not so prepared Not at all prepared

5 4 3 2 1

**How helpful did you find PREP?**

Extremely helpful Very helpful Somewhat helpful Not so helpful Not at all helpful

5 4 3 2 1

**What was the BEST part of PREP?**

**What was the WORST part of PREP?**

**How could we improve PREP?**

How important was the **Orientation to Inpatient Pulmonary** for your overall learning during PREP?

Extremely important Very important Somewhat important Not so important Not at all important

5 4 3 2 1

How important was the **Trach/Vent Lecture** for your overall learning during PREP?

Extremely important Very important Somewhat important Not so important Not at all important

5 4 3 2 1

How important was the **Trach/Vent Simulation Session** for your overall learning during PREP?

Extremely important Very important Somewhat important Not so important Not at all important

5 4 3 2 1

How important was the **CF JeoPARODY Session** for your overall learning during PREP?

Extremely important Very important Somewhat important Not so important Not at all important

5 4 3 2 1

How important was the **Intro to Airway Clearance and Lung Expansion Lecture** for your overall learning during PREP?

Extremely important Very important Somewhat important Not so important Not at all important

5 4 3 2 1

How important was the **Airway Clearance Hands-On Session** for your overall learning during PREP?

Extremely important Very important Somewhat important Not so important Not at all important

5 4 3 2 1

**Any additional comments or feedback?**
