# Supplementary material for: Pediatric Resident Education in Pulmonary (PREP): A Subspecialty Preparatory Boot Camp Curriculum for Pediatric Residents
Source: MedEdPORTAL. 2021 Jan 7;17:11066. doi: 10.15766/mep_2374-8265.11066 (PMC7809931; doi:10.15766/mep_2374-8265.11066)
Supplement: Supplementary file 1 — Example Agenda.docxOrientation Template.pptxIntroduction to Tracheostomies and Ventilators.pptxCystic Fibrosis JeoPARODY.pptxIntroduction to Airway Clearance and Lung Expansion.pptxInstructor Guide CPT.docxInstructor Guide IS.docxInstructor Guide PEP.docxInstructor Guide PAP.docxInstructor Guide OPEP.docxInstructor Guide Insufflator Exsufflator.docxInstructor Guide HFCWO.docxInstructor Guide IPV.docxPREP Day of Evaluation.docxPREP End of Rotation Evaluation.docxPREP Faculty Feedback Survey.docxPREP Focus Group Guide.docx [file mep_2374-8265.11066-s001.zip › O. PREP End of Rotation Evaluation.docx]

PREP End of Rotation Evaluation

Thank you for rotating with us on pulmonary and for participating in the Pediatric Resident Education in Pulmonary (PREP) Boot Camp. We would love your feedback on your experience. This survey will have no impact on your evaluations. It is anonymous and completely voluntary.

1. What program are you in?
   1. Categorical Pediatrics
   2. Medicine/Pediatrics
   3. Other combined Pediatrics
2. What are your career plans following graduation?
   1. Outpatient General Practice
   2. Hospitalist/Academic Medicine
   3. Subspecialty/Fellowship
   4. Undecided
3. If subspecialty/fellowship, which?
4. How did the inpatient pediatric pulmonary rotation and PREP influence your career choice?
   1. No change in my career choice
   2. Increased likelihood of pursuing pediatric pulmonology as a career
   3. Decreased likelihood of pursuing pediatric pulmonology as a career
   4. Confirmed my choice in pediatric pulmonology as a career
5. What was the best part of your pulmonary rotation?
6. What was the worst part of your pulmonary rotation?
7. Thinking back to your first night of call, how prepared did you feel in taking care of the service?
   1. Extremely prepared
   2. Very prepared
   3. Somewhat prepared
   4. Not so prepared
   5. Not at all prepared
8. How helpful was PREP Boot Camp?
   1. Extremely helpful
   2. Very helpful
   3. Somewhat helpful
   4. Not so helpful
   5. Not at all helpful
9. Please give an example of something you learned during PREP Boot Camp that you used during your inpatient pulmonary rotation, or beyond.
10. How can the inpatient pediatric pulmonology rotation or PREP Boot Camp be improved?
