# Supplementary material for: Pediatric Resident Education in Pulmonary (PREP): A Subspecialty Preparatory Boot Camp Curriculum for Pediatric Residents
Source: MedEdPORTAL. 2021 Jan 7;17:11066. doi: 10.15766/mep_2374-8265.11066 (PMC7809931; doi:10.15766/mep_2374-8265.11066)
Supplement: Supplementary file 1 — Example Agenda.docxOrientation Template.pptxIntroduction to Tracheostomies and Ventilators.pptxCystic Fibrosis JeoPARODY.pptxIntroduction to Airway Clearance and Lung Expansion.pptxInstructor Guide CPT.docxInstructor Guide IS.docxInstructor Guide PEP.docxInstructor Guide PAP.docxInstructor Guide OPEP.docxInstructor Guide Insufflator Exsufflator.docxInstructor Guide HFCWO.docxInstructor Guide IPV.docxPREP Day of Evaluation.docxPREP End of Rotation Evaluation.docxPREP Faculty Feedback Survey.docxPREP Focus Group Guide.docx [file mep_2374-8265.11066-s001.zip › P. PREP Faculty Feedback Survey.docx]

PREP Faculty Feedback Survey

The Pediatric Resident Education in Pulmonary (PREP) Boot Camp is a half day educational activity on the residents' first day of inpatient pulmonary. The residents learn the basics about trachs/vents, CF, and airway clearance during PREP. Meanwhile, the inpatient service is covered by outstanding Advanced Practice Providers (APPs), fellows, post-call residents, and willing faculty who opted in to participate. Please provide your honest feedback below - it is much appreciated!

1. How often have you been on the inpatient pulmonary service since implementation of PREP?
   1. 0 weeks
   2. 1-2 weeks
   3. 3-4 weeks
   4. >5 weeks
2. How valuable is PREP for the residents’ learning experience?
   1. Extremely valuable
   2. Very valuable
   3. Somewhat valuable
   4. Not so valuable
   5. Not at all valuable
   6. Decline to comment
3. What skills have the residents gained as a result of PREP? Have you noticed any actions that the residents are able to do since implementation of PREP that they were not able to do prior? Please describe here.
4. What can be improved about PREP?
