# Supplementary material for: Pediatric Resident Education in Pulmonary (PREP): A Subspecialty Preparatory Boot Camp Curriculum for Pediatric Residents
Source: MedEdPORTAL. 2021 Jan 7;17:11066. doi: 10.15766/mep_2374-8265.11066 (PMC7809931; doi:10.15766/mep_2374-8265.11066)
Supplement: Supplementary file 1 — Example Agenda.docxOrientation Template.pptxIntroduction to Tracheostomies and Ventilators.pptxCystic Fibrosis JeoPARODY.pptxIntroduction to Airway Clearance and Lung Expansion.pptxInstructor Guide CPT.docxInstructor Guide IS.docxInstructor Guide PEP.docxInstructor Guide PAP.docxInstructor Guide OPEP.docxInstructor Guide Insufflator Exsufflator.docxInstructor Guide HFCWO.docxInstructor Guide IPV.docxPREP Day of Evaluation.docxPREP End of Rotation Evaluation.docxPREP Faculty Feedback Survey.docxPREP Focus Group Guide.docx [file mep_2374-8265.11066-s001.zip › Q. PREP Focus Group Guide.docx]

**Pediatric Resident Education in Pulmonary (PREP) Boot Camp Focus Group Guide**

**Broad Initial Topics**

- Resident overall impression of PREP
- Resident experience caring for pulmonary patients daily
- Perceived changes or improvements in residents’ ability to provide care after PREP
- Perceived resident confidence and knowledge in caring for patients with tracheostomies or CF
- Experiences during acute events such as RRTs or codes on patients with tracheostomies or CF

**Sample questions**

1. Let’s start with some general information. Please state…
   1. Your program (Pediatrics, Med/Peds, other)
   2. What month you rotated through inpatient pulmonary
   3. And, in general, what was your experience during your inpatient pulmonary month?
2. For those of you who have rotated through the Pediatric Intensive Care Unit (PICU) or Hematology/Oncology Unit, how was the transition to inpatient pulmonary compared to those other rotations?
   1. What was different (better/worse) about pulmonary?
   2. What was the most helpful aspect of a transition to any rotation?
3. Think about an acute event, such as a code or a rapid response event, on a pulmonary patient where you assisted during the event. Please describe the event and what role you played.
   1. How did PREP prepare you for the event? Anything you learned during PREP that helped you during the acute event?
   2. How were you successful? Where did you struggle?
4. Think about when you cared for a patient with a tracheostomy during your inpatient pulmonary month.
   1. What was your comfort level with patients with tracheostomies?
   2. Did PREP help you feel more or less comfortable caring for these patients?
5. How can we improve the residents’ educational experience during inpatient pulmonary?
   1. What areas did you struggle with?
   2. What else should we be teaching during PREP and beyond?
   3. Would videos or other online resources be helpful? Would you review a video of any information you learned during PREP later (during your pulmonary month or beyond)?
6. Anything else?

Additional probing questions will be developed during the focus groups by the facilitator.  In addition, debriefing should occur with the investigators after each focus group and the questions modified for subsequent focus groups.
